# Abstracts of the 12^th^ European Cytogenomics Conference 2019: Salzburg, Austria. 6-9 July 2019

**DOI:** 10.1186/s13039-019-0439-z

**Published:** 2019-07-05

**COMMITTEES**

**E.C.A. Committee**

Mariano ROCCHI - President

Kamlesh MADAN - First Vice-President

Pat HESLOP - HARRISON - Second Vice-President

Konstantin MILLER - General Secretary

Jean-Michel DUPONT - Treasurer

**Scientific Programme Committee**

Mariano ROCCHI - Chair

Claudia HAFERLACH

Dieter KOTZOT

Damien SANLAVILLE

Joris VERMEESCH

Orsetta ZUFFARDI

**Organizing Secretariat**

DEKON Congress & Tourism

Sultan Selim Mah. Eski Büyükdere Cad. Hümeyra Sok. No.12

34415 Seyrantepe - Kağıthane - Istanbul - TURKEY

Telephone: +90 212 347 63 00

Fax: +90 212 347 63 63

E-mail: eca2019@eca2019.com or dekon@dekon.com.tr

Visit www.dekon.com.tr for more information

**Table:** 

**Scientific Programme**	
**SATURDAY, 6 July 2019**	
14:00-17:00	Permanent Working Groups
17:00-17:50	Symposium: Molecular Cytogenetics (BMC-Springer/Nature) symposium dedicated to the memory of Prof. Yuri Yurov
18:00-19:00	**Opening lecture**. Chairs: Mariano Rocchi - Dieter Kotzot
	**Joris Vermeesch:** Somatic chromosomal mosaicim
**SUNDAY, 7 July 2019**	
08:30-10:15	**Plenary session 1 - Recent advances in cytogenomics**
	Chairs: Mariano Rocchi – Thierry Lavabre-Bertrand
08:30-09:00	**Claudia Haferlach:** The future of cytogenomics in the diagnostics
09:00-09:30	**Michael Speicher:** Liquid biopsies in patients with cancer
09:30-10:15	Selected abstracts
09:30-09:45	**Pascal Chambon:** A simple, universal and cost-efficient dPCR method for the targeted analysis of copy number variations
09:45-10:00	**Laïla El Khattabi:** Next Generation Mapping, a novel approach that enables the detection of unbalanced as well as balanced structural variants
10:00-10:15	**Paolo Reho:** Low-coverage whole genome sequencing in plasma circulating cell-free
	DNA analysis: the Turner syndrome experience
10:15-10:45	Coffee break
10:45-11:15	**Plenary session 2 - 50 Years of chromosome banding** Chairs: Kamlesh Madan - José Garcia-Sagredo
10:45-11:15	**Felix Mitelman:** Chromosome banding: the end of the Dark Ages
11:15-11:45	**Darío G. Lupiáñez:** Structural variation in the 3D genomic era: implications for disease and evolution
12:00-14:30	Poster session and Satellite Symposia
14:30 - 15:45	Concurrent Sessions
**Concurrent Session 1 - 3D chromatin organization and dynamics** Chairs: Jean-Michel Dupont - Darío G. Lupiáñez	
14:40-15:05	**Alexandre Reymond:** Genome architecture and diseases: the 16p11.2 paradigm
15:05-15:30	**Mario Nicodemi:** Polymer physics predicts the impact on chromatin 3D structure of disease associated structural variants
15:30-15:45	Selected Abstract
	**Mireia Solé:** Chromosome radial positioning in spermatogenic germ cells from *Mus musculus*
**Concurrent Session 2 - Clinical cytogenomics**	
Chairs: Damien Sanlaville - Orsetta Zuffardi	
14:40-15:05	**Nicole de Leeuw:** CNV and diseases: an overview in constitutional diagnostics
15:05-15:30	**Malte Spielmann:** The effect of structural variation in the three-dimensional genome
15:30-15:45	Selected Abstract
	**Jesper Eisfeldt:** From cytogenetics to cytogenomics, whole genome sequencing as a comprehensive genetic test in rare disease diagnostics
15:45-16:15	Coffee break
16:15-17:20	Concurrent Sessions
**Concurrent Session 3 - Structural organization of the human genome**	
Chairs: Joris Vermeesch - Nicole de Leeuw	
16:15-16:40	**Megan Y. Dennis:** The role of duplicated genes in human brain evolution and disease
16:40-17:05	**Francesca Antonacci:** Inversion variants in the human genome
17:05-17:20	Selected Abstract
	**Anna Lindstrand**: Cytogenetically visible inversions are formed by multiple molecular mechanisms
**Concurrent Session 4 - Human infertility**	
Chairs: Elisabeth Syk Lundberg **-** Sevilhan Artan	
16:15-16:40	**Pierre Ray:** Male infertility in humans, interest of whole exome sequencing
16:40-17:05	**Terry Hassold:** Aneuploidy in humans: new insights into an age-old problem
17:05-17:20	Selected Abstract
	**Harita Ghevaria**: Next generation sequencing detects premeiotic errors in human oocytes and provides evidence of genetic influence
17:20-18:30	Poster session
**MONDAY, 8 July 2019**	
08:30-10:30	**Plenary session 3 - Tumor Cytogenomics I**
	Chairs: Felix Mitelman – Roberta Vanni
08:30-09:00	**Fredrik Mertens:** Clonal evolution among different sarcoma subtypes
09:00-09:30	**David Gisselsson:** Treatment resilience of cancer through clonal evolution
09:30-10:30	Selected Abstracts
09:30-09:45	**Roberto Valli**: Shwachman-Diamond Syndrome, expression arrays, clonal chromosome anomalies
09:45 10:00	**Paola Caria:** Three-Dimensional Telomere Organization in papillary thyroid cancers
10:00-10:15	**Jordi Camps:** Patterns of acquired uniparental disomy reveal biallelic inactivation of tumor suppressor genes in gastrointestinal cancers and in colorectal advanced adenomas
10:15-10:30	**Sabrina Haslinger:** ZNF384 gene fusions in B-ALL: A report of fifteen Austrian cases secured by systematic FISH and array screening
10:30-11:00	Coffee break
11:00-12:15	Concurrent Sessions
**Concurrent Session 5 - Tumor cytogenomics II**	
Chairs: Claudia Haferlach - Harald Rieder	
11:00-11:30	**Liran Shlush:** Clonal evolution and risk factors - from age-related clonal hematopoiesis to AML
11:30-12:00	**Floris Foijer:** Single cell DNA sequencing to quantify karyotype heterogeneity in cancer
12:00-12:15	Selected Abstract
	**Karla Svobodova**: Identification of cryptic aberrations allows more accurate prognostic classification of patients with myelodysplastic syndromes and clonal evolution
**Concurrent Session 6 - Animal and plant cytogenomics I**	
Chairs: Pat Heslop-Harrison - Valerie Fillon	
11:00-11:30	**Alain Pinton:** Chromosome rearrangements and meiosis in pig
11:30-12:00	**Ilya Kirov:** Plant repeatome: cytogenetic, transcriptomic and proteomic aspects
12:00-12:15	Selected Abstract
	**Alessandra Iannuzzi:** Cytogenetic and genomic assays in river buffalo (*Bubalus bubalis*, 2n=50) cows raised in urban and rural areas
12:15-14:30	Poster session and Satellite Symposia
14:30-15:45	**Plenary session 4 - Chromosomal Imbalances**
	Chairs: Mariano Rocchi - Konstantin Miller
14:30-14:55	**Orsetta Zuffardi:** The trisomy legacy: from numerical to structural abnormalities
15:00-15:30	**Iben Bache:** Long-term outcomes of prenatally detected de novo balanced chromosomal rearrangements
15:30-15:45	Selected Abstract
	**Aafke Engwerda:** Phenotype-genotype analysis in a large cohort of 85 individuals with a terminal 6q deletion
15:45-16:15	Coffee break
16:15-17:30	Concurrent Sessions
**Concurrent Session 7 - Animal and plant cytogenomics II**	
Chairs: Trude Schwarzacher - Leopoldo Iannuzzi	
16:15-16:45	**Raquel Chaves:** Satellite evolution in Bovidae
16:45-17:15	**Vincent Colot:** Transposable element mobilization: where, how and with what consequences?
17:15-17:30	Selected Abstract
	**Fengtang Yang:** Characterisation of complex genomic structure and variation by high-resolution fibre-FISH: an overview
**Concurrent Session 8 - Accreditation, quality control and education**	
Chairs: Konstantin Miller - Albert Schinzel	
16:15-16:45	**Thomas Liehr:** European Certification and continuous education of clinical laboratory geneticists working in cytogenetics
16:45-17:15	**Thomas Eggermann:** Next generation sequencing and quality assurance: challenges and opportunities
17:15-17:30	Selected Abstract
	**Ron Hochstenbach:** What should laboratory specialists in clinical genetics know about chromosomes ten years from now?
17:30-18:30	Poster session
18:30	**E.C.A. General Assembly**
20:00	**Conference Dinner of E.C.A.**
**Tuesday, 9 July 2019**	
09:00-10:30	**Plenary session 5 - Prenatal diagnosis**
	Chairs: Damien Sanlaville - Maria Rosario Pinto Leite
09:00-09:25	**Rossa Chiu:** Cell-free DNA analysis as a tool for “non-invasive cytogenetics”
09:25-09:50	**Nathalie Brison:** The landscape of pathogenic copy number variations in healthy, reproducing females
09:50-10:20	Selected Abstracts
09:50-10:05	**Ming Chen:** A silicon-based coral-like nanostructured microfluidics to isolate rare cells in human circulation: validation by SK-BR-3 cancer cell line and its utility in circulating fetal nucleated red blood cells
10:05-10:20	**Celine Dupont:** Six years of molecular cytogenetic in prenatal diagnosis: benefits, lessons and perspectives. A new approach according to observation of ultrasound abnormalities
10:20-10:40	Coffee break
10:40-11:25	**Plenary session 6**
	Chair: Jean-Michel Dupont
10:40-11:10	**Frank Pellestor:** Chromoanagenesis: cataclysms behind complex chromosomal rearrangements
11:10-11:25	Selected Abstract
	**Adriana Di-Battista:** Balanced X-autosome translocations and premature ovarian failure are associated with altered expression of growth factors, junction organization and immune pathways
11:25-12:15	**Keynote lecture**
	Chair: Mariano Rocchi
	**Stylianos Antonarakis:** Chromatin and single cell genomics, to understand the gene dosage imbalance in aneuploidies
12:15	**Closing Ceremony**: Mariano Rocchi

## Invited Lecture Abstracts

### L1 Somatic chromosomal mosaicism

#### Joris Robert Vermeesch (Joris.vermeesch@kuleuven.be)

##### Center for Human Genetics, KULeuven, Leuven, Belgium

It is generally assumed that all cells in the body are diploid. However, single cell genomic analysis is demonstrating that chromosomal mosaicism is common and that the mitotic error rate is significantly higher than previously assumed. We have been mapping this chromosomal instability starting from the beginning of life, from the zygote to the elderly. To map chromosomal mosaicism, we have been developing methods to accurately map chromosomal changes as well as methods to map haplotypes in single cells. The methods reconstruct genome-wide haplotype architectures as well as the copy-number and segregational origin of those haplotypes by employing phased parental genotypes and deciphering WGA-distorted SNP B-allele fractions. In another approach, we can map chromosomal anomalies by mapping the DNA fragments present in liquid biopsies and subsequently deconvoluting the signals. By looking at both haplotypes and copy numbers we are acquiring novel insights into chromosomal missegregation during the early cleavages, in placenta and in other somatic tissues. I will provide an overview of distinct and unique segregational aberrations.

### L2 The future of cytogenomics in the diagnostics

#### Claudia Haferlach (claudia.haferlach@mll.com)

##### MLL Munich leukemia laboratory, Max-Lebsche-Platz 31, 81377 München, Germany

The spectrum of genomic abnormalities present in human diseases deriving from germline and/or acquried genetic alterations is wide and encompasses gross and submicroscopic aberrations including copy number alterations, structural variants and small nucleotide variants. Thus, to date for a comprehensive genetic work-up a set of cytogenetic and molecular genetic techniques are performed. As sequencing technologies have evolved rapidly whole exome sequencing (WES), whole genome sequencing (WGS), and whole transcriptome sequencing (WTS) have already reached or are ready to be tested in a routine diagnostic setting. WGS provides the possibility to capture all genomic information in a single assay. However, reliable studies in a diagnostic setting are required to determine whether WGS can replace current techniques.

At MLL WGS was performed up to now in more than 4000 samples from patients with various hematological malignancies in order to evaluate the feasibility of WGS in a routine diagnotic setting and the impact WGS might have on the diagnostic work-up of hematological neoplasms in future. First analyses revealed a high detection rate by WGS of genomic abnormalities identified by standard diagnostic procedures. 555 of 574 (96.7%) balanced rearrangements detected by chromosome banding analysis were also identified by WGS. Further WGS detected 60 recurrent balanced rearrangements that were missed by CBA due to cytogenetically cryptic fusions or insufficient in vitro proliferation of the aberratin in vitro. Comparing 18,337,602 positions 18,031,728 (98%) yielded the same result with genomic array analysis and WGS with respect to gain, loss or normal copy number status, respectively.

The most challenging part of using WGS as a diagnostic tool is the elimination of sequencing and alignment artefacts as well as the clinical interpretation of rare variants. Thus, we built a pipeline integrating several data bases in order to facilitate data interpretation. The next steps on the road towards a diagnostic tool are the validation of copy number alterations, structural variants, and small nucleotide variants identified in addition to standard diagnostics and the determination of the coverage necessary to detect small clones relevant for patient care.

The technological advances will change future diagnostics dramatically challenging geneticists to transform the huge amount of genetic data into improved classification of diseases and individualization of treatment in order to improve patients outcome.

### L3 Liquid biopsies in patients with cancer

#### Michael R. Speicher (Michael.speicher@medunigraz.at)

##### Medical University of Graz, Institute of Human Genetics, Graz, Austria

Precision oncology seeks to leverage molecular information about cancer to improve patient outcomes and to this end tissue biopsies are widely used to characterize tumor genomes. Recently, attention has been turning to minimally-invasive liquid biopsies, which enable analysis of tumor components (including circulating tumor DNA [ctDNA]) in bodily fluids such as blood. Analyses of ctDNA has been used to track the evolutionary dynamics and heterogeneity of tumors. Furthermore, ctDNA analyses can detect very early emergence of therapy resistance, residual disease, and recurrence. Our group has developed several methods for the analysis of ctDNA. To date, we have analyzed more than 4,000 plasma samples from patients with cancer (breast, prostate, colon, renal and lung carcinoma), which allowed us to estimate the dynamics of clonal evolution of tumor genomes and to identify mechanisms of resistance against given therapies.

Recently we leveraged the fact that plasma DNA is nucleosome protected DNA. After whole-genome sequencing appropriate bioinformatics including support vector machines allowed the mapping of nucleosome positions based on the genomic sequencing coverage of plasma DNA fragments. For example, the genomic sequencing coverage of plasma DNA fragments around transcription start sites (TSSs) has a distinct pattern allowing the identification of actively transcribed genes of cells releasing their DNA into the circulation. The expression levels of genes in the corresponding tumor were reflected by the coverage around the TSSs in plasma of patients with cancer. Another approach, based on similar principles, allows assessment of TF activity based on cell-free DNA sequencing and nucleosome footprint analysis. To this end, we analyzed whole genome sequencing data for >1,000 cell-free DNA samples from cancer patients and healthy controls using a newly developed bioinformatics pipeline that infers accessibility of TF binding sites from cell-free DNA fragmentation patterns. We observed tumor-specific patterns, including accurate prediction of tumor subtypes in prostate cancer, with important clinical implications for the management of patients. Furthermore, we show that cell-free DNA TF profiling is capable of detection of early-stage colorectal carcinomas. The great potential of liquid biopsies makes ctDNA analyses to a promising tool for precision medicine.

### L4 Chromosome banding: the end of the Dark Ages

#### Felix Mitelman (felix.mitelman@med.lu.se)

##### Department of Clinical Genetics, Institute of Laboratory Medicine, University of Lund, Lund, Sweden

The introduction of the first chromosome banding technique (Q-banding) by Lore Zech and Torbjörn Caspersson 50 years ago completely revolutionized cytogenetic analysis. Whereas formerly identification of chromosomes was restricted to chromosome groups based on size and centromere location, now each chromosome, chromosome arm, and even individual chromosome regions could be precisely identified on the basis of their unique banding pattern. Very soon, several other banding techniques were developed, for example, G-, R-, C-, and NOR-banding, each having its own specific properties and applications.

The discovery of chromosome banding had an enormous impact and ushered in an unparalleled period of advancement in cytogenetics as well as in several areas of biology and medicine. The possibility to identify and exactly characterize chromosome aberrations in humans laid the foundation for the clinical application of cytogenetic analyses and established clinical cytogenetics as a medical specialty. Numerous constitutional chromosome abnormality syndromes were delineated and the detection of such aberrations in affected individuals or through prenatal diagnostic procedures soon became standard practice. At the same time, consistent or even specific cancer-associated chromosome changes, not imagined before this era, were disclosed in most tumor types. These findings had important implications. Characteristic acquired chromosome aberrations became an important diagnostic tool and also provided a means to unravel pathogenetic mechanisms by pinpointing the location of cancer-initiating genes.

The major contribution of banding techniques in basic research in general was the mapping of genes on chromosomes, refined substantially by the subsequent development of high-resolution banding. The utilization of highly elongated pro-metaphase or prophase chromosomes provided even greater precision than conventional banding by revealing more than twice the number of bands seen at metaphase. The assignment and localization of genes to chromosomes at the 850 sub-band level were central to the construction of genetic maps, and high-resolution banding played an important role in the verification of gene order in such maps. It is difficult to overstate the value of this contribution of chromosome banding to the effort to sequence the human genome.

### L5 Structural variation in the 3D genomic era: Implications for disease and evolution

#### Darío G. Lupiáñez (Dario.Lupianez@mdc-berlin.de)

##### Institute for Medical Systems, Biology, Max- Delbrück Center for Molecular, Medicine, Berlin- Buch, Germany

3D spatial organization is an inherent property of the vertebrate genome to accommodate the roughly 2m of DNA in the nucleus of a cell. On a larger scale, chromosomes display a nonrandom nuclear organization highly influenced by their gene density and transcriptional status. On a subchromosomal scale, the 3D organization of chromatin brings pairs of genomic sites that lie far apart along the linear genome into proximity. Within such organization, topologically associating domains (TADs) emerge as a fundamental structural unit that guides regulatory elements to their cognate promoters to induce transcription (Lupiáñez et al., 2016).

Structural and quantitative chromosomal rearrangements, collectively referred to as structural variation (SV), contribute to a large extent to the genetic diversity of the human genome and thus are of high relevance for cancer genetics, rare diseases and evolutionary genetics. Recent studies have shown that SVs can not only affect gene dosage but also modulate basic mechanisms of gene regulation (Lupiáñez et al., 2015; Franke et al., 2016; Will et al., 2017; Bianco et al., 2018; Kragesteen et al., 2018). SVs can alter the copy number of regulatory elements or modify the 3D genome by disrupting higher-order chromatin organization such as TADs. As a result of these position effects, SVs can influence the expression of genes distant from the SV breakpoints, thereby causing the appearance of certain pathogenic phenotypes or evolutionary traits. Therefore, the impact of SVs on the 3D genome and on gene expression regulation has to be considered when interpreting the phenotypical consequences of these variant types (Spielmann et al., 2018).

In this talk, I will show examples at different genomic loci, highlighting the potential of SVs to induce developmental disease by distinct pathomechanisms. Furthermore, I will discuss about the iberian mole Talpa occidentalis, a unique case of true XX mammalian hermaphroditism, and a prominent example of how SVs can also be a force of evolutionary innovation.

### L6 Genome architecture and diseases: the 16p11.2 paradigm

#### Alexandre Reymond (alexandre.reymond@unil.ch)

##### Center for Integrative Genomics, University of Lausanne, Genopode building, CH-1015 Lausanne, Switzerland

**Keywords**: CNV, 16p11.2, chromatin, evolution, GWAS

Copy number changes in 16p11.2 contribute significantly to neuropsychiatric traits. Besides the 600 kb BP4-BP5 (breakpoint) CNV found in 1% of individuals with autism spectrum disorders and schizophrenia and whose rearrangement causes reciprocal defects in head size and body weight, a second distal 220kb BP2-BP3 CNV is a likewise potent driver of neuropsychiatric, anatomical and metabolic pathologies. These two CNVs-prone regions at 16p11.2 are reciprocally engaged in complex chromatin looping and concomitant expression changes, as well as genetic interaction between genes mapping within both intervals, intimating a functional relationship between genes in these regions that might be relevant to pathomechanism.

These recurrent pathogenic deletions and duplications are mediated by a complex set of highly identical and directly oriented segmental duplications. This disease-predisposing architecture results from recent, Homo sapiens-specific duplications (i.e. absent in Neandertal and Denisova) of a segment including the BOLA2 gene, the latest among a series of genomic changes that dramatically restructured the region during hominid evolution. Our results show that BOLA2 participates in iron homeostasis and a lower dosage is associated with anemia. These data highlight a potential adaptive role of the human-specific expansion of BOLA2 in improving iron metabolism.

Finally, we combined phenotyping of carriers of rare copy variant at 16p11.2, Mendelian randomization and animal modeling to identify the causative gene in a Genome-wide association studies (GWAS) locus for age at menarche. Our interdisciplinary approach allowed overcoming the GWAS recurrent inability to link a susceptibility locus with causal gene(s).

### L7 Polymer physics predicts the impact on chromatin 3D structure of disease associated structural variants

#### Mario Nicodemi (mario.nicodemi@unina.it)

##### Dipartimento di Fisica "Ettore Pancini" Università di Napoli Federico II Via Cinthia, 21 - Edificio 6 80126 - Naples - Italy

Structural variants (SVs) are a frequent cause of disease and significantly contribute to the variability of our genome, yet their medical impact is usually hard to predict. Recent technologies, such as Hi-C, have revealed that SVs can alter the 3D architecture of chromosomes inducing ectopic contacts between genes and their regulators, leading to mis-expression in congenital diseases. Analogous effects have been also reported in cancer tissues. I discuss how chromosome 3D conformation and folding mechanisms can be understood in a principled way by use of polymer physic [1]. In particular, in the case study of the EPHA4 [2] and PITX1 [3] loci, I illustrate that the effects of pathogenic structural variants can be predicted in-silico, as validated by Hi-C data generated from mouse limb buds and patient-derived fibroblasts.

[1] M M. Barbieri, S.Q. Xie, E. Torlai Triglia, A.M. Chiariello, S. Bianco, I. de Santiago, M.R. Branco, D. Rueda, M. Nicodemi*, A. Pombo*, Active and poised promoter states drive folding of the extended HoxB locus in mouse embryonic stem cells. Nature Struct. Mol. Bio, 24, 515 (2017).

[2] S. Bianco, D.G. Lupiáñez, A.M. Chiariello, C. Annunziatella, K. Kraft, R. Schöpflin, L. Wittler, G. Andrey, M. Vingron, A. Pombo, S. Mundlos*, M. Nicodemi*, Polymer physics predicts the effects of structural variants on chromatin architecture, Nature Genetics 50, 662 (2018).

[3] B.K. Kragesteen, M. Spielmann, C. Paliou, V. Heinrich, R. Schoepflin, A. Esposito, C. Annunziatella, S. Bianco, A.M. Chiariello, I. Jerković, I. Harabula, P. Guckelberger, M. Pechstein, L. Wittler, W.-L. Chan, M. Franke, D.G. Lupiáñez, K. Kraft, B. Timmermann, M. Vingron, A. Visel, M. Nicodemi*, S. Mundlos* and G. Andrey*, Dynamic 3D Chromatin Architecture Determines Enhancer Specificity and Morphogenetic Identity in Limb Development. Nature Genetics 50, 1463 (2018).

### L8 CNV and Diseases: An overview in constitutional diagnostics

#### Nicole de Leeuw (Nicole.deLeeuw@radboudumc.nl)

##### Department of Human Genetics, Radboud university medical center, Nijmegen, the Netherlands

**Keywords**: CNV, array, exome, disease

Copy Number Variants (CNVs), copy number gains or losses ranging in size from less than 1 kb up to many megabases, are frequently identified as the genetic cause in a growing number of disorders. A CNV can involve a single gene leading to specific, phenotypic consequences limited to a single organ or affecting multiple organs, but a CNV may also affect numerous genes resulting in a syndromic phenotype. Some CNV-related clinical features can already be observed prenatally and are present at birth, whereas others develop or become apparent in the first years of life or at a later age.

Although CNVs were first considered to be predominantly involved in neurodevelopmental disorders and congenital anomalies, it is now known that they also play a role in many other disorders, ranging from hearing impairment to late-onset diseases. An illustrative overview will be given on the role of CNVs in human disease, predominantly based on the CNV findings in our diagnostic laboratory from a total of more than 22,000 SNP-based arrays and over 31,000 exomes.

The ability to detect both nucleotide variants and CNVs in a single exome sequencing test significantly increases the chance to identify the genetic cause for a patient's clinical phenotype, which can help to better define targeted interventions and improve clinical management.

### L9 The effect of structural variation in the three-dimensional genome

#### Malte Spielmann (spielmann@molgen.mpg.de)

##### Max Planck Inst. for Molecular Genetics, Ihnestraße 63-73, 14195 Berlin, Germany

Structural and quantitative chromosomal rearrangements (SVs) contribute to a large extent to the genetic diversity of the human genome and thus are of high relevance for cancer genetics, rare diseases and evolutionary genetics. Recent studies have shown that SVs can not only affect gene dosage but also modulate basic mechanisms of gene regulation. SVs can alter the copy number of regulatory elements or modify the 3D genome by disrupting higher-order chromatin organization such as topologically associating domains. As a result of these position effects, SVs can influence the expression of genes distant from the SV breakpoints, thereby causing disease. The impact of SVs on the 3D genome and on gene expression regulation has to be considered when interpreting the pathogenic potential of these variant types. In my talk I will discuss how SVs can modify the 3D organization of the genome by disrupting chromatin domains. I will also describe the phenotypic consequences of genomic disorders resulting from reshuffling of non- coding enhancer sequences and chromatin domain boundaries with the aim of presenting possible strategies for the medical interpretation of SVs in the 3D genome.

### L10 The role of duplicated genes in human brain evolution and disease

#### Aarthi Sekar^1,2^*, Daniela Soto^1,2^*, José Uribe-Salazar^1,2^, Gulhan Kaya1, Ruta Sahasrabudhe^3^, Megan Y. Dennis^1,2^

##### ^1^Genome Center, MIND Institute, and Department of Biochemistry & Molecular Medicine; ^2^Integrative Genetics and Genomics Graduate Group; ^3^DNA Technologies Sequencing Core Facility, University of California, Davis, CA 95616

###### **Correspondence:** Megan Y. Dennis (mydennis@ucdavis.edu)

* These authors contributed equally to this work.

The human cortex exhibits dramatic anatomical and cognitive differences from those of closely related primate species. Despite a few potential success stories, the underlying genetic contributors to unique human adaptive traits remain undiscovered. We posit that human-specific segmental duplications (HSDs; genomic regions >1 kbp in size with >98% identity) may be a source of neurological innovation and disease that have remained largely understudied. Two HSD genes, SRGAP2C and ARHGAP11B, have been previously implicated in cortex expansion. Using a human haploid-derived (CHM1) BAC resource, we previously performed Pacific Biosciences long-read sequencing to correct the largest, gene-containing HSDs, fixing over 18.2 Mbp in the current human reference build and identifying over 30 additional HSD gene families. Of these, we honed in on a set of ten duplicate gene families with the propensity to be functional today based on their presence in all of humans tested (thousands) and exhibiting gene expression in adult post-mortem tissues from GTEX. To refine our genes to those important in neurodevelopment, we are employing a multifaceted functional approach using cell lines, zebrafish, and mice. Understanding that genetic variation segregating in modern human populations can also inform on if a gene is functional (e.g., an excess of truncating mutations may indicate loss of function), we are leveraging sequence data of HSD gene paralogs. Unfortunately, only 1.7% of HSDs are accessible for variant calling using whole-genome shotgun short-read (Illumina) data from 1000 Genomes Project. Furthermore, 78% of HSD regions are completely depleted for common variants (dbSNP). As such, we are performing targeted long-read sequencing in diverse human populations to accurately detect variants in these typically inaccessible regions. Though a work in progress, if successful, the results of these studies will offer important insights into if/how HSD genes contribute to innovative neurological features that distinguish modern humans from related great ape species.

### L11 Inversion variants in the human genome

#### Francesca Antonacci (francesca.antonacci@uniba.it)

##### Dept. Biology, University of Bari, Bari, Italy

Structural variation is increasingly acknowledged as an important source of human genetic variation accounting for disease and population diversity. Significant advances have been made over the past few years in mapping and characterizing structural variation in the human genome. Inversion polymorphisms, however, represent a relatively unexplored form of structural variation. Although they are not usually associated with alterations in gene copy number and, thus, do not have a primary effect on phenotype, several of the polymorphic inversions identified to date confer a predisposition to further chromosomal rearrangements in subsequent generations. The majority of inversions described in the human genome are flanked by highly identical segmental duplications causing assembly errors in genome references as well as problems for inversion discovery using next-generation sequencing approaches. Combining molecular cytogenetics, genomic approaches, and sequencing of long molecules we recently characterized some of the largest inversion polymorphisms in the human population. We investigated their worldwide population characteristics, established their association to human disease, and unveiled their evolutionary history. Our data shows that inversion polymorphisms are common and some show striking population stratification. Inversions associate with regions predisposed to disease- causing microdeletions and reoccur at a high frequency due to the presence of duplicated sequences at their boundaries. These structural polymorphisms occur at varying frequencies in populations leading to different susceptibility and ethnic predilection.

### L12 Male infertility in humans, interest of whole exome sequencing

#### Pierre F. Ray (PRay@chu-grenoble.fr)

##### University Grenoble Alpes, INSERM U1209, CNRS UMR 5309, Institute for Advanced Biosciences, Team Genetics Epigenetics and Therapies of Infertility, 38000 Grenoble, France

**Keywords**: Infertility, spermatogenesis, genetic diagnosis, whole exome sequencing

Infertility is currently considered by the World Health Organization (WHO) as a major health concern affecting more than 50 million couples worldwide. An abnormality of the spermogram is found in half of the cases indicating a balanced responsibility between the man and the woman in the occurrence of couple infertility. Male infertility is characterized by a multifactorial etiology often associated with a strong genetic component, especially when it comes to severe phenotypes such as azoospermia and monomorphic teratozoospermia. The most commonly identified genetic causes of male infertility concern chromosomal defects affecting mainly the gonosomes like Klinefelter syndrome or Y microdeletions but also the autosomes as balanced translocations (robertsonian and reciprocal) are often associated with infertility. However, the realization of a karyotype and the screening for microdeletions permit to reach a diagnosis for no more than 20% of men with non-obstructive azoospermia and diagnosis efficiency is much reduced for the milder sperm defects. Hundreds of genes are specifically expressed in the testes and are necessary for spermatogenesis, the occurrence of genetic defects in any of these genes is therefore likely to result in male infertility. The profusion of these genes makes the identification of mutations responsible for male infertility difficult and complex. High throughput sequencing, and in particular whole exome sequencing (WES), is however revolutionizing this field and has recently permitted to identify numerous genes associated with different phenotypes of male infertility.

Our Grenoble team "epigenetic genetics and infertility therapies" has been working for 10 years to identify and characterize the causes of male infertility. Using homozygosity mapping techniques we could identify several major genes involved in different male infertility syndromes such as AURKC in macrozoospermia, DPY19L2 in globozoospermia, and DNAH1 in the phenotype of multiple flagellar morphological abnormalities (MMAF). Whole exome sequencing has recently allowed us to be much more efficient and to identify several other genes involved in the MMAF phenotype (CFAP43, CFAP44, CFAP69, AK7, FSIP2, WDR66, ARMC2) or azoospermia (SPINK2) or female infertility (PATL2). Genes localized on the X chromosome have also been identified with TEX11 and ADGRG2 resulting respectively in non-obstructive and obstructive azoospermia.

Some small genomic rearrangements (CNVs) have been identified and characterized and are the most frequent defects identified in DPY19L2 and WDR66. These abnormalities can be detected effectively by WES. The use of high throughput sequencing is transforming the diagnosis of male infertility and this technique is expected to become an integral part of the routine diagnosis proposed for the management of infertile.

### L13 Aneuploidy in humans: new insights into an age-old problem

#### Terry Hassold (terryhassold@wsu.edu)

##### School of Molecular Biosciences, Washington State University, 99164 Pullman WA USA

Keywords: aneuploidy, meiotic recombination

Aneuploidy is the most common genetic complication of pregnancy, with approximately 0.2-0.3% of newborn infants being trisomic. However, this represents just the tip of a large iceberg, because most aneuploid conceptions die in utero. Indeed, studies of preimplantation embryos suggest that a large proportion, if not a majority, of fertilized human eggs have extra or missing chromosomes. Because the vast majority of errors result from the fertilization of a chromosomally abnormal egg by a normal sperm, attention has focused on why human female meiosis is so error-prone.

In this presentation, we will briefly summarize our work indicating that there are multiple routes to female-derived aneuploidy; e.g., studies of model organisms indicating the contribution of errors occurring during the long meiotic arrest stage or as part of the meiotic cell cycle checkpoint machinery. We will also discuss our recent studies, which have focused on analyzing human meiosis “as it happens” in fetal oocytes and in spermatocytes. These studies demonstrate remarkable differences between human males and females in the way in which chromosomes find and synapse with one another, in the packaging of chromatin, and in the control of the meiotic recombination pathway. Further, they indicate that errors in fetal oogenesis - especially those that lead to failure to recombine or abnormally located crossovers - are surprisingly common in humans. Indeed, our observations suggest that the propensity to nondisjoin may be established – at least in part – at the very beginning of the development of the human oocyte.

### L14 Clonal evolution among different sarcoma subtypes

#### Fredrik Mertens (fredrik.mertens@med.lu.se)

##### Division of Clinical Genetics, Department of Laboratory Medicine, Lund University, SE-221 84 Lund, Sweden and Department of Clinical Genetics and Pathology, University and Regional Laboratories Region Skåne, SE-221 85 Lund, Sweden

Sarcomas are malignant tumors arising in soft tissues or bone. Although genetic analyses have shown that most morphologic subtypes have unique chromosomal features, the pathogenetic mechanisms behind sarcoma development may be broadly categorized into three distinct subgroups; the largest is characterized by complex combinations of chromosomal gains and losses, followed by sarcomas driven by gene fusions or supernumerary ring chromosomes. To compare patterns of clonal evolution in sarcomas arising through these three different mechanisms, we selected sarcomas with complex genomes (myxofibrosarcomas, MFS), gene fusion-driven myxoid liposarcomas (MLS), and amplicon-driven well-differentiated liposarcomas (WDLS) from which we had access to multiple samples during tumor progression; a further requisite was that at least one year should have elapsed between first and last sampling. We also studied multiple samples from some of the primary lesions, in order to evaluate intra-lesional heterogeneity. Clonal heterogeneity was assessed through a combination of chromosome banding, single nucleotide polymorphism (SNP) array, and whole-exome sequencing analyses.

We could show that the type of clonal evolution – i.e., whether nucleotide or chromosome level mutations predominate – and the rate by which new mutations accrue vary considerably among the three sarcoma types. In MFS, tumor progression was usually accompanied by accumulation of both chromosome and nucleotide level aberrations. Primary MLS display little intratumoral heterogeneity and few new mutations are found in local recurrences or metastases. WDLS, on the other hand showed extensive inter-cellular variation in terms of chromosome level aberrations; this variation, however, had only minor impact on the predominant clone in each tumor. Furthermore, no significant single nucleotide variants were seen in primary tumors or at relapse.

Thus, there is a considerable variation among sarcomas caused by different pathogenetic mechanisms with regard type of clonal evolution and to the rate by which new mutations become predominant. It is also of interest that, as exemplified by WDLS, marked variation at the single cell level does not necessarily translate into major changes in the tumor stemline.

### L15 Treatment resilience of cancer through clonal evolution

#### Jenny Karlsson^1^, Hiroaki Yasui^1,2^, Natalie Andersson^1^, Anders Valind^1,3^, Bjorn Bakker^4^, Linda Holmquist Mengelbier^1^, Floris Foijer^4^, David Gisselsson^1,5,6^

##### ^1^Division of Clinical Genetics, Department of Laboratory Medicine, Lund University, Lund, Sweden; ^2^Department of Obstetrics and Gynecology, Graduate School of Medicine, Nagoya University, Japan; ^3^Department of Pediatrics, Skåne University Hospital, Lund, Sweden; ^4^European Research Institute for the Biology of Ageing (ERIBA), University of Groningen, University Medical Center Groningen, Groningen, The Netherlands; ^5^Department of Pathology, Medical Services, Laboratory Medicine, Skåne Region, Lund, Sweden; ^6^Division of Oncology-Pathology, Department of Clinical Sciences, Lund University, Lund, Sweden

###### **Correspondence:** David Gisselsson (david.gisselsson_nord@med.lu.se)

*Background*: Most patients who die from cancer do so because of treatment resistant metastatic disease. Darwinian selection is essential for how cancers adapt to treatment. We explore this evolutionary process in three prototypical childhood cancers, neuroblastoma, Wilms tumor and rhabdomyosarcoma.

*Methods*: Genetic variation has been inventoried in >60 patients through multiregional sampling of primary tumors and their metastatic sites. At present >300 tumor regions have been subjected to high-resolution copy number analysis, complemented by whole exome and single cell whole genome sequencing. The detected genetic variation is used to reconstruct the evolutionary history of each tumor.

*Results*: We find that approximately 90% of primary tumors show a history of branching evolution, sometimes leading to regional variation in genetic markers that are used for treatment decisions e.g. 1q gain in Wilms tumors and the presence of structural vs. numerical aberrations in neuroblastoma. This variation can be traced back to four distinct evolutionary trajectories of which the ones indicating intense tumor cell competition are associated with a higher risk of relapse. Timelines inferred from the spatial distribution of genetic changes indicate that the genetic alterations associated with prognosis emerge at different points in evolutionary history for different tumor types, for example at the initiation of clonal expansion for neuroblastoma but closer to presentation for Wilms tumor. Tumors with a high relapse risk exhibit extensive branching into novel clones, leading to a rich subclonal underground that can act as a reservoir of genetic variation. In line with this, we find that chemotherapy typically leads to a replacement of the original clones by a set of collaterally related survivors. Similarly, the clones of metastatic relapse are usually distant collateral relatives of the primary tumor’s main clones. However, we find that relapse manifesting at multiple sites typically have a common single cell ancestor, indicating that a specific anatomic locus acted as a bridgehead for further metastatic spread.

*Conclusions*: From a clinical standpoint, our data indicate that (1) certain genetic markers used for treatment decisions today show a variation that in fact prevents unequivocal classification of tumors as positive or negative, (2) mutation analysis for the purpose of targeted therapy of relapsed tumors warrant resampling and reanalysis because data from the primary tumor can be misleading, and (3) strategies to prevent death from incurable relapse should focus more on early anatomic localization and elimination of the first relapsing clone to prevent dissemination to additional sites.

### L16 Single cell DNA sequencing to quantify karyotype heterogeneity in cancer

#### Bjorn Bakker, Hilda van den Bos, Diana C.J. Spierings, Floris Foijer

##### European Research Institute for the Biology of Ageing (ERIBA), University of Groningen, University Medical Center Groningen, Antonius Deusinglaan 1, NL-9713 AV Groningen, the Netherlands

###### **Correspondence:** Floris Foijer (f.foijer@umcg.nl)

Two out of three tumors display abnormal chromosome content, a state defined as aneuploid. Aneuploidy is the result of chromosome missegregation events, a process known as chromosomal instability (CIN). Aneuploidy and in particular CIN are associated with adverse clinical outcome, such as metastasis and therapy resistance. Paradoxically, CIN and the resulting aneuploidy inhibit cell proliferation of non-cancer cells, suggesting that cancer cells have acquired mutations that help them overcome the disadvantages of aneuploidy. In our lab, try to decipher such mechanisms, as targeting these mechanisms could provide a powerful means to selectively kill cancer cells. To this aim, we take three complementary approaches: 1) we develop tools to faithfully quantify and visualize CIN and the resultant aneuploidy, 2) we map and investigate biological pathways that help aneuploid cells convert into cancer cells, and 3) we use these tools and new mechanistic insight to design and test novel therapeutic intervention strategies that selectively kill aneuploid cancer cells.

In my presentation, I will focus mostly on our first aim and discuss findings of our recently developed toolkit to quantify aneuploidy and CIN at high resolution in primary cancer cells through single cells whole genome sequencing (scWGS). I will present some of our latest results from our mouse models in which we provoked CIN in a cancer and a non-cancer setting, which we use to study karyotype evolution in developing tumors. Furthermore, I will show some examples of scWGS for human cancer samples, exemplifying how scWGS can provide insight into the karyotype heterogeneity in a human background and how these data compare to our findings in mice.

### L17 Chromosome rearrangements and meiosis in pig

#### A. Pinton^1^, A, N. Mary^1^, H. Barasc^1^, N. Bonnet^1^, Calgaro^1^, P Berlib^1^, S. Ferchaud^2^, I. Raymond Letron^3^, T. Faraut^1^, H. Acloque^1^, and A. Ducos^1^

##### ^1^GenPhySE, Université de Toulouse, INRA, INPT, ENVT, Castanet Tolosan, France; ^2^GenESI Génétique, Expérimentation et Système Innovants Poitou Charentes F-17700 Saint-Pierre-d'Amilly, France; ^3^LabHPEC (Laboratoire d'HistoPathologie Expérimentale et Comparée), ENVT, Université de Toulouse, France and STROMALab, Université de Toulouse, CNRS ERL5311, EFS, ENVT, Inserm U1031, UPS, Toulouse, France

###### **Correspondence:** A. Pinton (a.pinton@envt.fr)

Constitutional chromosomal rearrangements are relatively frequent genetic abnormalities in humans and in animal species. They can lead to developmental disorders (malformations, mental retardations), and/or altered reproduction.

In domestic animal species, these rearrangements are potentially responsible for decreased reproductive abilities of the carriers and/or their mates and subsequent economic losses to the animal production industry.

Chromosomal rearrangements can be responsible for meiotic division impairments, leading in males to total or partial spermatogenesis arrest (oligo-, azoospermia). Generally, however, heterozygous carriers (male and female) of these abnormalities produce genetically unbalanced gametes, leading to early embryonic mortality. In pigs, this can result in a considerable reduction of the mates’ litter size.

The chromosomal control of pig populations has therefore been instigated in various countries. In France, for example, more than 42500 individuals have now been karyotyped, allowing more than 200 new structural chromosomal rearrangements to be detected and their prevalence estimated (about 0.5%). Among these, 87% were reciprocal translocations, 10% were inversions, 2% Robertsonian translocations and 1% uncharacterized rearrangements. Conversely to humans, altered semen quality (oligo- or azoospermia) has been reported in only a limited number of cases: in four X- or Y-autosome translocations (X/14 and Y/1, Y/9, Y/13, Y/14, Y/16) and 3 autosome/autosome translocations (1/14, 2/14, 1/15), suggesting that the meiotic process is different in pigs.

Besides structural rearrangements, sex chromosomal abnormalities have also been identified (2n=38,XY/2n=38,XX mosaicism and 2n=39,XYY or 2n=39,XXY).

The impact of some of these rearrangements on reproduction has been estimated by analyzing the meiotic process (meiotic pairing and recombination during pachytene) and the male and female meiotic segregation products.

Analyses of the meiotic prophase revealed an impaired meiotic process (accumulation of the γH2AX histone on autosomal segments and absence from the sex chromosomal regions), altered recombination patterns and gene expression disturbances (repression of autosomal genes and expression of normally repressed sex chromosomal genes) in individuals with altered semen quality.

As in humans, the frequency of unbalanced gametes production by male carriers of structural chromosomal rearrangements varied from one abnormality to another (reciprocal translocations from 24% to 48%, Robertsonian translocation about 3% and inversions <5%).

Comparisons of male and female carriers of the same abnormalities revealed that the meiotic segregation profiles differed between genders. Finally, results obtained for inversions showed that, conversely to humans, the frequency of unbalanced gametes, whatever the size of the inverted region, is relatively low, suggesting that pig chromosomes exhibit particular behavior during meiosis.

### L18 Plant repeatome: cytogenetic, transcriptomic and proteomic aspects

#### Ilya Kirov^1^, Marina Gilyok^2^, Igor Fesenko^3^, Andrey Knyazev^3^, Alexander Soloviev^1^, Ludmila Khrustaleva^2^

##### ^1^All Russian Research Institute of Agricultural Biotechnology, Moscow, Russian Federation; ^2^Russian State Agrarian University – MTAA, Moscow, Russian Federation; ^3^Shemyakin-Ovchinnikov Institute of Bioorganic Chemistry, Moscow, Russian Federation

###### **Correspondence:** Ilya Kirov (kirovez@gmail.com)

Repeatome is the complement of all types of repeated sequences in a genome including mobile elements, satellite DNA and others. Since the discovery at the 1960s that the bulk of eukaryotic genomes comprises repetitive DNA sequences they have been under intensive investigation. The repeatome has for a long time been considered as ‘junk’ and ‘parasitic’ DNA. The recent studies have shown that with its enormous variability and internal epigenetic dynamics, repeatome may play an important role in cell function. However, much of repeatome remains under-studied, poorly annotated and functionally mysterious. We performed an extensive study of repeatome in different plant species (onion, rose, moss and sunflower) and its mapping on plant chromosomes to understand the trends of genomic organization and evolution. Various repeat types have been isolated including heterochromatin- and nucleolar-associated tandem repeats and centromeric repeats. Applying bioinformatic analysis of total RNAseq as well as long-read sequencing data we showed that many repeat types are transcribed into lncRNAs. Moreover, using mass-spectrometry data the translation of uncharacterized open reading frames has been demonstrated for some retrotransposons and the corresponding peptides have been detected. Our ongoing work showed that the repeatome may contribute to the wide range of cellular mechanisms. Possible repeatome functions will be discussed in light of new data and novel tools.

This work was partially supported by RSF grants №17-46-07005 and №17-14-01189 and President’s grant № МК-2360.2019.4.

### L19 The trisomy legacy: from numerical to structural abnormalities

#### Orsetta Zuffardi (orsetta.zuffardi@unipv.it)

##### University of Pavia, Italy

Through a whole genome approach, including whole genome sequencing and genotyping of trios in de novo unbalanced rearrangements such as small supernumerary marker chromosomes, translocations, and insertions, we and others highlighted that many of them are the final product of a double-pass event triggered by chromosome non-disjunction at maternal meiosis, resulting in the formation of a trisomic zygote. The trisomic rescue through anaphase lagging of the supernumerary chromosome involved its repositioning within a micronucleus thus igniting a chromothripsis event with the rescue of only part of it.

### L20 Long-term outcomes of prenatally detected de novo balanced chromosomal rearrangements

#### Christina Halgren, Nete M. Nielsen, Lusine Nazaryan-Petersen, Asli Silahtaroglu, Ryan L. Collins, Chelsea Lowther, Susanne Kjaergaard, Morten Frisch, Maria Kirchhoff, Karen Brøndum-Nielsen, Allan Lind-Thomsen, Yuan Mang, Zahra El-Schich, Claire A. Boring, Mana M. Mehrjouy, Peter K.A. Jensen, Christina Fagerberg, Lotte N. Krogh, Jan Hansen, Thue Bryndorf, Claus Hansen, Michael E. Talkowski, Mads Bak, Niels Tommerup, Iben Bache

##### University of Copenhagen, Department of Cell. and Mol. Medicine Blegdamsvej 3B, DK-2200 Copenhagen Denmark

###### **Correspondence:** Iben Bache (ibache@sund.ku.dk)

Keywords: balanced chromosomal rearrangement, de novo, reciprocal translocation, inversion, prenatal diagnosis, long-term follow-up, morbidity risk, clinical recommendations

The 6%–9% risk of an untoward outcome previously established by Warburton for prenatally detected de novo balanced chromosomal rearrangements (BCRs) does not account for long-term morbidity. We therefore performed long-term follow-up (mean 17 years) of a registry-based nationwide cohort of 41 individuals carrying a prenatally detected de novo BCR with normal first trimester screening/ultrasound scan. A significantly higher frequency of neurodevelopmental and/or neuropsychiatric disorders than in a matched control group (19.5% versus 8.3%, p = 0.04) was observed, which was increased to 26.8% upon clinical follow-up. Chromosomal microarray of 32 carriers revealed no pathogenic imbalances, illustrating a low prognostic value when fetal ultrasound scan is normal. In contrast, mate-pair sequencing revealed disrupted genes (ARID1B, NPAS3, CELF4), regulatory domains of known developmental genes (ZEB2, HOXC), and complex BCRs associated with adverse outcomes. Two independent evaluations of the MPS-results, including a blinded evaluation, correctly predicted the clinical outcome in 5-6 of 11 affected carriers (sensitivity = 45-55%), and in all unaffected carriers (specificity = 100%) suggesting a significant value of MPS for interpretation of benign variation. However, the relatively low sensitivity might illustrate the challenges associated with prenatal risk prediction for long-term morbidity in the absence of phenotypic data given the still immature annotation of the morbidity genome and poorly understood long-range regulatory mechanisms. Seven carriers had unmappable autosomal;autosomal BCRs and were healthy, suggesting that BCRs involving pericentric/heterochromatic regions are associated with a low morbidity risk. In conclusion, we upwardly revise the previous estimates of Warburton to a morbidity risk of ~27% and recommend sequencing of the chromosomal breakpoints as the first-tier diagnostic test in pregnancies with a de novo BCR.

### L21 Satellite evolution in bovidae

#### Raquel Chaves^1,2^ (rchaves@utad.pt)

##### ^1^University of Trás-Os-Montes e Alto Douro, Department of Genetics and Biotechnology, CAG - Laboratory of Cytogenomics and Animal Genomics, 5000-801, Vila Real, Portugal; ^2^University of Lisboa, Faculty of Sciences, BioISI - Biosystems & Integrative Sciences Institute, 1749-016, Lisboa, Portugal

Centromeric regions remain the “dark matter” of current genome assemblies due to limitations of sequencing approaches, much due to the most abundant sequences in these regions: satellite DNA. These sequences consist of tandemly repeated DNA motifs, typically arranged in large arrays of hundreds or thousands of copies, located in blocks at the heterochromatic regions of chromosomes. Most eukaryotic species include multiple, unrelated, families of satDNA that differ in sequence, nucleotide composition, monomer unit length, abundance and chromosomal location. The Bovidae family, and specifically the Bovinae tribe, is an excellent model to study the evolution and complexity of centromeric satellite DNAs because of the great diversity of their satellite DNA families and impact in karyotype reorganization and chromosome evolution, resulting in the remodeling of the genomes of these species. In this talk, in an integrative approach, different aspects of the molecular nature and genomic behavior of Bovinae satellite DNA families identified in the bovine genome will be presented, including: organization, abundance, chromosome location, variation in sequence and evolutionary history. The genomic era, with the synergy between in silico and in situ approaches, opens up new perspectives, not only to reveal the fundamental features of satellite DNAs, but also to uncover a universal framework for understanding the roles of repetitive DNAs as a whole within the biology of chromosomes and genomes.

### L22 European certification and continuous education of clinical laboratory geneticists working in cytogenetics

#### Thomas Liehr (Thomas.Liehr@med.uni-jena.de)

##### Jena University Hospital, Institute of Human Genetics, Am Klinikum 1, Jena, Germany

There are four groups of specialists for human genetic diagnostics: Medical Geneticists (MDG), Genetic Nurses & Genetics Counsellors (GN/GC), Clinical Laboratory Geneticist (CLG) and Laboratory Genetics Technicians (LTG). While the first two groups are in direct patient contact, the latter two ones work ‘hidden’, not being less important, however, being invisible for the consultants. To make these professions more visible and also to make first steps towards uniform educational standards, the ESHG helped to give birth to the European Board of Medical Genetics (EBMG) in 2012. The “European Board of Medical Genetics division-CLG” was founded in 2014 with the goal to establish a unified European-based registration for individuals qualified in biomedicine. Recognition of the profession ‘European registered Clinical Laboratory Geneticist (ErCLG)’ based on a syllabus of core competences was established and initiated in 2015 with the goal to harmonize professional education. Five subtitles reflect the exact specialty of each ErCLG, who can reregister every 5 years. Interestingly, (Er)CLG profession includes numerous duties, which are quite similar in all regions of the world, like heading a lab, reporting, and teaching. Still CLG’s rights, like responsibly leading a lab, or responsibly signing a report are differently regulated according to country specific specificities. Here results of a survey on rights and duties of (Er)CLGs performed in 35 European and 18 non-European countries with 100 participating specialists are presented. Also the background of ErCLG registration is provided, and an update on the efforts for an official EU-recognition of the title based on EU Directives 2005/36/EC - policy developments and Proposal for modernizing the Professional Qualifications Directive = EU Directive 2013/55/EU is given.

### L23 Genomic Next Generation Sequencing and quality assurance: challenges and opportunities

#### Thomas Eggermann (teggermann@ukaachen.de)

##### Institute of Human Genetics, Aachen

Next Generation Sequencing (NGS) techniques of nucleic acids have evolved quickly since the last two decades and underlie permanent development and improvement. Many new platforms appear, but already established systems have vanished at the same time. However, NGS has become a basic tool of routine human genetic testing, and there is a further growing demand for higher throughput, lower costs and better quality of data. Whereas NGS based diagnostics was focused on the detection of basepair substitutions on DNA level in the beginning, NGS now becomes applicable for detection of copy number variations (CNVs) with the help of improved enrichment and bioinformatic solutions. Identification of structural variants is now also in the diagnostic focus, and it only a question of time that third generation/long read assays will allow the precise diagnostic characterisation of these disturbances. Additionally, the parallel analysis and comparison of genomic and transcriptomic data in the future will allow a significant improvement in determination of their functional relevance.

Despite these rapid and transient developments, standardized procedures and data formats are the obligate requisites for reliable, high quality and comparable genetic diagnoses. Accreditation measures have turned out to be adequate tools for quality assurance in NGS based diagnostic assays. Embedded in a general accreditation setting, quality measures are valuable tools to improve and streamline NGS pipelines, to make their use efficient and to provide high quality, comparable and transferable data and information. In these standardised NGS pipelines, all steps have to be considered and validated. In practice, the separate validation of the wetlab procedures, the data processing pipelines and the indication-specific analyses (e.g. region of interests) might be helpful, but accreditation also has to cover secondary quality parameters like confirmation of probe identity or variant verification. In fact, the quality parameters of wetlab assays should be the same for all labs, but the data processing and interpretation and the respective accrediation efforts depend on the aim of the service of a laborarty. In case it is restricted to data generation, the DIN ISO EN 17025 might be applied, whereas the interpretation and reporting of NGS results are subjects to the ISO/IECs 15189 (medical laboratories, including human genetics) or 17020 (pathology) and have to follow the respective guidelines and recommendations (e.g. EJHG guidelines, ACMG guidelines). In the talk, current standardization efforts and quality management will be overviewed and illustrated on the basis of examples.

### L24 Cell-free DNA analysis as a tool for “non-invasive cytogenetics”

#### Rossa W.K. Chiu (rossachiu@cuhk.edu.hk)

##### Li Ka Shing Institute of Health Sciences and Department of Chemical Pathology, Faculty of Medicine, The Chinese University of Hong Kong, Hong Kong SAR

Keywords: NIPT, cell-free fetal DNA, chromatin, chromosomal aneuploidies

Cell-free DNA analysis in maternal plasma for the screening of trisomies 21, 18 and 13 has led to substantial reductions in the numbers of chorionic villus sampling and amniocenteses being performed. On the other hand, maternal plasma cell-free DNA analysis are uncovering increasing cases of maternal cytogenetic abnormalities, rare autosomal trisomies in the fetus, true fetal mosaicism and confined placental mosaicism, which in turn led to the need for comprehensive cytogenetic workup. Chromosomal aberrations of cancers could also be detected by cell-free DNA analysis. Interestingly, recent studies revealed that the fragmentation patterns of cell-free DNA are reflective of the chromatin structure of the cell-of-origin. The analysis of such patterns, termed fragmentomics, could potentially provide a tissue map of the organs and cells contributing cell-free DNA and possibly the detection of diseases associated with those organs. (supported by the Hong Kong RGC Theme-based Research Scheme (T12-403/15N) and (T12-401/16-W) and a collaborative Research Grant from GRAIL).

### L25 The landscape of pathogenic copy number variations in healthy, reproducing females

#### Vancoillie Leen, Darine Villela, Brison Nathalie, Dehaspe Luc, Melotte Cindy, Dimitriadou Eftychia, Breckpot Jeroen, Peeters Hilde, Van Esch Hilde, Van Buggenhout Griet, Vogels Annick, de Ravel Thomy, Legius Eric, Devriendt Koen, Vermeesch Joris, Van Den Bogaert Kris

##### Center for Human Genetics, KU Leuven and UZ Leuven, Belgium

###### **Correspondence:** Vancoillie Leen (nathalie.brison@kuleuven.be)

Non-invasive prenatal testing (NIPT) is an efficient means to detect fetal aneuploidies. In our center, we use a genome-wide approach. Given that 90% of cell-free DNA is of maternal origin this also results in a genome-wide screening of the pregnant mothers for maternal copy number variations (CNV’s). These secondary variants may provide an opportunity for the pregnant mother and her fetus in optimizing follow-up and management. Since July 1, 2017, NIPT is reimbursed by the Belgian Social Security for all pregnant women resulting in a high uptake. We here report our one-year experience on reported maternal secondary variants in a series of 26,123 NIP-tests.

Autosomal rearrangements with a dominant effect were observed in 21 pregnancies (incidence 1/1243). These included five microdeletions involving a tumor suppressor gene (*NF1, BRIP1, MSH6, BRCA1* and *DICER1*). Four different genomic disorders were detected: the 3q29 microdeletion (n=1) , PWS/AS duplication (n=1), CMT1A (n=3) and HNPP (n=7). Two deletions included a gene causing a developmental disorder when deleted (ASXL3 (apparently non-mosaic); DNMT3A (mosaic)). Two large deletions were reported: a mosaic 17Mb 9q21 deletion and one non-mosaic 9.5Mb 4qter deletion. With regard to autosomal rearrangements with a recessive effect, 9 recurrent GJB6 microdeletions were reported.

Not unexpectedly, two thirds of actionable CNV’s were X-linked (65/95). The recurrent STS deletion was the most common (27 cases), followed by intragenic DMD deletions or duplications (n=11) and SHOX deletions (n= 4). Known XL-recessive disease genes that were found to be deleted include Factor 8, COL4A6/COL4A5, and four deletions involved a developmental disorder gene (IL1RAPL1, FRMPD4, ARSE, RPS6KA3 (n=2)). The recurrent int22h1/int22h2-mediated Xq28 duplication syndrome was observed 6 times; the reciprocal deletion 4 times. Six large X chromosomal deletions were detected.

These results show that 1/275 women carry a clinically significant CNV. Genome-wide NIPT has the potential of informing pregnant women on significant reproductive risks. However, this potential can only be fully reached on the condition that the interpretation is performed by an expert team and reporting is based on predefined guidelines with rapid access to follow-up genetic testing and high quality pre- and posttest counselling.

### L26 Chromoanagenesis: cataclysms behind complex chromosomal rearrangements

#### Franck Pellestor, Vincent Gatinois

##### Arnaud de Villeneuve Hospital, Montpellier CHRU, Unit of Chromosomal Genetics, Montpellier, France

###### **Correspondence:** Franck Pellestor (f-pellestor@chu-montpellier.fr)

During the last decade, genome sequencing projects in cancer genomes as well as in patients with congenital diseases and healthy individuals have led to the identification of new types of massive chromosomal rearrangements arising during single chaotic cellular events. These unanticipated catastrophic phenomenon are termed chromothripsis, chromoanasynthesis and chromoplexis., and are grouped under the name of “chromoanagenesis”.

For each process, several specific features have been described, allowing each phenomenon to be distinguished from each other and to understand its mechanism of formation and to better understand its aetiology. Thus, chromothripsis derives from chromosome shattering followed by the random restitching of chromosomal fragments with low copy-number change whereas chromoanasynthesis results from erroneous DNA replication of a chromosome through serial fork stalling and template switching with variable copy-number gains, and chromoplexy refers to the occurrence of multiple inter-and intra-chromosomal translocations and deletions with little or no copy-number alterations in prostate cancer. Cumulating data and experimental models have shown that chromothripsis and chromoanasynthesis may essentially result from lagging chromosome encapsulated in micronuclei or telomere attrition and end-to-end telomere fusion.

The concept of chromanagenesis has provided new insight into the aetiology of complex structural rearrangements, the connection between defective cell cycle progression and genomic instability, and the complexity of cancer evolution. Increasing reported chromoanagenesis events suggest that these chaotic mechanisms are probably much more frequent than anticipated.

### S1 Molecular cytogenetics – indispensability of the approach highlighted by characterization of small supernumerary marker chromosomes

#### Thomas Liehr, Ahmed Al-Rikabi

##### Jena University Hospital, Institute of Human Genetics, Jena, Germany

###### **Correspondence:** Thomas Liehr (Thomas.Liehr@med.uni-jena.de, Ahmed.Al-Rikabi@med.uni-jena.de)

There are major discussions going on at present in the field of genetics and genomics on whether – certain approaches may be outdated and should be replaced by more recent and modern ones. Especially in the field of human genetics, one easily finds papers claiming that banding and/or molecular cytogenetics could or should best be replaced by array comparative genomic hybridization (aCGH) and next generation sequencing (NGS) technologies. Often there is also the claim that aCGH is cheaper and NGS more reliable than the ‘old-fashioned’ approaches, even though both allegations have been already shown not (always) to be true. Here we discuss this problem and highlight the necessity to carefully select the methods by which patients should be studied. A good argument against the main stream opinion is the example of the infertile males with small supernumerary marker chromosomes (sSMCs). In these patients the sSMCs are derived predominantly from one of the acrocentric chromosomes, mainly chromosomes 15, 14, and 22; we recently showed that such sSMCs can be optimally characterized by single-cell directed (molecular) cytogenetics. A meta-analysis for detectability of sSMCs in these patients by aCGH revealed that up to 87% of the cases would not have been picked up by exclusive use of this method. Heterochromatic sSMCs would also be missed by NGS. Thus, this patient group is a model example showing that not all diagnostics are best done by non-chromosomal oriented studies. We therefore recommend some conservativeness in staying with, and remembering the advantages of the well-established standard approaches and using the available resources thoughtfully.

### S2 In memoriam: Professor Yuri B. Yurov — Molecular Cytogenetics (BMC-Springer/Nature) and beyond

#### Ivan Y Iourov^1,2^, Svetlana G Vorsanova^1,2^, Thomas Liehr^3^

##### ^1^Mental Health Research Center, Moscow, Russia; ^2^Veltischev Research and Clinical Institute for Pediatrics of the Pirogov Russian National Research Medical University; Moscow, Russia; ^3^Jena University Hospital, Friedrich Schiller University, Institute of Human Genetics, Jena, Germany

###### **Correspondence:** Ivan Y Iourov (Thomas.Liehr@med.uni-jena.de)

On 12 December, 2017, Professor Yuri Yurov passed away after courageous fight against a devastating disease. Yuri’s life is a picturesque example of unselfish devotion to science. His contribution to genetics and, more specifically, cytogenetics is impossible to overestimate. To mention briefly, Yuri’s research has made immense contribution to applied molecular cytogenetics, FISH-based genome research, studies of somatic mosaicism, neurocytogenetics, cyto(post)genomics etc. A serous part of Yuri’s life has been Molecular Cytogenetics, the journal founded by him and his colleagues (i.e. authors of this abstract, plus Lisa G Shaffer) in 2008. This journal has been dedicated to chromosome biology and molecular cytogenetics based on a principle, something like “good music for good people” being, however, a scientific journal. Yuri has become one of the Editors-in-Chief as one of the founders. It has taken 5 years to receive the first official impact factor and to be ranked first out of all cytogenetic journals in 2013. Yuri has considered Molecular Cytogenetics’ editorship an honorable duty. We are only starting to understand the extent of his contribution to the journal’s success. Our loss is untimely and unacceptable. For more details, please refer to 10.1186/s13039-018-0383-3 (Iourov IY, Vorsanova SG. Yuri B. Yurov (1951-2017). Molecular Cytogenetics. 2018; 11:36). Here we want to remember Yuri as a scientist and good friend and at the same time reflect about a decade of Molecular Cytogenetics which has been extremely productive. Numerous highly cited and recognized contributions in the fields of clinical cytogenomics, oncocytogenetics, somatic mosaicism, neurocytogenetics, chromosomal evolution and chromosome biology have been published. Accordingly, we invite new contributions encompassing all aspects of chromosome biology and the application of molecular cytogenetic techniques in all areas of biology and medicine to our journal.

## Oral Abstracts

### O1 A simple universal and cost efficient dPCR method for the targeted analysis of copy number variations

#### Kévin Cassinari^1^, Olivier Quenez^1^, Géraldine Joly-Hélas^1^, Ludivine Beaussire^1^, Nathalie Le Meur^1^, Mathieu Castelain^1^, Alice Goldenberg^1^, Anne Marie Guerrot^1^, Anne Claire Brehin^1^, Jean-François Deleuze^2^, Anne Boland^2^, Anne Rovelet-Lecrux^1^, Dominique Campion^1^, Pascale Saugier-Veber^1^, Nicolas Gruchy^3^, Thierry Frebourg^1^, Gael Nicolas^1^, Nasrin Sarafan-Vasseur^1^, Pascal Chambon^1^

##### ^1^Rouen University Hospital And Normandie Univ, Unirouen, Inserm U1245, Normandy Center for Genomic and Personalized Medicine, Department of Genetics, Rouen-France; ^2^Centre National De Recherche En Génomique Humaine, Cea, Institut De Génomique, Evry-France, Metropolitan; ^3^Caen University Hospital and Normandy Center for Genomic And Personalized Medicine, Department of Genetics, Caen-France

###### **Correspondence:** Pascal Chambon (pascal.chambon@chu-rouen.fr)

Background. Rare copy number variations (CNVs) are a major cause of genetic diseases. Simple targeted methods are required for their confirmation and segregation analysis. We developed a simple and universal CNV assay based on Digital PCR (dPCR) and universal Locked Nucleic Acid (LNA)-hydrolysis probes.

Methods. We analyzed the mapping of the 90 LNA hydrolysis probes from the Roche Universal Probe Library (UPL). For each CNV, selection of the optimal primers and LNA probe is almost automated, probes are reused across assays and each dPCR assay includes the CNV amplicon and a reference amplicon. We assessed the assay performances on 93 small and large CNVs and performed a comparative cost-efficiency analysis.

Results. UPL-LNA probes presented nearly 20,000,000 occurrences on the human genome and were homogeneously distributed with a mean interval of 156 bp. The assay accurately detected all the 93 CNVs, except one (<200 pb), with variation coefficients below 10%. The assay was shown to be more cost-efficient than all the other methods.

Conclusion. The universal dPCR CNV assay is simple, robust and cost-efficient as it combines a straightforward design allowed by universal probes and endpoint PCR, the advantages of a relative quantification of the target to the reference within the same reaction, and the high specificity of the LNA-hydrolysis probes. This method should be a useful tool for genomic medicine, which requires simple methods for the interpretation and segregation analysis of genomic variations

### O2 Next Generation Mapping a novel approach that enables the detection of unbalanced as well as balanced structural variants

#### Laïla El Khattabi^1^, Caroline Schluth-Bolard^2^, Nicolas Chatron^2^, Imane Baatout^1^, Aziza Lebbar^1^, Martine Montagnon^1^, Yannis Duffourd^3^, Antonio Vitobello^4^, Patrick Callier^4^, Jean François Deleuze^5^, Damien Sanlaville^2^, Jean Michel Dupont^1^

##### ^1^Aphp Hôpital Cochin; Paris Descartes University, Cochin Institute U1016, Cytogenetics, Paris-France; ^2^Hospices Civils De Lyon, Lyon, France Et Gendev Team, Inserm U1028; Cnrs Umr5292; Ucbl1;crnl, Genetics, Bron-France; ^3^Inserm Umr 1231 Gad, Faculty of Health Sciences, University Of Burgundy And Franche-Comté, Genetics, Dijon-France; ^4^Inserm UMR 1231 Gad, Faculty of Health Sciences, University Of Burgundy And Franche-Comté, Molecular and Chromosomal Genetics Laboratory, Biology Transfer Platform, Dijon-France; ^5^Reference Center for Research on Human Genomics, Francois Jacob Institute, Cea, Genomics, Evry-France

###### **Correspondence:** Laïla El Khattabi (laila.el-khattabi@aphp.fr)

Structural variants (SVs) include large unbalanced (CNVs) and balanced variants (insertions, inversions and translocations). Whereas the detection of unbalanced SVs has been significantly improved by technological breakthrough such as Chromosomal Microarray Analysis (CMA), the detection of balanced SVs still relies on karyotype despite its very low resolution. Massively parallel sequencing enables the detection of some SVs but its use in clinical setting is yet limited by technical and computational challenges, among which the read length.

Next Generation Mapping using the Bionano system is a novel non-sequencing based technology. Long high molecular weight DNA fragments are labelled at specific sites and then stretched out into a nano-channel system for fluorescence reading. The labelling pattern is then compared to a reference genome pattern allowing for the identification of SVs without complex bioinformatic analyses. We sought to evaluate the performance of this technology and its ease of use in a routine cytogenetic laboratory. Our study includes 29 patients bearing balanced (11 translocations and 4 inversions) or unbalanced SVs (1 unbalanced translocation, 7 CNVs ranging from 500kb to 4Mb), complex chromosomal rearrangements (n=4), isochromosomes (n=2) and one case of aneuploidy, all previously identified by karyotype or CMA. The results are analysed blindly and then compared to karyotype or CMA results. Preliminary data on four samples show reliable detection of the expected SVs.

This approach has the potential to improve the resolution of the pangenome detection of different sorts of SVs, and could hence complement or even replace karyotype and CMA as a unique, simple and comprehensive test. This would have a significant clinical impact for diseases in which balanced SVs are mainly involved, such as reproductive diseases and recurrent miscarriages.

### O3 Low coverage whole genome sequencing in plasma circulating cell free DNA analysis the Turner syndrome experience

#### Paolo Reho^1^, Aldesia Provenzano^1^, Debora Vergani^1^, Daniela Larizza^2^, Chiara Montalbano^2^, Miriam Carella^3^, Andrea La Barbera^1^, Lucia Tiberi^1^, Emanuele Bosi^1^, Angelica Pagliazzi^1^, Daniela Formicola^1^, Marilena Pantaleo^4^, Silvia Guarducci^4^, Sabrina Giglio^4^, Orsetta Zuffardi^3^

##### ^1^University of Florence, Experimental And Clinical Biomedical Sciences, Florence-Italy; ^2^Irccs Policlinico “san Matteo” Foundation, Pediatric Endocrinology Unit, Pavia-Italy; ^3^University of Pavia, Molecular Medicine, Pavia-Italy; ^4^Meyer Children's University Hospital, Medical Genetics Unit, Florence-Italy

###### **Correspondence:** Paolo Reho (paolo.reho@gmail.com)

Turner syndrome (TS) is characterized by complete or partial absence of the second sex chromosome, either in a mosaic form or in all cells, in phenotypic female patients.

Y chromosome is detected in 10-12% of TS cases and is associated with an increased gonadoblastoma risk, thus gonadectomy is mandatory before starting GH treatment. The detection of Y chromosome, at low-level mosaic or as marker chromosome, may be tricky by standard karyotype (SK) or FISH, to date the gold-standard methods for TS diagnosis. Therefore, molecular screening to detect Y-chromosomal sequences is strongly recommended in patients with masculine features who are negative by these approaches.

We performed low-coverage (0.2x) whole genome sequencing (lc-WGS) of plasma cell-free DNA (cf-DNA) to determine its potential role in TS diagnosis.

Our study was performed on 64 TS patients, previously characterized by SK and array-CGH analysis. Genome coverage information from healthy controls was used as reference to identify chromosomal structural variants.

We identified low-level mosaicism for XX or XY cell lines, partial deletions/duplications of sex chromosomes indicating interstitial deletions, iso-chromosomes or ring/marker chromosomes.

Lc-WGS analysis confirmed SK results in 50/64 cases, while we detected nine X chromosome rearrangements at low-level mosaic that, although hidden at SK, were confirmed by array-CGH in all but two cases. Moreover, previously unreported Y chromosome material was found in five patients.

Interestingly, in two patients dd-PCR assay confirmed the presence of Y chromosome in cf-DNA but not in genomic-DNA, suggesting that some chromosomal abnormalities may be detected only through cf-DNA analysis.

Our results show that, in clinical suspicion of TS, lc-WGS of cf-DNA is a cheap and valuable screening test for the detection of low-level mosaicism and complex structural chromosome abnormalities, whether they be germline or somatic.

### O4 Chromosome radial positioning in spermatogenic germ cells from mus musculus

#### Mireia Solé^1^, Joan Blanco^1^, Debora Gil^2^, Oliver Valero^3^, Gothami Fonseka^4^, Richard Frodsham^4^, Francesca Vidal^1^, Zaida Sarrate^1^

##### ^1^Universitat Autònoma De Barcelona, Biologia Cel·lular, Fisiologia I Immunologia, Barcelona-Spain; ^2^Computer Vision Center, Computer Science Department, Barcelona-Spain; ^3^Universitat Autònoma De Barcelona, Servei D'estadística Aplicada, Barcelona-Spain; ^4^Tecnopark, Cytocell Ltd, Cambridge-Spain

###### **Correspondence:** Mireia Solé (Mireia.Sole@uab.cat)

Chromosomes occupy specific regions of the nucleus called chromosome territories. In somatic cells, the radial positioning of the chromosomes is conditioned by size, gene density and transcriptional activity. These associations suggest the existence of a functional relationship between chromosomal territoriality and gene expression. Little is known about chromosomal positioning during spermatogenesis. Some authors have suggested that the chromosomes distribution in spermatogenic cells could affect spermatogenesis development and have implications for embryo gene expression. The objective of this work was to describe the radial chromosomal territoriality in spermatogenic cells from C57BL/6J Mus musculus and to identify their conditioning factors. Spermatogenic cells from disaggregated testicular tissue were fixed in polylysine slides to preserve the three-dimensionality of the nuclei. Fluorescence in situ hybridization of all mouse chromosomes were performed and 3D image captures were done by confocal microscopy. Images were analyzed using Matlab scripts. After the identification of cells by immunofluorescence procedures, five spermatogenic stages were analyzed: spermatogonia-early preleptotene, mid preleptotene-zygotene, pachytene, round spermatids and spermatozoa. Results showed that radial distribution of chromosomes is dynamic during spermatogenesis but non-random. Our data suggest that the bouquet formation affects the radial chromosome positioning at pachytene stage in accordance to chromosomal size, being the smallest chromosomes preferably located in the nucleus periphery. Moreover, several results suggest that gene activity is closely associated with the radial positioning of chromosomes throughout the spermatogenesis process. In all the stages analyzed, the interior of the nucleus presents a higher concentration of genetic material than the expected value. In addition, chromosomes with more number of genes involved spermatogenesis process or spermiogenesis (in round spermatids), show more chromosomal volume in the middle-internal area of the nucleus. Finally, we have observed that sex chromosomes present a dynamic radial positioning during spermatogenesis, directly related to their activation/inactivation state.

### O5 From cytogenetics to cytogenomics whole genome sequencing as a comprehensive genetic test in rare disease diagnostics

#### Jesper Eisfeldt^1^, Johanna Lundin^1^, Maria Pettersson^1^, Malin Kvarnung^1^, Agne Lieden^1^, Ellika Sahlin^1^, Kristina Lagerstedt^1^, Marcel Martin^2^, Sofia Ygberg^3^, Olof Bjerin^3^, Henrik Stranneheim^4^, Anna Wedell^4^, Magnus Nordenskjöld^1^, Maria Johansson Soller^1^, Ann Nordgren^1^, Valtteri Wirta^5^, Daniel Nilsson^1^, Anna Lindstrand^1^

##### ^1^Karolinska University Hospital, Department of Clinical Genetics, Stockholm-Sweden; ^2^Nbis, , Stockholm-Sweden; ^3^Karolinska Institute, The Institution for Women's and Children's Health, Neuropediatric Unit, Stockholm-Sweden; ^4^Karolinska Institutet, Department of Molecular Medicine and Surgery, Stockholm-Sweden; ^5^Kth Royal Institute of Technology, Scilifelab, School of Engineering Sciences in Chemistry, Biotechnology and Health, Stockholm-Sweden

###### **Correspondence:** Jesper Eisfeldt (jesper.eisfeldt@scilifelab.se)

Rare genetic diseases are caused by different types of genetic variants, from single nucleotide variants (SNVs) to large chromosomal rearrangements. Recent data indicates that whole genome sequencing (WGS) may be used as a comprehensive test to identify multiple types of pathologic genetic aberrations in a single experiment.

We present FindSV, a bioinformatic pipeline for detection of balanced (inversions and translocations) and unbalanced (deletions and duplications) structural variants (SVs). First, FindSV was tested on 106 validated deletions and duplications with a median size of 850 kb (Min: 511 bp, Max: 155 Mb). All variants were detected. Second, we demonstrated the clinical utility in 138 monogenic WGS panels increasing the diagnostic rate with 8%. Remarkably, a complex structural rearrangement involving two clustered deletions disrupting SCN1A, SCN2A, and SCN3A was identified in a three months old girl with epileptic encephalopathy. Finally, 100 consecutive samples referred for clinical microarray were also analyzed by WGS. The WGS data was processed for large (>5000bp) SVs genome wide, visualized with our newly developed tool vcf2cytosure, and for exonic SVs and SNVs in a panel of 700 genes linked to intellectual disability. We also applied short tandem repeat (STR) expansion detection and discovered one pathologic expansion in ATXN7. The overall diagnostic rate (27%) was more than doubled compared to clinical microarray (10%).

In conclusion, using WGS we detected a wide range of structural variation with high accuracy. Since WGS also allowed for analysis of SNVs and STRs this represents a powerful comprehensive genetic test in a clinical diagnostic laboratory setting.

### O6 Cytogenetically visible inversions are formed by multiple molecular mechanisms

#### Maria Pettersson^1^, Christopher M. Grochowski^2^, Josephine Wincent^1^, Jesper Eisfeldt^1^, Sau Wai Cheung^2^, Ana Cristina Victorino Krepischi^3^, Carla Rosenberg^3^, James R. Lupski^2^, Jesper Ottosson^4^, Lovisa Lovmar^4^, Jelena Gacic^5^, Elisabeth Syk Lundberg^1^, Daniel Nilsson^1^, Claudia M.b. Carvalho^2^, Anna Lindstrand^1^

##### ^1^Karolinska Institutet, Department of Molecular Medicine and Surgery, Stockholm-Sweden; ^2^Baylor College of Medicine, Department of Molecular And Human Genetics, Houston, Tx-United States; ^3^Institute of Biosciences, University of Sao Paulo, Department of Genetics and Evolutionary Biology, Sao Paulo-Brazil; ^4^Sahlgrenska University Hospital, Department of Clinical Genetics, Gothenburg-Sweden; ^5^Linköping University Hospital, Department of Clinical Genetics, Linköping-Sweden

###### **Correspondence:** Anna Lindstrand (maria.pettersson.1@ki.se)

Cytogenetically detected chromosomal inversions are generally assumed to be copy number and phenotypically neutral events. Early cytogenetic studies of inversions suggested that non-allelic homologous recombination (NAHR) between inverted repeats at both breakpoints could be the underlying mechanism of formation and the prevalence of NAHR-mediated inversions has been estimated to 67%.

In the present study, we used short-read whole-genome sequencing (WGS) and/or Sanger sequencing on 16 unique (24 in total) cytogenetically detected chromosomal inversions and were able to characterize 11/16 (69%) on the nucleotide level. We found that two seemingly recurrent inversions were identical by descent and follow-up analysis using the WGS data confirmed that the unrelated carriers shared both common and more rare haplotypes on the chromosomes with inversions. All inversions that were characterized in detail generally showed little to no microhomology in the breakpoint junctions, similar to what is commonly seen in reciprocal translocations and mutational signatures were consistent with non-homologous end-joining (NHEJ) or microhomology-mediated end-joining (MMEJ) (9/11, 82%) and fork-stalling and template switching/microhomology-mediated break-induced replication (FoSTeS/MMBIR) (2/11, 18%). Finally, the gene disruption frequency was similar to the frequency obtained for balanced translocations (7/27 breakpoints, 26%). In summary, the study indicates that high-coverage short-read WGS can detect a substantial fraction of copy number neutral inversions and resolve the breakpoints on the nucleotide level, and NAHR is likely not the major mechanism underlying the formation of large chromosomal inversions, as at least 69% (11/16) of inversions were mediated through other mechanisms than ectopic recombination.

### O7 Next generation sequencing detects premeiotic errors in human oocytes and provides evidence of genetic influence

#### Harita Ghevaria^1^, Sioban Sengupta^1^, Roy Naja^2^, Rabi Odia^3^, Paul Serhal^4^, Xavier Viñals Gonzalez^3^, Joy Delhanty^1^

##### ^1^Preimplantation Genetics Group, Institute For Women’s Health, University College London, London-United Kingdom; ^2^Molecular Genetics Laboratory, Igenomix Uk Ltd, London-United Kingdom; ^3^Embryology Department, The Centre for Reproductive And Genetic Health, London-United Kingdom; ^4^Clinical Department, The Centre For Reproductive and Genetic Health, London-United Kingdom

###### **Correspondence:** Harita Ghevaria (h.ghevaria@ucl.ac.uk)

Background: Oogenesis is an error prone process. As women age the risk of an aneuploid oocyte increases with most errors affecting meiosis I or II. The application of next generation sequencing (NGS) has allowed us to confirm our earlier finding, from molecular cytogenetic studies, that a significant proportion of apparently meiotic aneuploidy is in fact present in the early embryo, leading to a high risk of oocyte aneuploidy irrespective of age. It has also provided evidence that genetic factors influence premeiotic oocyte aneuploidy.

Methods/Participants: The oocyte DNA was extracted, amplified and NGS was performed using the Ion ReproSeqPGS Kit (ThermoFisher Scientific) or VeriSeqPGS Kit (Illumina). Immature oocytes included 29 Germinal Vesicles(GV), 11 Metaphase I (MI), 4 GV/MI stage oocytes. Mature oocytes included 20 Metaphase II – 1st polar body (MII-PB1) complexes where both cells were analysed together and 4 analysed separately. In total, 68 oocytes from 18 women, (average mat age 34.83 years) were tested.

Results : Only four of the women were infertile; most oocytes were donated from cycles of egg freezing for social reasons; two young women were preserving oocytes due to breast cancer treatment. Overall 11 of 68 (16%) oocytes showed premeiotic (PM) errors. Of the 18 patients 6 had oocytes with PM errors; none were infertile. From a total of 27 PM abnormalities, 19 were in five of 10 oocytes from the two cancer patients. In contrast 14 oocytes from one woman for social egg freezing were all euploid.

Conclusion: We conclude that the application of NGS has provided accurate information regarding the frequency of aneuploidy that is due to premeiotic errors compared with that caused by errors at MI of oogenesis and that on an individual basis this is influenced by genetic factors.

### O8 Expression studies in patients with Shwachman Diamond syndrome in relation to clonal chromosome anomalies in bone marrow

#### Roberto Valli^1^, Antonella Minelli^2^, Abdul Waheed Khan^1^, Annalisa Frattini^3^, Alessia Bogni^4^, Giovanni Porta^1^, Francesco Acquati^5^, Cesare Danesino^2^, Emanuela Maserati^1^, Francesco Pasquali^1^

##### ^1^University of Insubria, Department of Medicine and Surgery, Varese-Italy; ^2^University of Pavia, Department of Molecular Medicine, Pavia-Italy; ^3^National Council of Research, Uos Milano Irgb, Milano-Italy; ^4^Dg Joint Research Centre, Jrc, Ispra-Italy; ^5^University of Insubria, Dept. Of Biotechnology, Varese-Italy

###### **Correspondence:** Roberto Valli (roberto.valli@uninsubria.it)

Shwachman-Diamond syndrome (SDS), autosomal recessive bone marrow failure condition, implies a high risk of developing myelodysplastic syndrome and acute myeloid leukaemia. Two clonal chromosome changes are frequent in the bone marrow (BM): an isochromosome of the long arm of chromosome 7, i(7)(q10), and an interstitial deletion of the long arm of chromosome 20, del(20)(q). Both these clonal anomalies were shown to imply a positive prognostic role, due to the duplication of a mutation with milder effect, in the i(7)(q10), and to the loss of the EIF6 gene, in del(20)(q).

Since 1999, we follow-up 97 Italian patients with SDS. We report the expression analysis of bone marrow (BM) cells of patients with SDS, in relation to the presence of clonal chromosome anomalies: del(20)(q) (five cases), i(7)(q10) (one case), other anomalies (two cases). The study was performed by microarray technique considering the whole transcriptome (WT), and three gene subsets, selected as relevant in BM functions. The results were compared with those of nine patients with SDS without clonal anomalies, and of nine healthy subjects. There is a significant difference between gene expression in BM of SDS patients and healthy subjects, both at level of WT and of the gene sets selected. The deletion del(20)(q), with the gene EIF6 consistently lost, even in patients with the smallest losses of material, changes the transcription pattern: a low proportion of abnormal cells lead to a pattern similar to SDS patients without acquired anomalies, whereas a high proportion yields to a pattern similar to healthy subjects. Hence, the benign prognostic value of the del(20)(q). The only case of i(7)(q10) showed a transcription pattern similar to healthy subjects, paralleling the positive prognostic role of this anomaly as well.

### O9 Three Dimensional Telomere Organization in papillary thyroid cancers

#### Paola Caria^1^, Tinuccia Dettori^2^, Aline Rangel Pozzo^3^, Daniela Virginia Frau^1^, Daniel Lichtenzstejn^4^, John Gartner^5^, Roberta Vanni^1^, Sabine Mai^3^

##### ^1^University of Cagliari, Dept of Biomedical Sciences, Monserrato-Italy; ^2^University of Cagliari, Ept of Biomedical Sciences, Monserrato-Italy; ^3^University of Manitoba, Cell Biology, Research Institute of Oncology And Hematology,cancercare, Winnipeg-Canada; ^4^University of Cagliari Manitoba, Cell Biology, Research Institute of Oncology And Hematology,cancercare, Winnipeg-Canada; ^5^University of Manitoba, Department of Pathology And Immunology, Max Rady College of Medicine, Rady Faculty of Health Sciences, Winnipeg-Canada

###### **Correspondence:** Paola Caria (paola.caria@unica.it)

Well-differentiated thyroid cancers (WDTCs) are the most common endocrine malignancy and papillary thyroid cancer (PTC) represents a large group of WDTC with two main histologic variants: classic-PTC (cPTC), and follicular variant PTC (FV-PTC). FV-PTC is divided into two sub-groups based on two different morphological aspects: infiltrative and encapsulated nodules. Recently, the encapsulated variant has been reclassified as noninvasive follicular thyroid neoplasm with papillary-like nuclear features (NIFTP), because it shows features similar to non-malignant lesions. At the molecular level, PTC may show BRAF mutations and/or RET7PTC rearrangements (BRAF-like nodules) and RAS mutations (RAS-like nodules). Several studies using quantitative three-dimensional (3D) telomere imaging have shown that cancer cells have an altered 3D telomere organization, in contrast to normal cells. In thyroid cancer, this aspect has not been yet fully investigated. To evaluate if specific telomere architecture may characterize PTC histologic variants, quantitative fluorescence in situ hybridization (Q-FISH), 3D imaging and 3D analysis were performed in 16 thyroid lesions:5 cPTC, 3 FV-PTC, 4 NIFTP and 4 FTA (follicular thyroid adenoma), using normal thyroid tissue (NT) as control. Moreover, we investigated RET/PTC rearrangements and BRAF expression (indicative of BRAFV600E mutation) by FISH and immunofluorescence, respectively. We found different telomere profiles in tumors compared to control (p<0.05), and telomere profiles of FTA close to NT. The comparison of 3D telomere profiles of the tumors demonstrated that NIFTP has longer telomeres than cPTC and FV-PTC (p<0.001). No correlation between molecular alterations and 3D telomere profiles was observed. These data suggest that 3D telomere organization might have diagnostic utility and might help the clinical management of NIFTP, which has a still unclear characterization and an outcome often difficult to predict. Supported by FIR2018 and CancerCare Manitoba.

### O10 Patterns of acquired uniparental disomy reveal biallelic inactivation of tumor suppresssor genes in gastrointestinal cancers and in colorectal advanced adenomas

#### Eva Hernández-Illán^1^, Pau Erola^2^, Keyvan Torabi^3^, Maria Isabel Alvarez-Mora^4^, Marcos Díaz-Gay^1^, Queralt Ferrer^1^, Antoni Castells^1^, Sergi Castellví-Bel^1^, Montserrat Milà^4^, Immaculada Ponsa^3^, Juanjo Lozano^2^, Rosa Miró^3^, Jordi Camps^1,3^

##### ^1^Gastrointestinal and Pancreatic Oncology Group, Institut D'Investigacions Biomèdiques August Pi i Sunyer (IDIBAPS), Centro de Investigación Biomédica en Red de Enfermedades Hepáticas y Digestivas (CIBEREHD), Barcelona, Catalonia, 08036, Spain; ^2^Bioinformatics Unit, CIBEREHD, Barcelona, Catalonia 08036, Spain; ^3^Unitat de Biologia Cel·lular i Genètica Mèdica, Departament de Biologia Cel·lular, Fisiologia i Immunologia, Facultat de Medicina, Universitat Autònoma de Barcelona, Bellaterra, Catalonia, 08193, Spain; ^4^Biochemistry and Molecular Genetics Department, Hospital Clínic, IDIBAPS, Centro de Investigación Biomédica en Red de Enfermedades Raras (CIBERER), Barcelona, Catalonia, 08036, Spain

###### **Correspondence:** Jordi Camps (jcampspo@gmail.com)

**Abstract**

Somatically-acquired uniparental disomies (aUPDs), also known as copy number neutral loss of heterozygosity (cnLOH), are frequent events in solid tumors and have been associated with cancer-related genes. Here, we aimed at integrating aUPD profiles with whole-exome sequencing mutational data in a tumor-type specific manner. Using TCGA datasets for 1,032 gastrointestinal cancers, including colon (COAD), rectum (READ), stomach (STAD), esophageal adenocarcinoma (EAC) and esophageal squamous cell carcinoma (ESCC), we show that gastrointestinal cancers show tumor-type specific profiles of aUPD. By inferring genome ploidy, we demonstrate that an increased number of aUPD events, both affecting the whole chromosome or segments of it, were present in samples with higher DNA ploidy compared to near-diploid tumors. The integration of whole-exome sequencing and aUPD provides evidence of biallelic inactivation of tumor suppressor genes and activation of oncogenes in a tumor-type specific manner. Of note, APC was the most recurrently inactivated gene in COAD and READ by the presence of homozygous mutations as a consequence of aUPD. Likewise, ARID1A and NOTCH1 were biallelically inactivated by aUPD in STAD and ESCC, respectively. Furthermore, while TP53 showed inactivation caused by aUPD at chromosome arm 17p across all tumor types, copy number losses at this genomic position were also frequent. When we studied the presence of aUPD in premalignant lesions of the colorectum, i.e., advanced adenomas, we observe that 31% of samples showed aUPD at chromosome arm 5q. Intriguingly, all samples with aUPD displayed a homozygous mutation in APC. Finally, the presence of mosaic UPD was detected at a higher frequency in peripheral blood lymphocytes of patients with colorectal cancer in a case-control study. In summary, our study defines specific profiles of aUPD in gastrointestinal cancers and provides unequivocal evidence to achieve biallelic inactivation of tumor suppressor genes in cancer and in premalignant lesions.

**Keywords:** uniparental disomy, copy-number alterations, gastrointestinal cancers, colorectal advanced adenomas, single nucleotide variants, ploidy, mosaicism.

### O11 ZNF384 gene fusions in B ALL A report of fifteen Austrian cases secured by systematic FISH and array screening

#### Sabrina Haslinger^1^, Karin Nebral^1^, Andrea Inthal^1^, Margit König^1^, Dagmar Schinnerl^2^, Andishe Attarbaschi^3^, Georg Mann^3^, Sabine Strehl^2^, Oskar A. Haas^1^

##### ^1^Children's Cancer Research Institute, St. Anna Kinderkrebsforschung, Clinical Genetics, Vienna-Austria; ^2^Children's Cancer Research Institute, St. Anna Kinderkrebsforschung, Genetics of Leukaemias, Vienna-Austria; ^3^St. Anna Children’s Hospital, Medical University of Vienna, Department of Pediatric Hematology And Oncology, Vienna-Austria

###### **Correspondence:** Sabrina Haslinger (sabrina.haslinger@ccri.at)

The subdivision of childhood B-cell precursor acute lymphoblastic leukemia (BCP-ALL), based on specific genetic features, such as gene fusions or ploidy and copy number aberrations, provides the basis for treatment stratification and decisions. The so-called "B-other" group embraces all cases with rare recurrent abnormalities that are hitherto less well-defined. Because they include potential candidates for targeted and personalized therapies, they are currently the main focus of interest. One of these recently identified subgroups that accounts for approximately 4% of B-ALL and up to 10% of B-other cases, involves the ZNF384 gene, which is fused to at least ten different partners. These cases have commonly a CD10 negative (pro-B/BI) or CD10low immunophenotype with myeloid markers and a distinct gene expression pattern. To search for ZNF384 positive cases we screened all B other cases that were enrolled in the ALL-BFM 2009 study with SNP/CGH arrays as well as an additional selected cohort with a ZNF384-specific dual color break apart FISH probe set. We found fifteen patients with a ZNF384 fusion, which make-up approximately 5% of all B-other cases. Eight of them had an EP300-ZNF384, three a TCF3-ZNF384 and one had an EWSR1-ZNF384 fusion. The remaining three had novel fusion partners, two of which were ascertained as CCAR1 and NIPBL. The other one will hopefully identified with whole transcriptome RNA-sequencing, which is currently performed in all cases. Four of them had IKZF1 deletions and all but one are in remission, supporting the notion that ZNF384 positive cases seem to respond well to current therapies.

### O12 Identification of cryptic aberrations allows more accurate prognostic classification of patients with myelodysplastic syndromes and clonal evolution

#### Karla Svobodova^1^, Halka Lhotska^1^, Lucie Hodanova^1^, Lenka Pavlistova^1^, Denisa Vesela^1^, Monika Belickova^2^, Jitka Vesela^2^, Jana Brezinova^2^, Iveta Sarova^2^, Silvia Izakova^1^, Libuse Lizcova^1^, Magda Siskova^3^, Anna Jonasova^3^, Jaroslav Cermak^2^, Kyra Michalova^1^, Zuzana Zemanova^1^

##### ^1^Institute of Medical Biochemistry and Laboratory Diagnostics, General University Hospital and 1st Faculty of Medicine of Charles University, Center of Oncocytogenetics, Prague-Czech Republic; ^2^Institute of Hematology and Blood Transfusion, Institute of Hematology And Blood Transfusion, Prague-Czech Republic; ^3^General University Hospital and First Faculty Of Medicine of Charles University, 1st Medical Department, Prague-Czech Republic

###### **Correspondence:** Karla Svobodova (karla.svobodova@vfn.cz)

Myelodysplastic syndromes (MDS) are a heterogenous group of clonal hematological malignancies. In patients with MDS, the karyotype of bone-marrow cells at the time of diagnosis is one of the most important prognostic factors. In some MDS cases, acquisition of additional genetic aberrations (clonal evolution, CE) associated with clinical progression may occur during the disease. The aim of the study was to identify cryptic aberrations in MDS patients and to assess their potential role in clonal evolution.

Karyotypes were analyzed with conventional G-banding, I-FISH/mFISH/mBAND (Abbott, MetaSystems) and aCGH/SNP microarray (Illumina, Agilent). Mutational analysis of MDS associated genes was performed using NGS VariantPlex Myeloid Panel (ArcherDX) on the NextSeq System (Illumina) or amplicon deep sequencing on a Roche 454 GS Junior (Roche).

We confirmed CE in 36/469 MDS patients. The analysis of bone-marrow samples with combination of cytogenomic methods at diagnosis and after CE identified 217 chromosomal aberrations. The early genetic changes in the diagnostic samples were frequently MDS specific (19 MDS-specific/55 early changes). Most progression-related aberrations identified after CE were MDS non-specific (134 non-MDS-specific/160 progression-related changes). Copy number neutral loss of heterozygosity (CN-LOH) was detected in 19% of patients. MDS-specific CN-LOH (4q, 17p) was identified in three patients, and probably pathogenic homozygous mutations in TET2 (4q24) and TP53 (17p13.1) genes were also confirmed. We observed a statistically significant difference in overall survival between the groups of patients divided according to their diagnostic cytogenomic findings (p = 0.021), with worse OS in the group with complex karyotypes.

A combination of cytogenomic methods allows detection of many cryptic genomic changes and contributes to the identification of genes/genomic regions that offer potential therapeutic targets in patients with progressive MDS.

### O13 Cytogenetic and genomic assays in river buffalo (Bubalus bubalis, 2n=50) cows raised in urban and rural areas

#### Alessandra Iannuzzi^1^, Viviana Genualdo^1^, Angela Perucatti^1^, Cristina Rossetti^1^, Domenico Incarnato^1^, Anna Caputi-Jambrenghi^2^, M A Colonna^2^, Francesco Giannico^2^, Leopoldo Iannuzzi^1^

##### ^1^CNR-ISPAAM Agri-Food Naples-Italy; ^2^University of Bari Agricultural and Environmental Sciences Bari-Italy

###### **Correspondence:** Alessandra Iannuzzi (leopiannuzzi949@gmail.com)

The exposition to exogenous agents can lead to a variety of modifications on DNA, resulting in genome alterations. Aim of this study is to verify the differences between long- and short-term DNA damage in river buffalo lymphocytes by the following test: CA (chromosome and chromatid breaks), Sister Chromatid Exchanges (SCEs) and Cytokinesis-Block Micronucleus (CBMN) for long-term DNA damage test, and the relative Telomeres Length (TL), by Monochrome Multiplex Quantitative PCR (MMQPCR) method, for short-term DNA damage. Two group of buffaloes (20 animals per group, homogeneous for age, sex and feeding), raised in urban (group A) and rural (group B, as control) were studied.

Results. Mean values of CA/cell were 0.07±0.30 and 0.07±0.27 in groups A and B, respectively; SCE-mean values were 8.95±3.86 and 9.18±4.34, in the A and B-groups, respectively. For CBMC test, the Binucleated Cell Indexes (BCI) were 76.70±7.32 and 74.89±6.32 in the A- and B-groups, respectively. Mean values of the Bi-Nucleated cells with MN (BNMN) and MN for cell Bi-Nucleated they were 1.52±1.87 and 1.76±2.07 in the A- and B-groups, respectively. The TL values (expressed as telomere length relative to a single copy reference gene) were 1.35±0.92 and 1.44±1.21 in A and B-groups, respectively. No statistical differences were found between the two groups for each test.

Conclusions. All four test gave equivalent results and the two different environments do not originate differenced in chromosome stability in buffalo lymphocytes.

Acknowledgments

the research was supported by Project PON1_486- GENOBU

### O14 Phenotype genotype analysis in a large cohort of 85 individuals with a terminal 6q deletion

#### Aafke Engwerda^1^, Nadia Simoes De Souza^1^, Pauline Bouman^2^, Boudien Flapper^3^, Nicole Corsten-Janssen^1^, Morris Swertz^1^, Mirjam Plantinga^1^, Trijnie Dijkhuizen^1^, Conny Van Ravenswaaij-Arts^1^

##### ^1^University of Groningen, University Medical Centre Groningen, Genetics, Groningen-The Netherlands; ^2^Chromosome 6 Facebook Group, Facebook Contact Parent, Utrecht-The Netherlands; ^3^University of Groningen, University Medical Centre Groningen, Paediatrics, Groningen-The Netherlands

###### **Correspondence:** Aafke Engwerda (a.engwerda@umcg.nl)

Chromosome 6 aberrations are rare and parents of children with such an aberration often search the internet and unite in international social media platforms. Here we present our data on individuals with a terminal 6q deletion collected in a successful collaboration with the Chromosome 6 Facebook group.

Families signed up for the study via the secured project’s website by uploading the proband’s array report. Phenotype data was collected directly from the individuals or parents via an online multilingual questionnaire. Literature case reports were added to the database using the same questionnaire. We collected data on 30 and 55 individuals with a terminal 6q deletion via our website and literature, respectively.

The terminal 6q26q27 region encompasses thirty-nine protein coding genes of which seven are predicted to exhibit a haploinsufficiency effect (MAP3K7, PARK2, QKI, PDE10A, MLLT4, DLL1, TBP). We analysed the clinical data of the total group of individuals and of the seven subgroups based on the number of deleted haploinsufficiency genes.

The overall phenotype includes microcephaly, hypotonia, hypermobility of the joints, balance problems, vision problems, strabismus, feeding problems, sleeping problems, developmental delay, seizures and brain abnormalities, including cerebellum abnormalities, corpus callosum abnormalities and ventriculomegaly. In one third of the individuals short stature, respiratory problems, cardiac and kidney abnormalities were seen.

Subgroup analysis showed that umbilical hernia, spina bifida and hearing impairment were only seen in individuals with a deletion including the PARK2 gene. Developmental delay was also more severe in children with deletions including PARK2. Anal abnormalities were only seen in individuals with deletions including the QKI gene.

Social media helps in collecting large numbers of detailed genotype-phenotype data on rare chromosome aberrations, enabling a more precise description of the phenotypic spectrum.

### O15 Characterisation of complex genomic structure and variation by high resolution fibre FISH an overview

#### Fengtang Yang, Sandra Louzada, Beiyuan Fu, Ruby Banerjee

##### Wellcome Sanger Institute, Molecular Cytogenetics Core Facility, Cambridge-United Kingdom

###### **Correspondence:** Fengtang Yang (fy1@sanger.ac.uk)

The application of array-CGH and whole-genome sequencing has led the discovery of many genomic loci with highly variable structure, including copy number variants generated by duplications and deletions, as well as copy number neutral variants created by inversion and transposition. Nevertheless, characterisation of complex structural variation (SV), which is enriched with tandem duplications of low copy repeats and multi-allelic gene families, still represents a challenge to current high-throughput genomic methologies such as array-CGH, quantitative-PCR and next-generation sequencing. Additionally, many gaps in the reference genomes also contain poorly characterised gene families and/or duplicated segments. Fluorescence in-situ hybridisation (FISH), in particular fibre-FISH, remains the current method of choice for characterising such complex genomic structure and variation, since it enables direct visualisation of complex SVs and determination of SV haplotypes in a single hybridisation. In the past decade, our group have been involved in the validation and characterisation of a variety of complex genomic structures and SVs in human, mouse, zebrafish and pig by multi-colour fibre-FISH, using single-molecule DNA fibres generated by Molecular Combing and extended chromatin and DNA fibres prepared by alkaline lysis. Here, I will review the recent advance in fibre-FISH technology and contribution of fibre-FISH to the further improvement of reference genome assemblies of vertebrates, to the determination of SV haplotypes of polymorphic SVs and to the validation of target genome editing using CRISPR/Cas9. I will demonstrate that by the judicial use of probes generated by long-range PCR and sequenced genomic clones (BACs and fosmids), both simple and complex genomic rearrangements as well as SVs can be resolved using fibre-FISH, and that fibre-FISH remains one of the most accurate methodologies for typing multi-allelic SVs.

### O16 What should laboratory specialists in clinical genetics know about chromosomes ten years from now

#### Ron Hochstenbach^1^, Ellen Van Binsbergen^2^, Heleen Schuring-Blom^2^, Arjan Buijs^2^, Hans Kristian Ploos Van Amstel^2^

##### ^1^Amsterdam Umc, Department of Clinical Genetics, Amsterdam-The Netherlands; ^2^University Medical Centre Utrecht, Department of Genetics, Utrecht-The Netherlands

###### **Correspondence:** Ron Hochstenbach (p.hochstenbach@vumc.nl)

Whole Genome Sequencing (WGS) detects pathogenic gene variants, copy number variants, structural chromosome rearrangements and uniparental disomy in a single diagnostic test. Due to decreasing costs and high diagnostic yields, WGS is destined to become a first-tier test, replacing both arrays and light microscopy. Here, we discuss the status which clinical cytogenetics will have in a world dominated by WGS, and in particular, the minimal knowledge that laboratory specialists of the future should have about clinical cytogenetics. First, laboratory specialists should be aware of the types and frequencies of pathogenic genome variants that remain undetected by WGS. A survey of all 14,957 referrals for postnatal karyotyping during a 10-year period in a single center (Utrecht, The Netherlands) shows that about 8% of clinically relevant, abnormal results would be missed by WGS based on Illumina sequencing by synthesis chemistry at 30x mean genome coverage, the current industry standard. These include, for example, balanced chromosomal rearrangements with breaks in repetitive DNA sequences, as seen in couples with recurrent abortion, and low level sex chromosome mosaicism in referrals for short stature, premature ovarian failure and male infertility. Also, rare abnormalities such as r(20) epilepsy syndrome would not be detected. This spectrum of missed abnormalities may change as sequencing technology develops, but future laboratory specialists must also be able to recognize any requirement for follow-up studies by light microscopy when analyzing WGS data. Laboratory directors and boards of professional organizations will face the challenge of assuring competence in clinical cytogenetics in a time when the numbers of referrals for light microscopy are declining and laboratory specialists with a “classical” training and adequate experience in clinical cytogenetics will be harder to find.

### O17 A silicon-based coral-like nanostructured microfluidics to isolate rare cells in human circulation: validation by SK-BR-3 cancer cell line and its utility in circulating fetal nucleated red blood cells

#### Chung-Er HUANG^1^, Ming CHEN^2^

##### ^1^National Chiao-Tung University International Semiconductor College Changhua-Taiwan; ^2^Changhua Christian Hospital Dept. Genomic Medicine Changhua-Taiwan

###### **Correspondence:** Ming CHEN (mingchenmd@gmail.com)

Circulating fetal cells (CFC) are of immense importance in the research field of non-invasive prenatal diagnosis. We developed and had reported a chip-based microfluidic platform to capture the extremely rare cell population in human circulation. Recently we have optimized our platform in the following aspects: a production-ready silicon-based microfluidic microchip (PicoBioChip, we rename it as “Coral Chip”), a compact, fully automatic and easy-to-use machine for cell capture, a user-friendly, patent-protected graphical user interface (GUI) bundled software with machine learning capability, coupled with an automatic cell picker. The cells recovered can be subjected to subsequent cytogenetic and molecular genomic analyses. Here we report the validation results of the capture efficiency of our system by the SK-BR-3 cell line study (n=4), as well as the validation of the system to be used in non-invasive prenatal diagnosis (NIPD), in which the captured cells need to be verified were from fetal origin, in our selected prenatal cases with undisputable non-maternal genomic markers (N=14). The capture rates of our immunoaffinity-based positive enrichment "Cell Reveal" system coupled with silicon-based coral-like nanostructure "Coral Chip", and an automatic cell identification/retrieval system, are all exceeding 80% (80.24~94.56%). The SK-BR-3 cells being captured were successfully verified with ACGH and NGS. The captured cells, ranged from 2~71/2ml maternal blood, in all prenatal cases (n=14, GA 12+6~27+5 weeks) were verified to be fetal origin by demonstrating their non-maternal markers with SRY genotyping, FISH or ACGH.Our result demonstrated the nucleated cells we captured are indeed fetal origin, namely, the fnRBC. A prospective, large-scale, randomized study is needed to further prove the applicability of our system in clinical utility of cell-based non-invasive prenatal diagnosis

### O18 Six years of molecular cytogenetic in prenatal diagnosis benefits lessons and perspectives. A new approach according to ultrasound abnormalities observation

#### Myriam Rachid^1^, Jonathan Rosenblatt^2^, Jonathan Levy^1^, Aurélie Marc^1^, Marie-Adeline Thaly^1^, Cecile Le Long^1^, Sandra Bhattacharjee^1^, Claire Brousse^2^, Claire Loraschi^2^, Yvon Chitrit^2^, Jean-François Oury^2^, Brigitte Benzacken^3^, Eva Pipiras^3^, Severine Drunat^4^, Helene Dessuant^5^, Pascale Kleinfinger^6^, Stephane Serero^7^, Laurence Perrin^8^, Yline Capri^8^, Felicia Joinau^9^, Nadia Belarbi^10^, Marianne Alison^10^, Walid Abdennadher^11^, Elsa Seguro^12^, Fabien Guimiot^13^, Michel Benchimol^14^, Joseph Bouyou^15^, Morgane Valentin^16^, Catherine Guerin^17^, Olivier Sibony^2^, Anne-Claude Tabet^18^, Celine Dupont^18^

##### ^1^Robert Debre Hospital Ap-hp, Cytogenetic, Paris-France; ^2^Robert Debre Hospital Ap-hp, Obstetrics, Paris-France; ^3^Jean Verdier Hospital Ap-hp, Cytogenetics, Bondy-France; ^4^Robert Debre Hospital Ap-hp, Molecular Genetics, Paris-France; ^5^Biomnis Laboratory, Cytogenetics, Paris-France; ^6^Cerba Laboratory, Cytogenetics, Saint Ouen L-France; ^7^Cbcm Laboratory, Cytogenetics, Evreux-France; ^8^Robert Debre Hospital Ap-hp, Clinical Genetics, Paris-France; ^9^Delafontaine Hospital, Obstetrics, Saint Denis-France; ^10^Robert Debre Hospital Ap-hp, Radiology, Paris-France; ^11^Gonesse Hospital, Obstetrics, Gonesse-France; ^12^Beaujon Hospital Ap-hp, Obstetrics, Clichy-France; ^13^Robert Debre Hospital Ap-hp, Foetopahology, Paris-France; ^14^Jean Verdier Hospital Ap-hp, Obstetrics, Bondy-France; ^15^Lariboisière Hospital Ap-hp, Obstetrics, Paris-France; ^16^Robert Bichat Hospital Ap-hp, Obstetrics, Paris-France; ^17^Meaux Hospital, Obstetrics, Meaux-France; ^18^Robert Debre Hospital Ap-hp, Cytogenetics, Paris-France

###### **Correspondence:** Celine Dupont (c.dupont@aphp.fr)

Thanks to molecular technics now used in prenatal diagnosis, the resolution of cytogenetic’s analysis and the diagnostic yield have been improved. Our study aim to determine the yield of submicroscopic chromosomal anomalies identified in a large cohort of fetuses in a unique prenatal center at Robert Debre Hospital. We analyzed the nature of identified variants that allowed us to suggest an adapted support for at risk pregnancies according to fetal abnormality detected on prenatal ultrasound.

We retrospectively analyzed 4056 results of prenatal BoBs and 494 results of chromosomal microarray (CMA) for patients referred for prenatal diagnosis between 2011 and 2017. BoBs identified an abnormal result in 13,6% of cases, mainly trisomy 21 and 18. Microarray identified CNV in 13,4% of cases, among which 5.1% of cryptic CNVs. Fetal indications were classified into 19 groups. BoBs and microarray results were first analyzed regarding the nature of the genomic variant and then regarding the fetal anomalies in each group. We observed, as expected, that the diagnostic yield and the nature of chromosomal anomalies depend on the indication that motivated prenatal diagnosis. Results by indication allowed us to propose an adapted chromosomal diagnosis and genetic counseling according to ultrasound fetal abnormality. Moreover, we confirmed many known associations and reported some new ones such as IUGR associated with Williams’s syndrome and high nuchal translucency with 1p36 microdeletion. We also notified that most CAKUT are exceptionally associated with aneuploidy. So we proposed that these last types of malformations should benefit from first tier CMA without rapid aneuploidy assay (FISH or BOBs).

At the beginning of the era of pangenomic molecular fetal investigations, our study gives crucial informations about the chromosomal classification of sonographic markers.

### O19 Balanced X autosome translocations and premature ovarian failure are associated with altered expression of growth factors junction organization and immune pathways

#### Adriana Di-Battista^1^, Mariana Moyses-Oliveira^2^, Malu Zamariolli^1^, Maria Isabel Melaragno^1^, Alexandre Reymond^3^

##### ^1^Federal University of São Paulo, Morphology and Genetics, São Paulo-Brazil; ^2^Broad Institute of Mit And Harvard, Center for Genomic Medicine, Boston-United States; ^3^University of Lausanne, Center for Integrative Genomics, Lausanne-Switzerland

###### **Correspondence:** Adriana Di-Battista (dribattista@gmail.com)

Patients with balanced X-autosome translocations and premature ovarian failure (POF) are an interesting paradigm to study the positioning effect of chromosome segments. They present breakpoints that do not disrupt genes related to the phenotype and map within cytobands Xq13-Xq21, from which 80% cluster in Xq21. As deletions within Xq21 do not cause POF, and since different breakpoints and translocation with different autosomes lead to the same phenotype, we hypothesized a “position effect” as possible mechanism. We fine-mapped the breakpoints in six patients with POF and balanced Xq-autosome translocations, established lymphoblastoid cell lines from patients and matched female controls and profiled their transcriptome through RNA-seq. The 68 genes differentially expressed in the patients’ group are enriched for genes encoding important proteins for the organization of cell junctions and the immune response. Previous transcriptome studies pinpointed that cellular assembly and maintenance are among the most expressed pathways in ovarian cells. Additionally, GWAS showed that immune pathway-related genes were associated with age at normal menopause, allowing to postulate that genes involved in this regulation could also be involved in POF. As the perturbed transcripts are neither mapping to the X chromosome, nor to the autosome breakpoint, this might indicate that the effect is indirect, and the phenotype is triggered by perturbations of normal contacts between genes and their regulatory elements. To further challenge this model, we are currently assessing the chromatin accessibility of the same cell lines. These results should help elucidating the impact of derivative chromosomes repositioning within interphase nuclei.

Financial support

FAPESP#2017/20847-9.

## Poster Abstracts

### 1. Clinical Cytogenomics

#### 1.P1 Exposure and duration of lithium treatment affect leukocyte telomere length in bipolar disorder patients

##### Alessio Squassina^1^, Claudia Pisanu^1^, Donatella Congiu^1^, Carla Melis^1^, Eleni Merkouri Papadima^1^, Paola Caria^1^, Tinuccia Dettori^1^, Giovanni Severino^1^, Alberto Bocchetta^2^, Caterina Chillotti^2^, Maria Del Zompo^2^, Roberta Vanni^1^

###### ^1^University of Cagliari, Biomedical Sciences, Monserrato-Italy; ^2^University Hospital of Cagliari, Unit of Clinical Pharmacology, Cagliari-Italy

####### **Correspondence:** Tinuccia Dettori (dettorit@unica.it)

Bipolar disorder (BD) is associated with premature mortality and higher incidence of age-related disorders compared to the general populations. Several studies reported premature cell senescence in BD, by reduced telomere length in affected subjects and have also shown that antidepressants and lithium (Li) may have a protective effect against telomere shortening. In our previous work (Squassina et al., 2016) we showed that duration of Li treatment was positively correlated with leukocyte telomere length (LTL) in BD patients (more than 24 months). In this study, we compared LTL between 313 BD patients and 316 healthy controls and tested if Li treatment may affect LTL in BD in a larger sample.

Correlation between LTL and age at onset, number of manic and depressive episodes, years of illness before start of Li treatment and duration of Li treatment was assessed by real-time PCR.

LTL correlated negatively with age (P < 0.001) and was independent of sex (p > 0.05). Positive correlation between LTL and Li treatment in patients with at least 24 months of Li treatment (p = 0.03), while there was no effect of the other variables tested. BD patients Li-treatment had longer LTL compared to healthy controls (p=0.00002) and compared to patients never exposed (p=1.5x10-8). Never exposed group showed reduced LTL compared to controls ,not statistically significant (p>0.05).

Our data show: that BD patients with Li treatment have longer LTL compared to BD subjects never exposed to Li and to controls also in the extended sample, and support previous findings showing that long-term Li treatment has a protective effect against telomere shortening in BD. Further analyses are ongoing to validate our findings.

Funding

Fondazione di Sardegna and Regione Autonoma Sardegna.

#### 1.P2 Investigation of copy number variation and genomic imprinting alteration in 49 Prader Willi like patients by means of MLPA

##### Ingrid Camila Possa Paranhos^1^, Catielly Ferreira Rocha^2^, Lívia Teixeira Leite^3^, Sônia Regina Middleton^3^, Suely Rodrigues Dos Santos^2^, Carmen Lucia Antão Paiva^1^

###### ^1^Universidade Federal Do Estado Do Rio De Janeiro, Programa De Pós-Graduação Em Biologia Molecular E Celular, Rio De Janeiro-Brazil; ^2^Universidade Federal Do Estado Do Rio De Janeiro, Programa De Pós-Graduação Em Neurologia, Rio De Janeiro-Brazil; ^3^Universidade Federal Do Estado Do Rio De Janeiro, Departamento De Genética E Biologia Molecular, Rio De Janeiro-Brazil

####### **Correspondence:** Carmen Lucia Antão Paiva (clapaiva1@gmail.com)

Prader-Willi Syndrome (PWS), the most frequent type of syndromic obesity, is a complex multi-organ disease caused by the lack of expression of paternal genes in 15q11-q13. Prader-Willi-like phenotype (PWL) is a condition which the patient presents, along with obesity, other clinical features such as developmental delay and/or other conditions compatible with PWS.

The objective of this study was to further investigate, with MLPA technique, 49 patients suspected of PWS who were negative for alterations on 15q11-q13 investigated with the methylation-specific PCR testing. The following MLPA kits (MRC Holland, Amsterdam, Holland) were used to identify changes in copy number and genomic imprinting: P224-PPARG, ME028-PWS, P064-Mental Retardation, P036–Subtelomeric region, ME032-DUP 14, P220–Obesity, P070–Subtelomeric and ME031–GNAS. Out of these 49 PW-like patients, 11 of them had positive MLPA results such as:1) deletion of the 1p31.3 region; 2) duplication of exon 6 of PPARG; 3) deletion of SNRPN-u1b; 4) duplication of 4p16.3 and deletion 9p24.3; 5) duplication of 22q11.21; 6) deletion of 1p36; 7) deletion of 2q37.3; 8) hypomethylation pattern of segment 14q32.2, 9) deletion of 16p11.2, 10) deletion of GNAS exons 1A and 1; and 11) deletion of GNAS exon 7. It is important to mention that, as far as we know, these are the first reported cases of a duplication on PPARG exon 6, deletion on GNAS exon 1A and deletion of SNRPN-u1b, all leading to PWL. Array-CGH is going to be used to confirm these alterations of SNRPN-u1b, GNAS and PPARG. The other results reinforce the contribution of the mentioned cytogenetic and epigenetic alterations to obesity, energy balance and other PW-like characteristics.

#### 1.P3 A case report of 1p36 deletion syndrome

##### Valentyna Kurakova, Sholpan Kulbalaieva, Maryna Tcygankova, Vira Galagan

###### National Children Hospital, Medical Genetic Center, Kyiv-Ukraine

####### **Correspondence:** Valentyna Kurakova (kurakova.vv@gmail.com)

Introduction. Statistically, every year in Ukraine about 5.5-6.0 thousand children are born with congenital heart defects, 35-40% of which are in critical condition from the first days of life. They need emergency intensive care, surgical correction and adequate diagnosis of syndromic pathology.

Materials and methods. Clinical, standard karyotyping, FISH-analysis using the LSI p58 (1p36) / TelVysion 1p / LSI 1q25 locus-specific probe, Vysis.

Discussion. The proband, a 10-month-old girl, was referred to the center of medical genetic from the center of pediatric cardiology and cardiac surgery. She has congenital heart disease: multiple ventricular septal defects, congenital pulmonary valve stenosis, open oval window.

Anamnesis: Parents are 26 years; child from pregnancy II, II birth. Prenatal ultrasound screening was performed 3 times: 11-12 weeks - multiple ventricular septum defects in the fetus; 18 weeks - cleft lip, hydrocephaly and the central nervous system developmental malformation; on 22 weeks – tetralogy of Fallot.

Genetic counseling: the child does not hold her head, does not sit, no convulsions, normal height, weight - second degree of malnutrition. Phenotype: microcephaly, prominent forehead, sunken nose, antimongoloid shape of eyes, epicant, 2-sided complete cleft lip and palate, deformed ears, wide set nipples, violation of the finger row on the feet, broad great toe, muscular hypotonia, developmental delay.

Standard chromosomal analysis suggested a terminal deletion of the short arm of chromosome 1. FISH-analysis showed the absence of a 1p36 locus signal on one of the homologous chromosomes. Result: 46,XX,?del(1)(p36).ish del(1)(p36)(p58-).

Conclusion: 1p36 syndrome was confirmed for the first time in 10 years of clinical observations and is an important precursor for understanding this pathology.

Written, informed consent for publication was obtained from the patient [or parent/guardian for patients under 16]

#### 1.P4 Novel case with a double “apparently” balanced rearrangement disrupting EXT1 in a patient with hereditary multiple exostoses

##### Angelos Alexandrou^1^, Nicole Salameh^1^, Ioannis Papaevripidou^1^, Nayia Nicolaou^2^, Panayiotis Myrianthopoulos^1^, Andria Ketoni^1^, Paola Evangelidou^1^, George A. Tanteles^2^, Carolina Sismani^1^

###### ^1^The Cyprus Institute of Neurology and Genetics, Cytogenetics and Genomics, Nicosia-Cyprus; ^2^The Cyprus Institute of Neurology and Genetics, Clinical Genetics Department, Nicosia-Cyprus

####### **Correspondence:** Paola Evangelidou (paola@cing.ac.cy)

Hereditary multiple exostoses (HME) is an autosomal dominant skeletal disorder characterized by the development of multiple, circumscript, occasionally painful and usually symmetric bony protuberances called osteochondromas. HME is caused by EXT1 and EXT2 loss of function mutations. Most pathogenic mutations are nonsense followed by missense mutations and deletions. We report on a patient with a rare and complex genotype resulting in a classical HME phenotype.

Mutations in EXT1 and EXT2 were excluded by Sanger sequencing. The patient was subsequently referred for karyotype and array-CGH analyses. Results obtained were validated with FISH and qRT-PCR and parental studies determined the mode of inheritance.

Chromosomal analysis revealed a de novo “apparently” balanced double rearrangement: a balanced translocation between chromosomes 2 and 3 at breakpoints 2q22 and 3q13.2 and a pericentric 8p23.1q24.1 inversion, both of which were confirmed by FISH analysis. Subsequently, array-CGH analysis revealed a novel heterozygous deletion within the EXT1 gene at the inversion breakpoints, rendering the inversion as unbalanced. The inheritance mode as well as the size of the deletion was further investigated by qRT-PCR and the deletion was characterized as a de novo 3.1kb deletion removing exon 10. The inversion in combination with the 8p23.1 deletion most likely abolishes the transcription of EXT1 downstream of exon 10 hence resulting in a truncated protein.

To conclude, a rare and novel pathogenic cause of HME is presented in this study, highlighting the importance of additional comprehensive cytogenetic investigation when EXT1 and EXT2 mutation analysis is negative.

#### 1.P5 High occurrence of chromosomal rearrangements among genetic causes of congenital aniridia in Russia

##### Rena Zinchenko^1^, Tatyana Vasilyeva^2^, Vitaly Kadyshev^2^, Andrey Marakhonov^3^

###### ^1^Research Centre for Medical Genetics and Pirogov Russian National Research Medical University, Laboratory of Genetic Epidemiology, Moscow-Russia; ^2^Research Centre for Medical Genetics, Laboratory of Genetic Epidemiology, Moscow-Russia; ^3^Research Centre for Medical Genetics and Far Eastern Federal University, Laboratory Of Genetic Epidemiology, Moscow and Vladivostok-Russia

####### **Correspondence:** Vitaly Kadyshev (renazinchenko@mail.ru)

11p13 deletions are associated either with isolated congenital aniridia (OMIM #106210) (AN) or with WAGR syndrome (OMIM #194072) where aniridia is one of pathognomonic signs. If the deletion encompasses the WT1 gene Wilms tumor develops (in ~70% cases). Early nephroblastoma development poses the urgency of DNA diagnostics in all patients with clinical signs of congenital aniridia.

Materials and methods.

DNA diagnostics was performed in 195 patients from 163 unrelated families from Russia: 184 patients from 152 unrelated families with AN and 11 patients from 11 families with WAGR. Screening for mutations was carried out by Sanger sequencing, MLPA, and FISH analysis if the WT1 gene was affected.

Results.

A total of 149 different mutations: 107 small PAX6 intragenic variants and 42 large deletions were found in patients with AN. In all WAGR patients deletions encompassing the PAX6 and WT1 genes were defined. 3 patients showed neither small PAX6 mutations nor microdeletions of 11p13.

The proportion of chromosome rearrangements was about one third of all genetic causes of AN (42/149). The localization of chromosome breakpoints and the length of the deleted regions varied (0.1÷7.5Mb). The same deletion affecting PAX6 downstream regulatory regions (0.5÷1.5Mb) occurred especially frequent (17/42). One deletion (1/42) was found to be the result of pericentric inversion of chromosome 11 inv(11)(p13q14).

Conclusion.

Analysis of chromosomal imbalance of the 11p13 region is an important step of the DNA diagnostics in patients with AN in Russia, not only due to the importance of early detection of WAGR deletions, but also by the high frequency of large chromosome rearrangements among genetic causes of AN in the Russian population.

The study was supported by grant RSF №17-15-01051 and state task of the Ministry of education.

#### 1.P6 Susceptibility loci CNVs with incomplete penetrance accurate diagnosis with uncertain prognosis

##### Silvia Serafim^1^, Barbara Marques^2^, Hildeberto Correia^2^

###### ^1^Instituto Nacional De Saude Dr. Ricardo Jorge, Departamento De Genetica Humana - Unidade De Citogenetica, Lisboa-Portugal; ^2^Instituto Nacional De Saude Dr. Ricardo Jorge, Departamento De Genetica Humana - Unidade De Citogenetic, Lisboa-Portugal

####### **Correspondence:** Silvia Serafim (silvia.serafim@insa.min-saude.pt)

Chromosomal microarray analysis (CMA) is the first-tier test for developmental delay, autism spectrum disorders, and congenital abnormalities in postnatal diagnosis and for ultrasound abnormalities in prenatal diagnosis.

The detection of variants with clinical significance by CMA, when compared to karyotype, can increase up to 10-20% in postnatal diagnosis and up to 5-18% in prenatal diagnosis. Nevertheless CMA also detects incomplete penetrance neuro-Susceptibility Loci Copy Number Variants (SL-CNV), which although having clinical significance have an uncertain prognosis.

The aim of this study is to identify from the literature a set of SL-CNV, and the corresponding penetrance for each variant, determining their occurrence in our cohort of postnatal samples ran between January 2012 and August 2018 and prenatal samples ran between January 2015 and August 2018.

We have established a 21 SL-CNV set, and from a total of 835 postnatal samples and 317 prenatal samples we have identified 36 and 11 cases, respectively, with a variant in one of the 21 established SL-CNV.

The percentage of cases with a SL-CNV is relatively similar between postnatal samples (4.5%) and prenatal samples (3.5%), although the reason of referral for the two groups is not completely overlapping and also the total number of prenatal samples represents about half of the time span of the postnatal samples, which might have underestimated their occurrence. The estimated penetrance for each of the established SL-CNV present some inter-publication variability, especially concerning samples with different phenotypes. Nevertheless some variants show concordance.

Estimating the penetrance for SL-CNV, and their clinical impact for the patient or carriers in the family, is a complex task. Only time, analysis of larger cohorts, and future knowledge of genotype-environment-phenotype interactions will overcome this difficulty, decreasing uncertainty for the around 4% of patients diagnosed by CMA.

#### 1.P7 19q13.32 microdeletion syndrome further delineation of the clinical phenotype

##### Laura Van Zutven^1^, Conny Van Ravenswaaij-Arts^2^, Trijnie Dijkhuizen^2^, Magda Mcgregor-Schuerman^3^, Yolande Van Bever^1^, Malgorzata Srebniak^1^

###### ^1^Erasmus Mc, Clinical Genetics, Rotterdam-The Netherlands; ^2^Umcg, Clinical Genetics, Groningen-The Netherlands; ^3^Rkz St. Vincentius, Pediatrics, Paramaribo-Suriname

####### **Correspondence:** Laura Van Zutven (l.vanzutven@erasmusmc.nl)

Background

Interstitial 19q13.32 microdeletions are rare and have been reported in only five patients so far. Common features mentioned in the literature include intellectual disability/developmental delay, facial asymmetry, ptosis, oculomotor paralysis, orofacial clefting, micrognathia, kyphoscoliosis, cardiac abnormalities and constipation,. Since only a few patients have been reported, little is known about the phenotypic spectrum of these deletions.

Methods

To expand the knowledge on interstitial 19q13.32 microdeletions, we report two new patients with a de novo microdeletion in this region, and compare them with previously published patients.

Results

Evaluation of the phenotypic features in the five published and the two new patients showed that only developmental delay/intellectual disability was present in all patients. No cardiac abnormalities, facial asymmetry, ptosis or micrognathia were present in our patients. Patient 1, with a 1 Mb deletion 19q13.32q13.33, did not have oculomotor dysfunction, scoliosis or constipation, and patient 2, carrying a 3.2 Mb deletion 19q13.31q13.33, did not have a cleft palate. New features noted in patients with 19q13.32 microdeletions were ear abnormalities, including hearing loss, down slanting palpebral fissures and, in male patients, hypospadias.

Conclusions

Major clinical features seem to differ between the patients with 19q13.32 microdeletions reported so far. In addition, some of the previously presented key features for this microdeletion are absent in our patients. As a consequence, the syndrome may not be as recognizable as previously suggested. Identification of other patients is necessary to further delineate the clinical phenotype of the 19q13.32 microdeletion and to establish the critical region/genes responsible for intellectual disability in these patients.

#### 1.P8 X chromosome mosaicism in women experiencing fertility problems

##### Elisavet Kouvidi^1^, Sophia Zachaki^1^, Haroula Tsarouha^1^, Amelia Pantou^2^, Emmanouel Kanavakis^2^, Ariadni Mavrou^1^

###### ^1^Genesis Genoma Lab, Genetic Diagnosis, Clinical Genetics & Research, Cytogenetics, Athens-Greece; ^2^Genesis Genoma Lab, Genetic Diagnosis, Clinical Genetics & Research, Molecular Genetics, Athens-Greece

####### **Correspondence:** Elisavet Kouvidi (ekouvidi@hotmail.com)

AIM

To determine the incidence of X chromosome mosaicism in women planning to enter an in vitro fertilization (IVF) cycle.

PARTICIPANTS & METHODS

1,059 women (average age: 39 years) were included in the study. Peripheral blood karyotyping was performed by conventional cytogenetic techniques. 25 G-banded metaphases were studied from each case and if mosaicism was suspected, the analysis was extended to 100 metaphases. X chromosome mosaicism <4% and chromosomal polymorphisms were not reported. Written informed consent was obtained from all women. Statistical analysis was performed using Pearson Chi-square και Fisher’s exact tests with 5% significance level.

RESULTS

892 women (84.2%) had a normal karyotype (46,XX), while in 167 (15.8%) various chromosomal abnormalities were detected. Twelve women (1.1%) carried autosomal chromosome abnormalities and 155 (14.7%) had sex chromosome X aneuploidies in a mosaic form. Specifically, 72 (46.5%) exhibited low level mosaicism (4-6%), while in 83 women (53.5%) mosaicism was >6%. Women were categorized according to age into 3 groups; 23-34 years old (Ν=278), 35-45 (Ν=705) and >45 years old (Ν=76). The incidence of mosaicism was significantly higher in women >35 years of age, as compared to younger ones (18.4% vs 4.0%, p<0.001), a finding consistent with loss of chromosome X with ageing. Among women with X chromosome aneuploidy, 57/155(36.8%) had 2 cell lines (54 were 45,Χ/46,ΧΧ and 3 were 47,ΧΧΧ/46,ΧΧ), 94/155(60.7%) had 3 cell lines (45,Χ/47,ΧΧΧ/46,ΧΧ) and 4/155(2.5%) had 4 cell lines (45,Χ/47,ΧΧΧ/48,ΧΧΧΧ/46,ΧΧ). Two cell lines were observed more frequently in women <35 years old, as compared to older women (63.6% vs 34.7%, respectively, p=0.055).

CONCLUSIONS

Although X-chromosome mosaicism was commonly observed in women with infertility in our series, without comparison with age matched controls (fertile women) we are unable to say whether it was the cause of infertility.

#### 1.P9 Identification of Copy Number Variation by array CGH in Portuguese Children Diagnosed with Autism Spectrum Disorders

##### Sidonie Monteiro^1^, Joel Pinto^2^, Miguel Leão^3^, Sofia Dória^2^

###### ^1^Faculty of Medicine, University of Porto, Genetics Department, Porto-Portugal; ^2^Faculty of Medicine And Instituto De Investigação E Inovação Em Saúde, University Of Porto, Genetics Department, Porto-Portugal; ^3^Centro Hospitalar De São João, Neurogenetic Unit, Departement of Medical Genetics, Porto-Portugal

####### **Correspondence:** Sofia Dória (sdoria@med.up.pt)

Background: Autism spectrum disorders (ASD) are a heterogeneous group of neurodevelopmental diseases. ASD affect many children and usually manifest themselves in the earlier stages of life. Array CGH offers superior sensitivity for the identification of submicroscopic chromosomal abnormalities and it is considered to be the first-tier genetic testing technique for patients with ASD. Copy Number Variants (CNVs), which are known to predispose children to these neurodevelopmental disorders, may play a role in the etiology of ASD. The main objective of this study was to establish a clinical association between array-CGH results and ASD.

Methods: 253 patients admitted to a neurogenetic consultation and diagnosed with ASD were selected for array-CGH (Agilent 4x180K microarrays). CNVs were classified as benign, pathogenic, likely pathogenic, VOUS and pathogenic in recessive forms .

Results: 3,557% (9/253) of CNVs were classified as pathogenic CNVs. When likely pathogenic CNVs were considered the rate increased to 11,462% (29/253). This is similar to the frequencies found in the literature (around 10%). Some unexpected CNVs not always correlated to the ASD pathophysiology were also found. Taking into account a phenotype-genotype correlation the patients were divided in two groups. In the group with this correlation, we found 22 pathogenic or likely pathogenic CNVs (10 deletions and 12 duplications). Within this group we were able to perform parent studies in 6 cases, 5 inherited and 1 de novo.

Conclusion: The identification of copy number variations in children and adolescents with autistic disorders highlight the relevance of array comparative genomic hybridization as the first-tier genetic test.

Next sequencing generation (NGS), with specific ASD panels for the most common mutations, has widely been used to assess patients. We nonetheless underline the need for better data in NGS for reducing the uncertainty of the results.

#### 1.P10 Screening for mutations in the SHOX gene (SHOX) in patients with short stature

##### Pavlina Capkova^1^, Zuzana Capkova^1^, Peter Rohon^1^, Katerina Adamova^1^, Jirina Zapletalova^2^

###### ^1^University Hospital Olomouc (fnol), Department Of Medical Genetics, Olomouc-Czech Republic; ^2^Faculty of Medicine And Dentistry, Palacky University Olomouc, Children’s Clinic, Olomouc-Czech Republic

####### **Correspondence:** Pavlina Capkova (pcapkova@seznam.cz)

SHOX mutations have been described as a cause of Léri-Weill dyschondrosteosis (LWD), Langer Mesomelic Dysplasia (LMD) in approximately 75 % of the patients, and as a cause of Idiopathic Short Stature (ISS) in 6–15 % of the patients. Chromosomal aberrations including Turner syndrome or 47,XXX are also included among mechanisms leading to the loss or gain of SHOX. We performed an extensive screening in tested (N=174) and control (N=91) groups involving karyotyping (Ntested=169), MLPA with probemix SALSA MLPA P018-G1(SHOX)MRC Holland (Ntested=174), Sanger sequencing (Ntested=154), and in the relevant cases I-FISH on buccal smear (Ntested=51) with satellite enumeration probe X,Y (Cytocell) to detect CNV/mutations involving SHOX. The aim of the study was to assess the efficacy of particular methods for the detection of variants in SHOX and to evaluate the positive predictive value (PPV) of short stature in the detection of SHOX variants.

Results: In total, 28 and 16 variants influencing the SHOX gene were detected in the tested and control cohorts, respectively (p >.01). Heterozygous loss of SHOX was revealed in all cases of patients with LWD/TS phenotype. MLPA shows the highest (13.8 %) and sequencing the lowest detection rate (1.9 %) in the short stature group. Mosaicism of X monosomy and structural aberrations of gonosomes were also detected by karyotyping and FISH–detection rates were 3.6 % and 11.8 % respectively. FISH on buccal smear discovered cryptic mosaic 45,X in two cases with normal karyotype, which might have otherwise been overlooked.

Conclusion: Short stature as the standalone indicator for the screening of SHOX mutations has low PPV (16.10 %). This implies the strong influence of other genes contributing to human height especially in the group of ISS patients.

Supported by MH CZ-DRO(FNOl,00098892).

#### 1.P11 Three cases of mosaicism with an unbalanced translocation and a normal cell line

##### Marina Minzhenkova, Zhanna Markova, Natalia Kuzina, Nadezda Shilova

###### Research Centre for Medical Genetics, Laboratory of Cytogenetics, Moscow-Russia

####### **Correspondence:** Marina Minzhenkova (maramin@mail.ru)

Mosaicism with an unbalanced autosomal translocation and a normal cell line is extremely rare event. To our knowledge just about 20 cases are reported. We present three cases of mosaicism with an unbalanced autosomal translocation and a normal cell line detected by molecular cytogenetic diagnosis. The karyotypes and FISH analyses of the parents were normal in these cases, thereby all mosaic unbalanced translocations were de novo.

Case 1. The patient 2.10 year old was referred for evaluation because of developmental delay and dysmorphic features. The karyotype of the patient was normal. Array-CGH analysis revealed a terminal deletion of the chromosome 8 (8p23.1p23.3) and a terminal duplication of the chromosome 10 (10q26.11q26.3). Unexpectedly additional FISH analysis showed an unbalanced translocation der(8)t(8;10)(p23.1;q26.11) in 77% and a normal karyotype in 23% of cells.

Case 2. A newborn was referred for karyotyping of a Сri-du-chat syndrome. Chromosome analysis revealed a mosaicism with a normal cell line and a derivative chromosome 5. mFISH indicated an unbalanced translocation t(5;9). Subtelomeric probes on chromosome 9 were applied to identify a translocation arm and consider a level of mosaicism. FISH analysis showed a mosaic karyotype with an unbalanced translocation in 88% and a normal karyotype in 12% of cells. Combined with the banding data the karyotype was determined as mos 46,XY,der(5)t(5;9)(p13.3;р22)/46,XY.

Case 3. A 2.7-year old girl was referred for evaluation because of developmental delay, dysmorphic features, autism. Karyotype showed an additional material on chromosome 14q. mFISH and subtelomeric probes analysis revealed a mosaic unbalanced translocation der(14)t(3;14)(q26;q32.3) in 85% and a normal karyotype in 15% of cells. Moreover in this case we could determine mosaicism in both blood and on buccal mucosal cells. Using FISH a level of mosaicism on buccal cells was 70% (unbalanced translocation) and 30% (normal karyotype).

Written, informed consent for publication was obtained from the patient [or parent/guardian for patients under 16]

#### 1.P12 "Two hit" model as an explanation of variable expressivity of recurrent submicroscopic chromosomal rearrangements in children with intellectual disability and developmental delay

##### Marketa Wayhelova^1^, Eva Hladilkova^2^, Vladimira Vallova^1^, Jan Smetana^1^, Hana Filkova^2^, Renata Gaillyova^2^, Petr Kuglik^1^

###### ^1^Masaryk University, Institute of Experimental Biology, Faculty of Science, Brno-Czech Republic; ^2^University Hospital Brno, Department of Medical Genetics, Brno-Czech Republic

####### **Correspondence:** Marketa Wayhelova (marketa.way@gmail.com)

Microarray-based whole genomic analyses of submicroscopic chromosomal rearrangements (CNVs) lead to the identification of pathogenic genetic variants in 15-20% children with intellectual disability, autism spectrum disorders and congenital abnormalities. Using the genotype-first approach more than 250 loci across the human genome related to pathogenic microdeletions and microduplications were identified so far. The significant phenotypic variability among patients with identical pathogenic CNVs results in complications and uncertainty in the genetic counselling for the assessment of prognosis and challenges to additional whole genomic analyses. Based on the results of complex whole genomic analyses the phenomenon of variable expressivity leading to the phenotypic variability among patients with recurrent pathogenic CNVs can be explained using the "two-hit" model.

In the 8-year period of array-CGH analyses at the Department of Medical Genetics (University Hospital Brno, Czech Republic) 724 children patients were examined using 4X180K and 8X60K microarray platforms. In our study we present clinical characteristics of 8 patients with two structurally independent submicroscopic pathogenic CNVs (dup 2q12.1-q12.3 + dup 2q13, del 4q12-q13.1 + del 9q21.1, del 2q23.1-q23.3 + dup 7q21.12-q21.13, del 10q26.2-q26.3 + dup 11p14.3-p15.1, del 10p15.1-p15.3 + del 15q13.2-q13.3, del 13q12.12 + dup 13q21.31, dup 1q21.1 + del 1q21.1-q21.2) or one submicroscopic pathogenic CNV and chromosomal aneuploidy (47,XXY + del 7q11.23). The comparison of their phenotypes to the typical phenotypes related to given pathogenic CNVs may help to specify their partial impact on the total phenotypic effect.

Supported by the project of the Faculty of Science of Masaryk University MUNI/A/0958/2018 and the Institutional Support of the University Hospital Brno 2019 - PIG 5/19.

#### 1.P13 Case report 9q34.3 Microdeletion – Kleefstra syndrome

##### Birgitta Gläser, Katalin Komlosi, Evelyn Loeser, Andreas Tzschach, Judith Fischer

###### University Medical Center Freiburg, Institute of Human Genetics, Freiburg-Germany

####### **Correspondence:** Birgitta Gläser (birgitta.glaeser@uniklinik-freiburg.de)

We report on a male patient born from the first pregnancy of non-consanguineous healthy Caucasian parents referred to counseling at the age of 5 for evaluation for developmental retardation, severe delay in expressive speech but good receptive language abilities, brachycephaly, flat face, midface hypoplasia, hypotelorism, down-slanting palpebral fissures, flat nasal root, short nose, anteverted nares, short philtrum, thin upper vermillion, orofacial hypotonia with sialorrhea, small teeth with diasthema, tapering fingers, wide toes, and a mild generalized hypotonia. Apart from valvular pulmonary artery stenosis diagnosed at 2 weeks of age and unilateral cryptorchidism no other malformations were known.

Molecular karyotyping using microarray analysis revealed a heterozygous deletion of approximately 269 kb out of the chromosomal region 9q34.3 including the Euchromatin Methyl Transferase 1 (EHMT1) gene. Deletions and mutations of the EHMT1 gene are associated with the rare genetic disorder Kleefstra syndrome (Kleefstra et al. 2006; 2009). Kleefstra syndrome is characterised by intellectual disability, often accompanied by a spectrum of complex physical and clinical features. Congenital heart defects are seen in 50% of patients. There are approximately < 150 patients described in the literature. 80% of them show a microdeletion including EHMT1 the other 20% have intragenic mutations of the EHMT1 gene. Genotype – phenotype correlation studies revealed, that patients with intragenic mutations or small microdeletion like in our patient show very similar phenotypic abnormalities and only mild mental retardation (Yatsenko et al. 2005; Kleefstra et al. 2009).

#### 1.P14 Two cases of de novo small supernumerary marker chromosome (SSMC) detected postnatally by microarray. Clinical consequences

##### Kati Kuuse, Pille Tammur, Piret Ilisson, Mare Jürgenson, Kai Muru, Karit Reinson

###### Tartu University Hospital, Department of Clinical Genetics, Tartu-Estonia

####### **Correspondence:** Kati Kuuse (kati.kuuse@kliinikum.ee)

A small supernumerary marker chromosome (SSMC) is a structurally abnormal additional chromosome, most often lacking a distinct banding pattern and is rarely identifiable by conventional banding cytogenetic analysis. The origin and composition of an SSMC is recognizable by molecular cytogenetic analysis. The effects of SSMCs on the phenotype depend on the size, genetic content, and the level of mosaicism of the marker chromosome.

We report two cases of SSMCs: 1) 3-year-old boy, referred due to developmental problems, dysmorphism and microcephaly. Chromosomal microarray analysis (CMA, HumanCytoSNP-12 BeadChip, Illumina Inc.) revealed a ~7.9 Mb mosaic duplication of 2q11.1q12.1 material. G-banding showed the presence of an SSMC in the shape of a small ring chromosome in mosaic form (70%).

2) 4-year-old girl, referred due to developmental problems, epilepsy, multicystic kidney, small stature and dysmorphism. CMA revealed both single and two-copy-gain in region 15q11.2-q13.3. (~1.4 and ~7.6 Mb respectively). G-banding showed the presence of an SSMC and FISH confirmed the presence of two SNRPN signals on the marker. This is likely a more complex rearrangement than idic(15).

Both markers are de novo.

Clinical picture and laboratory results of both cases will be presented on the poster.

Written, informed consent for publication was obtained from the patient [or parent/guardian for patients under 16]

#### 1.P15 The phenotypic variability of duplications overlapping 15q13.3 region report of 5 patients

##### Magdalena Budisteanu^1^, Ioana Streata^2^, Mihai Cucu^2^, Andrei Pirvu^2^, Simona Serban-Sosoi^2^, Sorina Mihaela Papuc^3^, Catrinel Iliescu^1^, Cristina Anghelescu^1^, Doina Ioana^1^, Aurora Arghir^3^, Mihai Ioana^2^

###### ^1^“Prof. Dr. Alex. Obregia” Clinical Hospital of Psychiatry, Pediatric Neurology, Bucharest-Romania; ^2^University of Medicine and Pharmacy;5. Regional Center of Medical Genetics Dolj, Medical Genetics, Craiova-Romania; ^3^“Victor Babes” National Institute of Pathology, Medical Genetics Laboratory, Bucharest-Romania

####### **Correspondence:** Magdalena Budisteanu (magda_efrim@yahoo.com)

15q13.3 microduplications are rare chromosomal events with a wide spectrum of clinical presentation ranging from normal to different neuropsychiatric conditions, such as developmental delay, intellectual disability (ID), epilepsy, hypotonia, autistic features, attention-deficit hyperactivity disorder, and schizophrenia. The smallest region of overlap for 15q13.3 duplications harbours the CHRNA7 gene, a strong candidate for the behavioral abnormalities. Here we report on a series of 5 children carrying 15q13.3 duplications.

Material and methods: four boys and one girl were investigated by array CGH. In all patients the personal history, psychomotor development and behavior, presence of dysmorphic features, neuroimaging and EEG anomalies were noted.

Results: The size of the duplications ranged from 9.65 Mb to 0.38 Mb, and included the CHRNA7 gene in all patients. The most common clinical features present in all patients were speech delay and muscle hypotonia; autistic behavior was noted in 4 children, and ID was present in 3 patients. Four patients had feeding problems; one child presented epileptic seizures, but EEG anomalies were observed in 3 children. Regarding the dysmorphic features, we noted facial dysmorphic features in 3 patients only, including malformed protruding ears, up-slanting palpebral fissures, micrognathia; however, a specific facial gestalt could not be described. Neuroimaging studies showed anomalies in 2 children: Dandy-Walker malformation and small bilateral periventricular cysts, respectively. In our cases the duplication size does not correlate with the severity of clinical features.

Conclusions: 15q13.3 duplications are associated with different neuropsychiatric features, including speech delay, hypotonia, autism, and ID, also present in our patient group. Our results further support the association of 15q13.3 duplications with neuropsychiatric phenotypes, with a clinical variability, which is yet to be explained.

Acknowledgement

ERA-NET NEURON SYNSCHIZ project 6/2018; Projects of Excellence Funding in RDI, Contract No. 7PFE/16.10.2018

#### 1.P16 Gonosomal findings in three different germ layers in patients with Turner syndrome phenotype. Haplotyping of X chromosome

##### Katerina Adamova^1^, Dita Vrbicka^1^, Vrtel Radek^1^, Petr Vrtěl^1^, Martin Prochazka^1^, Eva Klaskova^2^

###### ^1^University Hospital And Palacky University Olomouc, Department Of Medical Genetics, Olomouc-Czech Republic; ^2^University Hospital and Palacky University Olomouc, Department of Paediatrics, Olomouc-Czech Republic

####### **Correspondence:** Katerina Adamova (dita.vrbica@email.cz)

Background:

Gonosomal mosaicism is a frequent finding in individuals with apparently full monosomy X; mosaics X/XX and X/i(X)(q10) are the ones encountered most often.

We examined 150 patients originally considered to have Turner syndrome. Karyotyping was performed uniformly in all. The proportion of gonosomes was examined by FISH in cells obtained from three different germ layers: uncultivated lymphocytes from peripheral blood (mesoderm), buccal smear (ectoderm) and smear from the root of the tongue (endoderm). In patients with monosomy X haplotyping was performed to detect the parental origin and the findings were correlated with phenotype.

Aims:

We aim to determine the gonosomal profile in the three germ layers, to verify the hypothesis of hidden mosaicism in Turner syndrome patients, to divide patients into subgroups according to cytogenetic findings, to detect the parental origin of the chromosome X and to correlate laboratory findings with phenotype.

Methods:

The G banded karyotype was performed from 50 metaphases from peripheral lymphocytes. FISH examination of 250 nuclei was performed with centromeric probes (Cytocell) on three germ layers. Haplotyping of X chromosome was done by fragment analysis using ARGUS X-12QS kit and evaluated by ABI 3130 with software gene Mapper v 4.1.

Conclusion:

In accordance with literature reports our most frequent findings are monosomy X, mosaic 45,X/46,XX and isochromosome i(X)(q10) or mosaic 45,X/i(X)(q10).

Our preliminary results show that in almost 20 % of patients the FISH result on uncultivated blood differs from the karyotype. Discordant FISH results in the individual germ layers occurs in more than 20 % of examined patients. This underlines the importance of FISH analysis of uncultivated lymphocytes and buccal smear cells in patients with Turner syndrome. According to this outcome it would be beneficial to reassess the way in which cytogenetic analysis is done for Turner syndrome diagnosis.

Supported by Ministry of Health of the Czech Republic, grant nr. 17-29111A. All rights reserved

Supported by MZ CR – RVO (FNOl, 00098892)

#### 1.P17 Supernumerary marker chromosome with neocentromere formation within 12q2 3q24.33 after complex rearrangement involving chromosome 4 and chromosome 12

##### Yvonne Stratis, Ulrike Siebers-Renelt, Peter Wieacker, Albrecht Röpke

###### University Hospital Münster, Institute of Human Genetics, Münster-Germany

####### **Correspondence:** Yvonne Stratis (yvonne.stratis@ukmuenster.de)

Neocentromere formation has been reported in more than 100 small supernumerary marker chromosomes (sSMC). We report the formation of a neocentromere within a supernumerary marker chromosome derived from chromosome 12 in a five year old patient with developmental delay, microcephaly and hexadactyly. Chromosomal analysis of the boy showed an aberrant karyotype with a derivative chromosome 12 demonstrating additional chromosome 4 specific material inserted into the distal part of the long arm (ins(12;4)(q24.33;q24q28)), a deletion of the chromosomal region 12q2?3-q24.33 on the same homolog and an additional supernumerary small marker chromosome. Array-CGH was performed and revealed a gain of 22.6 Mb in chromosomal region 4q24-q28. FISH analysis confirmed the interstitial insertion of chromosome 4 material into the long arm of the derivative chromosome 12. The marker chromosome stained completely with a wcp12 probe but centromere probes for chromosomes 4 and 12 were both negative suggesting the formation of a neocentromere within region 12q2?3q24.33. In total, the complex rearrangement resulted in a partial trisomy 4q in the boy as a presumable explanation for the phenotype. Conventional chromosomal analysis of the father was normal; the mother’s lymphocytes showed a balanced form of the aberrant karyotype found in her son. The healthy mother possesses the insertion ins(12;4)(q24.33;q24q28), the simultaneous resection of the 12q2?3-12q24.3 material and generation of the additional marker chromosome with this material. During meiosis a normal chromosome 4, the derivative chromosome 12 and the marker chromosome were passed on to the son leading to the unbalanced karyotype as described above. Further analysis with a pan-centromeric and pan-telomeric probe confirmed the existence of a neocentromere on the marker chromosome and the absence of telomeric signals showed that the marker is a ring chromosome. Presenting author: Yvonne.stratis@ukmuenster.de

#### 1.P18 A family with a complex chromosomal rearrangement (CCR) involving chromosomes 11 12 and 18

##### Anette Eek, Anne Signe Bø, Camilla Novy, Anne Gyrid Helland, Trine Prescott

###### Medical Genetics, Telemark Hospital, Skien-Norway

####### **Correspondence:** Anette Eek (anekil@sthf.no)

A six months old dysmorphic boy presented with developmental delay. His six year old half-sister (same father) had impaired hearing, cleft palate and developmental disability.

In the boy, array CGH analysis revealed a 10.5 Mb deletion on chromosome 11q22.1-q23.1. G-band analysis showed an apparently balanced reciprocal translocation between chromosome 11p and 18q (46,XY,t(11;18)(p15;21.1)). FISH analysis verified, as suspected on G-banding, that the 11q deletion involved the translocated chromosome 11.

The boy’s half-sister, investigated elsewhere, had the same unbalanced genomic changes.

Paternal G-band analysis, performed earlier for sub-fertility, revealed the 11;18 translocation. Further testing prompted by the findings in his children, including FISH, detected a paternal interstitial insertion translocation, with 11q22.1-q23.1 material inserted into 12p, explaining the deletion in his children. The same chromosome 11 was involved in both translocations.

So, in fact, the father is a carrier of an apparently balanced type III CCR involving three chromosomes, four breaks and an insertion. Deletion of chromosome 11q22.3 can cause dysmorphic features and intellectual disability. The deletion includes the SDHD gene. Paternally inherited mutations of this imprinted gene lead to increased risk of paragangliomas, necessitating tailored follow-up.

A multiple chromosomal aberration should raise the suspicion of a CCR, and further investigations (e.g. FISH) may be warranted. With the advent of array CGH, insertion translocations have been shown to be more common than previously assumed, perhaps indicating underdetection of CCRs.

Written, informed consent for publication was obtained from the patient [or parent/guardian for patients under 16]

#### 1.P19 Neurodevelopmental disorders associated with recurrent copy number variations of the short arm of chromosome 16

##### Anna Lengyel^1^, Eva Pinti^1^, Henriett Piko^2^, Eszter Javorszky^3^, Mariann Tihanyi^4^, Gyorgy Fekete^1^, Iren Haltrich^1^

###### ^1^Semmelweis University, Ii. Department of Pediatrics, Budapest-Hungary; ^2^Semmelweis University, I. Department of Internal Medicine, Budapest-Hungary; ^3^Semmelweis University, I. Department of Pediatrics, Budapest-Hungary; ^4^Zala County Hospital, Department of Genetics, Zalaegerszeg-Hungary

####### **Correspondence:** Anna Lengyel (lengyel.anna1@med.semmelweis-univ.hu)

The short arm of chromosome 16 (16p) is enriched for segmental duplications (approximately 10% of its sequence), making it susceptible to rearrangements mediated by non-allelic homologous recombination. These recurrent, reciprocal copy number variants of 16p present with variable, unspecific symptomatology, and are implicated in the etiology of several neuropsychiatric phenotypes, including intellectual disability, developmental coordination disorder, specific speech disorders and autism spectrum disorders.

Out of 72 patients who were referred to genetic counselling with intellectual disability and/or developmental delay, dysmorphic features and other congenital anomalies and were analyzed using chromosomal microarray, 11 patients had 12 copy number variations of chromosome 16p.

The following eight corresponded to recurrent copy number variations: (a) one deletion and one duplication of 16p12.2, the region associated with the 16p11.2p12.2 microdeletion/microduplication syndrome and the typical ~500 kb 16p12.2 microdeletion predisposing to neurodevelopmental disorders; (b) two deletions partially overlapped the distal, ~220 kb 16p11.2 region where the obesity associated SH2B1 gene is located; (c) four duplications of the well described BP4-BP5 autism spectrum disorder “hotspot” region of 16p11.2 were uncovered. Finally, we found four rare, non-recurrent proximal 16p11.2 alterations not yet associated with known disorders: one deletion, one large loss of heterozygosity and two duplications.

All patients underwent detailed clinical evaluation, with special emphasis on behavioral symptoms. Results were confirmed using either fluorescence in situ hybridization or polymerase chain reaction. We present the phenotypic and genotypic results of our patients and discuss our findings in relation to the available literature.

#### 1.P20 Relevant unintended finding of genomic imbalance by MLPA lead to the identification of mosaic trisomy 8 in a patient with submucous cleft palate

##### Gloria Soler Sánchez^1^, María Carmen Martínez Romero^1^, Juan Antonio Bafalliu Vidal^1^, Ana Teresa Serrano Antón^2^, Ascensión Vera-Carbonell^1^, Encarna Guillén-Navarro^2^, María Gabriela Cortez Lede^3^, María Angeles Rodríguez González^3^, Isabel López Expósito^1^

###### ^1^Hospital Clínico Universitario Virgen De La Arrixaca, Centro De Bioquímica Y Genética Clínica, Murcia-Spain; ^2^Hospital Clínico Universitario Virgen De La Arrixaca, Sección De Genética Médica. Servicio De Pediatría, Murcia-Spain; ^3^Hospital Clínico Universitario Virgen De La Arrixaca, Servicio De Cirugía Oral Y Maxilofacial, Murcia-Spain

####### **Correspondence:** Gloria Soler Sánchez (gloriasolersanchez@gmail.com)

Although the most common genetic cause of palatal anomalies, particularly velopharyngeal insufficiency, submucosal cleft palate, bifid uvula, and cleft palate is the chromosome 22q11.2 deletion syndrome, there have been several reports of chromosomal aberrations that are associated with these anomalies other than 22q11.2 deletions. One of these chromosomal defects is the mosaic trisomy 8, a rare genetic disorder with an estimated frequency about 1/25,000 to 50,000 liveborns, in which it has been reported about 10% of patients have cleft-palate.

We report a 3-year-old female case that was referred for 22q11.2 genetic evaluation by multiplex ligation-dependent probe amplification (MLPA) due to bifid uvula, submucous cleft palate and velopharyngeal insufficiency. MLPA provided a 22q11.2 normal result, but revealed a small genomic gain in three probes in chromosome 8p23.1 that are used as reference probes as well as for the detection of 8p23.1 duplication syndrome with features overlapping with 22q11.2 deletion syndrome. This unexpected finding was reported and further molecular analysis was advised. Array comparative genomic hybridization (array-CGH) was performed and mosaicism for trisomy 8 was detected. In this way we have been able to see that unexpected findings in genetic testing may provide evidence that leads to the detection of relevant chromosome imbalances, different from the primary purpose of the testing.

Constitutional trisomy 8 mosaicism could have remained undetected because of its wide phenotypic variability. This shows that array-CGH is more suitable for providing information on entire genomic dosage imbalances and should be considered before MLPA studies even if there is a sufficient clinical suspicion of a microdeletion/microduplication syndrome.

#### 1.P21 Association of Down and i(X)(q10) Klinefelter syndromes A unique case of a threefold non disjunction event

##### Eva Pinti, Anna Lengyel, Gyorgy Fekete, Iren Haltrich

###### Semmelweis University, Ii. Department of Pediatrics, Budapest-Hungary

####### **Correspondence:** Eva Pinti (evapinti1991@gmail.com)

A male newborn presented with the typical clinical features of Down syndrome. His classical cytogenetic evaluation identified a 47,XY,+21[80%]/48,XY,+i(X)(q10),+21[20%] karyotype. During the event-free pregnancy there were no signs of any fetal abnormalities. A review of the recent scientific literature yielded 17 pre- and 81 postnatally diagnosed cases of Down-Klinefelter double aneuploidy and 22 reported patients with i(X)(q10) Klinefelter syndrome. To the best of our knowledge, Down-Klinefelter double aneuploidy accompanied by an i(X)(q10) structural abnormality mosaicism has not been described until now. On the basis of these findings we try to predict the possible outcomes of our patient’s condition, and to clarify the etiology.

#### 1.P22 Detection of different complex chromosomal rearrangements as cause of heterozygous SHOX deletions in a father and his two short statured daughters

##### Eva-Maria Krimmel^1^, Uwe Heinrich^1^, Dagmar Wahl^2^, Christina Sofeso^3^, Imma Rost^4^

###### ^1^Center for Human Genetics and Laboratory Diagnostics, Dr. Klein, Dr. Rost and Colleagues, Cytogenetics, Martinsried-Germany; ^2^Medical Practice For Genetic Counseling And Psychotherapy, Clinical Genetics, Augsburg-Germany; ^3^Center for Human Genetics and Laboratory Diagnostics, Dr. Klein, Dr. Rost and Colleagues, Molecular Genetics, Martinsried-Germany; ^4^Center for Human Genetics and Laboratory Diagnostics, Dr. Klein, Dr. Rost and Colleagues, Clinical Genetics, Martinsried-Germany

####### **Correspondence:** Eva-Maria Krimmel (eva-maria.krimmel@medizinische-genetik.de)

The SHOX gene is located on the human pseudoautosomal region1 (PAR1) at the terminal end of the short arms of the gonosomes in Xp22.3 and Yp11.2. Heterozygous SHOX deletions are associated with idiopathic short stature and Leri-Weill-dyschondrosteosis. We report the surprising results, revealed in the context of the performed genetic investigations in a family, after the detection of differently sized SHOX deletions in the two short statured daughters of non-consanguineous parents via MLPA assays. Patient 1, 2-year-old, phenotypically had disproportionate short stature, anorectal malformation and developmental delay. MLPA showed a deletion in PAR1 (about 592 to 883 kb). Patient 2, the 4-year old sister, showed short stature only. MLPA detected a SHOX deletion in PAR1 (about 444 to 701 kb). After genetic counselling of the parents a varying combination of chromosome, FISH and microarray analyses and quantitative PCR was performed. Results: Patient 1: mosaicism with two aberrant cell lines [karyotype: 45,X,dic(X;18)(p22.33;p11.21)[7]/45,X[3] de novo] resulting in a deletion Xp22.33 (including SHOX), a deletion 18pter-p11.21 (De-Grouchy-syndrome I) and mosaicism for monosomy X (Ullrich-Turner-syndrome variant). Additional finding of a deletion 15q11.2 (Burnside-Butler syndrome) is inherited from the mother. Patient 3 (father 168 cm high): male karyotype with a derivative chromosome Y derived from a reciprocal translocation [der(Y)(t(Y;7)(p11.2;q36.3)] resulting in a deletion Yp11.32p11.2 (including SHOX) and a duplication 7q36.3. Patient 2: female karyotype with a paternally transferred recombinant chromosome X showing deletion of Xp22.33. (including SHOX) and terminal translocation of 7q36.3 to the short arm [Karyotype: 46,XX.ish rec(X)t(X;7)der(Y)t(Y;7)(p11.2;q36.3)pat]. The findings show that chromosomal rearrangements involving PAR1 can cause SHOX deletions and that only the combined application of different methods could reveal the complex situation in this family.

Written, informed consent for publication was obtained from the patient [or parent/guardian for patients under 16]

#### 1.P23 Shifting to skewed X chromosome inactivation pattern reduces the penetrance of X linked intellectual disability associated CNV in women with a history of pregnancy loss

##### Igor Lebedev^1^, Ekaterina Tolmacheva^1^, Elizaveta Fonova^1^, Anna Kashevarova^1^, Nikolay Skryabin^2^, Tatyana Nikitina^1^, Elena Sazhenova^1^, Maria Lopatkina^2^, Elena Belyaeva^2^, Larisa Minaycheva^3^, Lyudmila Nazarenko^4^

###### ^1^Research Institute of Medical Genetics, Tnrmc, Laboratory Of Cytogenetics, Tomsk-Russia; ^2^Research Institute of Medical Genetics, Tnrmc, Laboratory Of Molecular Diagnostics, Tomsk-Russia; ^3^Research Institute of Medical Genetics, Tnrmc, Genetic Clinics, Tomsk-Russia; ^4^Research Institute of Medical Genetics, Tnrmc, Laboratory Of Hereditary Pathology, Tomsk-Russia

####### **Correspondence:** Igor Lebedev (igor.lebedev@medgenetics.ru)

The reason for incomplete penetrance of the large-scale chromosomal copy number variation (CNV) in human genome is still debated. One of the possible compensatory mechanisms may be related to intrinsic genetically and developmentally determined epigenetic chromatin modifications. For example skewed X-chromosome inactivation (sXCI) may have a protective effect for a woman's health by suppressing X-linked CNVs in her karyotype. However, one can suggest, that inheritance of these CNVs from maternal side by the embryo may be incompatible with its normal development. If this true, male embryos or female embryos with random X-inactivation are in the elevated risk group in families with pregnancy loss. To test this hypothesis, X-inactivation status was estimated by classical methylation-specific assay in blood lymphocytes from 203 women, heterozygous for AR locus, with a history of spontaneous abortions with normal karyotype as well as in 97 women without reproductive problem, having two or more pregnancies with successful outcome. The incidence of sXCI was significantly higher in women with pregnancy loss than in the control (21% and 7%, respectively, p=0.009). Subsequent molecular karyotyping of 9 women with reproductive losses and extreme sXCI (≥90%) using SurePrint G3 Human CGH+SNP 4×180K Microarray Kit (Agilent Technologies, USA) revealed X-linked CNVs for eight of them presented by Xp22.33, Xq28 microduplications and Xp11.23, Xq24 microdeletions. Surprisingly, enrichment analysis revealed that genes localized within CNVs are associated with intellectual disability (RAB39B, UBE2A, L1CAM, CLIC2, p=0.0043), including X-linked syndromic mental retardation, Nascimento type (MIM 300860) and Xq28 microduplication syndrome (MIM 300815). An observed link between pregnancy loss and risk for birth of a child with developmental delay or intellectual disability requires special attention for further elucidation of essential genetic and epigenetic mechanisms related to prenatal selection and accumulation of X-linked CNVs in the population. This study is supported by the Russian Foundation for Basic Research, grant 18-015-00437.

#### 1.P24 P1070 - 9q21.13q21.31 deletion in a patient with intellectual disability severe speech delay and and dysmorphic features a newly recognized microdeletion syndrome

##### Barbara Marques^1^, Silvia Serafim^1^, Sonia Pedro^1^, Ana Rita Tarelho^1^, Cristina Ferreira^1^, Rui Gonçalves^2^, Hildeberto Correia^1^

###### ^1^Instituto Nacional De Saúde Doutor Ricardo Jorge, Ip, Departamento Genética Humana - Unidade De Citogenética, Lisboa-Portugal; ^2^Hospital Dona Estefânia, Centro Hospitalar De Lisboa Central Epe, Serviço De Genética, Lisboa-Portugal

####### **Correspondence:** Barbara Marques (barbara.marques@insa.min-saude.pt)

The increased use of chromosomal microarray analysis has led to the identification of new microdeletion/microduplication syndromes, enabling better genotype-phenotype correlations.

Interstitial deletions involving the long arm of chromosome 9 are rare but recently a microdeletion syndrome at 9q21.13 was suggested, with mental retardation, speech delay, epilepsy, autistic behaviour and moderate facial dysmorphism as the main characteristics.

Here we present a male child with intellectual disability, severe speech delay, microcephaly and dysmorphic features carrying an interstitial deletion, detected by the Affymetrix Cytoscan HD microarray, of 6.56 Mb at 9q21.13q21.31 region encompassing 16 OMIM genes (arr[GRCh37] 9q21.13q21.31(76551542_83116342)x1).

Among the genes in the deleted region the PRUNE2, PCSK5, RORB and TRPM6 genes are expressed in the nervous system and have been describe as being candidate genes to play a role in mental retardation or neurological disorders. Although the cohort of patients identified with deletions in this region is still small our patient phenotype partially overlaps the others described in the literature.

The collection of more cases with deletion of the 9q21.13 region will help establishing a clear classification for this CNV, finding the real weight in the patient’s phenotype, delineating the genetic counseling for their families, and clearly establishing this microdeletion as a syndrome.

#### 1.P25 Rare congenital chromosomal aberration dic(X;Y)(p22.33;p11.32) in adult patient with primary myelofibrosis

##### Lenka Pavlistova^1^, Silvia Izakova^1^, Karla Svobodova^1^, Lucie Bartuskova^1^, Martina Langova^2^, Pavlina Malikova^3^, Kyra Michalova^1^, Zuzana Zemanova^1^

###### ^1^Institute of Clinical Biochemistry and Laboratry Diagnostics, General University Hospital and 1st Faculty of Medicine, Charles University in Prague, Center of Oncocytogenetics, Prague-Czech Republic; ^2^Thomayer’s Faculty Hospital, Department of Medical Genetics, Prague-Czech Republic; ^3^Institute of Clinical and Experimental Medicine, Department of Clinical Hematology, Prague-Czech Republic

####### **Correspondence:** Lenka Pavlistova (lenka.pavlistova@vfn.cz)

Constitutional translocations between sex chromosomes are rather rare in humans. We report the unique case of a very rare congenital translocation between chromosomes X and Y, forming dicentric chromosome dic(X;Y)(p22.33;p11.32), in an adult male with a malignant hematological disease.

Primary myelofibrosis was diagnosed in a 63-year-old man following liver transplantation after hepatocellular carcinoma. Analysis of the bone marrow sample showed a karyotype 46,X,dic(X;Y)(p22.33;p11.32) in all mitoses; this was verified with mFISH. A cytogenetic examination of stimulated peripheral lymphocytes revealed the constitutional karyotype 46,X,dic(X;Y)(p22.33;p11.32)[20]/45,X[10]. The 45,X cell line was confirmed with FISH in 35% of interphase nuclei. The SRY locus was present on the dicentric chromosome. A CGH/SNP array (Illumina) revealed gain of 153,7 Mbp of the X chromosome and 803-kbp microdeletion (including the SHOX gene), which were also confirmed with FISH. SHOX encodes a transcriptional factor that regulates the growth of the long bones. The deletion of the SHOX gene together with the Madelung deformity of the forearm and the short stature of the proband led to a diagnosis of Léri-Weill dyschondrosteosis (LWD). The gain of almost the whole X chromosome (153,7 Mbp) was considered a variant of Klinefelter syndrome (KS). The levels of gonadotropins and testosterone were consistent with gonadal dysfunction. A malformation of the right external ear was detected.

We have reported a rare structural aberration dic(X;Y)(p22.33;p11.32) in a patient with primary myelofibrosis. The related genomic imbalance is associated with two known hereditary syndromes, LWD and a KS variant, identified in our proband at an advanced age. Because the breakpoints did not involve known cancer genes, we inferred that the hematological malignant disease suffered by the proband is unrelated to this congenital chromosomal aberration.

Supported by RVO-VFN64165.

#### 1.P26 Case report of a small deletion in 1p36.11 encompassing the AHDC1 gene causative for Xia Gibbs syndrome

##### Susanne Anders^1^, Stephanie Demuth^2^, Anne Gocht^1^, Birgit Eichhorn^3^, Mirjam Klaus^1^, Annelore Junge^1^

###### ^1^Mvz Mitteldeutscher Praxisverbund Humangenetik, Cytogenetics, Dresden-Germany; ^2^Mvz Mitteldeutscher Praxisverbund Humangenetik, Genetic Counseling, Erfurt-Germany; ^3^Mvz Mitteldeutscher Praxisverbund Humangenetik, Molecular Genetics, Dresden-Germany

####### **Correspondence:** Susanne Anders (Susanne.Anders@genetik-dresden.de)

Several patients with de novo mutations in the AHDC1 gene have been described since Xia et al. presented the Xia-Gibbs syndrome (OMIM #615829) in 2014. Patients exhibit common features like developmental delay, hypotonia, language delay, mild dysmorphic features, sleep apnoea, seizures, and hypoplasia of corpus callosum. The AHDC1 gene encodes a nuclear protein with two AT-hook DNA binding motifs, however its function is still unknown. It is located proximal to the well-known chromosome region of the monosomy 1p36 syndrome (OMIM #607872). To the best of our knowledge, only three patients with a microdeletion including the AHDC1 gene have been published in the literature to date.

Here, we report a case of a two-year-old boy with severe motor and language delay, sleep apnoea as well as minor facial dysmorphic signs from a non-consanguineous couple. Oligonucleotide array CGH revealed a 1p36.11 microdeletion encompassing the AHDC1 gene with a size of about 136 kb. This is the smallest deletion in the sub-band 1p36.11 being causative for Xia-Gibbs syndrome described so far. The array CGH of both parents showed no abnormalities, approving the de novo origin of the deletion.

Our report provides further evidence that a haploinsufficiency of AHDC1 is disease causing for the Xia-Gibbs syndrome. Additionally, this case report emphasizes the critical role of AHDC1 gene alterations in patients with developmental delay especially with severe language delay. Therefore, this gene should be considered for routine diagnostics for patients with profound language impairment.

Written, informed consent for publication was obtained from the patient [or parent/guardian for patients under 16]

#### 1.P27 Two unbalanced recombinant cases in a family with an intrachromosomal insertion in 1q

##### Bettina Pfütze^1^, Viola Langhof^1^, Susanne Anders^1^, Peter Lorenz^2^, Thomas Liehr^3^, Mirjam Klaus^1^, Annelore Junge^1^

###### ^1^Mvz Praxisverbund Humangenetik, Cytogenetics, Dresden-Germany; ^2^Mvz Praxisverbund Humangenetik, Genetic Counseling, Meerane-Germany; ^3^Jena University Hospital, Institute of Human Genetics, Jena-Germany

####### **Correspondence:** Bettina Pfütze (bettina.pfuetze@genetik-dresden.de)

We report on a male infant with bilateral foot malposition, cranial deformation, unilateral ear dysplasia, unilateral inguinal testicles and suspected double kidney. Conventional karyotyping showed a non-homologous banding pattern in the long arm of one chromosome 1, which was suspected to be the result of an intrachromosomal rearrangement after whole chromosome painting fluorescence in situ hybridization (FISH). Array CGH revealed a duplication of 1q12→q23.1 and targeted FISH confirmed the duplication of 1q12→q23.1. The karyotype of the phenotypically normal father also showed an aberrant chromosome 1, while array CGH showed no imbalances. Clarification of the aberration using Multi-color banding FISH revealed a paracentric intrachromosomal insertion ins(1)(q42.1q12q23.1) in the father’s karyotype. The unbalanced karyotype in our index patient is a result of a recombination event during paternal meiosis and was described as 46,XY,rec(1)dup(1)ins(1)(q42.1q12q23.1)pat. Further cytogenetic investigation of other family members revealed two more balanced carriers of the ins(1). One of them has a 22 year old, severely handicapped son harboring the same recombinant unbalanced insertion like our index patient. This case description illustrates that carriers of insertions - depending on size and position of the inserted segment - have an increased risk of chromosomally unbalanced offspring. To the best of our knowledge the existence of two relatives harboring the identical unbalanced karyotype in consequence of a familial insertion is a very rare event.

#### 1.P28 An unusual apparently balanced de novo complex chromosomal rearrangement in a child with a mild phenotype

##### Nadezda Shilova^1^, Marina Minzhenkova^1^, Zhanna Markova^1^, Darya Guseva^2^

###### ^1^Research Centre for Medical Genetics, Laboratory of Cytogenetics, Moscow-Russia; ^2^Research Centre for Medical Genetics, Department of Clinical Genetics, Moscow-Russia

####### **Correspondence:** Nadezda Shilova (nvsh05@mail.ru)

Complex chromosomal rearrangements (CCRs) are rare structural genome variations that involve multiple breaks with exchanges of chromosomal segments. CCRs can be de novo or familial. The occurrence of de novo CCRs is associated with an increased risk of mental retardation and congenital abnormalities. We report on a 1 year 2 months old child, who had relatively mild dysmorphic features such as short neck, hypertelorism, broad filtrum, hypospadias and bilateral cryptorchidism. Two three-way CCRs were detected by conventional karyotyping using GTG-banding. Parental karyotypes were normal. FISH with various DNA-probes showed great complexity of CCR, involving six chromosomes and eight breakpoints. Final karyotype was : t(Y;6;1)(Ypter→Yq12::6q13→6qter;1pter→1p31::6p22→6q13::Yq12→Yqter;6pter→6p22::1p31→1qter),t(8;13;15)(8pter→8q12::13q34→13qter;13pter→13q12::15q26→15qter;15pter→15q26::13q12→13q34:: 8q12→8qter)dn. The first CCR t(Y;6;1) involved three chromosomes and four breakpoints; the der(6) was made up of segments from 1p, the centric segment of 6 and a segment of Yq . The second CCR, t(8;13;15), involved three chromosomes, four breakpoints and an insertion of 13q12→13q34 into the der(15). The Chromosomal microarray test revealed deletions of two genomic segments: 3,2 Mb at 8p12 and 1,7Mb at 8p11.1p11.21. Although the overall size of these two microdeletions was 5 Mb, the significance of these CNVs is not clear. Whole genome sequencing is required to provide some insight into genotype–phenotype correlations. Further (mental) development will be of interest but so far the mild phenotype of the child, considering the complexity of this de novo rearrangement, is remarkable

#### 1.P29 Dup15q11.2 and del22q11.2 in a newborn a challenge for cytogeneticists

##### Cristina Candeias^1^, Manuela Mota Freitas^1^, Sílvia Pires^2^, Paula Rendeiro^3^, Raquel Lemos^3^, Joaquim Sá^4^, Cíntia Ventura^3^, Sara Domingues^5^, Ana Cristina Barros^5^, Céu Mota^5^, Natália Oliva-Teles^1^

###### ^1^Centro De Genética Médica Doutor Jacinto Magalhães/centro Hospitalar Do Porto-epe; Unit For Multidisciplinary Research In Biomedicine/icbas, Cytogenetics, Porto-Portugal; ^2^Centro De Genética Médica Doutor Jacinto Magalhães/centro Hospitalar Do Porto-epe, Cytogenetics, Porto-Portugal; ^3^Cgc Genetics/unilabs, Cytogenetics, Porto-Portugal; ^4^Cgc Genetics/unilabs, Medical Genetics, Porto-Portugal; ^5^Centro Materno Infantil Do Norte/centro Hospitalar Do Porto-epe, Neonatology, Porto-Portugal

####### **Correspondence:** Cristina Candeias (cristina.candeias@gmail.com)

The presence of a duplication and a deletion of non-subtelomeric regions in the same patient is a rare event. Duplication of 15q11.2 (OMIM # 608636) and deletion of 22q11.2 (OMIM #188400) each leads to a well-known syndrome but their presence in the same patient is very unusual.

The authors present the case of a newborn referred for cytogenetic studies due to multiple malformations. Array CGH was performed using Cytoscan 750K (Applied Biosystems), followed by chromosome analysis with GTL banding and MLPA panels P250-B2 and P343-C3 (Salsa® MLPA®, MRC Holland) in both the proband and her parents. FISH analysis using UBE3A, TUPLE1 and centromere #15 probes was performed.

Chromosomal microarray analysis revealed both a 4,05Mbp duplication in 15q11.2 and a 3,424Mbp deletion in 22q11.2. All karyotypes were normal and the microarray result was confirmed by MLPA in the proband. On finding a family history of Angelman syndrome, FISH using UBE3A probe was performed in the mother and showed a signal in a group G chromosome. Since chromosome 22 was partially deleted in the index case, FISH TUPLE1 probe was used to confirm the presence of a balanced translocation between 15q11.2 and 22q11.2; and applying FISH #15 centromere probe proved that both centromeres 15 and 22 were involved in the translocation. The mother carries a whole arm translocation with two breakpoints, 15q11.2 and 22q11.2. This cryptic translocation was later shown to have been inherited from her mother.

This case of a three-generation translocation transmission alerts us to the importance of good clinical and family history which, when combined with different and complementary cytogenetic techniques, such as FISH, unravels abnormalities of balanced origin that would otherwise be missed or misinterpreted, thus providing better genetic counselling for patients and their families.

#### 1.P30 Small supernumerary marker chromosome a case of Cat eye syndrome

##### Manuela Mota Freitas^1^, Fernanda Paula Oliveira^2^, Cristina Candeias^1^, Sara Felício^2^, Ana Rita Soares^3^, Ana Fortuna^4^, Natália Oliva-Teles^1^

###### ^1^Centro De Genética Médica Doutor Jacinto Magalhães/centro Hospitalar Do Porto-Epe; Unit For Multidisciplinary Research In Biomedicine/ Icbas, Cytogenetics, Porto-Portugal; ^2^Centro De Genética Médica Doutor Jacinto Magalhães/centro Hospitalar Do Porto- Epe, Cytogenetics, Porto-Portugal; ^3^Centro De Genética Médica Doutor Jacinto Magalhães/centro Hospitalar Do Porto- Epe, Medical Genetics, Porto-Portugal; ^4^Centro De Genética Médica Doutor Jacinto Magalhães/centro Hospitalar Do Porto- Epe; Unit For Multidisciplinary Research In Biomedicine/ Icbas, Medical Genetics, Porto-Portugal

####### **Correspondence:** Manuela Mota Freitas (manuela.motafreitas@gmail.com)

Cat eye syndrome (CES, OMIM #115470) is clinically characterized by the combination of coloboma of the iris and anal atresia with fistula, downslanting palpebral fissures, frequent occurrence of heart and renal malformations and normal or near-normal mental development. CES is generally associated with a supernumerary marker chromosome containing material of chromosome 22, causing tetrasomy of 22p and part of 22q11.2. The authors describe a rare case of Cat eye syndrome detected in a two-year-old child referred for karyotype to our laboratory presenting with a malformation syndrome.

Chromosome analysis was performed on GTL banded metaphases obtained from peripheral blood cultures, complemented with CBG and AgNOR banding. To identify the origin and composition of the marker chromosome, fluorescent in situ hybridization (FISH) with probes for centromeres acrocentric chromosomes (Cytocell) and MLPA technique using panel Subtelomeres (P036, P070) and panel DiGeorge Syndrome (P250, MRC-Holland) were performed. Parental karyotypes have been requested.

The standard cytogenetics techniques revealed that the supernumerary marker chromosome was bisatellited and dicentric in all cells. A partial tetrasomy 22q11.2, which includes genes IL17RA, SLC25A18, BID, MICAL3 and USP18, associated with Cat eye syndrome, was identified by molecular cytogenetic techniques.

The authors compare the child’s phenotype with a previously published case and enhance the importance of the correct characterization of marker chromosomes for an adequate genetic counselling.

#### 1.P31 Axenfeld Rieger anomaly associated with a 6p25 interstitial deletion Case report

##### Elisa Lopes^1^, Sílvia Pires^1^, Cristina Candeias^2^, Manuela Mota Freitas^2^, Cláudia Falcão Reis^3^, Natália Oliva-teles^2^

###### ^1^Unidade De Citogenética, Centro Genética Médica Doutor Jacinto Magalhães/centro Hospitalar E Universitário Do Porto - Epe, Genética, Porto-Portugal; ^2^Unidade De Citogenética, Centro Genética Médica Doutor Jacinto Magalhães/centro Hospitalar E Universitário Do Porto - Epe; Umib/icbas, Genética, Porto-Portugal; ^3^Serviço De Genética Médica, Centro Genética Médica Doutor Jacinto Magalhães/centro Hospitalar E Universitário Do Porto - Epe, Genética, Porto-Portugal

####### **Correspondence:** Elisa Lopes (elisa.lopes@chporto.min-saude.pt)

Axenfeld-Rieger syndrome is genetically heterogeneous and approximately 16% of cases are caused by heterozygous mutations in the FOXC1 gene, a transcription factor regulating neural crest cell development located on chromosome 6p25.3. Axenfeld-Rieger syndrome type 3 (OMIM: #602482) is a rare autosomal dominant disorder characterized by anomalies of the anterior segment of the eye and systemic signs including craniofacial dysmorphism, sensorineural hearing loss and cardiac defects. There is clinical overlap between Axenfeld-Rieger syndrome and 6pter-p24 deletion syndrome (OMIM: #612582), a microdeletion syndrome characterized by heterozygous loss of FOXC1 and contiguous genes. In both syndromes, FOXC1 haploinsufficiency is considered pathogenic.

The authors present a patient with a 46,XY,del(6)(p25.3p25.2) karyotype with typical clinical features of 6pter-p24 deletion syndrome, including craniofacial dysmorphism (broad forehead, hypertelorism, midface hypoplasia), mixed hearing loss, posterior embryotoxon, mild intellectual disability, multiple cerebral subcortical white matter lesions, and, additionally, intracranial calcifications.

Standard chromosome analysis is usually unrevealing, and the clinical diagnosis mainly confirmed by molecular cytogenetic procedures, however in this case the high resolution chromosome banding allowed us to identify the deletion. The size and breakpoints of the deleted region were further characterized using comparative genomic hybridization arrays: 1,92Mb and 6p25.3-6p25.2, respectively – arr[GRCh37] 6p25.3p25.2(510590_2435100)x1.

The authors discuss the presence or absence of significant genes and review the clinical features of Axenfeld-Rieger and 6pter-p24 deletion syndromes, while establishing the genotype-phenotype correlation between this patient and previous cases described in the literature.

#### 1.P32 Cytogenetic characterization of a terminal deletion of the X chromosome found by array CGH revealed a mosaic karyotype with an isodicentric X chromosome

##### Alexandra Mascarenhas^1^, Susana Ferreira^1^, Cláudia Pais^1^, Isabel Marques Carreira^2^, Joana Barbosa Melo^2^

###### ^1^Faculty of Medicine, University of Coimbra, Cytogenetics and Genomics Laboratory, Coimbra-Portugal; ^2^ICBR-cimago - Center of Investigation on Environment Genetics And Oncobiology - Faculty of Medicine, University of Coimbra, Cytogenetics and Genomics Laboratory, Coimbra-Portugal

####### **Correspondence:** Joana Barbosa Melo (alexandramascarenhas@gmail.com)

Introduction: Isodicentric X chromosomes are uncommon and usually occur in a mosaic form with a 45,X cell line. An isodicenrtic can be formed by end-to-end fusion of chromatids, following a break and subsequent loss of the acentric fragment. Many of these cases are mosaics which makes phenotype-genotype correlations difficult.

Case Report: We present a girl with developmental delay, dysmorphisms and obesity. Array comparative genomic hybridization analysis (aCGH) revealed a 40 Mb terminal deletion of the long arm of chromosome X (Xq23q28). Conventional cytogenetics of the girl and her parents was performed in order to characterize the origin of the deletion. GTG-banded chromosome analysis revealed a mosaic female karyotype with two cell lines. One cell line (64% of the metaphases) with a large isodicentric X chromosome, resulting in terminal deletion of the long arm of chromosome X and a duplication of the whole short arm and almost half of the long arm of chromosome X. The other cell line (26% of the metaphases) showed a 45,X karyotype. Karyotypes of both parents were normal, showing that the isochromosome had arisen de novo.

Conclusion: aCGH is now adopted as a first-tier clinical diagnostic test in individuals with unexplained developmental delay/intellectual disability and congenital anomalies. Mosaicism involving 45,X/46,X,idic(X) is associated with variable phenotypes. Moreover, patients that present Xq deletion due to the isodicentric X chromosome show different clinical features from those with Xp deletion. The present study highlights the importance of the cytogenetic characterization of an aCGH detected imbalance, as it can mask a complex rearrangement coupled with mosaicism. The combination of multiple technologies allows a correct diagnosis and an improved genetic counseling.

#### 1.P33 Familiar Microdeletion syndrome a case report

##### Marta Souto^1^, Pedro Botelho^1^, Susana Sousa^2^, Márcia Martins^3^, Osvaldo Moutinho^4^, Rosário Pinto Leite^1^

###### ^1^Centro Hospitalar De Trás-Os-Montes E Alto Douro, Laboratório De Genética, Vila Real-Portugal; ^2^Centro Hospitalar De Trás-Os-Montes E Alto Douro, Serviço De Pediatria, Vila Real-Portugal; ^3^Centro Hospitalar De Trás-Os-Montes E Alto Douro, Consulta De Genética, Vila Real-Portugal; ^4^Centro Hospitalar De Trás-Os-Montes E Alto Douro, Serviço De Ginecologia/obstetrícia, Vila Real-Portugal

####### **Correspondence:** Rosário Pinto Leite (mlleite@chtmad.min-saude.pt)

INTRODUTION: The 1q21.1 microdeletion is a very rare syndrome, up to date there are only 55 cases described. This disorder demonstrates a heterogeneous spectrum of manifestations including developmental delay (speech and motor delays), mild intellectual disability, microcephaly, slight facial dysmorphic features and eye abnormalities. Less frequently are heart defects, abnormalities of the genitalia or urinary system, bone abnormalities (particularly in the hands and feet), and hearing loss. The authors present a case of a familiar microdeletion syndrome.

CLINICAL REPORT: 10 year old boy with a microdeletion on 1q21.1q21.2 chromosome region detected by array Comparative Genomic Hybridization (aCGH). The segment deleted was 2.6 Mbp. The child has development delay, particularly affecting the development of motor skills, peculiar facies, and cryptorchidism (already operated). The cerebral resonance revealed left hippocampal malformation and mild cortical-subcortical atrophy.

Parents’ aCGH analysis revealed a similar deletion on the father with the segment deleted smaller (1.935 Mbp) than the son. Although the father has not yet come to the genetic consultation, it is known that he has a slight delay.

DISCUSSION: The present case is a familiar deletion of the 1q21.1q21.2 region, with different manifestations. The deletion was inherited from the father, who showed mild mental retardation, while the son presented phenotypic features consistent with 1q21.1 deletion syndrome, like intellectual disability, motor developmental delay and genitourinary anomalies.

In the 1q21.1 syndrome, the deletion size varies from 1.35 kb to 2 Mb and at least 50% of the deletions are inherited, with parents presenting mild abnormal or completely normal clinical findings. This suggests that the 1q21.1 microdeletion has reduced penetrance and variable expressivity.

All new cases detected should be reported in order to obtain a more precise correlation between genotype/phenotype to be used in genetic counseling.

Written, informed consent for publication was obtained from the patient [or parent/guardian for patients under 16]

#### 1.P34 Malan syndrome in a patient with 19p13.2 deletion encompassing NFIX and CACNA1A genes clinical implication and review of the literatüre

##### Fernanda Teixeira Bellucco^1^, Claudia Berlim De Mello^2^, Vera Ayres Meloni^1^, Maria Isabel Melaragno^1^

###### ^1^UNIFESP - Universidade Federal De São Paulo, Department of Morphology And Genetics, São Paulo-Brazil; ^2^NIFESP - Universidade Federal De São Paulo, Department of Psychobiology, São Paulo-Brazil

####### **Correspondence:** Fernanda Teixeira Bellucco (fernandatsb@gmail.com)

Malan syndrome is a recently introduced clinical condition described in a limited number of individuals. Haploinsufficiency of the NFIX gene, located in the 19p13.2 region, has been proposed as the leading causative mechanism. To date, patients described present microdeletions in the 19p13.2 region or mutations in NFIX. The only difference among them seems to be the increasing prevalence of seizures or epilepsy when the CACNA1A gene is included in the deleted region. Due to the limited number of cases and different deletion sizes, genotype/phenotype correlations are still limited. Here, we report a new case of Malan syndrome caused by a 990 kb deletion in 19p13.2, characterized by G-banding, MLPA, and array techniques. On physical examination, he showed macrocephaly, frontal bossing, deep-set eyes, pointed chin, micropenis, hypotonia, postnatal overgrowth, developmental delay, severe thoracolumbar scoliosis, which was surgically corrected, and seizures. Neuropsychological assessment, including intellectual (WASI; VABS-II) and behavioral (CBCL) measures, showed overall and severe cognitive, language and adaptive functions impairments. The 19p deleted region of our patient encompasses, besides NFIX and CACNA1A, 20 other genes. Among them, CC2D1A and NACC1 are associated with mental retardation, epilepsy and delayed brain myelination, which could contribute to the severe cognitive and adaptive functions impairments found in our patient. Apart from postnatal overgrowth and psychomotor developmental delay, the most consistent physical features among all patients with deletions encompassing the NFIX gene are hypotonia, skeletal anomalies, mainly scoliosis, and facial dysmorphisms that are consistent with the phenotypic findings in our patient. These data emphasize the importance of detailed cytogenomic and clinical analyses for an accurate diagnosis, prognosis, and genetic counseling and provide an opportunity to improve genotype-phenotype correlations of Malan syndrome patients. Financial Support: FAPESP, Brazil.

#### 1.P35 X chromosome inactivation spread into autosomal sequences replication timing analysis in patients carrying unbalanced X autosome translocations

##### Bianca Favilla^1^, Vera Ayres Meloni^1^, Ana Beatriz Perez^1^, Danilo Moretti‑Ferreira^2^, Fernanda Teixeira Bellucco^1^, Maria Isabel Melaragno^1^

###### ^1^Unifesp - Universidade Federal De São Paulo, Department of Morphology And Genetics, São Paulo-Brazil; ^2^Unesp - Universidade Do Estado De São Paulo, Department of Genetics, São Paulo-Brazil

####### **Correspondence:** Bianca Favilla (biancafavilla1@gmail.com)

X-chromosome inactivation (XCI) is an epigenetic mechanism that silences genes on one X chromosome in females. Even though both X chromosomes have the same chance of being inactivated in normal female cells, patients carrying structural rearrangements involving the X usually have a skewed XCI pattern. In unbalanced X-autosome translocations, for instance, the derivative X is often inactivated, and it is suggested that autosomal genes may also undergo inactivation, due to spreading of XCI onto the autosomal segment. Here, we describe three patients carrying different unbalanced X-autosome translocations, confirmed by G-banding karyotype and array techniques. We have analyzed their XCI pattern and inactivation spread into autosomal regions, by performing HUMARA, ZDHCC15 gene assay and 5‑ethynyl‑2′‑deoxyuridine (EdU) assay. There was an extremely skewed XCI pattern for all the patients and a variable replication timing of the autosomal regions on the derivative chromosomes. Since all patients presented phenotypic features that could not be entirely associated with the genomic imbalances that were found on CGH-array, we hypothesized that the spread of XCI into autosomal regions could possibly be responsible for some of their features. By comparing their phenotype with other patients’ presenting deletions of the late-replicating regions, we could identify an overlap of phenotypic features, suggesting that the spread of XCI into autosomal regions may be responsible for their highly variable phenotype. Thus, our data corroborate the hypothesis of XCI spread into autosomal regions as well as highlight EdU assay as a useful tool to evaluate inactivation status in such cases. Financial Support: FAPESP, Brazil.

#### 1.P36 Chromosome Y structural abnormalities in females with gonadal dysgenesis

##### Liubov Tavokina^1^, Alexandra Gorodna^2^, Kateryna Kozyrieva^2^, Dmytro Mykytenko^3^, Ludmila Livshits^2^

###### ^1^Clinic Adonis Family, The Cytogenetics Laboratory, Kyiv-Ukraine; ^2^Institute of Molecular Biology and Genetics, Human Genomics Department, Kyiv-Ukraine; ^3^Clinic of Reproductive Medicine “nadiya”, Molecular Genetics Department, Kyiv-Ukraine

####### **Correspondence:** Liubov Tavokina (l.tavokina@gmail.com)

Different structural abnormalities of the Y chromosome are commonly identified in patients with wide range of phenotypes from males with spermatogenetic failure to gonadal dysgenesis in women with signs of Turner syndrome.

We investigated two cases of females with gonadal dysgenesis. Cytogenetic studies were performed on blood lymphocytes according to standard protocols (GTG-banding, FISH - CEP, LSI).

The first patient revealed a karyotype with isodicentric chromosome Y, two copies of the SRY locus and four copies DYZ3 locus: 47,X,idic(Y)(pter→q11.2:q11.2→pter),+mar.ishidic(Y)(p11.1-q11.2)(SRY++,DYZ3++,DYZ1-;DYZ3++). The second patient on the other hand revealed karyotype with der(Y), one copy of the SRY locus, two copies of the Yq12 locus (DYZ1) and one copy of locus Yp11.1-q11.1 (DYZ3): 46,X,der(Y)(p11.32). ish der( Y)(p11.3-q12)( SRY+,DYZ1++, DYZ3+).

Cytogenomic studies were performed on DNA extracted from a peripheral blood sample by using the MicrogenChip60K Array-CGH. We detected the following complex results: for the first patient – PAR1 duplication (Yp11.32p11.2)x2, PAR2 deletion (Yq12)x0; for the second patient - PAR1 deletion (Yp11.32p11.2)x0.

We suggest that most functionally significant genes, such as SRY, PPP2R3B, CRLF2, ZFY, RPS4Y1, TGIF2LY, involved in duplication (first patient) and deletion (second patient) of PAR1 region can contribute to the development of DSD-phenotypes. It was shown that SRY, ZFY and CRLF2 genes are strongly involved in gonadal development during embryogenesis. PPP2R3Band RPS4Y1may contribute to the development of Noonan and Turner syndromes respectively. It is also interesting to mention that TSPY, FAM197Y, TTTY gene families involved in revealed CNVs are transcripted in testis and involved in spermatogenesis. The further investigation of the revealed CNVs regions and implicated genes will give more comprehensive understanding of complex phenotypes for such patients.

#### 1.P37 Balanced de novo X autosome translocation disrupting the ATP7A gene in the girl with Menkes disease

##### Marlena Młynek^1^, Victor Murcia Pieńkowski^2^, Dorota Wicher^1^, Dorota Gieruszczak-Białek^2^, Małgorzata Rydzanicz^2^, Joanna Kosińska^3^, Aleksandra Marczak^1^, Małgorzata Krajewska-Walasek^1^, Marzena Kucharczyk^1^, Krystyna Chrzanowska^1^

###### ^1^Children's Memorial Health Institute, Department of Medical Genetics, Warsaw-Poland; ^2^Warsaw Medical University, Department of Medical Genetics, Warsaw-Poland; ^3^Warsaw Medical University, Postgraduate School of Molecular Medicine, Warsaw-Poland

####### **Correspondence:** Marlena Młynek (m.mlynek@ipczd.pl)

Menkes disease (MD; MIM 309400) is a rare, X-linked recessive, and severe neurodegenerative disorder of the copper metabolism caused by mutation in the ATP7A gene. Absence or profound reduction of the ATP7A activity leads to the copper distribution impairment and dysfunction of large number of the copper-requiring enzymes. MD usually affects males. To date, 19 females with MD phenotypes have been reported.

Here, we describe the clinical manifestations and laboratory findings in a 1,5 years-old girl with MD. The diagnosis of MD was based on clinical features: sparse and kinky hair, developmental delay with regression, hypotonia and cutis laxa. Ceruloplasmin concentration was decreased, microscopic hair examination confirmed pili torti, abdominal sonography showed the bladder diverticulosis, MRI revealed the delayed myelination and narrow corpus callosum without rostrum. The EEG was abnormal but seizures were not observed. In echocardiography pulmonary valve stenosis and dilatation of the ascending aorta were reported.

Conventional G-banding karyotyping revealed a de novo translocation 46,X,t(X;1)(q21.1;p13.3). Microarray-based comparative genomic hybridization showed normal results. Shallow genome-wide mate-pair library sequencing was applied to identify the areas where the breakpoints were present. The breakpoint on chromosome X disrupted the 1st intron of ATP7A gene, while the breakpoint on chromosome 1 was located in long non-coding RNA LINC01676.

The phenotypic and genetic findings in our patient will be compared with those in previously reported females with MD to illustrate the variability and severity of the clinical manifestations in females with MD. Presumably the occurrence of X-linked recessive MD in the female might be explained by the skewed X inactivation forced by the selection against active derivative X chromosome.

This study was supported by the NCN Grant No. UMO-2016/21/B/NZ5/02541.

#### 1.P38 Exonic deletion of CUL4B detected with comparative genomic hybridization

##### Alberto Plaja^1^, Joan Colomer^1^, Neus Castells^1^, Irene Valenzuela^1^, Anna Maria Cueto^1^, Ivón Cuscó^1^, Ida Paramanov^1^, Encarnación Oliveros^1^, Maria Serrano^1^, Desirée Martínez^1^, M Guadalupe Gala^1^, Clara Serra^1^, Anna Abulí^1^, Elena Garcia-Arumí^1^, Urdcat Consortium^2^, Eduardo Tizzano^1^

###### ^1^Hospital Vall D'hebron, Área De Genética Clínica Y Molecular, Barcelona-Spain; ^2^Generalitat De Catalunya, Departament De Salut, Barcelona-Spain

####### **Correspondence:** Alberto Plaja (aplaja@gmail.com)

INTRODUCTION: CUL4B (MIM #300304) is one of the most commonly mutated genes with loss-of-function variants in patients with intelectual disability (ID), regardless of their mode of inheritance, with reported frequencies 0.9 to 3 %. The overlapping phenotype in patients with CUL4B variants consists of ID, seizures, tremors, gait abnormalities, behavioral problems, macrocephaly, short stature, obesity, hypogonadotrophic hypogonadism and variable dysmorphic features. Variants of CUL4B are known to be the cause of Cabezas syndrome (MIM #300354). Very few deletions within or around CUL4B have been observed as disease-causing alterations.

SUBJECTS: Here we describe a phenotypically normal mother and her two affected sons with severe intellectual disability and speech delay, macrocephaly, facial dysmorphic traits, abdominal obesity, a behavioral profile including hyperactivity and aggressive outbursts.

METHODS AND RESULTS: A first comparative genomic hybridization assay using the Agilent ISCA 8x60K v2 array did not detect pathogenic copy number anomalies. A second genomic hybridization assay using the Oxford Gene Technology CytoSure Constitutional 8 × 60K v3 with enhanced exon-level CNV coverage of 354 developmental disorder genes revealed an hemizygous deletion reported as arr[hg19]Xq24(119691862_119693931)x0 in both affected brothers and a heterozygous deletion in their normal mother. Deletion breakpoints given by array CGH analysis involve only exon 4 (NM_003588). Breakpoints were redefined at the sequence level as chrX:119691734-119693999 using TruSightOne (Illumina) and SeqCap EZ MedExome v2 (Roche) assays. Thus, the deletion involves the whole exon 4 and a small portion of exon 5 (NM_003588), and changes the reading frame producing a new stop codon that reduces the original 913 aa protein in a shorter form of 184 aa.

CONCLUSION: Array comparative genomic hybridization assay with enhanced exon-level CNV coverage provides a robust, cost effective assay in intellectual disability. However, NGS based methods are required for accurate definition of breakpoints.

(PERIS SLT002/16/00174)

#### 1.P39 A complex chromosome 7p 7q rearrangement in a girl with Silver Russell like phenotype

##### Ilaria Catusi^1^, Maria Paola Recalcati^1^, Maria Teresa Bonati^2^, Eleonora Orlandini^2^, Lidia Larizza^1^, Daniela Giardino^1^

###### ^1^Istituto Auxologico Italiano, Irccs, Laboratorio Di Citogenetica Medica, Cusano Milanino-Italy; ^2^Istituto Auxologico Italiano, Irccs, Ambulatorio Di Genetica Medica, Milano-Italy

####### **Correspondence:** Maria Paola Recalcati (p.recalcati@auxologico.it)

Maternal uniparental disomy of chromosome 7 is present in 5–10% of patients with Silver-Russell syndrome (SRS), and duplication of 7p including GRB10 (Growth Factor Receptor-Bound Protein 10), an imprinted gene with effects including pre-and postnatal growth retardation, has been associated with SRS-like phenotype.

Here we report a 17 year old girl referred for array-CGH analysis for SRS-like clinical features including growth and psychomotor delay, relative macrocephaly and mild dysmorphisms.

Array-CGH analysis showed two copy number variants (CNVs): a ~9 Mb loss in 7q11.21-q11.23 and a ~12,7 Mb gain in 7p13-p11.2, involving GRB10. No copy number variation characterizes the pericentromeric region of ~6,6 Mb located between the two CNVs.

CGH+SNP-array analysis demonstrated that CNVs occurred on the maternal chromosome 7 and defined the 7p13-p11.2 duplication as maternal heterodisomy. FISH analysis on the proband establishes that the duplicated region maps on 7q with an inverted orientation and the normal copy number pericentromeric region is inverted too. Interestingly, FISH experiments performed on the mother showed a pericentric inversion of ~28.3Mb that might have mediated the complex rearrangement found in her daughter.

Chromosome 7 is highly enriched in segmental duplications some of which localized at the breakpoints of both the mother’s inversion and the daughter’s CNVs. Although unbalanced gametes are thought to occur rarely in carriers of pericentric inversions shorter than 50 Mb, as it is the case of the mother of our index case, pairing of highly homologous sequences might have perturbed the correct meiotic chromosome segregation leading to an unbalanced outcome.

The putative meiotic mechanism causative of the proband rearrangement is outlined and the phenotype of the proband is compared to those of patients with similar CNVs supporting the presence in 7p of a locus associated with the SRS-like phenotype.

Written, informed consent for publication was obtained from the patient [or parent/guardian for patients under 16]

#### 1.P40 Characterization and meiotic segregation of a supernumerary marker chromosome in the sperm of a phenotypically normal male

##### Zhanna Markova, Anastasia Tarlicheva, Marina Minzhenkova, Nadezda Shilova

###### Research Centre for Medical Genetics, Laboratory of Cytogenetic, Moscow-Russia

####### **Correspondence:** Zhanna Markova (zhmark71@mail.ru)

Small supernumerary marker chromosomes (SMCs) are structurally abnormal chromosomes that occur in the population of phenotypically normal subjects with the frequency of 0.043%. In patients with fertility problems the SMCs rate increases to 0.17%. We report a 35-year old phenotypically normal man, referred for pre-conceptional counseling after his wife had experienced three miscarriages. A SMC was found in 30% of the proband’s metaphases by conventional karyotyping. FISH analyses revealed a heterochromatic origin of the dicentric SMC derived from chromosome 22 - inv dup(22)(q11.1). The SMC was found also in 42% of the buccal smear cells. For the analysis of meiotic segregation of SMC inv dup(22) four-color FISH with a combination of different DNA-probes (WCP22, MD DiGeorge T-box1 (22q11) / 22q13 (SHANK3), SE 18- D18Z1) was performed. A total 1400 sperm cells were analyzed. The frequency of unbalanced spermatozoa, showing one chromosome 22 and a SMC, was 27.5%, one chromosome 22 and two SMCs - 0.6%. The level of disomy for chromosome 22 and 18 was 0.7% and 0.2%, respectively. Interchromosomal effect for chromosomes 13 and 21 was not found by FISH analysis of 1000 spermatozoa (frequency of spermatozoa with aneuploidy was 0.1% and 0.31% respectively). Thus, an increased level of aneuploidy was detected only for chromosome 22. In cases of previous recurrent abortions in combination with a sSMC pre-implantation genetic diagnosis (PGD) can be considered to reduce the risk of early fetal loss following chromosome imbalance. Since only normal chromosomal embryos are transferred to the uterus, the risk of losing the first and second trimester is reduced.

Prior to this study, the patient was informed of the investigations and gave his consent.

Written, informed consent for publication was obtained from the patient [or parent/guardian for patients under 16]

#### 1.P41 Report of two siblings carriers of the same rare chromosomal aberration

##### Nikoletta Selenti^1^, Eirini Tsoutsou^1^, Maria Tzetis^1^, Amenta Stella^1^, Voula Velissariou^2^, Elena Fryssira^1^

###### ^1^Department of Medical Genetics, School of Medicine, aghia Sophia” Children’s Hospital, Athens, Greece, Medical Genetics, Athens-Greece; ^2^Bioiatriki, Cytogenetics and Molecular Cytogenetics, Athens-Greece

####### **Correspondence:** Nikoletta Selenti (nikoletta_selenti@hotmail.com)

Introduction: Although distal deletion of 5p and partial duplication of 21q are two well characterized chromosomal abnormalities, the coexistence of both is very rare. It may occur in various forms and its clinical phenotypes are heterogeneous depending on the location and the accurate size of the aberrations.

Material and Methods: We present two female siblings with partial deletion of 5p and partial duplication of 21q.

Case 1, is an 11 years old female, the first child of two healthy unrelated parents. She presented with moderate mental retardation, hypotonia and morphological features such as downslanding palpebral fissures, hypertelorism, epicanthus, broad base to nose, micrognathia, small chin and low set ears. At birth, she was diagnosed with ventricular septal defect (VSD).

Case 2, is an 18 months old female, the second child of the family. Her phenotype was like her sister’s including hypotonia, psychomotor retardation, VSD and severe feeding difficulties. She was fed through nasogastric tube. Brain MRI was normal. Head circumference at the 10th centile.

Results: In both children conventional karyotype was: 46,XX, der(5)t(5;21)(p15.1;q22.1). Further parental cytogenetic analysis revealed that the mother was a carrier of the translocation t(5;21)(p15.1;q22.1)[46,XX,t(5;21)(p15.1;q22.1)], while the father had a normal karyotype, 46,XY.

Conclusion:Timely diagnosis of parental balanced chromosomal rearrangements can reduce the risk of subsequent miscarriages as well as abnormal offspring.

Written, informed consent for publication was obtained from the patient [or parent/guardian for patients under 16]

#### 1.P42 DSD XY and Wolf Hirschhorn syndrome a genotype phenotype correlation and a candidate gene

##### Khouloud Rjiba^1^, Olfa Kraiem^2^, Hédia Ayech^3^, Imen Ben Hadj Hmida^1^, Afef Jelloul^1^, Nabiha Mahdhaoui^3^, Ali Saad^1^, Soumaya Mougou-Zerelli^1^

###### ^1^Farhat Hached University Teaching Hospital, Laboratory of Human Cytogenetics, Molecular Genetics And Biology of Reproduction, Sousse-Tunisia; ^2^Regional Hospital, Kairouan, Pediatric Department, Kairouan-Tunisia; ^3^Farhat Hached University Teaching Hospital, Pediatric Department, Sousse-Tunisia

####### **Correspondence:** Khouloud Rjiba (khouloud.rjiba@yahoo.fr)

Wolf-Hirschhorn (WHS) is a set of congenital physical anomalies and a mental retardation associated with a partial deletion of the short arm of chromosome 4. To establish a genotype-phenotype correlation, we carried out a molecular cytogenetic analysis on two Tunisian WHS patients. Patient 1 was a one-year-old boy who presented a typical WHS phenotype, while patient 2, a boy of two days, in addition to the typical WHS phenotype, presented hypospadia and micropenis. Both the comparative genomic hybridization (CGH array) and Fluorescence in Situ Hybridization techniques (FISH) were used. Results of the analysis showed that patient 2 had a greater deletion size of chromosome 4 than patient 1. Size deletions were 4.8 Mb and 3.4 Mb, respectively. First of all, these results suggest that the deletion size could have a direct impact on the number of genes involved and then on the severity of the WHS phenotypic presentation. Second, if we analyze the uncommon deleted region between patient 1 and patient 2 we found that the MSX1 gene encompasses this region. It is a critical transcriptional repressor factor, expressed in the ventral side of the developing anterior pituitary and implicated in gonadotrope differentiation. Msx1 acts as a negative regulator in pituitary development by repressing the gonadotrope-specific GnRHR genes during embryogenesis. We hypothesized that the deletion of MSX1 in our patient may deregulate the androgen synthesis, resulting in higher concentrations of highly active androgens and thereby causing hypospadias during a critical phase of embryogenesis. Based on MSX1 gene function, its absence might be indirectly responsible for the hypospadia phenotype by contributing to spatiotemporal regulation of GnRH transcription during development.

Written, informed consent for publication was obtained from the patient [or parent/guardian for patients under 16]

#### 1.P43 Cytogenomic and clinical profile of balanced sex chromosome autosome translocations

##### Vivi. M. Srivastava^1^, Bidish Patel^1^, Vandana Kamath^1^, Mary P. Chacko^1^, Renu E. George^2^, Annie Regi^3^, Beena Koshy^4^, Sumita Danda^5^

###### ^1^Christian Medical College, Cytogenetics, Vellore-India; ^2^Christian Medical College, Dermatology, Vellore-India; ^3^Christian Medical College, Obstetrics, Vellore-India; ^4^Christian Medical College, Developmental Paediatrics, Vellore-India; ^5^Christian Medical College, Clinical Genetics, Vellore-India

####### **Correspondence:** Vivi. M. Srivastava (vivi@cmcvellore.ac.in)

We report the peripheral blood karyotypes and clinical presentations of nine individuals with balanced translocations of sex chromosomes and autosomes seen over a 17-year period (2001 to 2018). At least 25 G-banded metaphases were analysed from each sample.

There were eight t(X;autosome) and one t(Y;autosome). Breakpoints on chromosome X were varied and extended from p11.3 to q23; the autosomal regions involved were 4q31.1, 6q25, 7p21, 7q32, 13p11.2 , 13q13, 14q11.2, 16p13.3, and 17q24. The t(Y;autosome ) involved Yp11.2. The translocations seen were : 46,X,t(X;4)(p11.2;q31.1), 46,X,t(X;6)(q13;q25), 46,X,t(X;7)(q22;q21), 46,X,t(X;13)(q21;p11.2), 46,X,t(X;13)(p22.1;q13),46,X,t(X;14)(q13;q11.2), 46,X,t(X;17)(q23;q24), 46,Y,t(X;16)(p11.3;p13.3), and 46,X, t(Y;7)(p11.2;p21).

Our nine patients ranged from 1-31 years of age. Seven of the t(X;autosome) were females and included four adults aged 18-29 years with primary amenorrhea / premature ovarian failure / infertility. The three girls with t(X;autosome) were aged 1-2 years and presented with developmental delay and dysmorphism / epilepsy; one girl also had features of Menke’s kinky hair syndrome. Both males, one with t(X;16) and one with t(Y;7) had infertility with severe oligospermia / azoospermia.

None of our patients showed involvement of chromosomes 15, 21 or 22, which are reported to be most predisposed to translocations with chromosome X. Chromosomes 7 and 13 were each involved in two patients, one of whom had the t(Y;7).

This analysis illustrates the varied effects of these rare translocations, namely, a normal phenotype with ovarian dysfunction / infertility / azoospermia ; an abnormal phenotype with developmental delay ; manifestation of an X-linked recessive disorder in a female (Menke’s kinky hair syndrome). Cytogenomic analysis is important to identify these translocations so that appropriate counselling maybe provided.

#### 1.P44 The use of chromosomal microarray analysis in patients with possible genetic epilepsy

##### Ilya Kanivets^1^, Irina Romanova^1^, Sergey Korostelev^1^, Artem Sharkov^2^, Denis Pyankov^3^

###### ^1^Genomed Ltd., Genetics, Moscow-Russia; ^2^Genomed Ltd., Neurology, Moscow-Russia; ^3^Genomed Ltd., Laboratory, Moscow-Russia

####### **Correspondence:** Ilya Kanivets (dr.kanivets@genomed.ru)

Chromosomal microarray analysis (CMA) is widely used as a first-tier test for patients with congenital malformations and developmental delay. The aim of our study was evaluate the prevalence of clinically significant copy number variations (CNVs) in patients with epilepsy. We used Cytoscan HD, Cytoscan 750K and Cytoscan Optima (Thermo Fisher Scientific, USA) SNP-oligonucleotide arrays for CNVs and contiguous absence of heterozygosity loci (AOH) detection. Interpretation of clinical importance of CNVs was based on patient’s medical records and ACMG Standards and Guidelines for constitutional cytogenomic microarray analysis. The CMA was performed for 844 patients with epilepsy. Pathogenic CNVs described as a syndrome in the OMIM database were found in 8.6% (73 patients). Pathogenic deletions (25 cases) and duplications (9 cases) of a single chromosome not annotated in OMIM were found in 4% (34 patients). Chromosomal abnormality of two non-homologous chromosomes were detected in 2.1% (18 patients). Contiguous AOH loci were found in 3.4% (29 patients). Further evaluation was recommended for 8 of these patients, because they have epilepsy-associated genes in AOH region. 76 of 132 patients with disease-causing CNVs have well described clinical history. Congenital brain malformations were described in 28 patients, cardiovascular and genitourinary malformations described in 12 and 10 patients respectively. CMA in patients with epilepsy was able to detect disease-causing CNVs in 15.6% (132 patients). This test should be recommended as the first-tier test for patients with epilepsy and congenital malformations, predominantly of the brain and heart.

#### 1.P45 Array CGH and FISH characterization of six new cases of Small Supernumerary Marker Chromosomes encompassing the 15q11q13 region

##### Wafa Slimani^1^, Molka Kammoun^1^, Ahlem Atig^1^, Soussen Meddeb^2^, Jihen Mathlouthi^3^, Damien Sanlaville^4^, Thomas Liehr^5^, Ali Saad^1^, Soumaya Mougou-Zerelli^1^

###### ^1^Farhat Hached University Teaching Hospital, Department of Human Cytogenetics, Molecular Genetics And Biology of Reproduction, Sousse-Tunisia; ^2^Farhat Hached University Teaching Hospital, Department of Gynecology And Obstetrics, Sousse-Tunisia; ^3^Farhat Hached University Teaching Hospital, Department of Pediatrics, Sousse-Tunisia; ^4^University Hospital of Lyon, Department of Genetics, Lyon-France; ^5^Jena University Hospital, Friedrich Schiller University, Institute of Human Genetics, Jena-Germany

####### **Correspondence:** Wafa Slimani (slimani_wafe@yahoo.com)

Supernumerary marker chromosomes (SMC) are additional abnormal chromosomes that can be derived from any chromosome. However, more than 50% of SMC are derived from acrocentric chromosomes, mainly chromosome 15.

The aim of this study was to characterize six SMC detected in six children presenting heterogeneous phenotypes ranging from mild to severe intellectual disability and/or developmental delay and dysmorphic features. Autism spectrum disorder, seizure and speech delay were noted in two patients.

R-Banding karyotypes of patients and their parents showed a de novo SMC 15 in all cases except for one where the marker was maternally inherited, and one was mosaic. In situ fluorescence hybridization (FISH) analysis using the SNRPN, GABRB3, IGF1R, Midi54, Subcentromere 15q, Centromere 15 and Whole chromosome painting 15 probes allowed us to determine the shape of these SMCs and to delimit approximately the breakpoints. The study of the extent of the duplicated region by array CGH allowed to identify the exact segmental gains and to precisely delimit the breakpoints.

In four cases, the SMC was of inverted duplicated shape, encompassing the Prader-Willi/Angelman critical region (PW/ASCR). In two cases, the SMC was of smaller size and did not contain the PW/ASCR. A disproportional subset of SMCs are derived from chromosome 15 conferring a trisomy or tetrasomy of the 15q11-q13 region. In fact, this region is prone to unequal crossover responsible for the variable size of chromosomal rearrangements due to the existence of five known breakpoints. It is also a parentally imprinted region that undergoes differential phenotypic expression depending on the parental origin.

The presence of breakpoints, parental imprinting and PW/ASCR, the content of euchromatic material and mosaicism are responsible for the morphological and clinical heterogeneity of the SMC15.

#### 1.P46 A ring 22 resulted from an atypical inv dup del 22 lessons from SNP array analysis

##### Myriam Rachid^1^, Jonathan Levy^2^, Damien Haye^2^, Sandrine Passemard^2^, Hala Nasser^2^, Anna Maruani^3^, Frederique Amsellem^3^, Céline Dupont^2^, Edwina Thimodent^2^, Thomas Bourgeron^4^, Richard Delorme^3^, Anne-Claude Tabet^1^

###### ^1^Robert Debré Hospital, Aphp And Institut Pasteur, Genetics Department and Human Genetics And Cognitive Functions, Paris-France; ^2^Robert Debré Hospital, Aphp, Genetics Department, Paris-France; ^3^Robert Debré Hospital, Aphp And Institut Pasteur, Department of Child and Adolescent Psychiatry and Human Genetics and Cognitive Functions, Paris-France; ^4^Institut Pasteur, Human Genetics and Cognitive Functions, Paris-France

####### **Correspondence:** Anne-Claude Tabet (anne-claude.tabet@aphp.fr)

Phelan-McDermid syndrome (PMS) is a neurodevelopmental disorder characterized by intellectual disability, delayed or absent speech, autism and variable comorbidities. PMS is mostly caused by SHANK3 (at 22q13.3) deletion or truncated mutations. SHANK3 deletions occur de novo or result from unbalanced translocation. Less frequently, PMS was associated with a ring 22 or more complex chromosomal rearrangements in severely affected patients.

We reported on two patients referred for intellectual disability and autism. Both carried a terminal 22q13.3 deletion encompassing SHANK3 associated with an adjacent 22q13 gain of copy identified by SNP-array. In the first case, the rearrangement was concordant with an inv dup del resulted from a U-type exchange mechanism leading to a de novo 1.4 Mb terminal deletion and an adjacent 22q13 duplication of 7.8 Mb.

In the second case, SNP array revealed a complex profile with a terminal 22q13.3 homogenous deletion (7.2 Mb) and an adjacent complex gain of copy consisting in a mosaic triplicated segment and a proximal homogenous duplicated segment. Standard karyotype and FISH analysis identified two r(22) of different size in distinct cellular populations.

We suggested an atypical U-type exchange leading to different ring chromosomes (RC) depending on the breakpoints of the intermediate dicentric chromosome as a mechanism of formation for this rearrangement.

We showed that depending on the size of the terminal deletion, the telomere capture mechanism that stabilized the chromosome, could be involved in a large proportion of RC of which the yields and the complex molecular nature is probably underestimated.

Regarding the phenotype, concomitant interstitial duplication and terminal deletion, or complex rearrangements could contribute to the severity of PMS observed in r(22).

Finally, SNP-array improves the molecular characterization of complex chromosomal rearrangements.

#### 1.P47 Family studies of two complex chromosomal translocations

##### Iren Haltrich^1^, Anna Lengyel^1^, Eva Pinti^1^, Karin Nebral^2^, Henriett Piko^3^, Gyorgy Fekete^1^, Oskar A Haas^2^

###### ^1^Semmelweis University, 2nd Department of Pediatrics, Budapest-Hungary; ^2^Children’s Cancer, Research Institute (ccri), Department of Clinical Genetics, Vienna-Austria; ^3^Semmelweis University, 1st Department of Medicine, Budapest-Hungary

####### **Correspondence:** Iren Haltrich (haltrich.iren@med.semmelweis-univ.hu)

Complex chromosomal translocations (CCRs) are structural rearrangements involving three or more chromosomes and breakpoints. We present two unique cases of CCRs in two different families. Proband 1: A 3-month-old girl was referred to our Cytogenetic Laboratory for dysmorphism (high and large forehead, bitemporal constriction, round and flat face, fine and arched eyebrows, broad nasal bridge, low set ears, short neck), severe neonatal hypotonia, congenital heart disease (ASD, VSD), macroglossia, widespread naevus flammeus and palpebral ptosis. The G-banded karyotype and FISH examination revealed additional material originated from 10q on the chromosome 8 short arm. Examination of parents and grandparents identified a de novo CCR in the healthy mother: 46,XX,t(21;11;10;8)(q22;q23;q25;p23)dn. The kayotype of Proband 1 was defined as: 46,XX,der(8)t(8;10)(p23;q25)mat. Her phenotype overlapped with the severe manifestation of 10q trisomy syndrome: cardiac involvement, severe kyphoscoliosis, dilatation of ventriculi and intellectual disability. In a subsequent pregnancy the amniotic cells presented the karyotypic pattern of the mother. Array-CGH excluded any related chromosome imbalances.

Proband 2: The 4-year-old boy presented a Prader-Willi-like phenotype (neonatal hypotonia, hyperphagia, overweight), speech delay, severe intellectual disability and psychiatric/behavioral features. The patient’s karyotype showed a CCR of chromosomes 1, 2, 4, 5, 10 and 17; FISH analysis of the relevant chromosomes with whole chromosome painting probes did not reveal any imbalances. Array-CGH identified a 1,4 Mb heterozygous deletion including exon 1 of CNTNAP5 (NM_130773) (5‘ deletion): arr[hg19] 2q14.3(123,431,180-124,854,926)x1. Parental karyotypes were normal. The CNTNAP5 belongs to the neurexin family, which plays a role in the correct development and proper functioning of the nervous system, cell adhesion and intercellular communication. The few previously described patients with deletions of this gene exhibited learning disability-, and language impairment. New data suggest that genomic disruption of CNTNAP5 gene may confer autism spectrum disorder.

#### 1.P48 A case of maternal UPD20 detected by routine SNP array diagnostics

##### Blom E., Saris J. J., Van Zutven L. J. C. M., Hoefsloot E. H.

###### Erasmus Medical Center, Clinical Genetics, Rotterdam-The Netherlands

####### **Correspondence:** Blom E. (e.blom@erasmusmc.nl)

In our laboratory we perform routine SNP array analysis for several clinical phenotypes, including intellectual disability, congenital abnormalities, and prenatal indications. Although this technique is not primarily aimed for UPD (Uniparental Disomy) diagnostics, a patient with a maternal UPD of chromosome 20 was discovered. UPD as a consequence of heterodisomy is not detectable by SNP array analysis. However, the presence of (large) homozygous regions on a single chromosome can be suggestive for an isodisomy UPD, which can be further tested for with another technique.

Here, we present a case of an infant with severe feeding difficulties needing gastric-tube feeding. Routine SNP array analysis did not show any particularities, except two terminal regions of homozygosity, at 20p and 20q, which is suggestive for UPD. To confirm the possible presence of UPD, we requested parental samples and performed a SNP trio-analysis. SNP trio-analysis indeed confirmed the presence of UPD20, which showed of a maternal origin. Maternal UPD20, or upd(20)mat syndrome, is a very rare condition for which only a very limited number of cases are described in literature. The main symptoms of this disorder are prenatal and postnatal growth retardation and (severe) feeding difficulties requiring gastric-tube feeding in the first years of life. Literature of the upd(20)mat syndrome and mechanisms leading to UPD formation are reviewed.

#### 1.P49 The first case of 3p26.3 deletion containing only CHL1 gene associated with ASD

##### Andreea Cristina Stanciu^1^, Ioana Streata^2^, Mihai Ioana^2^, Ina Focsa^3^, Magdalena Budisteanu^4^

###### ^1^'carol Davila' University of Medicine And Pharmacy, Faculty of Medicine, Bucharest-Romania; ^2^University of Medicine and Pharmacy Craiova, Human Genomics Laboratory, Craiova-Romania; ^3^'carol Davila' University of Medicine and Pharmacy, Department of Genetics, Bucharest-Romania; ^4^'victor Babes' National Institute of Pathology; 'prof. Dr. Alex. Obregia' Clinical Hospital of Psychiatry; 'titu Maiorescu' University, Head of Psychiatry Research Laboratory 'prof. Dr. Alexandru Obregia' Clinical Hospital of Psychiatry, Bucharest-Romania

####### **Correspondence:** Andreea Cristina Stanciu (stanciuandreeacristina@gmail.com)

Background: The 3p deletion syndrome is a rare contiguous gene disorder caused by deletions in the 3p25-pter region. It is characterized by growth and mental retardation, developmental delay, dysmorphisms, microcephaly and ptosis. It was suggested that a deletion of a critical region spanning two genes, CRBN and CNTN4, is sufficient to cause the typical clinical features. CHL1 is located distally to CRBN and CNTN4, a gene highly expressed in the brain that plays an important role in neuronal migration, neurite overgrowth and regulation of synapse function. Loss of the CHL1 gene was associated with cognitive impairment. To date only four well documented familial cases presenting a terminal deletion (500 Kb-1.1 Mb) at 3p26.3 including only CHL1 gene, have been reported. All the patients presented a common atypical phenotype for the 3p-syndrome with poor verbal function and developmental delay. It was suggested that this could be good evidence of a new emerging syndrome associated with the deletion of CHL1 gene alone.

Methods and Results: We describe a 12-year old boy in whom array-CGH analysis disclosed a submicroscopic 3p26.3 deletion of 12 Kb spanning only a part of CHL1 gene. Our patient presented mild intellectual disability, speech delay, motor delay and hyperkinesia. The personal history revealed autism spectrum disorder (ASD) and epileptic seizures.

Conclusion: In all five documented cases, including the present case, the phenotype is similarly marked by verbal function and developmental delay, further supporting the possibility of a new syndrome associated with a deletion spanning the CHL1 gene. However, phenotypically this is the first case associated with ASD and genotypically this is the first case with a deletion of such a small size. Further work is necessary to more clearly delineate genotype-phenotype relationships in 3p26.3 deletions.

Written, informed consent for publication was obtained from the patient [or parent/guardian for patients under 16]

#### 1.P50 Intertissue variability of gonosomal mosaicism in patients with sex chromosome abnormalities and DSD

##### Natalia Oparina^1^, Svetlana Kalinenkova^1^, Valentina Antonenko^1^, Arthur Latypov^1^, Nadezhda Raygorodskaya^2^, Zhanna Markova^3^, Marina Minzhenkova^3^, Vyacheslav Chernykh^4^

###### ^1^Moscow Regional Research Clinical Institution, Medical Genetic Centre, Moscow-Russia; ^2^Saratov State Medical University, Endocrinology, Saratov-Russia; ^3^Research Centre for Medical Genetics, Cytogenetics, Moscow-Russia; ^4^Research Centre for Medical Genetics, Genetics of Reproduction Disoredrs, Moscow-Russia

####### **Correspondence:** Vyacheslav Chernykh (chernykh@med-gen.ru)

Background: Gonosomal mosaicism is common for sex chromosome aneuploidies and structural abnormalities, and often results in Disorders of Sex Development (DSD), sub-/infertility. The phenotypes depend on the types and quantitative distribution of cell lines, but inter-tissue differences of mosaicism and geno-phenotypic correlations are not sufficiently studied.

Materials and methods: We examined 156 patients with sex chromosome abnormalities and/or DSD, including Klinefelter (KS, n=40) and Turner syndrome (TS, n=42) patients, trisomy X (n=8), polysomy Y (n=2), 46,XX-testicular DSD (n=6), XX and XY gonadal dysgenesis/ovotesticular DSD (n=25), XX/XY (n=6) and non-TS X/XY (n=27) mosaics. Chromosome analysis was done on cultured peripheral blood lymphocytes using the GTG-staining. FISH with the DNA probes (DXZ1, DYZ3, DYZ1, LSI SRY) was performed on peripheral blood lymphocytes, buccal smear cells, cultured skin fibroblasts and gonadal biopsies. At least 100 cells were analyzed for each analysis. Multiplex PCR was done for SRY, AMELX/Y loci and AZFa,b,c region markers.

Results: In KS patients, gonosomal mosaicism was found in cases with mosaicism in leucocytes. Y polysomic clone was major in both patients with tri-/tetrasomy Y (57-60%). Trisomy X was mosaic in 50% patients. Complex dynamic Y chromosome mosaicism was revealed in 10% X/XY mosaics. Terminal Yq11.2 deletions and AZF microdeletions were detected in 60% and 17% X/XY mosaics, respectively. XX/XXY patients presented mosaicism with 2-95% Y+ cells, more in buccal cells or other tissue than in the lymphocytes. Two of them were chimeras because of haemopoetic cells transplantation; at least two XX/XY patients are true chimeras. Prominent discrepancy of cell lines distribution between lymphocytes and other tissues was found in 2.5% KS, 12.5% trisomy X, 25% TS patients, 20% X/XY and 30% XX/XY mosaics. One SRY+ 46,XY DSD patient with ambiguous genitalia presented 45,X cells only in genitals. Mosaic X-derivate chromosome was detected in two of five SRY+XX-sex reversed patients and one female patient with X;X translocation.

Conclusion:

Gonosomal mosaicism is still under evaluated. The proportion of the cell lines can widely varies from one tissue to another in the same patient, especially in TS, X/XY and XX/XY individuals, including cryptic mosaicism. The analysis of only 1-2 tissues in DSD patients is insufficient, especially in patients with different distribution of clones in different tissues.

#### 1.P51 Clinical utility of molecular karyotyping in patients with unexplained developmental delay intellectual disability without additional congenital anomalies

##### Luca Lovrecic, Nuša Trošt, Borut Peterlin

###### University Medical Center Ljubljana, Clinical Institute of Medical Genetics, Ljubljana-Slovenia

####### **Correspondence:** Luca Lovrecic (lucalovrecic@gmail.com)

Copy number variation analysis using array based comparative genomic hybridization (aCGH) has been acknowledged as a first-tier genetic diagnostic test in patients with developmental delay (DD)/ intellectual disability (ID) and congenital anomalies since 10 years and numerous studies report on a diagnostic yield between 5-30%, depending on inclusion criteria. Nevertheless, the data on cohorts of patients with unexplained DD/ID without any major congenital anomalies are lacking.

Our aim was to evaluate the clinical utility of aCGH in a group of 731 patients referred for genetic testing due to the unexplained DD/ID in the years between 2012 and 2018. There were 475 male and 256 female patients with the average age of 6.9±6.3 years. The clinical phenotype included developmental delay and/or intellectual disability, in some cases associated with minor dysmorphic features and autism spectrum disorder. No patients with congenital anomalies or other significant phenotype abnormalities were included (such as epilepsy, hearing loss). The study revealed a pathogenic or likely pathogenic CNV in 130 cases, giving a diagnostic yield of 17.8%. In this group, 103 CNVs represented known microdeletion/microduplication syndromes, some less common pathogenic CNVs and individual, previously unreported pathogenic chromosomal rearrangements, representing new genotype-phenotype correlations. The remaining 27 CNVs represented a risk factor linked to neurodevelopmental disorders, such as 16p11.2 deletion or 1q21.1q21.2 deletion. Variants of unknown significance were identified in 52 patients (7.1%). In 6 additional individuals (0.8%) molecular karyotyping identified a secondary finding, not related to the patient’s phenotype but important for proband's future health or for other family members.

The present study demonstrates the clinical utility of aCGH in the genetic work-up of patients with unexplained DD/ID without any congenital anomalies. In addition to a significant diagnostic yield (17.8%)and identification of known causal CNVs, this approach still deciphers new genotype-phenotype correlations.

#### 1.P52 Late diagnosis of Edwards’ syndrome Case report

##### Suely Santos, Mariana De Souza

###### Unirio, Dgbm, Rio De Janeiro-Brazil

####### **Correspondence:** Suely Santos (surodosan@yahoo.com.br)

Introduction: Multiple congenital anomalies, short stature and mental retardation are characteristic of autosomal trisomies, such as, trisomy 18 or Edwards syndrome. While mosaic cases may be more mildly affected than nonmosaic ones, we cannot relate the degree of the defect with the proportion of abnormal cells in any one tissue; also, mild clinical findings may be shared with non-chromosomal syndromes. The challenge is to know when to suspect a chromosomal anomaly. Case report: A fifteen year old girl, 3rd child from a first grade consanguineous couple, was born from a cesarean delivery, preterm with 1800g and neonatal hypoxia. She showed with developmental delay, mild mental retardation, learning disabilities, short stature , syndactyly on 2nd/3rd toes, camptodactyly on fingers and clinodactyly of the fifth right finger, normal pubertal development, corneal opacification in the right eye, no malformation. Systemic arterial hypertension (SAH) and tachycardia were observed during tonsillectomy and adenoidectomy surgery. SAH Investigation did not detect abnormalities. Familial history of SAH was positive and at first glance, a recessive autosomal condition was suspected, including adrenal synthesis defect, but hormonal evaluation was normal. Based on the presence of mental retardation, short stature and dysmorphisms, a karyotype was made and the result was 47,XX, +18 [2]/46,XX [18]. Discussion: Despite the importance of the technological advances in diagnosing previously unknown structural chromosomal syndromes, classical cytogenetics still has its place in clarifying mild phenotype of numerical chromosomal anomalies as we found in this case of mosaicism. We emphasize the importance of the karyotype in patients with mental retardation and / or short stature, taking into account that this procedure is inexpensive, practical and available, especially, in developing countries.

Written, informed consent for publication was obtained from the patient [or parent/guardian for patients under 16]

#### 1.P53 Telomere dysfunction in patients undergoing fertility treatment

##### Radhia M'kacher^1^, Bruno Colicchio^2^, Valentine Marquet^3^, Wala Najar^4^, William M. Hempel^1^, Leonhard Heidingsfelder^5^, Patrice Carde^6^, Alain Dieterlen^2^, Eric Jeandidier^7^, Catherine Yardin^3^

###### ^1^Cell Environment, Oncology, Paris-France; ^2^Université De Haute-Alsace, Irimas, Mulhouse-France; ^3^Chu Dupuytren, Cytogénétique, Génétique Médicale, Et Biologie De La Reproduction, Limoges-France; ^4^Université Paris Descartes, Faculté De Médicine, Paris-France; ^5^Metasystems, Metasystems, Altlussheim-Germany; ^6^Gustave Roussy Cancer Campus, Hematology, Villejuif-France; ^7^Groupe Hospitalier De La Région De Mulhouse Et Sud Alsace Mulhouse, Genetic, Mulhouse-France

####### **Correspondence:** Radhia M'kacher (radhia.mkacher@cell-environment.com)

Purpose:

Telomeres, the protective ends of linear chromosomes, play a major role in the maintenance of genome integrity and stability. Telomere shortening is involved in natural aging and age-related disorders, such as cancer and cardiovascular disease. Mounting evidence in favour of the implication of telomere dysfunction in ovarian senescence as well as in sperm quality has emerged. This study aimed to assess the accuracy of “biological clock” concept in fertility complications.

Materials and methods:

Fifty patients (17F/33M; mean age 34 years (23 –54 years) undergoing fertility treatment and 100 healthy donors (40 years (23- 54 years) were included in this study. Telomere length and telomere aberration (loss of telomere and the formation of telomere doublet) were performed using Q-FISH technique. Chromosomal aberrations were detected using conventional cytogenetic and FISH techniques.

Results:

All patients showed significantly shorter telomeres in comparison to healthy-matched controls (5.9kb (4.8-6.9kb) for patients vs 8.7kb (5.2-13.9kb) for donors) (P<10-16). Significant high rates of telomere loss (p< 10-14) and telomere doublet formation (p<10-5) were found in patients compared to controls. Numerical and structural chromosomal aberrations were also observed in 50% of patients. No significant correlation was observed between the telomere dysfunction and the presence of chromosomal aberrations. Nevertheless, dicentric chromosomes were found in 9/25 patients with aberrations, and 85% of breakpoints were localized in peri-centromeric regions leading to pericentric inversions, iso-chromosomes and Robertsonian translocations.

Conclusion: Our findings demonstrate that telomere dysfunction represents the main common cytogenetic characteristic of patients undergoing fertility treatment. The relationship between telomere dysfunction and peri-centromeric breakpoints needs additional investigation. Telomere status could be a possible novel and useful tool as an outcome predictor for assisted reproduction.

#### 1.P54 Combination of Classical cytogenetics Spectral karyotyping and CMA for resolving three different cases

##### Ela Fliesmacher^1^, Galina Margolis^1^, Irit Bar-Am^2^, Nirit Katzin^2^, Esther Manor^3^

###### ^1^Soroka University Medical Center, Human Genetics, Beer-Sheva-Israel; ^2^Applied Spectral Imaging, Applied Spectral Imaging, Yokneam-Israel; ^3^Soroka University Medical Center, Ben-Gurion University In The Negev, Human Genetics, Beer-Sheva Israel

####### **Correspondence:** Ela Fliesmacher (manore@bgu.ac.il)

Here we describe three unique cases in which the use of a combination of two or three techniques was needed for a reliable result: Two couples admitted to our laboratory due to fertility failure and one case of a 23-year-old woman with premature amenorrhea and high FSH level. The first couple suffered from early loss of pregnancies and two pregnancy terminations due to holoprosencephaly. Cytogenetic analysis showed a normal karyotype for both wife and husband, CMA analysis of one of the aborted fetus showed duplication of about 14 mega bases of the 3p and deletion of about 10 mega base on 18p. SKY analysis resulted in a normal karyotype for the husband while the wife carries a balanced 3;18 translocation. The second couple showed a wife with normal karyotype while the husband carried an uncertain complex karyotype which needed a further evaluation by SKY. This revealed a balanced complex karyotype with a 11;22 translocation and ins(15;14)(q26.1;q22.1q24.3). Familial evaluation showed the same chromosomal changes in his brother while his mother carries only the balanced t(11;22), pointing to the conclusion that the dead father carried the insertion, ins(15;14)(q26.1;q22.1q24.3) . The combination of classic cytogenetics and SKY analysis was also needed in the third case. The complex karyotype was further examined by SKY analysis which revealed a translocation involving chromosomes 5, 9, 17 and X. The importance and the significance of the combination of techniques in the described cases will be discussed.

* Corresponding author: manore@bgu.ac.il

Written, informed consent for publication was obtained from the patient [or parent/guardian for patients under 16]

#### 1.P55 A MID1 gene deletion in an Albanian patient with X linked Opitz G BBB syndrome

##### Anila Babameto-Laku^1^, Dorina Roko^1^, Anila Mitre^1^, Flogerta Alia^1^, Chris Konialis^2^, Constantinos Pangalos^2^

###### ^1^University Hospital Center ”Mother Teresa”, Laboratory Genetics Service, Tirana-Albania; ^2^Diagnostic Genetic Center, Intergenetics, Athens-Greece

####### **Correspondence:** Anila Babameto-Laku (dorinaroko@yahoo.com)

Mutations in the MID1 gene in Xp22, result in X-linked Opitz G/BBB syndrome (XLOS), a disorder that affects development of midline structures and comprises hypertelorism, cleft lip/palate, hypospadias, and laryngo-tracheo-esophageal abnormalities. Also cardiac, anal and neurologic defects are present. MID1 gene abnormalities include missense, nonsense, and splicing mutations, small insertions, small deletions, and complex rearrangements. Deletions consist of either single exons or the entire MID1 coding region. We present a three month old boy with a pathogenic hemizygous 451 Kb microdeletion of Xp22.2 encompassing the entire gene MID1. The clinical features included hypertelorism, palpebral ptosis, especially on the left side, flat nasal bridge, congenital imperforate anus, hypospadias and perimembranous ventricular septal defect. The microarray-based comparative genomic hybridization revealed a pathogenic hemizygous 451 Kb microdeletion of Xp22.2 encompassing the entire gene MID1, arr(hg 19)Xp22.2(10,401,387-10,852,119)x0 (451) (OMIM genes : MID1). The whole genome microarray CGH (aCGH) analysis was performed in order to investigate the presence of copy number aberrations and to determine their possible association with pathological phenotypes. As the above microdeletion in this male child may have been inherited from a mother-carrier, genetic testing of the mother for the presence of the microdeletion was highly recommended prior to future pregnancies, in order to ascertain the recurrence risk. Conclusion. To our best knowledge, this is the first case report of X-linked Opitz G/BBB syndrome with MID1 gene deletion in Albania. Mutations and hemizygous deletions of the MID1 gene have been previously reported in the literature and international databases in male patients with X-linked Opitz G/BBB syndrome. But the variety of described mutations and the lack of a strict genotype-phenotype correlation suggested us to report the case.

#### 1.P56 Candidate genes’ panel for investigation of pathogenic allelic variants in patients with the 22q11.2 deletion syndrome

##### Natalia Nunes^1^, Anelisa Gollo Dantas^1^, Malú Zamariolli^1^, Diogo Cordeiro De Queiroz Soares^2^, Vera Ayres Meloni^1^, Chong Ae Kim^2^, Vera Lúcia Gil Da Silva Lopes^3^, Maria Isabel Melaragno^1^

###### ^1^Universidade Federal De São Paulo, Genetics Division, Department of Morphology And Genetics, São Paulo-Brazil; ^2^Universidade De São Paulo, Genetics Unit, São Paulo-Brazil; ^3^Universidade Estadual De Campinas, Department of Medical Genetics, Campinas-Brazil

####### **Correspondence:** Natalia Nunes (natrdn@gmail.com)

22q11.2 deletion syndrome (22q11.2DS) results from hemizygous deletions of chromosome 22, flanked by low copy repeats, which predisposes the chromosome to aberrant recombination. Even though most 22q11.2DS patients have the same size deletions of 3 Mb, the phenotype is highly variable among individuals. About 180 clinical manifestations have been described in the syndrome, such as congenital cardiac malformations, characteristic facial features, immune deficiency, and psychiatric disorders. Although the dose-sensitive genes mapped in the deleted region are the main candidates for the phenotype, it is suggested that more complex molecular mechanisms are involved in the etiology of the phenotypic variability of the syndrome. The presence of single nucleotide variants (SNVs) on the remaining allele of 22q11.2 or in other genes outside the 22q11.2 region is suggested to have a role in the phenotype variability observed. We studied 60 22q11.2DS patients with 3 Mb deletions and sequenced the coding regions of seven candidate genes located in and outside the 22q11.2 hemizygous region: CRKL, MAPK1, HIRA, TANGO2, PI4KA, ZFPM2, JAM3, using Ion Ampliseq methodology. We identified variants that have not been previously described in the literature and that may have a role as modifiers in the 22q11.2DS. This represents a great potential for exploration in later studies and contributions to the field after validation. The use of gene panels with candidate genes can foment the search for genetic modifiers and improve the treatment of patients with 22q11.2DS. Financial support: FAPESP, Brazil.

#### 1.P57 Genetic origin of congenital heart defects in Tunisian population about 66 cases

##### Leïla Dardour^1^, Afef Jelloul^2^, Jihene Mathlouthi^3^, Sarra Dimassi^2^, Karim Ben Ameur^4^, Houda Ajmi^5^, Samia Tilouche^5^, Najla Soyah^5^, Hayet Ben Hmida^4^, Khaled Ben Hlel^6^, Mohamed Taher Sfar^7^, Nabiha Mahdhaoui^3^, Ali Saad^2^, Soumaya Mougou^2^

###### ^1^University of Sousse, Department of Cytogenetics and Reproductive Biology & Department of Genetics, Faculty of Medicine, Sousse-Tunisia; ^2^University of Sousse, Department of Cytogenetics and Reproductive Biology, Sousse-Tunisia; ^3^University of Sousse, Department of Neonatology, Sousse-Tunisia; ^4^University of Monastir, Department of Neonatology, Monastir-Tunisia; ^5^University of Sousse, Department of Pediatrics, Sousse-Tunisia; ^6^University of Sousse, Department of Pediatrics and Neonatology, Kairouan-Tunisia; ^7^University of Monastir, Department of Pediatrics and Neonatology, Mahdia-Tunisia

####### **Correspondence:** Leïla Dardour (leiladardour.ferhi@gmail.com)

Congenital heart defects (CHD) are the most common type of birth defects with an incidence of 7 ‰ live births, and encompass a wide range of malformations. CHD account for nearly one-third of all major congenital anomalies, and represent a major cause of infant morbidity and mortality.

In Tunisia, the incidence of CHD is estimated to be 6.8 ‰. Using conventional and molecular cytogenetic techniques, we studied the genetic causes of CHD in Tunisian patients.

In a cohort of 66 patients with CHD, 45 patients had syndromic CHD. The most frequent CHD was ventricular septal defect (VSD) present in 25 patients, isolated or associated to another CHD. Atrial septal defect (ASD) and valvulopathies were equally found in 13 patients each, followed by coarctation of the aorta (6), atrioventricular canal defect (6), patent ductus arteriosus (5) and tetralogy of Fallot (4).

The genetic etiology was determined in 22 patients. Trisomy 21 was the most prevalent cause of CHD in our population (10 patients); most of them had septal defect. Williams-Beuren syndrome was diagnosed in 4 patients who had either pulmonary or aortic stenosis. Di-George Syndrome was found in 3 patients with VSD. Two girls were diagnosed with Turner syndrome; one had aortic stenosis and the other a VSD. Two other patients had Trisomy 18: one had a tetralogy of Fallot and the other a double outlet right ventricle. One patient with coarctation of the aorta was carrying a 9q34.3 deletion, which corresponds to a Kleefstra syndrome.

Syndromic CHD was present in 68% of our patients. Half of them were shown to carry chromosomal anomalies and pathogenic copy number variants (CNV). The unsolved CHD cases are likely to result from gene mutations or cryptic chromosomes rearrangements. The previously described CHD heterogeneity emphasis the importance of chromosome anomalies, CNV and candidate gene mutations in the CDH genetic diagnosis. This indicates the usefulness of high resolution techniques which make array CGH and whole genome sequencing attractive tools to study CHD. The latter techniques are discussed for the 44 unsolved CDH patients.

Written, informed consent for publication was obtained from the patient [or parent/guardian for patients under 16]

#### 1.P58 Different chromosomal aberrations for one entity 9p Duplication syndrome

##### Wafa Slimani^1^, Hela Ben Khelifa^1^, Hayet Ben Hmida^2^, Afef Jelloul^1^, Hanene Hannachi^1^, Rania Sakka^2^, Ali Saad^1^, Soumaya Mougou-Zerelli^1^

###### ^1^Farhat Hached University Teaching Hospital, Department of Human Cytogenetics, Molecular Genetics and Biology of Reproduction, Sousse-Tunisia; ^2^Centre De Maternité Et De Néonatologie De Monastir, Chu Fattouma Bourguiba, Department of Neonatology, Monastir-Tunisia

####### **Correspondence:** Soumaya Mougou-Zerelli (slimani_wafe@yahoo.com)

9p duplication is a clinically recognized syndrome (S.dup9). It includes microcephaly and particular recognizable dysmorphic features. Here, we report the clinical and cytogenetic results in three Tunisian patients with different chromosomal rearrangements resulting in complete 9p duplication in 2 patients and tetrasomy 9p in the third. At physical examination, our patients presented common features including low-set ears, horizontal palpebral fissures, hypertelorism and microretrognathia. They also had microcephaly and psychomotor delay. Cleft palate and clubfeet were observed in one patient. Chromosome rearrangements were characterized using conventional karyotype, fluorescent in situ hybridization (FISH) and array comparative genomic hybridization (array CGH). The karyotype showed three different de novo rearrangements: a supernumerary chromosome marker (MCS) classified as a derivative of the short arm of chromosome 9 (1), isochromosome 9p (2) and a duplication of the short arm of chromosome 9 (3). Array CGH allowed delimiting the extent of duplication to 38 Mb from 9p13.1 to 9p24.3 in two patients. Unlike the majority of cases reported in the literature, duplication was not secondary to a parental translocation. It appeared de novo in all three patients suggesting that an accident occurred during meiosis. S.dup9 has a minimal critical region 9p22.3-9p22.2 responsible for the observed phenotype. A variety of morbid genes such as KANK1, GLIS3, KCNV2, and SLC1A1 are known to be involved in cytoskeletal formation, embryonic development, and cell signaling. At 9p22, the CER1 gene expressed in the anterior mesoderm at the embryo gastrula stage and the FREM1 gene, acting in epidermal adhesion, could contribute to epithelial embryo deregulation leading to orofacial abnormalities. Nevertheless, it is necessary to study the dosage effect of other genes in this region to have a better genotype-phenotype correlation.

#### 1.P59 Interstitial 14q31.3 q32.13 deletion the role of molecular karyotyping in clarifying the etiology of developmental delay

##### Kristina Crkvenac Gornik^1^, Ivana Tonkovic Djurisevic^1^, Anita Pokupec Bilic^1^, Sanda Huljev Frkovic^2^

###### ^1^University Hospital Centre Zagreb, Division of Cytogenetics, Department of Laboratory Diagnostics, Zagreb-Croatia; ^2^University Hospital Centre Zagreb, Division of Genetics And Metabolism, Department of Pediatrics, Zagreb-Croatia

####### **Correspondence:** Kristina Crkvenac Gornik (kcrkvena@kbc-zagreb.hr)

Aim: With the exception of ring chromosome 14 or translocations, interstitial deletions of the long arm of chromosome 14 are very rare. All patients with these deletions share common phenotypic characteristics, primarily mild dysmorphia and developmental delay. Molecular karyotyping (array CGH) enabled the precise breakpoint determination and improved the analysis of genotype-phenotype correlations.

Case presentation: In a 7-year-old girl array CGH was performed due to developmental delay. The array CGH study showed 8.3Mb de novo interstitial deletion of the 14q31.3–q32.13 region.

Conclusions: Comparison of our patient’s phenotype with previously reported chromosome 14q interstitial deletion cases confirmed the presence of common clinical features and highlights the utility of array CGH as a diagnostic tool in clarifying the developmental delay etiology.

Written, informed consent for publication was obtained from the patient [or parent/guardian for patients under 16]

#### 1.P60 The applicability of array CGH and MLPA for the detection of an unbalanced translocation

##### Ivana Tonkovic Djurisevic^1^, Kristina Crkvenac Gornik^1^, Sanda Huljev Frkovic^2^, Anita Pokupec Bilić^1^

###### ^1^University Hospital Centre Zagreb, Department of Laboratory Diagnostics, Division for Cytogenetics, Zagreb-Croatia; ^2^University Hospital Centre Zagreb, Department of Pediatrics, Division for Genetics, Zagreb-Croatia

####### **Correspondence:** Ivana Tonkovic Djurisevic (itonkovi@kbc-zagreb.hr)

We report a case that shows the applicability of array comparative genomic hybridization (array CGH) and multiplex ligation-dependent probe amplification (MLPA) analysis for the detection of an unbalanced translocation.

The patient is a 2-year-old boy with dysmorphic facial features, severe developmental and growth delay. He was hypotonic with congenital contractures of the hips, knees and elbows. Brain MRI showed a simplified gyral pattern in frontotemporal areas and reduction of the white matter with a normal corpus callosum. The initial karyotype was normal 46,XY.

Array-CGH revealed an ~9.2 Mb deletion of 7q36.1q36.3 and a ~12.5 Mb duplication of 16q23.1q24.3. MLPA analysis with subtelomere SALSA MLPA P070 probemix confirmed the existence of the 7q36.1 deletion and 16q23.1 duplication.

Karyotyping and fluorescence in situ hybridization (FISH) analysis of subtelomeric regions of chromosomes 7 and 16 was performed on peripheral blood lymphocytes from the parents. The mother had a balanced translocation 46,XX,t(7;16)(q36.1;q23.1). The patient's karyotype was therefore 46,XY,der(7)t(7;16)(q36.1;q23.1)mat. After discovering the cause of the condition of their child, the parents decided to become pregnant again. Amniocentesis was performed at 17 week's of gestation. MLPA analysis on DNA from amniotic fluid showed a normal result. FISH analysis of cultured amniotic fluid cells detected the inherited, maternal balanced translocation.

MLPA and aCGH can detect small changes that cannot be seen by conventional cytogenetics (microdeletion and microduplication). However, FISH analysis is required in order to detect balanced translocation carriers. Genetic counseling is essential before and after such a diagnosis.

Written, informed consent for publication was obtained from the patient [or parent/guardian for patients under 16]

#### 1.P61 A rare case of partial monosomy 7p22.3p22.1 and partial trisomy 8q24.23q24.3

##### Rahma Touhami^1^, Soumaya Mougou Zerelli^2^, Ilhem Ben Youssef^3^, Sarra Dimassi^2^, Ali Saad^2^, Damien Sanlaville^4^, Amel Haj Khelil1^1^

###### ^1^Faculty of Pharmacy, Molecular Biology and Anthropology Applied to Development And Health, Monastir-Tunisia; ^2^Chu Farhat Hached, Laboratory of Cytogenetics, Molecular Genetics and Human Biology Reproduction, Sousse-Tunisia; ^3^National Institute of Neurology, Department of Pediatric Neurology, Tunis-Tunisia; ^4^University Hospital of Lyon, Department of Cytogenetics, Lyon-France

####### **Correspondence:** Rahma Touhami (rahma.genetique@gmail.com)

Introduction : Intellectual Disability (ID) is defined as the stopping or incompleteness of mental development. Cytogenetics have has allowed the description of a variety of syndromes involving syndromic ID, rarely non syndromic ID.

We report on a consanguineous Tunisian patient of consanguineous parents presenting a facial dysmorphism associated to intellectual deficiency. Clinical examination revealed psychomotor delay, and dysmorphic features, including hypertelorism, high bonbant front, wide nose base, oblique palpebral fissures on the lower side and on the outside, short philtrum.

The standard karyotype analysis revealed a 46,XX,der(7)t(7;8) inherited from a maternal balanced reciprocal translocation.

Comparative genomic hybridization revealing a 9Mb duplication at chromosome 8q24.23q24.3(137078730_146280020)x3 with a loss of 4 Mb on the 7p22.3p22.1(83325_4642192)x1.

Discussion: The phenotype of our patient is characterized by severe psychomotor delay and intellectual deficiency. Tthe duplicated segment found in our patient includes KCNK9 gene (OMIM 605879) whose a mutation of which is associated to with mental retardation, hypotonia and specific dysmorphism: hypertelorism. The 7p22.3p22.1 deletion involves ACTB gene (OMIM 102630)

which is described as candidate gene in developmental delay and short stature. While similar cases of 8q duplication were previously reported, none of them involved the same duplicated segment (8q24.23-8q24.3) identified in our case.

It is interesting to note that cerebral magnetic resonance imaging shows leukodystrophy. These two genes could explain the clinical results of our case.

Conclusion: Array-CGH plays a central role in the characterization of these rare aberrations. The symptoms and associated results may be variable and may depend on the specific size and location of the deleted or the duplicated segment.

#### 1.P62 The first case of 3p26.3 deletion containing only CHL1 gene associated with ASD

##### Andreea Cristina Stanciu^1^, Ioana Streata^2^, Mihai Ioana^2^, Ina Focsa^3^, Magdalena Budisteanu^4^

###### ^1^'Carol Davila' University of Medicine and Pharmacy Faculty of Medicine Bucharest-Romania; ^2^University of Medicine and Pharmacy Craiova Human Genomics Laboratory Craiova-Romania; ^3^'Carol Davila' University of Medicine and Pharmacy Department of Genetics Bucharest-Romania; ^4^'Victor Babes' National Institute of Pathology; 'Prof. Dr. Alex. Obregia' Clinical Hospital of Psychiatry; 'Titu Maiorescu' University Head of Psychiatry Research Laboratory 'Prof. Dr. Alexandru Obregia' Clinical Hospital of Psychiatry Bucharest-Romania

####### **Correspondence:** Andreea Cristina Stanciu (stanciuandreeacristina@gmail.com)

Background: The 3p deletion syndrome is a rare contiguous gene disorder caused by deletions in the 3p25-pter region. It is characterized by growth and mental retardation, developmental delay, dysmorphisms, microcephaly and ptosis. It was suggested that a deletion of a critical region spanning two genes, CRBN and CNTN4, is sufficient to cause the typical clinical features. CHL1 is located distally to CRBN and CNTN4, a gene highly expressed in the brain that plays an important role in neuronal migration, neurite overgrowth and regulation of synapse function. Loss of the CHL1 gene was associated with cognitive impairment. To date only four well documented familial cases presenting a terminal deletion (500 Kb-1.1 Mb) at 3p26.3 including only CHL1 gene, have been reported. All the patients presented a common atypical phenotype for the 3p-syndrome with poor verbal function and developmental delay. It was suggested that this could be good evidence of a new emerging syndrome associated with the deletion of CHL1 gene alone.

Methods and Results: We describe a 12-year old boy in whom array-CGH analysis disclosed a submicroscopic 3p26.3 deletion of 12 Kb spanning only a part of CHL1 gene. Our patient presented mild intellectual disability, speech delay, motor delay and hyperkinesia. The personal history revealed autism spectrum disorder (ASD) and epileptic seizures.

Conclusion: In all five documented cases, including the present case, the phenotype is similarly marked by verbal function and developmental delay, further supporting the possibility of a new syndrome associated with a deletion spanning the CHL1 gene. However, phenotypically this is the first case associated with ASD and genotypically this is the first case with a deletion of such a small size. Further work is necessary to more clearly delineate genotype-phenotype relationships in 3p26.3 deletions.

Written, informed consent for publication was obtained from the patient [or parent/guardian for patients under 16]

#### 1.P63 Cytogenetic findings in ectopic endometrial tissue from infertile women

##### Sophia Zachaki^1^, Marina Kalomoiraki^1^, Elisavet Kouvidi^2^, Manos Promponas^3^, Stefanos Syrkos^3^, Amelia Pantou^4^, Ariadni Mavrou^2^, Emmanuel Kanavakis^4^, Kalliopi Manola^1^

###### ^1^National Center for Scientific Research (NCSR) “Demokritos”, Cytogenetics, Athens-Greece; ^2^Genesis Genoma Lab, Cytogenetics, Athens-Greece; ^3^Genesis Athens Clinic, Surgical Department, Athens-Greece; ^4^Genesis Genoma Lab, Molecular Genetics, Athens-Greece

####### **Correspondence:** Sophia Zachaki (szachaki@genlab.gr)

BACKGROUND/AIM

Endometriosis, is a common gynecological disease, affecting 10% of women of reproductive age and 50% of infertile women. It is characterized by the presence of ectopic endometriotic tissue, and is associated with pain, infertility and in certain cases cancer. Aim: to reveal chromosome and gene alterations of endometrial tissue associated with the disease and possibly malignant transformation.

MATERIALS & METHODS

After informed consent, 22 infertile women who underwent laparoscopic endometriosis surgery at the Athens Genesis Clinic were enrolled in the study. From 17 women with endometriosis, ectopic endometrial tissue was surgically removed and further analyzed by conventional and molecular cytogenetic techniques in 12 cases. FISH was carried out using specific probes for p53, ATM, MYC and IGH genes, the centromeres of chromosomes 7 and 8 and 7q22/7q31 chromosomal regions. Peripheral blood karyotype in all women was also performed.

RESULTS

Constitutional karyotype was normal in 10/12 women, while low X chromosome mosaicism was observed in 2/12. Cell culturing and subsequent karyotypic analysis was successful in 11/12 endometriotic tissue specimens. Sporadic chromosomal abnormalities involving mainly chromosomes 9, 11 and 17 were noticed in 6/11 samples. No clonal chromosomal abnormalities were found. FISH analysis showed IGH gene rearrangements in 6/11(55%) endometriotic tissues, while normal hybridization pattern was observed for p53, ATM and MYC genes. An increased frequency of polyploidy, however, was shown by conventional karyotyping and confirmed by FISH in 8/11 endometriotic tissues.

CONCLUSIONS

A high frequency of polyploidy and IGH gene rearrangements were observed in ectopic endometrial cells indicating their potential role in the development and/or progression of endometriosis and maybe in the endometriosis-related malignancy. Further investigation in a large series of women according to the stage of endometriosis will allow determination of their possible involvement in the pathogenesis of this complex disease.

#### 1.P64 Copy Number Variation (CNV) in Patients Diagnosed with Growth Retardation

##### Emine Ikbal Atli (emine.ikbal@gmail.com)

###### Trakya University Faculty Of Medical, Medical Genetics, Edirne-Turkey

Early recognition of children who cannot exhibit appropriate psychomotor abilities; special education, physical therapy, rehabilitation and other treatment approaches are very important in terms of timely implementation. Early diagnosis of developmental retardations involving all areas of psychomotor development is important, not only cerebral palsy or learning difficulties. Delay in the areas of speech and language development, motor development, social development and cognitive development is defined as developmental delay. Growth retardation is seen 12-16% in childhood. The retardations in the field of language and speech are also parameters for specific developmental situations. In early childhood there is a 5-10% language development problem, 8% learning disabilities, 1-1.5% cognitive dysfunction. Many children experience regression in multiple developmental areas. Growth retardation may be limited to only one area, or may be two or more areas (global growth retardation)

Development areas are classified into four main groups:

1. Motor development
2. Language development (Articulation, language skills, use of nonverbal symbols)
3. Adaptive or cognitive development
4. Personal or social development

Some of the tools used in our country to evaluate development and standardized are as follows:

Denver-II, Gazi Early Childhood Development Assessment Tool, Ankara Development Screening Inventory, Guidance for Monitoring and Supporting Development.

In this respect, CNV + SNP array was performed after karyotyping in 20 patients aged between 1-18 years who were diagnosed with growth retardation among the patients who applied to the Medical Genetics Department of Trakya University. Pathogenic variants were detected in 4 patients. In 1 patient, the change was evaluated as a vous.

#### 1.P65 Trisomy 21 and jumping translocation involving the Y and acrocentric chromosomes

##### Marija Volk, Alenka Veble, Borut Peterlin, Karin Writzl

###### UMC Ljubljana, Clinical Institute of Medical Genetics, Ljubljana-Slovenia

####### **Correspondence:** Marija Volk (marija.volk@siol.net)

Introduction: Translocations between heterochromatin of the long arm of chromosome Y (Yq12) and the short arm of the acrocentric chromosome 15 (15p11-13) represent a common polymorphism. FISH studies showed that bivalent 15 regularly associates with the sex vesicle at the pachytene of normal male meiosis, hence the high frequency of translocations between the chromosomes 15 and Y. Jumping translocations are rare, usually mitotic events, however, they may also occur during meiosis.

Case report: We report on a prenatal case of female karyotype with trisomy 21, where one of three copies of chromosome 21 was derivative chromosome 21, with Yq12 segment translocated to the short arm of chromosome 21 (der(21)t(Y;21)(q12;p13)). Parental karyotyping revealed that mother was a carrier of derivative chromosome 15 with Yq12 segment translocated to the short arm of chromosome 15 (der(15)(Y;15)(q12;p13)). In addition, we performed FISH analyses of fetal and maternal metaphases using probes Vysis CEP15(D15Z1), CEPY(DYZ1), TelVysison Xq/Yq(SYBL1), TelVysison 21q(VIJyRM2029) and Kreatech Acro-p-arms to confirm chromosome rearrangement, aCGH to rule out the presence of euchromatic regions other than SYBL1 gene and STR-testing to exclude UPD15.

Discussion: Carriers of der(15)t(Y;15)(q12;p13) have normal phenotype and fertility, despite a loss of p arm of an acrocentric chromosome and an extra copy of Yq12 or vice versa. Y-acrocentric translocations represent ancestral events that are regularly transmitted to the offspring and are considered as polymorphism of population. However, individual reports of abnormal conceptions or offspring refute a complete harmlessness in the carrier of der(15)(t(Y;15)(q12;p13), whether due to classic recombination or other forms of rearrangements.

Conclusion: Genetic counseling should be reassuring and an offer of prenatal diagnosis be discretionary in case of fortuitously discovered familial der(15)(t(Y;15)(q12;p13). However, a firmer stance may be appropriate if there has been a previous history of an apparently associated reproductive abnormality.

#### 1.P66 Combination of copy number variant at Chr22q and missense mutation in MAP2K1 gene clinical and genetic data

##### Elena Okuneva^*1*^, Anna Krasnenko^2^, Ekaterina Surkova^1^, Olesya Klimchuk^3^, Nadezhda Shilova^4^, Zhanna Markova^4^, Valery Ilinsky^5^

###### ^1^Genotek Ltd., Medical Genetics, Moscow-Russia; ^2^Genotek Ltd., Laboratory, Moscow-Russia; ^3^Genotek Ltd., Bioinformatics, Moscow-Russia; ^4^Medical Genetics Research Centre, Cytogenetic Laboratory, Moscow-Russia; ^5^Genotek Ltd., Director, Moscow-Russia

####### **Correspondence:** Elena Okuneva (elokuneva@bk.ru)

Introduction: chr22q (18745397-19026660) duplication combined with pathogenic variant in the MAP2K1 gene has been reported as a possible cause of developmental delay. Proband: 14-year-old girl with developmental delay, mental retardation, abnormality of the face, contractures of the joints of the lower limbs, symptomatic seizures, atopic dermatitis, sparse, curly hair, spastic tetraparesis. Previously supposed to be cardiofaciocutaneous syndrome.

Methods: We performed targeted sequencing of peripheral blood using Illumina HiSeq2500, NEBNext preparation protocol, Agilent FocusedExome panel and our own analytical pipeline. Variant calling and pathogenicity scoring were done based on ACMG guidelines. Copy number variants were called using reads depth analysis approach and CNVkit software.

Results: We detected a potentially pathogenic copy number variant (CNV) - duplication, 0.28 Mb in size, within the 22q11 region.

The GGT3P, PRODH, DGCR6, DGCR5, DGCR10, DGCR2 and DGCR9 genes are involved in the duplication. Interestingly, DGCR6, DGCR5, DGCR10, DGCR2 and DGCR9 are Digeorge syndrome critical region genes.

Deletion of chromosome 22q11.2 may present with a variety of phenotypes: velocardiofacial syndrome; conotruncal anomaly face; and isolated outflow tract defects of the heart including tetralogy of Fallot, truncus arteriosus, and interrupted aortic arch. A collective acronym CATCH22 has been proposed for these differing presentations.

We also detected a likely pathogenic missense mutation c.389A>G (p.Tyr130Cys) in the MAP2K1 gene. Pathogenic variants in this gene lead to cardiofaciocutaneous syndrome 3 (MIM 614279). The neurologic signs of this syndrome were described as less severe compared to our proband. Probably, a combined effect of CNV and missense mutation in the MAP2K1 gene has resulted in more severe clinical manifestation.

Conclusion: Application of NGS for developmental delay case allowed combined diagnosis of monogenic disorders and conditions caused by chromosomal microanomalies.

Written, informed consent for publication was obtained from the patient [or parent/guardian for patients under 16]

### 2. Tumour Cytogenomics

#### 2.P1 Cytogenetic findings in a large series of patients with acute promyelocytic leukemia

##### Gabriel Vasilakis, Paraskevi Diamantopoulou, Sophia Zachaki, Maria Ioanna Margariti, Domna Pantelia, Marina Kalomiraki, Anastasia Stamatopoulou, Konstantina Panagiotopoulou, Constantina Sambani, Kalliopi Manola

###### Ncsr “demokritos”, Cytogenetics, Athens-Greece

####### **Correspondence:** Kalliopi Manola (pmanola@ipta.demokritos.gr)

Aim: Identification of additional chromosomal aberrations to the classic t(15;17)(q24;q21) and variant translocations in patients with Acute Promyelocytic Leukemia (APL), by conventional and molecular cytogenetic analysis. Materials and methods: Ninety four patients diagnosed with APL were included. The cytogenetic analysis was performed on 24h and 48h unstimulated bone marrow cultures using GTG-banding. Karyotypes were described according to ISCN 2016. Molecular cytogenetic analysis was performed by fluorescence in situ hybridization (FISH) especially in cases with variant t(15;17) and additional chromosomal abnormalities. XL t(15;17) translocation/dual fusion probe was used to detect the chromosomal region 15q24 (PML gene), 17q21 (RARA gene) and the PML-RARA and RARA-PML fusion genes. RARA break-apart probe was used to detect RARA variant translocations. For each sample, 200 interphases (iFISH) and 10 metaphases (mFISH) were analyzed. Results: The sex ratio (males/females) was 1.19 (51 men and 43 women) and the mean age was 50 years (range 10-80). The karyotypic analysis was successful in all patients. Classic t(15;17)(q24;q21) was found in 88 patients (93.6%): in 70/94 (74.5%) as a sole abnormality and in 18/94 (19.1%) with additional abnormalities. The most frequent additional abnormalities were +8 and ider(17)(q10) (4.3% each). Less frequent abnormalities were: add(21q)(2.1%), -7, del(11)(q21), del(9)(q22), t(8;12)(q24;q13), t(1;14)(q21;q32), del(2q), add(15)(p11.2), der(20), t(12;20)(q13;p13) and del(X)(p11.1) (1.1% each). Variant translocations including t(15;17;19)(q24;q21;q13.1-13.3), t(15;18;17)(q24;q21;q21), t(7;17;15)(q22;q21;q24), t(X;15;17;1)(q13;q24;q21;p36), t(11;15;17)(p13;q24;q21) and t(11;17)(q23;q21) were observed in 6/94 patients (6.4%). Conclusions: APL is characterized by a variety of additional chromosomal aberrations to t(15;17) and variant translocations, while the exact prognosis of each of them is not known yet. Our results underline the necessity of karyotype for identification of the whole spectrum of chromosome abnormalities in APL and FISH for identification of variant translocations. Two new variants, t(X;15;17;1)(q13;q24;q21;p36) and t(11;15;17)(p13;q24;q21) are described for the first time.

#### 2.P2 Unique case of e6e2 BCR ABL1 fusion gene transcript in an infant with Ph positive acute lymphoblastic leukemia

##### Tatiana Gindina^1^, Grigory Tsaur^2^, Nikolai Mamaev^1^, Elmira Boychenko^3^, Boris Afanasyev^1^

###### ^1^Pavlov First Saint Petersburg State Medical University, R.m. Gorbacheva Memorial Institute Of Children Oncology, Hematology and Transplantation, Saint-Petersburg-Russia; ^2^Regional Children’s Hospital No 1, Molecular Biology, Immuniphenotyping and Pathology Laboratory, Ekaterinburg-Russia; ^3^City Children’s Hospital No 1, Hematology Department, Saint-Petersburg-Russia

####### **Correspondence:** Tatiana Gindina (tatgindina@gmail.com)

Background. Ph-positive acute lymphoblastic leukemia (Ph+ ALL) is a common entity in adult ALL. In pediatric ALL translocation t(9;22)(q34;q11) is found less frequently - in approximately 3% of cases. Little is known about prevalence of this type of ALL in infant ALL. Combined analysis of 162 infants with ALL and absence of MLL rearrangements enrolled onto Interfant-99 and COG P9407 studies revealed only 2 Ph-positive cases. (P. de Lorenzo Leukemia. 2014; 28(2): 428–430). Ponte di Legno study group presented treatment results of 610 children with Ph-positive ALL, among them there was only 1 case younger than 1 year of age. (M. Arico et al, J Clin Oncol 28:4755-4761). The vast majority of children with Ph-positive ALL carry e1a2 p190 fusion gene transcript.

Material and methods. Cytogenetic analysis, BCR-ABL1 FISH, multicolor FISH, multicolor banding, reverse-transcriptase PCR (RT-PCR) and real-time quantitative PCR (RQ-PCR) were performed on bone marrow sample obtained at initial diagnostics.

Results. 4-month-old girl had a complex karyotype, including reciprocal translocation t(9;22)(q34;q11), monosomy 7, unbalanced translocations der(10)t(7;10), der(13)t(13;21), der(21)t(7;21), as well as insertions of chromosome #10 in the derivative chromosomes ## 9 and 21. By RT-PCR fusion gene transcript BCR-ABL1 vbcr p185 variant e6a2 was detected. This finding was confirmed by direct sequencing of PCR product. Real-time PCR showed that at the time of diagnosis BCR-ABL1/ABL1 normalized copy number was 12.287%. Patient obtained combination of conventional ALL chemotherapy and imatinib. She achieved PCR-negativity at day 15 of remission induction and stayed in hematological and molecular remission.

Conclusion. Based on our knowledge this is the first case of e6e2 BCR-ABL1 fusion gene transcript revealed in infant Ph-positive ALL. Our patient was successfully treated by combination of chemotherapy with imatinib.

“Written, informed consent for publication was obtained from the patient [or parent/guardian for patients under 16]”

#### 2.P3 Novel evidence of karyotype instability in Shwachman Diamond Syndrome

##### Roberto Valli^1^, Abdul Waheed Khan^1^, Giovanni Porta^1^, Annalisa Frattini^2^, Giuseppe Montalbano^1^, Emanuela Maserati^1^, Francesco Pasquali^1^

###### ^1^University of Insubria, Department of Medicine and Surgery, Varese-Italy; ^2^National Council of Research, Uos Milano Irgb, Milano-Italy

####### **Correspondence:** Francesco Pasquali (roberto.valli@uninsubria.it)

Shwachman-Diamond syndrome (SDS), an inherited bone marrow failure condition, implies a risk of developing myelodysplastic syndrome/acute myeloid leukaemia. Two clonal chromosome changes are frequent in the bone marrow (BM): an isochromosome of the long arm of chromosome 7, i(7)(q10), and an interstitial deletion of the long arm of chromosome 20, del(20q).

We suggested in 2000 a peculiar kind of karyotype instability in SDS, with anomalies of chromosome 7 and 20, including the two most frequent ones.

Since 1999, we follow-up a cohort of 97 Italian patients with SDS. Some unexpected odd particularities were found in three cases which further confirm the karyotype instability in BM.

Patient UPN 13: patient who developed a clonal del(20q) in 2009. Array-based comparative genomic hybridization (a-CGH) was performed twice, in 2009 and in 2017. The deletion was different in the two analyses: longer in 2009, shorter in 2017. The only explanation is that the two clones with the deletion originated independently, at different times.

Patient UPN 24: in 2004, the i(7)(q10) was detected. The a-CGH result obtained in 2006 was as expected, with monosomy of the short arm and trisomy of the long arm. In 2012, a-CGH was repeated and the profile of chromosome 7 was different: the monosomy of the short arm was not homogeneous. Only a structural rearrangement of chromosome 7 short arm additional to the i(7)(q10) may explain this result.

Patient UPN 63: patient with the del(20q) found in 2017. In 2018, the a-CGH showed a very small interstitial deletion of the long arm of chromosome 20 (1,729,390 bp): this deletion would not be visible at chromosome analysis. The only explanation is that, at least in 2018, two different clones were present in BM with different del(20q), probably arisen independently from each other.

#### 2.P4 The usability of cytological and molecular tests in pre clinical diagnostics of non invasive urothelial carcinoma. Case study

##### Tadeusz Kałużewski^1^, Grzegorz Przybylski^2^, Jarosław Szwalski^3^, Marek Rożniecki^4^, Adam Jędrzejczyk^5^, Dominika Szewczyk^1^, Agnieszka Morel^1^, Magdalena Owczarek^1^, Michał Bednarek^1^, Bogdan Kałużewski^1^

###### ^1^Genos, Laboratory of Medical Genetics, Łódź-Poland; ^2^Mygenome, Laboratory of Medical Genetics, Poznań-Poland; ^3^Cytopath S.a., Histopathological Laboratory, Łódź-Poland; ^4^Lekarze Urolodzy Marek Rożniecki I Partnerzy, Non-public Department of Urology, Łask-Poland; ^5^Voividship Hospital In Bełchatów, Department of Urology, Bełchatów-Poland

####### **Correspondence:** Tadeusz Kałużewski (t.kaluzewski@gmail.com)

In the presented case, a 66 years old male, neither exposed to harmful occupational factors nor smoking, demonstrated lower urinary tract symptoms (LUTS) without erythrocyturia. In view of the lack of improvement after an initial conservative treatment, an extended diagnostic workup was applied with urethrocystoscopic examination, identifying features of bladder outlet obstructions with no unequivocal macroscopic changes in the bladder mucosa. The patient had for almost two years been pharmacologically treated, without any discernible improvement. A retrospective analysis of intraoperatively obtained video records contributed to the following tests: Cytourofish(+) (a cytological study of urinary sediments, together with FISH- 9p21, 9cen, 17cen chromosome probes and IHC Ki67) and the Bladder EpiCheck test of DNA samples, acquired from urinary sediments (15 proprietary methylation biomarkers). The results of either test indicated the presence of a urinary tract carcinoma. In the course of a subsequent urethrocystoscopic procedure, a randomised biopsy of the bladder mucosa was carried out. A histopathological analysis (endorsed by IHC reactions: CK20, p53, Ki67) of the collected material demonstrated the presence of an urothelial carcinoma in situ. A particular attention was also given to the presence of chromosome 17 polysomy in 100% of the evaluated urinary sediment cells, as well as to the homo-24% and hemizygotic-60% loss in 9p21 sequence. A next-generation sequencing analysis of a DNA sample, acquired from urine sediments, allowed for reproduction of the hypothetical carcinogenesis process activation pathway in the presented case. Those observations highlighted the role of modern, non-invasive, pre-clinical tests in the diagnostics of this disease. Their early implementation may then be considered legitimate and necessary to confirm/exclude the presence of bladder carcinoma, when it comes to LUTS-like symptoms, which may be concomitant to neoplastic changes.

“Written, informed consent for publication was obtained from the patient [or parent/guardian for patients under 16]”

#### 2.P5 When to test MYC rearrangement in multiple myeloma patients

##### Ana Doplihar Kebe, Hermina Čibej, Judita Nussdorfer, Samo Zver, Helena Podgornik

###### University Medical Centre Ljubljana, Department of Haematology, Ljubljana-Slovenia

####### **Correspondence:** Ana Doplihar Kebe (ana.kebe@kclj.si)

Background. MYC rearrangements (MYCr) are late progression events in multiple myeloma (MM). Reported frequency differs considerably among studies ranging from 15% and up to 50% of MM patients. Increased MYC expression is associated with aggressive course of MM, short remissions and overall survival. The aim of our study was to evaluate a possible benefit of MYCr testing in newly diagnosed MM patients.

Methods. MYCr was determined in 124 newly diagnosed MM by FISH on CD-138 isolated plasma cells along with other cytogenetic abnormalities: del(13q), del(17p), IGH rearrangements, 1q gain, del(1p), and hyperdiploidy. Kreatech DNA probe MYC TC BA with established cut-off value 2.5% was used to detect MYCr. Differences between categorical variables were determined by Fisher’s exact test using Medcalc software.

Results. MYCr was found in 15% (18/124) of patients. The clone size ranged from 8 - 95% of plasma cells. There was no statistical difference in frequency of routinely determined chromosomal rearrangements between MYCr positive and MYCr negative group. MYCr was also observed with comparable frequency in standard (8/64) and high risk (10/60) group of patients. As a sole abnormality MYCr was found in 2/18 patients only.

Conclusions. The observed frequency of MYCr in our cohort of almost exclusively newly diagnosed MM patients is consistent with previous literature reports. While MYCr brings no added information to patients with high risk cytogenetics, in about 12% of MM patients with standard risk cytogenetics, corresponding to 6% of newly diagnosed MM patients, it can help to refine prognostication considering the fact that 8q24 abnormalities are a marker of high risk MM. Therefore, a stepwise diagnostic approach should be used with MYCr determination in standard risk MM patients only which present about a half of newly diagnosed MM patients.

#### 2.P6 Acute lymphoblastic leukemia and complex karyotype in a 6 year old patient. Case report

##### Ave Auser^1^, Maarja Karu^2^, Kadri Saks^3^, Riin Klade^1^, Piret Ilisson^1^, Pille Tammur^1^

###### ^1^Tartu University Hospital, United Laboratories, Department of Clinical Genetics, Tartu-Estonia; ^2^University of Tartu, Faculty of Medicine, Tartu-Estonia; ^3^Tallinn Children's Hospital, Department of Haematology and Oncology, Tallinn-Estonia

####### **Correspondence:** Ave Auser (ave.auser@kliinikum.ee)

The translocation t(12;21) is one of the most common rearrangements in pediatric acute B-cell lymphoblastic leukemias (B-ALL) and it is usually associated with a favorable prognosis with a cure rate of 90%. Herein we report a pediatric B-ALL case with t(12;21) within the context of a complex karyotype.

The patient, 6-year-old girl, was referred with the diagnosis of thrombocytopenia. Since the blood work showed pancytopenia, a bone marrow examination was done. The bone marrow morphological findings were consistent with the diagnosis of acute B-cell lymphoblastic leukemia.

Conventional cytogenetics showed a karyotype with multiple abnormalities: deletions in chromosomes 3, 8, 11, 16 and derivative chromosomes 12 and 19.

FISH studies identified t(12;21) ETV6/RUNX1 with a loss of one ETV6 copy in all analyzed cells.

In order to better understand the complex changes, mFISH and a chromosomal microarray analysis (CMA) were additionally carried out.

mFISH revealed a complex translocation t(8;12;21) and a suspected t(3;19).

CMA revealed more duplications and deletions and helped to determine breakpoints of the deletions more accurately. In addition, mosaic monosomy X (15-20%) was found.

With the help of all of the above mentioned approaches the karyotype was characterized as 46,XX,del(3)(p21.31),?t(3;19),del(8)(q21.12),t(8;12;21),del(11)(q14.1q23.3),del(12)(p13.31p12.3),del(12)(q21.33),del(16)(q22.1),del(19)(q13.32),del(20)(q13.12q13.13).

Treatment was started in accordance with the NOPHO ALL-2008 non-high risk protocol and she has tolerated it reasonably well.

The present case highlights the importance of the combination of approaches, i.e., standard karyotyping, interphase FISH, mFISH and CMA for the detection of more complex rearrangements. Precise determination of all rearrangements in complex karyotypes brings important information about the chromosomes, regions, and genes involved in these rearrangements and leads to a better understanding of their clinical and biological importance and its role in leukemogenesis.

“Written, informed consent for publication was obtained from the patient [or parent/guardian for patients under 16]”

#### 2.P7 DSP30 IL2 stimulation in non CLL B cell malignancies

##### Hermina Čibej, Ana Doplihar Kebe, Karmen Dežman, Barbara Kern, Samo Zver, Helena Podgornik

###### University Medical Center, Department of Haematology, Ljubljana-Slovenia ^1^

####### **Correspondence:** Helena Podgornik (helena.podgornik@kclj.si)

Introduction: In accordance with ECA Guidelines the oligonucleotide DSP30 and the cytokine IL2 (DSP30-IL2) should be used in short term cultivation of chronic lymphocytic leukemia (CLL) to maximise the yield of metaphases for successful analysis of banded chromosomes (CBA). We analysed efficiency of DSP30-IL2 stimulation in samples with a suspected B-cell malignancy intended for CBA.

Methods: Peripheral blood (29) and bone marrow (40) samples were cultivated for 48 up to 96 hours with DSP30-IL2. CBA was performed following the standard GTG procedure. 44 analysis was done in CLL patients, 6 in mantle cell lymphoma, 12 in splenic lymphoma, and 3 in cases with suspected B-ALL. Differences between categorical variables were determined by Fisher’s exact test.

Results: CBA was successfully performed in all 69 samples with a high rate of rearranged karyotypes (65% of samples). Significantly (P=0.018) higher proportion of rearranged karyotypes was observed in non-CLL (21/25; 84%) in comparison to CLL samples (24/44; 54%). Also complex karyotypes with more than 3 abnormalities were significantly (P=0.02) more frequent in non-CLL malignancies (8/22; 36%) than in CLL (7/44; 16%). A complex karyotype was revealed in all three cases with suspected ALL. In one of them peripheral blood with 7% of immature granulocytes and 45% of blasts was cultivated with and without DSP30-IL2 stimulation. Although mitotic indices were only slightly higher in stimulated cultures (24‰ vs. 16‰) the rearranged karyotype was revealed only in DSP30-IL2 culture in half of metaphases analysed.

Conclusions: It has been shown that using DSP30-IL2 stimulation CBA can be successfully accomplished also in B-cell malignancies other than CLL including cases with very small clones of malignant B-cells. DSP30-IL2 stimulation results in higher proportion of karyotypes with established clonal rearrangements in different B-cell malignancies. Therefore, its use should become a part of standard procedure in cultivation of malignant lymphocytes B.

#### 2.P8 MAML2 gene rearrangements by fluorescence in situ hybridization (FISH) is diagnostic of mucoepidermoid carcinoma (MEC)

##### Alvin Soon Tiong Lim^1^, Tse Hui Lim^1^, Yvette Yee Shan Yeap^1^, Yit Jun Ng^1^, Josephine Kah Kee Ho^1^, Klara Cheng Har What^1^, Sim Leng Tien^1^, Kah Weng Lau^2^

###### ^1^Singapore General Hospital, Molecular Pathology, Singapore-Singapore; ^2^Singapore General Hospital, Anatomical Pathology, Singapore-Singapore

####### **Correspondence:** Alvin Soon Tiong Lim (alvin.lim.s.t@sgh.com.sg)

Background

Mucoepidermoid carcinoma (MEC) is the most common salivary gland malignancy. It exhibits a spectrum of morphological variation between cases. Differential diagnosis between MEC and MEC-mimics (Warthin tumours, adenosquamous carcinomas (ASC) and oncocytic neoplasms) can be challenging because histochemical stains or immunohistochemistry are often less useful in routine diagnosis.

Aim

Recurrent translocations t(11;19) and t(11;15) resulting in CRTC1-MAML2 or CRTC3-MAML2 fusion oncogenes are identified in a large proportion of MECs of the salivary gland / lung and have impact on prognosis. RT-PCR has been used but because of unknown breakpoints in CRTC1/3 and MAML2, current RT-PCR assay designs might not detect fusion variants. Additionally, RT-PCR may be unsuitable when fusion transcript levels are low. This study sought to determine if FISH might aid to provide a definitive diagnosis.

Methods:

Eleven cases were subjected to MAML2 FISH (6 salivary gland MECs, 1 pulmonary MEC, 1 parotid carcinomas, 1 parotid cyst, 1 low grade neoplasm with clear cell change, 1 pulmonary ASC). An MAML2 break-apart probe (ZytoVision, Germany) was employed using standard FISH protocol.

Results:

Results were obtained in all cases. Eight were MAML2-positive except for the ASC case. FISH confirmed MEC diagnosis in 7 cases, and established the diagnosis of MEC in a case of palatal low grade neoplasm with clear cell change, where the differential diagnosis included acinic cell carcinoma.

Conclusion:

Because MAML2 rearrangements are common and specific for MEC, ascertaining MAML2 disruption is useful in differentiating MEC from MEC-mimics. FISH is an important diagnostic tool in delineating true MEC cases from MEC-mimics.

#### 2.P9 Evaluation of Xpert®Bladder Cancer Detection and UroVysion Bladder Cancer Kit in detection of bladder cancer

##### Andreja Zagorac^1^, Niko KavČiČ^2^, Dejan Bratuš^2^, Ivan PeriĆ^2^, Dragana Taskovska^2^, Franc Kramer^2^, Nadja Kokalj VokaČ^1^

###### ^1^University Medical Centre Maribor, Laboratory of Medical Genetics, Maribor-Slovenia; ^2^University Medical Centre Maribor, Department of Urology, Maribor-Slovenia

####### **Correspondence:** Andreja Zagorac (andreja.zagorac@ukc-mb.si)

Urothelial bladder cancer (UBC) is the 7th most prevalent cancer among men and the 17th most prevalent cancer in women. 75% of newly diagnosed UBCs are non-muscle-invasive bladder cancers (BC), less invasively treated, whereby the urinary bladder is preserved. Because of high recurrence/progression rate these patients should be accurate monitored for early diagnosis and treatment. Regular cytologies, cystoscopies and upper urinary tract imaging are the gold standard for monitoring patients with BC. Many urine molecular tests have been developed to complement or replace cytology and cystoscopy.

We compared two tests: mRNA-based urinary marker test Xpert®Bladder Cancer Detection and FISH test UroVysion Bladder Cancer Kit in a group of 101 persons with hematuria suspected of having BC or subsequent monitoring for tumor recurrence in patients previously diagnosed with BC. Xpert BC test measures the levels of five target mRNAs (ABL1,CRH, IGF2, UPK1B, ANXA10) by RT-PCR and UroVysion Kit detects aneuploidy of chromosomes 3, 7, 17 and loss of the 9p21 locus by FISH. Both tests were performed on voided urine samples prior to cystoscopy.

The sensitivity for both tests was 100%. The overall specificity was 85% for FISH and 79% for Xpert. We found that both tests performed better specificity in the hematuria population than in the surveillance population, 92% versus 72% for FISH and 86% versus 55% for Xpert.

Both tests had high sensitivity and negative predictive value. Lower specificity of both tests, positive FISH and Xpert results with negative cytology/cystoscopy, still can predict a significantly decrease time to recurrence over those patients with both tests negative.

Xpert BC test and UroVysion Kit can help physician in the management of BC. The clinical usefulness of both tests need to be evaluated in a prospective study.

#### 2.P10 Amplification of MYC as double minutes in a patient with acute myeloid leukemia with micronuclei in blast cells

##### Anastasia Athanasiadou^1^, George Papaioannou^1^, George Karavalakis^2^, Maria Gkaitatzi^1^, Zografia Lazarou^1^, Olga Asteriou^1^, Aliki Tsompanakou^1^, Vasiliki Kalaitzidou^2^, Maria Kaliou^2^, Anna Kioumi^2^, Achilleas Anagnostopoulos^1^

###### ^1^G.papanicolaou Hospital, Hematology Department and Hct Unit, Thessaloniki-Greece; ^2^Papageorgiou Hospital, Hematology Department, Thessaloniki-Greece

####### **Correspondence:** Anastasia Athanasiadou (anastasiaathanas@yahoo.gr)

Introduction. Double minutes (dmins) are small chromatin particles that represent a form of extrachromosomal gene amplification. Their presence in acute myeloid leukemia (AML) is rare and is associated with a poor prognosis. The most commonly amplified gene in dmins is MYC. Micronuclei are small intracellular nucleus-like structures that bud or are expelled from a nucleus. They typically contain amplified oncogenes, acentric chromosomal fragments, or whole damaged chromosomes. We present a new case of MYC amplification due to dmins in a patient with AML and micronuclei in blasts.

Case presentation. A 58-year-old woman presented with progressive fatigue. Her medical history involved hypothyroidism, arterial hypertension and hypercholesterolemia. WBC and PLT counts were normal and Hb was 8,6gr/dl. The only biochemical abnormality was a LDH high level. No coagulation abnormalities were present. Bone marrow (BM) aspiration revealed myelodysplastic changes and 38% myeloblasts; some of them with micronuclei. There were dysplastic myelocytes with slightly basophilic cytoplasm that retained the primary azurophilic granules. The BM immunophenotype showed HLADR and CD34 negativity. An increased expression of CD33, CD38, CD13, CD117 and MPO raised the possibility of acute promyelocytic leukemia (APL). BM biopsy revealed infiltration with immature cells with an eccentric nucleus. The cells were MPO positive, had a C Kit expression of 40% and morphological evidence of multilineage dysplasia that supported the diagnosis of APL. Chromosome analysis on BM revealed complex karyotype: 46,XX,+4,add(5)(q13),del(10)(q21),del(17)(p11),idic(22)(p11;p11),+2-56dmin[14]/47,sl,+6[3]/46,XX[3]. FISH analysis using LSI RARA probes showed no rearrangement. Molecular analysis for PML-RARA fusion was also negative. FISH analysis with LSI MYC confirmed C-MYC amplification in the form of dmins. All of the above provided the diagnosis of AML with myelodysplasia–related changes. The patient received an “7+3” induction chemotherapy with Cytarabine and Idarubicin. She had a lower respiratory tract infection on day +9, acute hepatitis on day +12 and encephalopathy on day +17. The patient died on day +40. BM smear showed complete hematologic remission but the examination of cerebrospinal fluid revealed CNS infiltration by AML blasts.

Conclusion. Recently an association between AML with dmins and presence of complex karyotype, trisomy 4, deletion 17p and micronuclei in blasts has been reported. Two patients with M3 morphology with dmins and MYC amplification and no 15;17 translocation have also been reported. One of them died from CNS involvement six weeks after diagnosis having no RARA rearrangement. These cases might represent a new cytogenetic and molecular AML entity sharing common morphologic and clinical features.

“Written, informed consent for publication was obtained from the patient [or parent/guardian for patients under 16]”

#### 2.P11 Rapid and comprehensive screening for disease relevant copy number alterations in multiple myeloma using digital multiplex ligation dependent probe amplification

##### Szabolcs Kosztolanyi^1^, Richard Kiss^2^, Lilit Atanesyan^3^, Ambrus Gango^2^, Karel De Groot^3^, Maryvonne Steenkamer^3^, Pal Jakso^4^, Andras Matolcsy^2^, Bela Kajtar^4^, Laszlo Pajor^4^, Karoly Szuhai^5^, Suvi Savola^3^, Csaba Bodor^6^, Donat Alpar^4^

###### ^1^Clinical Center, University of Pécs, 1st Dept. of Internal Medicin, Pecs-Hungary; ^2^Semmelweis University, MTA-SE Lendulet Molecular Oncohematology Research Group, 1st Dept. of Pathology and Experimental Cancer Research, Budapest-Hungary; ^3^Mrc-Holland, Mrc-Holland, Amsterdam-The Netherlands; ^4^University of Pecs Medical School, Department of Pathology, Pecs-Hungary; ^5^Leiden University Medical Center, Department of Cell And Chemical Biology, Leiden-The Netherlands; ^6^Emmelweis University, MTA-SE Lendulet Molecular Oncohematology Research Group, 1st Dept. of Pathology and Experimental Cancer Research, Budapest-Hungary

####### **Correspondence:** Szabolcs Kosztolanyi (kosztolanyi.szabolcs@pte.hu)

Multiple myeloma is a currently incurable malignancy with diverse genomic background and heterogeneous clinical behavior. Presence, number and composition of recurrent copy number alterations (CNAs) emerging during the pathogenesis and progression of the disease have biological, prognostic and/or predictive significance.

Diagnostic bone marrow samples from 56 myeloma patients were screened for CNAs using digital multiplex ligation-dependent probe amplification (digitalMLPA), a recently developed technique combining conventional MLPA with next-generation sequencing readout. The assay allowed for the assessment of copy number status at 371 genomic loci and for the specific detection of the therapeutically targetable point mutation BRAF V600E.

CNAs including whole chromosome and/or subchromosomal aberrations were detected in all but one sample. The average number of revealed alterations was 4.4 per patient with the highest value of 13. Detailed mapping of CNAs on chromosome 1 identified 24 different patterns among 38 patients harboring loss(1p) and/or gain(1q). Interphase fluorescence in situ hybridization and conventional MLPA used for validating the digitalMLPA copy number data showed a congruency of 95% at genomic loci analyzed by all 3 methods. DigitalMLPA efficiently characterized all whole chromosome changes in patients with hyperdiploid karyotype and unraveled 156 CNAs not detected by the other two methods in 45 patients (80%). Activating V600E mutation of the BRAF kinase was unveiled by digitalMLPA and successfully validated by digital droplet PCR in two patients. DigitalMLPA is a robust technique providing comprehensive and highly rationalized profiling of disease-relevant CNAs as well as specific point mutations in multiple myeloma. The assay has high throughput (potentially up to 192 samples per sequencing run), low input requirement (≥20ng DNA) and short turn-around time (36 hours) facilitating its versatile application in research and clinical diagnostics. Contact: kosztolanyi.szabolcs@pte.hu

#### 2.P12 Molecular characterization of acute myeloid leukemia by next generation sequencing and microarray Technologies

##### Aurora Arghir^1^, Sorina Mihaela Papuc^2^, Alina Erbescu^2^, Raluca Colesniuc^2^, Diana Cisleanu^3^, Dan Soare^3^, Anca Nicolescu^4^, Mihaela Gaman^3^, Irina Voican^3^, Cristina Marinescu^3^, Viola Popov^5^, Meilin Murat^5^, Mihaela Popescu^5^, Oana Patrinoiu^5^, Maria Dobre^6^, Oana Stanca^7^, Silvana Angelescu^8^, Nicoleta Berbec^8^, AnaMmaria Vladareanu^9^, Horia Bumbea^9^

###### ^1^Victor Babes National Institute of Pathology; Carol Davila University of Medicine and Pharmacy, Medical Genetics Laboratory, Bucharest-Romania; ^2^Victor Babes National Institute of Pathology, Medical Genetics Laboratory, Bucharest-Romania; ^3^Carol Davila University of Medicine and Pharmacy; Emergency Universitary Clinical Hospital, Hematology Department, Bucharest-Romania; ^4^Emergency Universitary Clinical Hospital, Hematology Department, Bucharest-Romania; ^5^Colentina Clinical Hospital, Hematology Department, Bucharest-Romania; ^6^Victor Babes National Institute of Pathology, Molecular Pathology Department, Bucharest-Romania; ^7^Coltea Clinical Hospital, Hematology Department, Bucharest-Romania; ^8^Carol Davila University of Medicine and Pharmacy; Coltea Clinical Hospital, Hematology Department, Bucharest-Romania; ^9^Carol Davila University of Medicine and Pharmacy; Emergency Universitary Clinical Hospital; *equal Contribution, Hematology Department, Bucharest-Romania

####### **Correspondence:** Aurora Arghir (aurora.arghir@ivb.ro)

Background: Acute myeloid leukemia (AML) is a heterogeneous disorder characterized by a wide range of driver genetic defects. DNA microarray and next generation sequencing (NGS) proved helpful for deciphering the genomic architecture of AML with important consequences for clinical practice. We report on the results of genomic investigations of 27 AML patients.

Material and methods: Cytogenetic analysis was done on diagnostic bone marrow samples. FLT3 gene internal tandem duplication testing was performed by PCR followed by fragment analysis as previously described. CGH+SNP microarray 4x180k cancer design platforms (Agilent Technologies) were used for genomic profiling. Targeted NGS with a panel covering 19 genes (AML Research Panel) was performed on Ion PGM (ThermoFisher Scientific).

Results and discussion: The cytogenetic risk category was intermediate for 25 patients and adverse for 2 patients. Genomic imbalances and copy-neutral loss of heterozygosity were detected in 7 patients each. The mutational screening revealed a total of 61 mutations, with 51 driver mutations and 10 mutations rarely or unreported in cancer databases. In addition, some uncommon associations were observed, such as between NPM1 mutation (non-type A, B and D insertion) and NRASQ61. The highest mutation prevalence was observed for NPM1 gene (16 patients), followed by DNMT3A (10 patients), and FLT3 (9 patients).

Conclusions: The results obtained in our patients are consistent with previously published data, while also revealing new insights. Sequence analysis and CGH+SNP arrays proved useful for accurate diagnosis, improved prognosis and better therapeutic decisions in cytogenetically characterized AML patients.

Acknowledgments

Partially supported by Ministry of Research and Innovation in Romania, under Program 1 – The Improvement of the National System of Research and Development, Subprogram 1.2 – Institutional Excellence – Projects of Excellence Funding in RDI, Contract No. 7PFE/16.10.2018 and Grants PN19.29.01.03 and PN18.21.01.03.

#### 2.P13 Variability in the extent of del(5q) in myelodysplastic syndromes (MDS)

##### Zuzana Zemanova^1^, Jana Brezinova^2^, Karla Svobodova^1^, Halka Lhotska^1^, Denisa Vesela^1^, Monika Belickova^3^, Jitka Vesela^3^, Silvia Izakova^1^, Iveta Sarova^2^, Libuse Lizcova^1^, Sarka Ransdorfova^2^, Lenka Pavlistova^1^, Jaroslav Cermak^4^, Tomas Stopka^5^, Anna Jonasova^5^, Kyra Michalova^1^

###### ^1^General University Hospital and First Faculty of Medicine, Charles University, Center of Oncocytogenetics, Institute of Medical Biochemistry and Laboratory Diagnostics, Prague 2-Czech Republic; ^2^Institute of Hematology and Blood Transfusion, Department of Cytogenetics, Prague-Czech Republic; ^3^Institute of Hematology and Blood Transfusion, Department of Genomics, Prague-Czech Republic; ^4^Institute of Hematology and Blood Transfusion, Laboratory of Anemias, Prague-Czech Republic; ^5^General University Hospital And First Faculty of Medicine, Charles University, 1st Internal Clinic-clinic of Hematology, Prague-Czech Republic

####### **Correspondence:** Zuzana Zemanova (zuze@vfn.cz)

The interstitial deletion del(5q) is the most common cytogenetic abnormality in myelodysplastic syndromes (MDS) occurring either as a sole aberration or as a part of complex karyotypes (CK). MDS with isolated del(5q) are associated with a favorable outcome while MDS with CK relate to poor prognosis. It remains unclear whether extent of del(5q) matters for the different MDS phenotypes. The aim was to compare the extent of deletion in 264 MDS patients and to assess the relationship of del(5q) extent and TP53 gene mutation.

Extent of del(5q) was analyzed using I-FISH (Abbott), mBAND (MetaSystems) and array CGH/SNP (Illumina or Agilent). Sequence analysis of TP53 gene was performed in 84 cases using NGS on a 454 GS Junior system (Roche) or MiSeq sequencing instruments (Illumina).

In the group of 78 cases with isolated del(5q), the most frequently deleted segment ranged between the bands 5q14 and 5q33.3, with the smallest deletion encompassing the 5q31.1-5q31.3 region (18.527 Mb). The TP53 mutation was proved in 19.4% cases. In the group of 148 cases with CK, the deletion often involved entire long arm including the telomeric region. In this group, the mutation of TP53 and/or LOH17p was detected in 49% of patients. The CDRs were located at the region 5q31.1 (5.522 Mb) in cases with isolated del(5q) and between the bands 5q31.1 and 5q31.3 (18.527 Mb) in patients with CK.

Patients with isolated del(5q) had a smaller size of the deleted segment. More extensive 5q deletion was associated with higher karyotype complexity, increased frequency of TP53 aberrations and worse prognosis. Accurate analysis of breakpoints and range of del(5q) points out to the correlation of deletion size with increasing genomic instability in MDS and contributes to a better understanding of the MDS pathogenesis.

#### 2.P14 Detection of chromosomal aberrations in prostate cancer patients by FISH

##### Magdy Aly, Hanaa Mahmoud

###### Beni-Suef University, Zoology, Beni-Suef-Egypt

####### **Correspondence:** Magdy Aly (magdyaly@yahoo.com)

Fluorescence in situ hybridization (FISH) is a powerful tool for quantitative analysis of chromosomes and genes and can be applied in a variety of specimens, including cell cultures, isolated nuclei from fresh and fixed tissues, and histological tissue sections.Fluorescence in situ hybridization (FISH) with centromere probes was used to investigate numerical aberrations of chromosomes 1, 7, 8, 9, 10, 17, 18, and Y in 52 prostate carcinoma (PC) and 21 benign prostatic hyperplasia (BPH) samples from Egyptian patients. Loss of the Y chromosome was the most frequent chromosomal abnormality in BPH patients and was observed in two patients (9.5%). There was no statistically significant relationship among age, PSA, prostate volume, and chromosomal changes. Forty-seven of 52 PC specimens showed numerical aberrations of one or more chromosomes. All investigated chromosomes showed numerical aberrations in at least 34% of the specimens, gain being more frequent than loss. Chromosome 7 was the most frequently gained (57%), and Y the most frequently lost chromosome (19%). Both groups of tumors differed significantly (P < .05) in the number of copies for chromosomes 7, 8, 9 and 10, but not for 1, 18, and Y. These results suggest strongly that gains of chromosomes 7, 8, and 10 and loss of Y chromosome are involved in PC progression. To our knowledge, this is the first cytogenomic analysis of prostate cancer in Egyptian patients.

#### 2.P15 Cytogenetic abnormalities in uterine leiomyoma cells in vivo and in vitro

##### Alla S Koltsova^1^, Anna A Pendina^2^, Olga A Efimova^2^, Olga V Malysheva^2^, Natalia Yu Shved^2^, Thomas Liehr^3^, Moneeb A K Othman^3^, Maka I Kakhiani^4^, Vladislav S Baranov^2^

###### ^1^St. Petersburg State University, Genetics and Biotechnology, St. Petersburg-Russia; ^2^D.o. Ott Research Institute of Obstetrics, Gynecology and Reproductology, Laboratory for Prenatal Diagnosis of Congenital and Inherited Diseases, St. Petersburg-Russia; ^3^Universitätsklinikum Jena, Institut für Humangenetik, Jena-Germany; ^4^D.o. Ott Research Institute of Obstetrics, Gynecology and Reproductology, Department of Operative Gynecology, St. Petersburg-Russia

####### **Correspondence:** Alla S Koltsova (rosenrot15@yandex.ru)

Uterine leiomyomas (ULs) are benign tumors which are characterized by high frequency of chromosomal abnormalities. We aimed to compare the frequency of cells with chromosomal abnormalities between cultured and non-cultured samples of the same ULs. A total of 32 UL cultures were karyotyped. Twelve out of 32 (38%) UL cultures were karyotypically abnormal. In these ULs, using interphase fluorescence in situ hybridization with DNA probes specific to revealed chromosomal abnormalities, we compared the frequencies of karyotypically abnormal cells in cultured and non-cultured samples. In most cases, the frequencies of karyotypically abnormal cells differed significantly between cultured and non-cultured samples of the same ULs. The frequencies of cells with 47,XX,+12, 46,XX,t(6;10;16)(p21;q22;p13) and 46,XX,del(7)(q21.11q36),t(12;14)(q15;q23) karyotypes were significantly higher in cultured compared to non-cultured samples. In contrast, the frequencies of cells with 46,XX,del(7)(q21.11q22.3), 46,XX,t(4;10;12;?)(p11;q22;q15;?) and 46,XX,t(12;14)(q15;q23) karyotypes as well as with chromothripsis involving chromosomes 1, 8, 14 were significantly lower in cultured compared to non-cultured samples. The frequency of cells with 46,XX,inv(1)(p22p36),t(1;10)(p36;q26) karyotype did not differ significantly between cultured and non-cultured samples. Our findings suggest that UL cultures consist of cytogenetically heterogeneous cell populations, some of which are characterized by a selective growth.

#### 2.P16 Rare MECOM rearrangements in four cases of myelodysplastic syndrome acute myeloid leukemia

##### Gloria Cantamessa^1^, Donatella Fantasia^2^, Giulia Sabbatinelli^3^, Adina Di Nardo^2^, Loredana Cerasa^1^, Melissa Alfonsi^4^, Stefano Grillo^1^, Stefano Pulini^5^, Prassede Salutari^5^, Chiara Cantò^5^, Paolo Guanciali Franchi^1^, Giuseppe Calabrese^1^

###### ^1^G. D’annunzio University, Medical Genetics, Dept. of Medical, Oral and Biotech. Sciences, Pescara-Italy; ^2^Asl Pescara, Oncohaematological Genetics, Pescara-Italy; ^3^G. D’annunzio University, Dept. of Neuroscience, Imaging and Clinical Sciences, Chieti-Pescara-Italy; ^4^ASL Chieti, Medical Genetics, Chieti-Italy; ^5^ASL Pescara, Hematology Unit, Pescara-Italy

####### **Correspondence:** Gloria Cantamessa (gloriacantamessa@libero.it)

MECOM (MDS1-EVI1 Complex locus) rearrangements in 3q26, associated with poor prognosis, occur in 2% of myelodysplastic syndrome (MDS) and acute myeloid leukemia (AML) (De Braekeleer et al., 2015; Hu et al., 2018).

We identified 2 patients with MDS and 2 with AML carrying different rare MECOM rearrangements. In each patient we performed array-CGH and FISH analyses with a panel of 9 RP11-BAC clones spanning the MECOM locus at 3q26.

Patient 1, a 72-year-old male with MDS, presented with a t(3;16)(q26;q22), patient 2, a 64-year-old female with MDS, showed a t(3;12)(q26;q21), patient 3, a 44-year-old female with AML, had a t(2;3)(p21;q26), and patient 4, a 71-year-old female with AML and complex karyotype, had a dicentric chromosome 3 and a t(3;5)(q26;q13).

FISH analysis allowed to map the breakpoints within 3’MECOM in all patients, and in patient 1 revealed a coexisting deletion 150kb in size. Array-CGH was unable to confirm FISH data in patient 1, probably for the presence of leukemic clones with different proliferative rates. In patients 2 and 3 array-CGH revealed breakpoint-associated deletions spanning 73kb and 302kb, respectively, while in patient 4 it confirmed the complex rearrangement, but detected no deletion within the MECOM region.

Patients 1, 2 and 4 died 18 months from diagnosis, while patient 3 underwent allogeneic stem cell transplantation and is currently in hematological and molecular remission. These data confirm the poor prognosis related to MECOM rearrangements. Karyotyping, FISH and array-CGH analyses allowed to identify a 390kb critical region for MECOM rearrangements and highlighted their complementarity in characterization of oncoheamatological rearrangements. Further molecular studies are ongoing to unravel biological and clinical correlations.

“Written, informed consent for publication was obtained from the patient [or parent/guardian for patients under 16]”

#### 2.P17 MLL gene rearrangements in acute myeloid leukemia a series of 17 patients

##### Gayathri Lakshmi Sugumaran^1^, Anjan Madasu^2^, Rodaina H Yousef^3^, Bassam Odeh^4^, Sara Mani^1^, Sehba Naveed^1^, Kesy Liza Varghese^1^, Hussain Essa Alsamt^1^, Sabita K.Murthy^1^, Mahmoud Marashi^2^

###### ^1^Dubai Health Authority, Pathology and Genetics Department, Dubai-United Arab Emirates; ^2^Dubai Health Authority, Pediatric Hematology Unit, Dubai-United Arab Emirates; ^3^Dubai Health Authority, Medical Affairs Department, Dubai-United Arab Emirates; ^4^Dubai Health Authority, Pathology And Genetics Centre, Dubai-United Arab Emirates

####### **Correspondence:** Gayathri Lakshmi Sugumaran (sgayathrilakshmi@gmail.com)

Rearrangements of the MLL (KMT2A) gene on chromosome 11 band q23 region are associated with an adverse risk except for the t(9;11) and the t(1;11) in both acute myeloid leukemia (AML) as well as acute lymphoblastic leukemia (ALL).

We describe a series of 17 out of 370 AML patients who showed MLL gene rearrangements diagnosed at our Centre between November 2010 and February 2019. Interphase FISH analysis using Vysis breakapart probe for the MLL gene was positive for MLL gene rearrangement in all the 17 patients. Fourteen of the 17 patients had shown the rearrangement in G-banded karyotypes also.

The t(10;11) was seen in the majority (n=5) of patients presented with the clinical diagnosis of acute myeloid leukemia -AML M4/M5. Out of these five patients, three were pediatric (median age 20 months) and two were adults ( age 49 and 62 years). There were four males. The translocation involved an inversion 11q in one patient while the other four appeared to show a simple reciprocal t(10;11). The t(10;11) was found as the sole abnormality in four patients, while one patient showed an additional abnormality of partial deletion 9q.

The other translocations involving the MLL gene include the t(4;11) in two patients, the t(1;11),t(7;11), t(9;11),t(11;17), and the t(11;19) in one patient each. All the patients with the t(9;11),the t(11;19),the t(1;11) and the t(4;11) showed some additional cytogenetic abnormalities.

All patients with acute myelomonocytic or monoblastic/monocytic leukemias, with abnormalities of 11q, almost irrespective of the bands involved, should be suspected to harbor a fusion gene resulting from a MLL rearrangement. Hence, FISH or RT‐PCR analyses should definitely be considered in such cases.

#### 2.P18 Ovarian cancer cytogenomics and nuclear motors

##### Aakila Sammy, Joanna Bridger

###### Brunel University London, Life Sciences, Uxbridge-United Kingdom

####### **Correspondence:** Aakila Sammy (Aakila.Sammy@brunel.ac.uk)

In 2012, worldwide there were 152,000 deaths due to ovarian cancer and by 2035 this is projected to increase by 67%. Contributing factors vary by region, however, the two main challenges currently faced are late diagnosis and drug resistance. This study represents a step forward against the disease by probing into the depths of the nucleus, an organelle that codes and arranges essential information by processes not fully understood yet.

To do this we have started by measuring the extent of reorganisation of specific ovarian cancer-related chromosomes and genes whilst comparing them to the cytogenetics of healthy ovarian cells using Fluorescent in-situ Hybridisation (FISH). Also under examination is chromosome reorganisation in drug-resistant cells to likely elucidate the poorly understood dilemma. If drug resistance is recognised as a cytogenetic issue, investigations would carry onto the nuclear motors such as nuclear myosins that chromosomes have been found to use as an adaptation under different physiological conditions. Using RNA interference (RNAi) in conjunction with FISH, the nuclear motor proteins would be temporarily knocked down thus, starving the chromosomes of their adaptive motor facilitator, where exploration of their cytogenetic stagnancy or changes, as a result, can present therapeutic breakthroughs.

Analytic cytogenomics is a new avenue for ovarian cancer that can possibly replace late diagnosis with early onset diagnosis, to progression and prognosis. Also, understanding drug therapy on the same level can present the potential to refine and personalise treatments to effectively reduce toxic drug dose and resistance.

#### 2.P19 FISH studies on CD 138+ cells in multiple myeloma

##### Dolors Costa^1^, Cándida Gómez^2^, Ana Carrió^1^, Amparo Arias^2^, Tania Diaz^3^, Elena Lozano^3^, Gonzalo Rodriguez^4^, Carlos Fernandez^4^

###### ^1^Phd, Hematopathology Unit, Hospital Clinic., Barcelona-Spain; ^2^Technician, Hematopathology Unit, Hospital Clinic., Barcelona-Spain; ^3^Technician, Amyloidosis and Myeloma Unit. Department of Hemathology . Idibaps. Hospital Clínic., Barcelona-Spain; ^4^Dr., Amyloidosis and Myeloma Unit. Department of Hemathology . Idibaps. Hospital Clínic., Barcelona-Spain

####### **Correspondence:** Dolors Costa (dolorscostabordes@gmail.com)

Multiple myeloma (MM) is a clonal late B-cell disorder in which malignant plasma cells expand and accumulate in the bone marrow. The aim of this study was to assess the role of FISH studies on CD138 sorted myeloma cells in the detection of the recurrent cytogenetic abnormalities. The work- flow protocol performed in isolated CD138 cells from 226 patients of our center included: 1) FISH for TP53 and IGH gene, 2) if the IGH gene is rearranged do FISH for IGH/FGFR3, if it is not rearranged, 3) FISH for IGH / MAF, and finally if it is not rearranged 4) FISH for IGH / CCND1. The chromosomal anomalies included in our FISH panel are those recommended by The International Myeloma Working Group (IMWG). Deletion of the TP53 gene was found in 26 (11%) and rearrangement of the IGH gene in 93 (41%) cases. Twenty cases with rearrangement of the IGH gene showed a deletion of 5’ IGH gene that was associated with the IGH / CCND1 rearrangement in 88% of the cases. The rearrangements found in the 67 cases where FISH studies could be performed were: IGH / CCND1 genes (n = 41; 61%), IGH / FGFR3 (n = 13; 19%) and IGH / MAF (n = 4; 6%). In the remaining 9 cases the partner of the IGH gene could not be identified. Conclusion. In case of loss of the 5’IGH gene, it is mandatory to look for the partner of the IGH gene. A new work-flow is proposed based more on the frequency of the rearrangement (IGH/CCND1) than the prognostic value of the rearrangement (IGH / FGFR3 and IGH / MAF).

#### 2.P20 Cytogenetic aberrations in patients with myelodysplastic syndrome and acute myeloid leukemia treated with 5 azacitidin

##### Arpad Szomor^1^, Zsuzsanna Szijarto^2^, Renata Csalodi^3^, Szabolcs Kosztolanyi^3^, Agnes Nagy^3^, Orsolya Toth^3^, Judit Pammer^3^, Bela Kajtar^4^, Alizadeh Hussain^3^

###### ^1^University Pecs, First Department of Medicine, Pecs-Hungary; ^2^University Pecs, Department of Ophthalmology, Pecs-Hungary; ^3^University Pecs, First Department of Medicin, Pecs-Hungary; ^4^University Pecs, Institute of Pathology, Pecs-Hungary

####### **Correspondence:** Arpad Szomor (szomor.arpad@pte.hu)

Myelodysplastic syndrome (MDS), a malignant myeloid disorder is characterized by pancytopenia, hypercellular bone marrow involvement with ineffective hematopoiesis. Patients usually are older with very poor clinical outcome. Similar outcome is present in older patients with acute myeloid leukemia (AML). These disorders are generally incurable, allogeneic hemopoietic stem cell transplantation can give chance to longer survival only in younger patients. Agressive chemotherapy causes serious, often life threatening complications, mainly in the older patients. A new chemotherapeutic drug, 5-azacitidine (5-aza), works as a hypomethylating agent. Side effect profile of this medicine is tolerable, it is a valuable tool both in older patients and in younger individuals, offering a bridge to allogeneic stem cell transplantation. The presence of cytogenetic aberrations is an important part of the prognosis. The aim of our study was to analyze the therapeutic results of 5-aza in the light of cytogenetics. In the last 3 years we treated 16 patients with 5-aza. The age of the patients was 65,6 (26-77) years, 9 female, 7 male pts. Pathologic subtypes of MDS were MDS-RA: 1, CMMoL: 1, MDS-RAEB1: 3, MDS-RAEB2: 5, 6 pts had AML. Nine patients had no chromosomal abnormality, 4/16 had single ( -Y, del(5), -7, ins(3;3) and 3 had complex cytogenetic abnomality. During the observation period 8/16 patients died, 2/16 had allogeneic stem cell transplantation.

#### 2.P21 Trastuzumab augments apoptotic cell death of MCF 7 and MDA MB 231 breast cancer cell lines

##### Mona A. M. Abo-Zeid (monaabozeid@yahoo.com)

###### National Research Centre, Genetics and Cytology Department, Cairo-Egypt

Trastuzumab, a tyrosine kinase inhibitor, is used as a humanized monoclonal antibody to target HER2 amplified and/or overexpressed breast cancer cells. We aimed in the present research to evaluate the efficacy of trastuzumab to enhance apoptotic effect and DNA damage in moderate- and low-expressed HER2 of MCF-7 and MDA-MB-231 breast cancer cell lines. We assessed the cytotoxic effect of trastuzumab on both cell lines after 6, 12, 24 and 48h. Then, the IC50 value related to each cell line after 24h was used to evaluate apoptosis-necrosis effect, DNA damage through micronucleus test and screening immunocytochemistry of caspase-3 antibody as one of the apoptotic proteins. Trastuzumab induced cytotoxic effect on both cell lines in a concentration dependent manner. This cytotoxicity was increased by elevating time-intervals from 6 to 48h. After 24h, the IC50 values were 17.53μg/ml and 26.16μg/ml for MCF-7 and MDA-MB-231 cells respectively. The mean percentages of cell death through apoptosis and induction of micronuclei were increased significantly (p<0.001) by raising the concentration of trastuzumab on both cell lines. Caspase-3 protein was overexpressed in both cell lines after treatment with high concentrations of trastuzumab regarding that observed in non-treated cells. In conclusion, trastuzumab triggered cytotoxicity, caspase-3 protein over-expression and DNA damage of MCF-7 and MDA-MB-231 cell lines, which consequently elevated apoptotic cell death remarkably. Further investigations are required to analyze its mode of action in moderate- and low-expressed HER2 breast cancer cells.

#### 2.P22 A novel complex three way variant Philadelphia translocation involving chromosomes 9 17 and 22 in chronic myeloid leukemia a case presentation

##### Esther Nibbeling^1^, Roos Klinkenberg^2^, Corine Buitenhuis^1^, Marlies Hogenboom^1^, Peter van Balen^3^, Wilma Kroes^1^

###### ^1^Lumc, Clinical Genetics, Leiden-The Netherlands; ^2^Lumc, Internal Medicine, Leiden-The Netherlands; ^3^Lumc, Hematology, Leiden-The Netherlands

####### **Correspondence:** Esther Nibbeling (e.a.r.nibbeling@lumc.nl)

Chronic myeloid leukemia (CML) is a haematological malignancy characterized by neoplastic overproduction of myeloid cells and platelets. The Philadelphia chromosome, t(9;22)(q34;q11), is found in 95% of CML patients, and results in the formation of the BCR-ABL fusion gene with tyrosine kinase activity. However, ~5% of CML patients show complex variant translocations. A novel three-way Philadelphia translocation variant was identified in a 61-year old male patient who presented with weight loss, splenomegaly and leucocytosis and was diagnosed with CML, chronic phase. Karyotyping of bone marrow showed the presence of a three-way translocation involving the long arms of chromosomes 9, 17 and 22. FISH-studies with a BCR-ABL dual-colour dual-fusion probe showed only one fusion signal, on the derivative chromosome 17. Further FISH-tests led to the hypothesis that a (possibly constitutional) paracentric inversion might be present on chromosome 22 with a breakpoint within the BCR gene. We propose that this inversion chromosome 22 is then involved in a two-step mechanism where first a t(9;22) occurs, followed by a second translocation of the derivative chromosome 22 with chromosome 17. Presence of the BCR-ABL fusion transcript was confirmed by PCR and a cytoscan HD SNP-array was performed to exclude loss of chromosomal material. These complex translocations usually do not influence clinical prognosis, and as expected, the patient shows good response to Imatinib treatment. In conclusion, we show a novel complex three-way variant Philadelphia translocation possibly starting from a constitutional aberration in a CML patient.

#### 2.P23 t(11;21)(p14;q22) A translocation with different implications

##### María Talavera^1^, Mónica García Cosío^2^, Ernesto Rodán^3^, Eulalia Rodriguez^3^, Dolores Rey^1^, Jesús Villarrubia^4^, Rosa Lobo^5^, Miguel Piris^4^

###### ^1^University Hospital Ramon Y Cajal, Genetics, Madrid-Spain; ^2^University Hospital Ramon Y Cajal, Pathology, Madrid-Spain; ^3^University Hospital Ramon Y Cajal, Inmunology, Madrid-Spain; ^4^University Hospital Ramon Y Cajal, Hematology, Madrid-Spain; ^5^University Hospital Rio Hortega, Clinical Analysis, Valladolid-Spain

####### **Correspondence:** María Talavera (mtyaguez@gmail.com)

A 21-year-old man diagnosed in 2009 with Ewing’s sarcoma of high grade of left femur treated with neoadjuvant chemotherapy, radiotherapy and surgery (wide resection + intraoperative radiotherapy + prosthesis), was referred to our service for suspected progression (February 2017). At this time, he presents thrombocytopenia and neutropenia. Bone marrow cytology showed a hypocellular marrow with 26% blasts and dysgranulopoiesis, compatible with secondary acute myeloid leukemia (AML). Flow cytometry detected a bone marrow aspiration with 9% blasts (5.5% of myeloid lineage and 3.5% of monocytic lineage). These results are compatible with MDS with excess blasts (WHO 2016 classification).

The pathologist reported a hypocellular bone marrow (15%) with fatty atrophy and lymphoplasmacytic cellularity. The hematopoietic cellularity present is mainly constituted by a red series of regenerative characteristics. Hypocellular granulocytic and megakaryocytic series with correct maturation. Blasts (CD 34 +) were interstitially observed in a percentage of 30% of the total cellularity, which are myeloperoxidase +, Glycoforin A -, compatible with therapy-related myeloid neoplasms.

Conventional cytogenetics showed the karyotype 46,XY,t(11;21)(p14;q22)[9]/46,XY[11]. FISH analyses were performed with probes from Vysis : LSI EGR1/D5S23 Dual Color Probe, D7S486/CEP7 FISH Probe Kit, LSI MLL Dual Color, Break Apart Rearrangement, CBFB Break Apart FISH Probe Kit, LSI RUNX1T1/RUNX1 ES Dual Color Translocation Probe. Interphase FISH with the RUNX1-RUNX1T1 probe revealed in the 25% of nuclei two red signals for RUNX1T1 and three green signals for RUNX1, two smaller than the other. The two smaller green signals suggested splitting of the RUNX1 gene.

This chromosomal rearrangement has only been described in three more patients. A case of MDS with t(11;21)(p14;q22) involving the RUNX1 locus with RUNX1 gene amplification and a complex karyotype (Moosavi et al., 2009), a case of AML-M4 46,XX,t(11;21)(p14;q22)[19]/46,idem,t(12;13)(q22-24;q11)[6] (Arber et al., 2002), and a 78-year-old man diagnosed as AML-M1 with 46,XX,t(11;21)(p14;q22)(Akihiro Abe et al 2012). Our patient and the last one are the only ones who have the translocation as the only anomaly. Their patient was diagnosed with AML, subtype M1, without any dysplasia in his bone marrow. A complete remission could not be achieved. Their patient received palliative chemotherapy and supportive care for 2 years before death. Our patient was diagnosed with AML, subtype M4, was transplanted from his sister without major complications. He relapsed in November (2018), received chemotherapy and in February 2019 the patient was transplanted again with success.

In conclusion, cytogenetics is still necessary in clinical practice, both for the diagnosis and for the follow-up but we need to incorporate new diagnostic techniques in clinical practice to learn more about the translocations and their consequences in terms of gene functions. Such data may have important implications for prognosis and treatment.

“Written, informed consent for publication was obtained from the patient [or parent/guardian for patients under 16]”

#### 2.P24 Complex molecular genetic study of a patient with multiple myeloma

##### Tatiana Abramova^1^, Anna Sergeeva^2^, Vadim Surin^2^, Tatiana Obukhova^1^, Maria Dovydenko^3^, Maria Suntsova^4^, Anton Buzdin^5^, Larisa Mendeleeva^6^

###### ^1^National Research Center for Hematology, Karyology Laboratory, Moscow-Russia; ^2^National Research Center for Hematology, Laboratory of Genetic Engineering, Moscow-Russia; ^3^National Research Center for Hematology, Bone Marrow Transplant Department, Moscow-Russia; ^4^Shemyakin-Ovchinnikov Institute of Bioorganic Chemistry, Group for Genomic Regulation Of Cell Signaling Systems, Moscow-Russia; ^5^Omicsway Corp., Omicsway Corp., Walnut, Ca-United States; ^6^National Research Center for Hematology, Department of High-Dose Chemotherapy of Paraproteinemic Hemoblastoses, Moscow-Russia

####### **Correspondence:** Tatiana Abramova (abramova.blood@gmail.com)

Progression of multiple myeloma (MM) is characterized by the accumulation of secondary chromosomal aberrations, mutations and abnormal gene expression.

We report results of a molecular genetic study of a 59-year-old male with MM at diagnosis and disease progression. The patient received induction therapy with bortezomib-based courses (5 PAD and 4 VCD) followed by autologous hematopoietic stem cell transplantation (auto-HSCT). Before auto-HSCT stringent complete response was observed. On the 100th day after auto-HSCT relapse was diagnosed. Reinduction bortezomib-based therapy was not effective. After 4 months from relapse, the patient died because of severe infectious complications. We performed FISH with DNA probes (XL IGH, XL P53, XL DLEU/LAMP, XL 1p32/1q21, XL cMYC, XL 5p15/9q22/15q22 (MetaSystems)) on mononuclear cells and CD138-positive cells at diagnosis and in progression, respectively. We performed whole-transcriptome analysis by RNA-sequencing (RNA-Seq) on Illumina HiSeq (Illumina). Somatic mutations of the N-RAS, K-RAS, TP53, BRAF (exon 15) genes were analyzed in CD138-positive plasma cells by Sanger sequencing.

FISH revealed hyperdiploidy (trisomies 5,9,15) at diagnosis and in progression. Amp1q21 was not found at diagnosis and was detected in disease progression only. Before treatment and in disease progression a heterozygous clonal mutation p.182A>C (p.Q61P) was detected in the N-RAS gene, which is involved in MAPK-pathway regulation. RNA-Seq showed a dramatic increase (30 times) of IL6 gene expression at the time of relapse. Disease progression was accompanied by increased expression of key regulatory genes (c-MYC, Notch2, MDM2, RAF1, STAT4, mTOR) and a strong reduction of IGH genes expression, which caused a deep immunodeficiency.

Thus, we conclude that complex molecular genetic study at diagnosis as well as during disease progression allows to identify genetic factors that appear in disease progression and are probably associated with progression and resistance to therapy.

“Written, informed consent for publication was obtained from the patient [or parent/guardian for patients under 16]”

#### 2.P25 Detection of homogeneously staining regions in leukemic patients

##### Andrea Blahová, Alexandra Oravcová, Kristína Lengyelová, Marta Bombarová, Alena Žákovicová, Kristína Juríková, Jakub Petrík, Renata Lukačková

###### Department of Genetics, Medirex a.s., Bratislava, Slovakia

####### **Correspondence:** Andrea Blahová (andrea.blahova@medirex.sk)

Homogeneously staining regions (hsr) just as double minute chromosomes (dmin) represent cytogenetically visible signs of gene amplification. It is known that they contain copies of an amplified DNA segment. They were first described in a direct preparation of cells from a patient with untreated bronchogenic carcinoma. Altohough found in a variety of human cancer cells, their presence in hematologic malignancies is rare. Their role in leukemogenesis is not clear but they have been reported to be associated with rapid progression and short survival time. The mechanism for generating the hsr is not known exactly. Characteristically, hsr can be detected after chromosome banding in metaphase preparations as a large chunk of diffusely staining chromatin somewhere inside an ordinary chromosome.

We report three patients with acute myeloid leukemia (AML) and myelodysplastic syndrome (MDS) who were examined at the time of diagnosis at our department of genetics. All of them were examined with cytogenetics, FISH and molecular methods. Conventional G-banding analysis after 24hour cultivation of bone marrow revealed complex karyotype with deletion of long arm of chromosome 5, with amplifications of different genes, that created homogenously stained regions, and with other numerical and structural changes. The use of FISH lead to the identification and specification of the percentages of these amplifications and aberrations. For detecting genetic changes at the molecular level we had used MLPA or AMLplex analysis. Futhermore, we examined WT1, NPM1 and FLT3 genes. All 3 our patients shared the same characteristics: they were old, they had a diagnosis of novo AML or MDS with complex karyotype, they had deletion of 5q, had bad prognosis and short survival.

#### 2.P26 Mantle cell lymphoma – possibilities of FISH in the diagnosis

##### Jana Zmolikova^1^, David Starostka^2^, Natalie Lencova^2^, Sylva Pitronova^1^, Magdalena Uvirova^1^, Jarmila Simova^3^, Radek Mech^3^, Martin Tichy^4^, Jana Dvorackova^4^

###### ^1^Cgb Laboratory Inc., Cytogenetic Laboratory, Ostrava-Vitkovice-Czech Republic; ^2^Hospital With Health Center Havirov, Department of Clinical Hematology, Havirov-Czech Republic; ^3^Cgb Laboratory Inc., Laboratory of Molecular Biology, Ostrava-Czech Republic; ^4^Cgb Laboratory Inc., Laboratory of Pathology, Ostrava-Czech Republic

####### **Correspondence:** Jana Zmolikova (zmolikova@pathology.cz)

Mantle-cell lymphoma (MCL) is an incurable, aggressive hematologic cancer with a poor prognosis (median survival 4 to 5 years). MCL is an uncommon subtype of non-Hodgkin lymphoma with distinctive clinical, biological, and molecular characteristics. The molecular hallmark in MCL is the t(11;14)(q13;q32) translocation, which transposes the cell cycle regulator CCND1 (11q13) under control of the immunoglobulin heavy chain (IGH) locus (14q32) and leads to overexpression of cyclin D1.

During 2012 to 2018 40 samples (bone marrow (BM) aspirates, BM biopsies, lymph nodes, peripheral blood) from 31 MCL patients (23 males and 8 females, with a median age of 73 years) were investigated for the t(11;14)(q13;q32) translocation by FISH (Kreatech CCND1/IGH t(11;14) Fusion). The t(11;14)(q13;q32) was detected in 10/16 cases of BM aspirates and these 6 negative patients had paraffin-embedded samples of BM biopsies investigated – 1/6 case was t(11;14) positive. Peripheral blood – 2/2 cases were t(11;14) positive. Lymph node – 11/11 cases were t(11;14) positive. Three t(11;14) positive patients were investigated also after the treatment and then were negative.

The identification of t(11;14)(q13;q32) in diagnosis of MCL is crucial. It is possible to detect this translocation in various types of cells, thus to supplement the examination in patients where the aspiration of BM was not successful or possible, as well as where the translocation in BM aspirate was not proven. By comparing the results of different patient materials, it is possible to individualize the treatment and stratify patients to the prognostic groups e.g., CD5- form of MCL without t(11;14)(q13;q32) in BM and with cyclin D1 positivity, which according to the literature may have indolent course of disease. Therefore, to determine the prognosis and the choice of the correct treatment all available data should be considered: clinical, cytomorphological, histological, immunophenotypic and genetic.

#### 2.P27 Rare Philadelphia chromosome variant translocations and their clinical outcome a single center experience

##### Marija Vukovic^1^, Mirjana Beric^1^, Jadranka Mirjanić^2^, Sandra Lazarevic^3^, Semir Mesanovic^4^

###### ^1^University Clinical Centre, Department of Medical Genetics, Banja Luka-Bosnia and Herzegovina; ^2^University Clinical Centre, Department of Haematology, Banja Luka-Bosnia; ^3^University Clinical Centre, Department of Haematology, Banja Luka-Bosnia and Herzegovina; ^4^University Clinical Center, Department of Patology, Tuzla-Bosnia and Herzegovina

####### **Correspondence:** Marija Vukovic (zaramarie96@gmail.com)

The genetic basis of chronic myeloid leukemia (CML) is in 90% of cases a rearrangement between the BCR gene on chromosome 22 and the ABL1 gene on chromosome 9. This translocation generates a BCR-ABL1 fusion gene located on the derivative chromosome 22, cytogenetically known as Philadelphia chromosome (Ph). In 10% of cases one or more chromosomes can be involved in generating the Philadelphia chromosome. During 10 years we detected four CML cases with variant Ph translocation. Three of them were three-way rearrangements including chromosome regions 10q22, 17q21 and 19q13.1. The fourth case was a complex Ph variant including four chromosome regions 5q11, 9q34, 21q22 and 22q11.2.

All these karyotypes were detected at diagnosis of CML. BCR-ABL1 fusion gene was detected and measured using quantitative reverse transcription polymerase chain reaction (RQ-PCR). The patients were treated with Imatinib, first generation tyrosine kinase inhibitors (TKI). The patients were continiously monitored every 3 months according to molecular response. All of them had a very good clinical outcome. The fourth case, a young women, succesfully delivered a healthy baby and another one has no detectable BCR-ABL transcript by ASO PCR method, after two years of therapy with TKI. Our experience does not show worse prognosis of patients with complex variants of t(9;22).

#### 2.P28 Status of TMPRSS2–ERG gene fusion in a cohort of prostate cancer patients and its association with clinicopathological characteristics

##### Alena Burdova^1^, Honza Bouchal^2^, Zuzana Lencesova^1^, Zdenek Kolar^2^

###### ^1^Agel Laboratories, Laboratory of Medical Genetics, Novy Jicin-Czech Republic; ^2^Faculty of Medicine and Dentistry, Palacky University and University Hospital, Department of Clinical and Molecular Pathology, Olomouc-Czech Republic

####### **Correspondence:** Alena Burdova (alenaburda@seznam.cz)

Prostate cancer (CaP) is one of the most frequent malignancies and the most common cause of death in men. This is a heterogeneous disease that currently does not have reliable diagnostic and predictive markers and very often there is indicated an inadequately aggressive therapy. The main focus of current research on CaP is therefore finding and testing of new biomarkers. Such a marker could be the TMPRSS2-ERG gene fusion.

We used formalin-fixed paraffin-embedded tissue and evaluated TMPRSS2-ERG gene fusion by fluorescence in situ hybridization (FISH). The results were correlated to clinicopathological characteristics which are: age, PSA, Gleason score, pT stage and lymph node metastasis.

Of 155 cases examined, positive FISH results were seen in 39% (60/155) of cases. Deletion associated with the TMPRSS2-ERG gene fusion was detected in 33 patients (55 % positive cases). Rearrangement of TMPRSS2 gene was found in 27 patients (45 % positive cases). The ploidy of 21q22 locus was also detected in 20 cases of total 155 cases.

We demonstrated a statistically significant positive correlation between the percentage of cells with TMPRSS2-ERG gene rearrangement in CaP and metastatic potential of tumors. The higher the percentage of positivity, the more frequent the incidence of lymph node metastases (p=0.011). The Fisher test confirmed a statistically significant increase in lymph node metastasis in cases of CaP with 4n ploidy (p=0.049). No statistically significant association with other clinicopathological characteristics, mentioned above, was observed.

In summary, these data suggest that the TMPRSS2–ERG gene fusion in our cohort of CaP patients may contribute to a more aggressive CaP phenotype. Our results also confirm the important role of the TMPRSS2-ERG gene fusion in the pathogenesis of CaP.

#### 2.P29 Hyperdiploid acute lymphoblastic leukemia with genome wide copy neutral loss of homozygosity (CN LOH)

##### Karin Nebral^1^, Sabrina Haslinger^1^, Andrea Inthal^1^, Margit König^1^, Sören Mai^2^, Sven Wohlmacher^2^, Sabine Strehl^3^, Andishe Attarbaschi^4^

###### ^1^St. Anna Kinderkrebsforschung, Children's Cancer Research Institute, Ccri, Clinical Genetics, Vienna-Austria; ^2^Labdia, Gmbh, Vienna, Austria, Clinical Genetics, Vienna-Austria; ^3^St. Anna Kinderkrebsforschung, Children's Cancer Research Institute, Ccri, Genetics of Leukaemias, Vienna-Austria; ^4^St. Anna Children’s Hospital, Medical University of Vienna, Department of Pediatric Hematology And Oncology, Vienna-Austria

####### **Correspondence:** Karin Nebral (karin.nebral@medgen.at)

Pure tetrasomies are the hallmark of a distinct subset of hyperdiploid ALL in children. The analysis of such cases with CGH/SNP array revealed a CN-LOH of all disomic as well as the duplication of both homologs of all tetrasomic chromosomes. It is generally believed that this distinct pattern can only derive from the duplication of a preexistent analogous hyperhaploid clone. As apparent indicator of poor prognosis, such hyperhaploid/-diploid ALL forms are nowadays stratified as high risk in ongoing treatment protocols. Our array analyses of patients that were enrolled in Austrian AIEOP-BFM treatment trials identified so far seven such cases (4 girls, 3 boys). Their chromosome copy number ranged from 48 to 58 and comprised tetrasomies of chromosomes 21 (7x), X (5x), Y (3x), 14 (3x), 18 (2x), 4, 9 and 10 (1x each), respectively. Our careful evaluation with cytogenetic, FISH and DNA index analyses identified a hyperhaploid clone in two of them. One of these cases was stratified as standard, three as medium and three as high risk (one because of a concomitant small BCR/ABL1-positive clone and the two bi-clonal ones according to protocol risk group specification). Five patients are in continuous first remission already between 2.1 and 9.5 (median 8.5) years, one for 6 months and one lost to follow-up. Our results strongly indicate that, at least in the AIEOP/BFM treatment setting, the prognostic outlook of patients with only a CN-LOH hyperdiploid clone seems to be extremely favorable, a notion that needs to be further corroborated in a larger cohort. Since hyperhaploid and analogous hyperdiploid forms cannot be distinguished based on array patterns alone, we consider it essential and mandatory to assess their individual contribution in an at least semiquantitative manner with cytogenetic, FISH and/or DNA index analyses.

#### 2.P30 Is RB1 gene deletion a prognostic marker in chronic lymphocytic leukemia

##### Beyhan Durak Aras^1^, Nur Sena Inci^1^, Abdulvahab Aslan^2^, Olga Meltem Akay^3^, Eren Gunduz^4^, Tuba Bulduk^4^, Sevgi Isik^1^, Oguz Cilingir^1^, Gulcin Gunden^1^, Filiz Yavasoglu^5^, Mustafa Karagulle^6^, Ahmet Seyhanli^7^, Sevilhan Artan^1^, Hulya Ozen^8^, Zafer Gulbas^9^

###### ^1^Eskisehir Osmangazi University Faculty of Medicine, Department of Medical Genetics, Eskisehir-Turkey; ^2^Private Umit Hospital, Department of Hematology, Eskisehir-Turkey; ^3^Koc University Faculty of Medicine, Department of Hematology, Istanbul-Turkey; ^4^Eskisehir Osmangazi University Faculty of Medicine, Department of Hematology, Eskisehir-Turkey; ^5^Afyon Health Sciences University Faculty of Medicine, Department of Hematology, Afyon-Turkey; ^6^Yunus Emre State Hospital, Department of Hematology, Eskisehir-Turkey; ^7^Dokuz Eylul University Faculty of Medicine, Department of Hematology, Izmir-Turkey; ^8^Eskisehir Osmangazi University Faculty of Medicine, Department of Biostatistics, Eskisehir-Turkey; ^9^Anadolu Medical Center, Department of Hematology, Kocaeli-Turkey

####### **Correspondence:** Beyhan Durak Aras (bdurak@ogu.edu.tr)

Deletion of 13q14 is the most common cytogenetic change (50%) in chronic lymphocytic leukemia (CLL), and it is a good prognostic factor if detected as a sole aberration in FISH analysis. In recent studies two main types of 13q14 deletions are proposed: type I (short) and type II (larger) which includes RB1. Some investigators suggest type II deletion to be associated with a more aggressive course. In this study, we aimed to investigate the relationship between RB1 gene deletion and overall survival (OS), disease stage and time to first treatment (TTFT) in patients with isolated 13q deletion.

RB1 deletion was analyzed in peripheral blood of 53 patients with isolated 13q deletion by FISH. The median age of patients is 66.88 ± 8.83 (42-88), and the 64% of patients are male. RB1 deletion was detected in 41% (22/53) of patients, but there was no statistically significant difference between RB1 deletion and TTFT, stage and OS (p=0,26, p=0,73, p=0,92). At the same time, statistically significant difference was detected between high 13q14 deletion (>60%) and TTFT (p=0,0038).

The statistical analysis of our data regarding association between RB1 deletion and TTFT, stage, and OS has not confirmed that type II deletion cause poor prognosis. But the patiens with RB1 deletion have shorter OS and TTFT. Unfortunately, our statistical evaluation was done without clinical data for six patients (4/6 of them are RB1 deletion positive); thus, our results must be regarded as preliminary. In particular, more data on patients with isolated 13q deletions are needed. A continuation of our study is in progress.

#### 2.P31 Cytogenetic and molecular techniques (karyotype FISH mFISH and array CGH) for precise characteristization of struсtural chromosome abnormalities in chronic lymphocytic leukemia a case report

##### Maria Kislitsyna^1^, Tatiana Obukhova^1^, Tatiana Abramova^1^, Lyuba Grebenyuk^1^, Darima Badmazhapova^2^, Milyausha Tagirova^3^

###### ^1^National Research Center for Hematology, Cytogenetic Laboratory, Moscow-Russia; ^2^National Research Center for Hematology, Gematology, Moscow-Russia; ^3^National Medical Research Center for Obstetrics, Gynecology and Perinatology Named After Academician V.i.kulakov, Pathology, Moscow-Russia

####### **Correspondence:** Maria Kislitsyna (makislitsyna@gmail.com)

Chronic lymphocytic leukemia (CLL) is a clinically and genetically heterogeneous disease. Interstitial deletion of 13q14 is the most frequent chromosomal aberration detected by FISH and can be represented by mono- or biallelic deletion. In 50% of patients del13q14 is submicroscopic and is not visible by chromosome banding analysis (CBA). aCGH allows not only to detect submicroscopic copy number changes, but also to determine their size. Using mFISH identification of all balanced and unbalanced chromosomal rearrangements is possible.

We report on a case of 81-year-old male with CLL analyzed by CBA, FISH, aCGH and mFISH for precise characteristization of the tumor clone.

CBA was performed by using oligonucleotide DSP30 и IL2. We used DNA probes for detection trisomy 12, deletions 13q14, 11q22, 17p13, rearrangements IGH for FISH; a CytoSure Constitutional v3 array (80x60k) and a 24XCyte mFISH probe.

By CBA we identified del(13)(q12q22) in one allele of chr13. FISH-analysis detected interstitial del13q14 in one allele and large deletion of chr13 in another allele (lack of signals of 13q14 and 13q34) and dim signal of IGHV (14q32). aCGH confirmed loss 14q32.33 and 13q14, but deletion region was q13q34 (116,74 Mb) and revealed additional change - gain of 3q26.1q29 locus, that was not consistent with karyotype data. mFISH showed additional chromosomal material 3q26q29 on chr13. Thus, on the basis of the results of all used methods we identified interstitial del13q14 in one allele, unbalanced t(3;13)(q26-q29;q13) with large del13(q13-q34) in another allele and partial deletion of locus IGHV/14q32.

We conclude that the combination of cytogenetic and molecular methods CBA, FISH, mFISH and aCGH is effective for detailed characterization of structural chromosomal abnormalities in patients with hematologic tumors.

“Written, informed consent for publication was obtained from the patient [or parent/guardian for patients under 16]”

#### 2.P32 The assessment of chromosomal instability using telomere and centromere staining followed by M FISH technique Requirements and perspectives

##### Radhia M'kacher^1^, Bruno Colicchio^2^, Leonhard Heidingsfelder^3^, Kevin Soehnlen^1^, William M Hempel^1^, Alain Dieterlen^2^, Patrice Carde^4^, Eric Jeandidier^5^

###### ^1^Cell Environment, Dna Damage R&d, Paris-France; ^2^Université De Haute-Alsace, Laboratoire Mips-Groupe Tiim3d, Mulhouse-France; ^3^Metasystems Gmbh, Microscopy, Altlussheim-Germany; ^4^Gustave Roussy Cancer Campus, Department Of Medicine, Villejuif-France; ^5^Groupe Hospitalier De La Région De Mulhouse Et Sud-alsace, Service De Génétique, Mulhouse-France

####### **Correspondence:** Eric Jeandidier (jeandidiere@ghrmsa.fr)

Purpose:

Chromosomal instability is the most important predictive and prognostic biomarker in the management of cancer patients. Telomere dysfunction is associated with dicentric chromosome formation leading to malignant cancer progression and poor clinical outcome. However, the detection of dicentric chromosomes using conventional techniques can easily be mistaken for translocations. In this study, we improved the detection of dicentric chromosomes and telomere dysfunction, both being chromosomal instability driving forces.

Material and methods:

A cohort of fifty patients with hematopoietic malignancies and 100 healthy donors were analyzed. In addition, twenty human cell lines were examined. Sequential analysis using telomere and centromere staining followed by M-FISH technique was performed to characterize telomere dysfunction and chromosomal aberrations.

Results:

Significant telomere shortening was found in cancer patients compared to similar age matched healthy donors (p<10-8). Telomere dysfunction (losses and deletions) was also detected in cancer patients. Moreover, dicentric chromosomes were detected in 28/50 patients. Interestingly, significant decrease (p<10-3) of telomere length was observed in patients with dicentric chromosomes compared to patients without dicentrics. Two different mechanisms were involved in the formation of the dicentric chromosomes. The most frequently observed mechanism resulted in centromere breakpoint associated with the presence of specific configuration of dicentric chromosome with both centromeres in close proximity and with the loss of 17p (TP53). The other mechanism was related to telomere breakpoints and chromosome end fusions leading to the formation of dicentric chromosomes. Breakage/fusion/bridge cycles related to the presence of dicentric chromosomes lead to chromosomal instability. Re-evaluation of the karyotype of human cell lines using this approach confirmed the presence of dicentric chromosomes related to telomere end fusions or to centromere breakpoints.

Conclusion:

Sequential analysis using telomere and centromere staining followed by M-FISH technique has not only allowed accurate detection and characterization of chromosomal aberrations, but has also rendered it possible to assess the potential role of telomere dysfunction and chromsomal instability to improve initial treatment strategy on an individual basis.

#### 2.P33 PON1 Q192R genetic polymorphism as a predisposing factor for leukemia and its specific cytogenetic aberrations

##### Agapi Ioannidou, Vasiliki Chatzidrosou, Sophia Zachaki, Aggeliki Daraki, Paraskevi Diamantopoulou, Maria Ioanna Margariti, Domna Pantelia, Panagiotis Nakopoulos, Contantina Sambani, Kalliopi Manola

###### NCSR “Demokritos” Cytogenetics ATHENS-Greece

####### **Correspondence:** Agapi Ioannidou (mrshcn@gmail.com)

Aim: Investigation of Q192R polymorphism of PON1 (paraoxonase) gene, a gene which plays a vital role in antioxidant defense, as a predisposing factor in Chronic Lymphocytic Leukemia (CLL) and Acute Myeloid Leukemia (AML) and the formation of their specific chromosomal aberrations.

Method: The study included 300 CLL patients, 85 AML patients and 104 healthy individuals. Karyotypic analysis was performed on 24h, 48h and 72h bone marrow cultures of the patients and Q192R genotyping using PCR-RFLP assay. Genotypic and allele frequency distributions were compared by x2 test. p-value<0.05 was considered significant. Odd ratios were given with 95% confidence interval.

Results: Cytogenetic analysis in CLL was successful in all patients. Normal karyotypes were found in 118/300 (39.3%) patients while abnormal in 182/300 (60.7%) including del(13q) (n=61 patients), +12 (n=56), del(11q) (n=40), del(17p) (n=24), abn14q32 (n=21), del(6q) (n=9), complex karyotypes (n=56). Cytogenetic analysis in AML was successful in 83/85 patients (98%). Normal karyotypes were noticed in 36% while abnormal in 64% including +8 (n=10), t(15;17) (n=6), -7/del(7q) (n=8), abn11q (n=5), +21 (n=3), t(9;22) (n=3), +11 (n=2), inv(3) (n=2) and complex karyotypes (n=7).The genotypic analysis was 100% successful. Similar genotypic and allele frequency distributions were found between AML patients and controls (p=0.761). On the contrary, in CLL, a statistically significant higher frequency of the mutant genotypes (QR/RR) was observed in patients compared to controls (QQ:48.3%, QR:44.0%, RR:7.6% vs QQ:63.5%, QR:31.7%, RR:4.8%, respectively, p=0.028). R allele frequency was also higher in CLL patients (29.67% vs 20.67%, p=0.012). Moreover, a higher frequency of the mutant genotypes was found in CLL patients with abn(14q32) (p=0.03) and del(6q) (p=0.021) compared to controls.

Conclusion: PON1 Q192R polymorphism may be implicated in CLL pathogenesis and the formation of specific chromosomal aberrations but not in AML pathogenesis.

#### 2.P34 Case report of rare translocation t(3;3)(p24;q26) in myeloid leukemia patient

##### Lyubov Grebenyuk^1^, Tatiana Obukhova^1^, Lyubov Shishigina^1^, Tatiana Abramova^1^, Svetlana Kuznetsova^1^, Andrey Sudarikov^2^, Elena Parovichnikova^3^, Valeriy Savchenko^3^

###### ^1^National Research Center for Hematology, Сytogenetic Laboratory, Moscow-Russia; ^2^National Research Center for Hematology, Laboratory of Molecular Hematology, Moscow-Russia; ^3^National Research Center for Hematology, Department of Chemotherapy of Hemoblastosis, Hematopoietic Depression and Bone Marrow Transplantation, Moscow-Russia

####### **Correspondence:** Lyubov Grebenyuk (lyuba.grebenyuk@yandex.ru)

The EVI1/3q26 gene is currently recognized as one of the dominant oncogenes associated with myeloid leukemia. Chromosomal abnormalities involving the EVI1/3q26 gene locus are found in approximately 2% of patients with myeloid leukemia and are extremely unfavorable prognostic factors.

We report on a case of Ph-negative myeloproliferative neoplasm, unclassifiable (MPN-U), with rare t(3;3)(p24;q26) in 59-year-old male patient.

In 2014, at diagnosis, conventional cytogenetic analysis (CCA) of bone marrow cells revealed normal karyotype. We performed molecular cytogenetic and molecular genetic analysis that included FISH, RT-PCR and Sanger sequencing for detection BCR/ABL translocation, EVI1 rearrangement and mutations in JAK2, MPL, CALR, FLT3, NPM1, CEBPA genes and did not found any chromosomal aberrations or mutations.

Since May 2017, on the cytoreductive therapy, progression of the disease was diagnosed with the development of blast myeloid crisis. The CCA revealed a derivative chromosome 3 and additional unidentifiable chromosomal material on 20p. We performed FISH, mFISH, mBAND and revealed rare t(3;3)(p24;q26) with EVI1/3q26 rearrangement and unbalanced t(6;20)(q21;p12). Using fragment analysis FLT3-ITD mutation was found. In 13 months patient died of chemotherapy resistance and disease progression.

We revealed an extremely rare t(3;3)(p24;q26) in Ph-negative myeloproliferative neoplasm patient. Currently, in the literature there are descriptions of two clinical cases with translocation t(3;3)(p24;q26) and ten cases of pericentric inv(3)(p24q26). EVI1 partner in these rearrangements is still unknown. In our case, t(3;3)(p24;q26) with EVI1/3q26 was not found at diagnosis by CCA and FISH, detected in disease progression and associated with chemotherapy resistance. Precise identification of this translocation was possible with additional application of molecular cytogenetic methods mFISH and mBAND.

“Written, informed consent for publication was obtained from the patient [or parent/guardian for patients under 16]”

#### 2.P35 Cytogenetic abnormalities in uterine leiomyoma cells in vivo and in vitro

##### Alla S Koltsova^1^, Anna A Pendina^2^, Olga A Efimova^2^, Olga V Malysheva^2^, Natalia Yu Shved^2^, Thomas Liehr^3^, Moneeb A K Othman^3^, Maka I Kakhiani^4^, Vladislav S Baranov^2^

###### ^1^St. Petersburg State University Genetics and biotechnology St. Petersburg-Russia; ^2^D.O. Ott Research Institute of Obstetrics, Gynecology and Reproductology Laboratory for prenatal diagnosis of congenital and inherited diseases St. Petersburg-Russia; ^3^Universitätsklinikum Jena Institut für Humangenetik Jena-Germany; ^4^D.O. Ott Research Institute of Obstetrics, Gynecology and Reproductology Department of Operative Gynecology St. Petersburg-Russia

####### **Correspondence:** Alla S Koltsova (rosenrot15@yandex.ru)

Uterine leiomyomas (ULs) are benign tumors which are characterized by high frequency of chromosomal abnormalities. We aimed to compare the frequency of cells with chromosomal abnormalities between cultured and non-cultured samples of the same ULs. A total of 32 UL cultures were karyotyped. Twelve out of 32 (38%) UL cultures were karyotypically abnormal. In these ULs, using interphase fluorescence in situ hybridization with DNA probes specific to revealed chromosomal abnormalities, we compared the frequencies of karyotypically abnormal cells in cultured and non-cultured samples. In most cases, the frequencies of karyotypically abnormal cells differed significantly between cultured and non-cultured samples of the same ULs. The frequencies of cells with 47,XX,+12, 46,XX,t(6;10;16)(p21;q22;p13) and 46,XX,del(7)(q21.11q36),t(12;14)(q15;q23) karyotypes were significantly higher in cultured compared to non-cultured samples. In contrast, the frequencies of cells with 46,XX,del(7)(q21.11q22.3), 46,XX,t(4;10;12;?)(p11;q22;q15;?) and 46,XX,t(12;14)(q15;q23) karyotypes as well as with chromothripsis involving chromosomes 1, 8, 14 were significantly lower in cultured compared to non-cultured samples. The frequency of cells with 46,XX,inv(1)(p22p36),t(1;10)(p36;q26) karyotype did not differ significantly between cultured and non-cultured samples. Our findings suggest that UL cultures consist of cytogenetically heterogeneous cell populations, some of which are characterized by a selective growth.

#### 2.P36 Frequency of secondary chromosomal abnormalities and impact on the prognosis of patients with acute myeloid leukemia and translocation t(8;21)(q22;q22); RUNX1 RUNX1T1 in a Peruvian Oncological Institute. 2005 2013

##### Richard Zapata Zapata-Dongo^1^, Yesica Llimpe M.^2^, Luis Rodríguez D.^1^

###### ^1^Universidad Nacional Pedro Ruíz Gallo, Facultad De Ciencias Biológicas, Lambayeque-Peru; ^2^Universidad Nacional Mayor De San Marcos, Facultad De Medicina, Lima-Peru

####### **Correspondence:** Richard Zapata Zapata-Dongo (richardzapata1303@gmail.com)

Patients with Acute myeloid leukemia and genetic translocation t(8;21)(q22;q22); RUNX1/RUNX1T1 are clinically associated with good prognosis. However, other abnormalities accompanying to this translocation named secondary chromosomal abnormalities (SCAs) have also been described. It is believed that these findings could modified the prognosis of patients. In our retrospective study (conducted during 2005 and 2013) in a Peruvian Oncological Institute, 50 patients with SCAs were selected. The loss of one sex chromosomes (LSC) (n=36; 72%) and deletion on chromosome 9: del(9)(q22) (n=7;14%) are among the most frequent SCAs. Other less frequent SCAs were numerical alterations (n=11; 22%): +4, +5,+7, +8, -14 and +21, structural abnormalities (n=8; 16%) such as t(1;10), add(2), der(3), t(6;13), del(7), ins(9), del(11), del(15) and del(21). On the other hand, the impact generated by the presence of the SCAs related to prognosis showed that the prognosis of patients < 15 years and > 60 years with SCAs remains vulnerable (n=12; 67% dead patients), whereas the prognosis of patients aged 31 to 45 years with SCAs did not show a significant impact (n=7; 14% dead patients). In addition, patients with complex karyotypes (n=2) and numerical SCAs (not LSC) (n=2) exhibited an adverse prognosis, while patients with only LSC (n=23; 46%), autosomal structural SCAs (n=7; 14%) not modified the prognosis. In summary, our results suggest that the presence of SCAs plays an important role in the prognosis of patients according to their ages and complexity of the chromosomal abnormalities.

#### 2.P37 Spectrum of cytogenetic abnormalities detected by fluorescence in situ hybridization A retrospective study of 1140 AML patients from India

##### Hemani Jain^1^, Dhanlaxmi Shetty^1^, Hasmukh Jain^2^, Bhausaheb Bagal^2^, Avinash Bonda^3^, Sachin Punatar^3^, Anant Gokarn^3^, Nayak Lingraj^3^, P. G. Subramanian^4^, Gaurav Narula^2^, Manju Sengar^2^, Navin Khattry^3^, Sripad Banavali^2^

###### ^1^Advanced Centre For Treatment, Research & Education in Cancer (ACTREC), Tata Memorial Centre, Cancer Cytogenetics Department, Navi Mumbai-India; ^2^Tata Memorial Hospital, Department of Medical Oncology, Mumbai-India; ^3^Advanced Centre for Treatment, Research & Education in Cancer (ACTREC),Tata Memorial Centre, Department of Medical Oncology, Navi Mumbai-India; ^4^Advanced Centre for Treatment, Research & Education in Cancer (actrec), Tata Memorial Centre, Hematopathology Department, Navi Mumbai-India

####### **Correspondence:** Hemani Jain (hemanijain7@gmail.com)

Introduction

Identification of underlying genetic aberrations helps in classification of acute myeloid leukemia (AML) into favourable, intermediate and adverse risk groups. The aim of the study was to check the efficacy of FISH in detection of recurrent and rare cytogenetic aberrations in Indian AML patients, thus aiding in risk stratification.

Material and Methods

Retrospective study was conducted on 1140 AML patients (Jan 2017-Dec 2018) at a tertiary cancer centre from India. FISH analysis included probes for RUNX1-RUNX1T1, KMT2A, CBFB, BCR-ABL1, +8, -5, -7, del 5q, del 7q and TP53 deletion.

Results

Of 1140 AML patients, 247 (22%) were pediatric, 893 (78%) were adult and 254/1140 (22%) were cytogenetically abnormal. Incidence of RUNX1-RUNX1T1, CBFB and KMT2A rearrangements was lower in adult AML as compared to pediatric patients (11%, 3% and 5% vs 28%, 5% and 11% respectively) while chromosome 5 aberrations was higher in adult (5% vs 2%). Monosomy 7/del(7q) was detected in 8% adult and 9% pediatric patients. Twenty-four (2%) adult patients exhibited TP53 deletion. BCR-ABL1 fusion (1%) and trisomy 8 (9%) was prevalent in adult. FISH detected additional cryptic and rare aberrations like ABL1 amplification, MLL amplification, duplication of MLL gene, monoallelic deletions, rearrangement of ABL1, RUNX1, RUNX1T1, i(5p), i(7q) and t(7q). Detection of monosomies with/without structural aberrations helped in prediction of monosomal or complex karyotypes.

Conclusion

FISH could efficiently detect recurrent cytogenetic abnormalities as well as rare cryptic aberrations associated with poor risk. It also helped in speculation of monosomal and complex karyotypes thus helping in prognostication and classification of disease. As FISH panel testing has higher resolution than conventional cytogenetic analysis, rapid turnaround time, and a considerable diagnostic and prognostic utility, it should be used as an adjunct to conventional karyotyping.

#### 2.P38 A unique peripheral blood karyotype characterized by multiple complex chromosomal translocations is caused by homozygosity for the CHEK2 p.Gly167Arg variant

##### Tamar Paperna^1^, Nitzan Sharon-Shwartzman^1^, Alina Kurolap^1^, Yael Goldberg^2^, Nivin Moustafa^1^, Yariv Carasso^3^, Miora Feinstein-Linial^2^, Adi Mory^1^, Gili Reznick-Levi^1^, Claudia Gonzaga-Jauregui^4^, Alan R. Shuldiner^4^, Lina Basel-Salmon^2^, Yishai Ofran^3^, Elizabeth E. Half^5^, Hagit Baris Feldman^1^

###### ^1^Rambam Health Care Campus, The Genetics Institute, Haifa-Israel; ^2^Rabin Medical Center, Raphael Recanati Genetics Institute, Petach Tikva-Israel; ^3^Rambam Health Care Campus, Department of Haematology and Bone Marrow Transplantation, Haifa-Israel; ^4^Regeneron Pharmaceuticals, Regeneron Genetics Center, Tarrytown, Ny-United States; ^5^Rambam Health Care Campus, Department of Gastroenterology, Haifa-Israel

####### **Correspondence:** Nivin Moustafa (n_moustafa@rambam.health.gov.il)

Introduction: Cancer cells are often characterized by chromosomal instability; therefore, mutations in genes involved in DNA repair may lead to aberrations that confer cancer susceptibility. We describe two patients: Patient 1 presented with primary multi-organ tumorogenesis, while Patient 2 had early-onset acute myeloid leukemia. Karyotype analysis of both patients revealed a unique phenotype of multiple chromosomal abnormalities in peripheral blood lymphocytes.

Materials and Methods: Diagnostic measures for the patients included TruSight One (TSO) panel and whole-exome sequencing (WES) for Patient 1, and FoundationOne® Heme genomic analysis for Patient 2. The candidate variant was confirmed in the patients and available family members by Sanger sequencing. Karyotype analysis was performed on peripheral blood, bone marrow and other tissues, as available.

Results: Both patients, who are reportedly unrelated, were homozygous for the CHEK2 c.499G>A; p.Gly167Arg variant, located in the FHA domain of the protein. Karyotype analysis of peripheral blood lymphocytes revealed 30-60% cells with multiple different chromosomal translocations, which were not observed in bone marrow, fibroblasts and renal cells. Karyotyping of a cancer patient with homozygosity for a different CHEK2 variant: p.Ser428Phe, located in the kinase domain, revealed only one cell (out of 30) with a balanced translocation between the short arms of chromosomes 7 and 14.

Conclusions: CHK2, the protein encoded by the CHEK2 gene, is essential for double-strand break and homologous recombination repair. This role is supported by our findings of multiple chromosomal translocations observed in lymphocytes from both patients with homozygous CHEK2 p.Gly167Arg. We suggest that FHA domain function, which is required for CHK2 dimerization, is critical for protein function, so that its disruption impairs proper correction of DNA breaks, leading to increased susceptibility to tumorigenesis.

#### 2.P39 P1143 - De novo acute myeloid leukemia with BCR ABL1 accompanied by t(8;21)(q22;q22.1) RUNX1 RUNX1T1 a rare association

##### Marija Dencic Fekete^1^, Ljubomir Jakovic^2^, Marijana Virijevic^2^, Jelica Jovanovic^2^, Biljana Todoric-Zivanovic^3^, Teodora Karan Djurasevic^4^, Nada Kraguljac-Kurtovic^2^, Sonja Pavlovic^4^, Andrija Bogdanovic^2^

###### ^1^Institute of Pathology, Medical Faculty, Belgrade-Serbia; ^2^Clinic for Hematology, Clinical Center of Serbia, Belgrade-Serbia; ^3^Military Medical Academy, Military Medical Academy, Belgrade-Serbia; ^4^Institute of Molecular Genetics And Genetic Engineering, University of Belgrade, Belgrade-Serbia

####### **Correspondence:** Marija Dencic Fekete (marijadfekete@yahoo.com)

The issue of de novo Philadelphia positive (Ph+) acute myeloid leukemia (AML) and its distinction from blastic transformation of chronic myeloid leukemia (CML), has been a matter of debate for a long time. Revised WHO classification (2016) recognized AML with BCR-ABL1 as a provisional entity. Moreover, additional reccurent genetic abberations in this AML subtype are rare, with only three published cases of de novo AML with BCR-ABL1 with t(8;21) so far.

We present a 34 years old male patient, diagnosed as de novo AML with BCR-ABL1 and t(8;21). Diagnostic workup revealed leucocytosis (34x109/L), leukemic hiatus (blasts 84%) and thrombocytopenia (27x109/L). Splenomegaly and basophilia were absent. Bone marrow analysis showed presence of POX+myeloblasts with Auer rods (76% NC) and early myeloid immunophenotype (CD34+, CD117+, CD33+, CD13+, cMPO+, CD15+) with abberant expression of CD56+ and CD19+. Cytogenetical signature was 45,X,-Y,t(8;21)(q22;q22),t(9;22)(q34;q11)[18]/46,XY[2]. Concomitant presence of BCR-ABL and RUNX1-RUNX1T1 were confirmed by FISH and RT-PCR, also revealing a p190 bcr-abl transcript. Diagnosis of high-risk AML with BCR-ABL1/CD56+CD19+ was established. The induction chemotherapy “3+7” together with imatinib [400 mg/d], resulted in cytomorphological, cytogenetic and molecular remission. Moreover, minimal residual disease by flow cytometry was not detected in the bone marrow (<0.01% NC) and peripheral blood (<0.01% WBC, European LeukemiaNet criteria). Five months after diagnosis, the patient underwent matched allografting. He remained in complete molecular remission for both transcripts, 22 months after the allotransplant.

In our case, it might be speculated that t(8;21) can be due to clonal evolution of Ph+ clone. However, our findings including absence of splenomegaly, both aberrations present in the same metaphases, Ph+ detection in less than 100% of analyzed cells and the bcr-abl transcript type p190, strongly supports de novo AML with BCR-ABL1 with additional t(8;21).

“Written, informed consent for publication was obtained from the patient [or parent/guardian for patients under 16]”

#### 2.P40 Circulating microRNAs as potential biomarkers in paediatric acute lymphoblastic leukaemia

##### Andrea Rzepiel^1^, Nóra Kutszegi^1^, Bálint Egyed^1^, András Gézsi^2^, Judit C. Sági^1^, Csaba Szalai^2^, Ágnes F. Semsei^2^, Dániel J. Erdélyi^1^

###### ^1^Semmelweis University 2nd Department of Paediatrics Budapest-Hungary; ^2^Semmelweis University Department of Genetics, Cell- and Immunobiology Budapest-Hungary

####### **Correspondence:** Andrea Rzepiel (rzepiel.andrea@semmelweis-univ.hu)

Acute lymphoblastic leukaemia (ALL) is the most common paediatric malignancy. Nowadays the overall survival is 85-90%. Minimal residual disease monitoring is part of the clinical routine diagnostics. Flow cytometry and PCR methods are currently used techniques with sensitivity of 10-3-10-4 and 10-4-10-6, respectively. MicroRNAs are of great interest in scientific research. Their role is not fully discovered especially not in paediatric leukaemia although altered microRNAs expression in many malignant conditions are established.

The aim of this study was to determine if a set of microRNAs are associated with paediatric ALL, different karyotypes and changes during the treatment correlated with progression of disease. Peripheral blood platelet free plasma (PB PFP) samples were obtained from 28 newly diagnosed de novo and 5 relapsed paediatric ALL patients during the first months of therapy. Following RNA isolation (Qiagen miRNeasy kit), quantification of literature based previously selected 46 miRNAs was performed using Custom TaqMan Advanced miRNA Assay (TLDA). Higher expression of all the 4 miRs identified on TLDA were validated on the expanded cohort by qPCR. Statistical analyses were carried out using R statistical program.

Expression of 19 miRs in ALL PB PFP at diagnosis were significantly different compared to control samples (p<0.05) measured by TLDA.

The expression of all four miRs in PB PFP decreased significantly by day 8 during the first months of therapy measured by qPCR. Two studied microRNAs’ expression changes between day 0 and day 8 correlated with day 15 bone marrow flow cytometry MRD assessment. In normal karyotype subgroup the same observation was found.

Expression changes of microRNAs were observed in paediatric ALL patients’ PB PFP. One microRNA’s expression changes by day 8 of therapy was correlated with day 15 flow cytometry MRD assessment. The suggested role of microRNA as a potential biomarker is proposed.

### 3. Prenatal Diagnosis

#### 3.P1 Prenatal Chromosomal Microarray Analysis (CMA) Experience of a Cytogenetics Laboratory in Singapore

##### Jaymie Lacsamana^1^, Mui Li Tan^1^, Lee Tian Toh^1^, Hui Yi Yon^1^, Sharon Ling^1^, Angeline Lai^2^, Tai Wai Yeo^3^, Min Hwee Yong^1^

###### ^1^Kk Women's and Children's Hospital, Department of Pathology And Laboratory Medicine, Singapore-Singapore; ^2^Kk Women's and Children's Hospital, Genetics Services, Department of Paediatrics, Singapore-Singapore; ^3^Kk Women's and Children's Hospital, Department of Fetal Maternal Medicine, Singapore-Singapore

####### **Correspondence:** Jaymie Lacsamana (jaymie_912@icloud.com)

Aim

CMA is becoming the frontline diagnostic test for prenatal specimens because of its increased diagnostic yield. About 1-2% of pregnancies with advanced maternal age and 6.5% with an abnormal ultrasound detected will have a significant clinical finding on CMA after a normal karyotype. Clinical guidelines have recommended CMA as the first line of diagnostic testing following the detection of a fetal anomaly. Being the first Cytogenetics laboratory to implement prenatal CMA diagnostically in Singapore, we aim to document our experience since its launch in October 2018.

Methods

DNA was extracted from prenatal amniotic fluid samples using a modified protocol from Puregene Cell Kit. CMA using Affymetrix CytoScanTM 750K Array Kit was performed on these DNA samples. For specimens with presence of red blood cells, maternal DNA extracted from an EDTA tube and the fetal DNA are sent for maternal cell contamination test. Analysis is done using Chromosome Analysis Suite 3.2 (ChAS)software.

Standard G-band analysis using Ikaros (Metasystems) was done for specimens with additional karyotyping requests.

Results

Out of 48 samples analysed, 42 showed a normal result and two showed an abnormal CMA result. The remaining four had variants of uncertain significance. The clinical indication for the two cases with abnormal results was increased Trisomy 21 risk from their combined First Trimester Screening. Karyotyping results for one abnormal case was apparently normal and one is consistent with Klinefelter.

Conclusion

With CMA being the recommended test for prenatal diagnosis, implementing CMA has added value to our Cytogenetics services. CMA, performed regardless of clinical indications, was able to detect clinically significant copy number changes that could have been missed if karyotyping alone was done. This proves that CMA improves our diagnostic yield and improves the quality of care for patients.

#### 3.P2 Bifid cardiac apex in pallister killian syndrome case report

##### Anita BarišiĆ^1^, Aleks Finderle^2^, Oleg PetroviĆ^2^, Nada StarČeviĆ ČizmareviĆ^1^, Sa a OstojiĆ^1^, Jadranka VranekoviĆ^1^

###### ^1^Faculty of Medicine, University of Rijeka, Department of Medical Biology And Genetics, Rijeka-Croatia; ^2^Clinical Hospital Center, Department of Obstetrics and Gynecology, Rijeka-Croatia

####### **Correspondence:** Jadranka VranekoviĆ (jadranka.vranekovic@uniri.hr)

Background: Pallister-Killian syndrome (PKS) is a sporadic, rare chromosomal disorder, caused by tissue-limited mosaicism for an isochromosome 12p (i12p). Prenatal diagnosis of PKS is generally incidental at karyotyping in case of fetal anomaly detection or advanced maternal age. Although clinical presentation of PKS varies, cytogenetic findings are constant, and include a tetrasomy of chromosome 12p diagnosed by chorionic villus sampling, amniocentesis or cordocentesis. We report a case of prenatally diagnosed PKS with unique dysmorphic feature: bifid cardiac apex, a type of morphology that has not been documented ever before in the literature. Case report:. Our patient is the 38-year-old pregnant woman who underwent amniocentesis at 18 weeks and 1 days’ gestation upon of an ultrasonographic imaging of fetal cleft lip and advanced maternal age. Cytogenetic analysis of amniotic fluid detected a male mosaic karyotype with a supernumerary chromosome (SMC) in 18/28 (64%) of fetal amniocytes. To determine the chromosomal origin of SMC, fluorescence in situ hybridization (FISH) was performed on metaphase spread chromosomes and mosaicism of tetrasomy 12p was confirmed: mos 47,XY,+mar[18]/46,XY[10].ish i(12p)(8M16/SP6++,CEP12+,VIJyRM2196-). Ultrasound examination showed a male fetus with abnormal facial profile, an echogenic focus in the left ventricle of the heart and shortened fetal long bones. After receiving a genetic counseling for established diagnosis of PKS, the woman requested a termination of pregnancy due to medical reasons. A postmortem inspection and autopsy of the fetus revealed a complex heart anomaly that includes bifid cardiac apex and ventricular septal defect. Conclusion: This report expands the clinical manifestations of PKS with a unique feature of bifid cardiac apex, and highlights the targeted diagnosis of Pallister-Killian syndrome in a second and third trimester of pregnancy if specific ultrasound markers are present.

#### 3.P3 Prenatal diagnosis of aneuploidy in twins

##### Oksana Vashchenko, Iryna Gordienko, Tetiana Nikitchina, Ganna Grebinichenko, Olena Tarapurova, Andriy Velychko

###### Si «institute of Pediatrics, Obstetrics and Gynecology Named After Academician O.lukyanova of The Nams of Ukraine», Department of Fetal Medicine, Kyiv-Ukraine

####### **Correspondence:** Oksana Vashchenko (zoa_83@ukr.net)

Objective: To analyze results of the ultrasonographic (US) and karyotyping data in twins from high risk pregnancies.

Material and methods. There were 152 high risk pregnant women with twins who underwent invasive procedures for fetal karyotyping. Among those prenatally diagnosed: 85 (55.9 %) spontaneous dichorionic-diamniotic twins, 40 (26.3 %) monochorionic diamniotic, 18 (11.8 %) dichorionic-diamniotic twins conceived by ART and 9 (5.9 %) monochorionic monoamniotic. In all cases expert US exam was performed. Indications for karyotyping were: high risk of chromosomal pathology (CP) because of combined I trimester screening or US markers in 41.4% of cases, fetal structural malformations (SM) in 40.8%, maternal age in 17.8%. Karyotyping of 247 (81.3%) of 304 fetuses was available. For cytogenetic analysis of chorion/placenta biopsy samples direct method of villi processing was used, for fetal blood we used half-micromethod.

Results. CP was diagnosed in 9 of 247 fetuses (3.6%). There were 3 cases of trisomy 21 (33.3%), 2 of triploidy (22.2%), 2 of Klinefelter syndrome (22.2%), 1 of X-monosomy (11.1%) and 1 of trisomy 18 (11.1%). Among the 3 cases of trisomy 21, in 1 fetus SM were present, 2 others had US markers in favour of CP. US markers of I trimester were present both in 1 case of triploidy and in trisomy 18 case. There were fetal SM in the 2nd case of triploidy and in fetus with X-monosomy.

Conclusion. The analysis of the results of prenatal diagnosis showed that in the examined twin fetuses the structural anomalies (40.8%) were much more often diagnosed than chromosomal pathology (3.6%). Thus, not all structural anomalies can be taken as indications for karyotyping.

#### 3.P4 Chromosomal mosaicism in human chorionic villus sample

##### Nadia Voskoboinik^1^, Daniel Konik^1^, Smadar Yogev^1^, Liat Cohen^1^, Hila Kolog^1^, Sasha Emshanov^1^, Idit Kaplan Ber^1^, Tova Naiman^1^, Rotem Grinberg^1^, Avi Orr-Urtreger^2^, Yuval Yaron^2^, Anat Bar-Shira^1^

###### ^1^Tel Aviv Sourasky Medical Center, Genetic Institute, Tel Aviv-Israel; ^2^Tel Aviv Sourasky Medical Center, The Sackler Faculty Of Medicine, Tel Aviv University, Genetic Institute, Tel Aviv-Israel

####### **Correspondence:** Nadia Voskoboinik (nadiavosko@gmail.com)

Molecular cytogenetic techniques as noninvasive prenatal testing (NIPT), fluorescence in situ hybridization (FISH), quantitative fluorescence polymerase chain reaction (QF-PCR) and chromosomal microarray analysis (CMA) allow diagnosis of chromosomal aberrations on uncultured cells, but are also limited as to the spectrum of chromosomal abnormalities and mosaicism detected.

We report the clinical case of a 37-year-old pregnant women who was referred to our institute with an abnormal NIPT result- suspected trisomy of chromosome 21.

A chorionic villus sample was taken at 11 weeks gestation.

QF-PCR analysis was undertaken for the rapid detection of aneuploidy for chromosomes 13,18,21,X and Y on uncultured cells. QF-PCR results showed trisomy 21. Trisomy 21 was confirmed also by CMA analysis on non-cultured cells.

Trisomy 21 was not found by further chromosome analysis on short term cultures: direct karyotyping of cytotrophoblasts was normal for female fetus in ten metaphases - 46,XX. But cultured chorionic villus mesenchymal cells showed trisomy 21 in ten metaphases- 47,XX,+21. Cytogenetic analysis revealed mosaicism with two cell lines: one with trisomy of chromosome 21 in long cell cultures and another with normal karyotype in short term cultures (direct method). FISH study, using probe specific for chromosome 21, was done on long term cell cultures in order to characterize the cytogenetic findings. Our FISH study confirmed the cytogenetic findings - fetoplacental chromosomal mosaicism for trisomy of chromosome 21; 86% of the cells were with trisomy 21 and 14% - were normal.

Cytogenetic and FISH analysis of both parents demonstrated normal results.

Conclusion: in our case NIPT, QF-PCR, CMA results and karyotype of mesenchymal cells were abnormal, but karyotype of cytotrophoblasts was normal. Our cytogenetic results confirmed fetoplacental mosaicism and demonstrate that karyotype of trophoblasts is not always representative for the fetal karyotype.

“Written, informed consent for publication was obtained from the patient [or parent/guardian for patients under 16]”

#### 3.P5 SNP microarray – our experience in prenatal diagnosis

##### Anca Pavel^1^, Luiza Dimos^2^, Eliza Rentea^1^, Andreea Tutulan-Cunita^1^, Anca Mocanu^1^, Danai Stambouli^2^

###### ^1^Cytogenomic Medicallaboratory, Molecular, Bucharest-Romania; ^2^Cytogenomic Medicallaboratory, Cytogenetics, Bucharest-Romania

####### **Correspondence:** Anca Pavel (dimos@cytogenomic.ro)

Currently, in our laboratory in Romania, the diagnostic power of microarray based techniques in prenatal diagnosis, has gained the trust of obstetricians. The requests have greatly increased since the beginning of 2013, especially in those pregnancies with echographical findings.

In 2018, we analyzed by SNP microarray 335 (45%) out of 730 prenatal samples, the rest being tested using conventional methods. Based on the indication, a preferred type of analysis was registered: out of 730 cases, 44% were referred for echographical findings, out of which, over half were sent for SNP microarray. On the other hand, 30% were referred for high risk after biochemical screening and from these only in one fourth SNP microarray was requested. Regarding advanced maternal age, no preferential type of analysis was observed.

DNA was extracted from chorionic villus samples and amniotic fluid cells of 335 prenatal cases. Molecular karyotyping analysis was performed using CytoScan 750K Arrays, GeneScan 3000 (Applied Biosystems by ThermoFisher Scientific) and the data were analyzed by Chromosome Analysis Suite (ChAS) software (ThermoFisher Scientific).

In 38 (11%) out of 335 SNP cases, a clinically relevant genetic abnormality was detected. In 21 (55%) out of those 38, a chromosomal aberration was detected, whereas in the other 17 (45%) cases, submicroscopic abnormalities were found.

In conclusion, SNP microarray, providing fast and high resolution results, has started to become a tier 1 diagnostic test in the cases of high-risk pregnancies for the Romanian medical community, despite the high cost.

#### 3.P6 Molecular characterization of a structural abnormal Y chromosome that showed conflicting results by QF PCR and conventional karyotyping

##### Marta Pinto^1^, Ana Jardim^1^, Luis Miguel Pires^1^, Nuno Lavoura^1^, Patricia Paiva^1^, Isabel Carreira^2^

###### ^1^Faculty of Medicine, University of Coimbra, Cytogenetics and Genomics Laboratory, Coimbra-Portugal; ^2^ICBR-Cimago - Center of Investigation on Environment Genetics and Oncobiology - Faculty of Medicine, Cytogenetics and Genomics Laboratory, Coimbra-Portugal

####### **Correspondence:** Isabel Carreira (mrtpinto@gmail.com)

Human Y chromosome harbours the essential genes for male sex determination, early sexual differentiation and control of spermatogenesis, therefore structural anomalies of this chromosome are particularly hazardous for male reproductive function.

We present a prenatal case of a structural abnormal Y chromosome in which analyses by QF-PCR for the rapid screening for common aneuploidies and classical cytogenetics revealed apparently discordant results. The molecular assay was compatible with a Y chromosome disomy while the classical cytogenetics showed an apparently normal male karyotype.

Considering the results, CBG banded Y chromosomes were also analyzed and no staining pattern for the heterochromatic region was observed. To clarify the alteration, FISH, using the SRY and centromeric probes, was performed. This analysis identified a duplication of the SRY locus, confirming the result previously obtained by QF-PCR, and the presence of two Y chromosome centromeres. To delineate the duplicated and deleted regions involved, oligoarray-CGH was carried out. A duplication of all the short arm Y(p11.32p11.2) and part of the long arm Y(q11.222q11.23) and deletions of the long arm Y(q11.222q11.23) and region Y(q12) were observed.

This male fetus has a duplication on Yq that involves the AZFa region and a Yq deletion involving the AZFb and AZFc regions, therefore, the growth and development of sperm is expected to be compromised.

The der(Y) was not identified by classical karyotyping. Nevertheless the QF-PCR, alone, would also lead to an incorrect diagnosis. The FISH and aCGH helped us to solve the conflicting results allowing the characterization of the abnormal chromosome that would be missed by conventional cytogenetics, which is still the most common test after a positive combined prenatal screening.

#### 3.P7 Detection of multiples CNVs and complex chromosomal restructuration in a sole chromosome in two prenatal cases matching criteria for chromoanasyntesis

##### Elisabet Lloveras, Anna Canellas, Laura Barranco, Marta Costa, Cristina De La Iglesia, Begoña Mendez, Nuria Palau, Daniel Fernandez, Meritxell Pique, Diana Yeste, Marc Auge, Marta Herrero, Laia Puig, Susana Martin, Cristina Perez

###### Laboratori Central Barcelona. Synlab International Group., Departament De Genètica, Esplugues De Llobregat-Spain

####### **Correspondence:** Elisabet Lloveras (elilloveras70@gmail.com)

Introduction: In 2011, a new mechanism of complex chromosomal restructuring called chromothripsis is described for the first time. According to this hypothesis, due to a cellular stress of unknown origin, the pulverization of all or part of a chromosomal followed by a chaotic regrouping of the fragments would take place. Recently it has been shown that this chaotic process of fragmentation could also generate complex restructurings with the presence of multiple variations of copy number, mainly duplications and triplications. This process has been described as chromoanasynthesis.

We present two cases with complex restructuring involving multiple CNVs restricted to a single chromosome and that could be associated with the recent phenomenon described as chromoanasynthesis.

Patients and methods:

Case 1. Pregnant woman who underwent prenatal diagnosis because of increase nuchal translucency (NT 7 mm), dilation of the renal pelvis and single umbilical artery. QF-PCR, arrayCGH and karyotype were performed. The result of arrayCGH showed the presence of multiple CNVs on chromosome 18, reorganized in a complex way as observed in the karyotype study.

Case 2. Pregnant woman who underwent prenatal diagnosis for nasal bone hypoplasia, 3mm NT and short (Crown-to-Rump Length) CRL. QF-PCR, arrayCGH and karyotype were performed. The study of the karyotype showed a chromosome 1 short arm with a de novo atypical band pattern. Multiple CNVs in a fragment of chromosome 1p were detected by arrayCGH.

Comments:

New technologies, as arrayCGH, could allow the detection of rare phenomena of complex chromosomal rearrangements.

The genetic counselling for chromoanasyntesis can be a challenge in prenatal diagnosis. Despite the lack of phenotypic association with the copy number variation detected, the presence of many breakpoints could imply a high risk of phenotypic alterations.

#### 3.P8 CMA re evaluation of previous chromosomal studies in the context of new consultations by families allows improvement of genetic counselling

##### Carmen Mediano^1^, Anna Gassol^1^, Eulalia Rovira^1^, Lourdes Trobo^1^, Neus Castells^1^, Maite Aviles^2^, Elena Garcia-Arumi^1^, Eduardo Tizzano^1^

###### ^1^Hospital Vall D'hebron, Unitat De Genètica, Barcelona-Spain; ^2^Hospital Vall D'hebron, Unitat De Diagnòstic Prenatal, Barcelona-Spain

####### **Correspondence:** Carmen Mediano (carmenmediano@yahoo.es)

Chromosomal microarray analysis (CMA) is the best available tool to detect copy number variations in chromosomal make up. In two independent families with apparently closed clinical cases by conventional kartotype analysis, we re-evaluated their results using CMA.

Family 1: A pathological CMA in a malformed fetus led to the finding of a reciprocal translocation t(5;12)(q35.1;p13.3) in the mother and also in the grandmother. A brother of this grandmother had documentation of a previous diagnosis of reciprocal translocation t(5;6)(q35.1;q27). The new studies performed in this side of the family showed the correct translocation detected in our lab t(5;12)(q35.1;p13.3) in the brother and his children.

Family 2: A dizygotic twins’ pregnancy was analysed by CMA in chorionic villi due to increased nuchal translucency in one of the fetuses. A concurrent 2p duplication and 9p deletion was detected suggesting an unbalanced derivative of a t(2;9)(p;p) reciprocal translocation. Karyotype confirmed the chromosomal rearrangement and further cytogenetic studies in the parents demonstrated a maternal t(2;9)(p25.1;p22.2) reciprocal translocation. The normal fetus was a carrier of the balanced maternal translocation. The 30 years’ old mother carrier of the translocation had a previous report of cytogenetic study performed in amniotic fluid when she was a fetus with a result of normal 46,XX karyotype.

These two cases illustrate the importance to re-evaluate previous and apparently closed cases with new tools to allow a more precise or correct cytogenetic diagnosis with new implications in genetic counselling. Thus, in the first case two apparently different reciprocal translocations in a same family ended in just one and better defined t(5;12)(p;q) translocation. The correct diagnosis in family 2 changed the future reproductive expectations of the mother as her “normal” previous karyotype was in fact a reciprocal translocation.

“Written, informed consent for publication was obtained from the patient [or parent/guardian for patients under 16]”

#### 3.P9 Array comparative genomic hybridization with enhanced exon level CNV coverage detects a significant number of clinically relevant chromosome abnormalities in normal fetal ultrasound pregnancies

##### Joan Colomer^1^, Neus Castells^1^, Alberto Plaja^1^, M. Ángeles Sánchez^2^, Eulàlia Rovira^1^, Anna Abulí, Clara Serra^1^, Encarnación Oliveros^1^, Maria Serrano^1^, Desirée Martínez^1^, M. Guadalupe Gala^1^, Carlota Rodó^2^, M. Teresa Avilés^2^, Teresa Higueras^2^, Manuel Mendoza^2^, Carmen Mediano^1^, Maria Antolin^1^, Elena Garcia-Arumí^1^, Eduardo Tizzano^1^

###### ^1^Hospital Vall D'hebron, Àrea De Genètica Clínica I Molecular, Barcelona-Spain; ^2^Hospital Vall D'hebron, Servei De Obstetricia I Medicina De La Reproducció, Barcelona-Spain

####### **Correspondence:** Joan Colomer (colomer.joan@gmail.com)

INTRODUCTION: Following a normal karyotype, chromosomal microarray analysis (CMA) identifies an additional significant chromosomal abnormality in 4.7-6.0% of pregnancies with at least 1 major structural ultrasound abnormality, and an additional 1.7-2.5% of pregnancies with other indications for testing. The American College of Obstetricians and Gynecologists (ACOG) and the Society for Maternal‐Fetal Medicine (SMFM) recommend CMA in the setting of 1 or more structural fetal anomalies identified by ultrasound and CMA or karyotyping” in the setting of a normal fetal ultrasound. This study evaluates CMA detection rate of clinically significant chromosomal abnormalities in both groups and the incidence of exon-level copy number variants (CNVs).

METHODS: 926 pregnancies with a normal QF-PCR result were evaluated with the Oxford Gene Technology CytoSure Constitutional 8 × 60K v3 comparative genomic hybridization assay with enhanced exon-level CNV coverage of 354 developmental disorder genes. 289 pregnancies had one or more structural fetal anomalies identified by ultrasound and 637 had a normal fetal ultrasound.

RESULTS: 27/289 pregnancies (9.34%) with abnormal ultrasound and 15/637 (2.35%) with normal ultrasound had a pathogenic chromosomal abnormality. Exon-level information was relevant in 5 fetuses (four of them with normal ultrasound findings). CNVs detected by CMA of less than 5–10 Mb were considered no detectable by karyotyping and account for 51,9% of the total (14/27) in abnormal ultrasound group and 40% (6/15) in normal ultrasound group.

CONCLUSIONS: A significant number of clinically relevant chromosome abnormalities would be missed if all pregnancies with normal ultrasound findings have only a karyotype performed. Exon-level CNV information can be especially relevant in the group of normal ultrasound pregnancies.

#### 3.P10 Circulating fetal cells for aneuploidies screening by ‘dual probe FISH’ analysis in pregnant women at high risk

##### Giuseppe Calabrese^1^, Giulia Sabbatinelli^2^, Donatella Fantasia^3^, Melissa Alfonsi^4^, Elisena Morizio^1^, Chiara Palka^1^, Claudio Celentano^5^, Paolo Guanciali-Franchi^1^, Giammaria Sitar^6^

###### ^1^University of Chieti, DSMOB-Medical Genetics, Chieti-Italy; ^2^University of Chieti, Imaging and Clinical Sciences, Chieti-Italy; ^3^Asl Pescara, Oncohematological Genetics, Pescara-Italy; ^4^Asl Chieti, Medical Genetics, Chieti-Italy; ^5^Asl Chieti, Obstetrics & Gynecololy, Chieti-Italy; ^6^University of Pavia, Policlinico S Matteo, Pavia-Italy

####### **Correspondence:** Giuseppe Calabrese (g.calabrese@dsb.unich.it)

Background: Rare circulating fetal cells in the mother blood have a strong potential to be target for non-invasive prenatal testing (NIPT) during the first trimester, but it has not yet been achieved a rapid, simple, consistent, and low-cost procedure suitable for routine clinical practice. Previously, we have developed ‘dual-probe FISH’ protocol on fetal cells isolated from maternal blood for detection of possible fetal aneuploidies (Calabrese et al 2012; Guanciali et al 2017).

Materials and methods: We offered ‘dual-probe FISH’ approach on circulating fetal cells to 68 pregnant women with singleton pregnancy who were at high risk for fetal aneuploidy using first trimester biochemical prenatal non-invasive screening (i.e. contingent test) and prior any invasive prenatal diagnosis (IPD) procedures. Dual-probe FISH analysis on interphase nuclei was carried out using different combinations of commercial dual/triple-color FISH probes for chromosomes 18 and 21. Slides were observed under a fluorescence microscope by direct visualization.

Results: The FISH analysis disclosed 51 cases with only diploid signals for chromosomes 21 and 18, and in 10 cases extra FISH signals were present. The 10 chromosome abnormalities comprised of 4 cases with trisomy 21, 4 trisomy 18 and 2 triploid. In all 68 cases, IPD confirmed either a normal karyotype or the chromosome abnormalities indicated by the FISH screening.

Conclusion: In pregnant women at high risk for aneuploidies following first trimester contingent screening test, the application of the dual-probe FISH protocol on fetal cells isolated from maternal blood allows an accurate molecular identification of fetal aneuploidy, providing a significant improvement of conventional screening tests to select pregnant women who would benefit most from IPD procedures, reducing costs and loss of fetuses.

#### 3.P11 Diagnostic performance of noninvasive prenatal testing (NIPT)

##### Rosa Maria Lobo^1^, Maria Talavera^2^, David Arrabal^1^, Maria America De Leon^1^, Jose Vicente Peñuelas^3^, Javier Guisasola^4^, Guadalupe Ruiz^1^

###### ^1^Hospital Rio Hortega, Genetic, Valladolid-Spain; ^2^Hospital Ramon Y Cajal, Genetic, Madrid-Spain; ^3^Hospital Santa Barbara, Gynecology, Soria-Spain; ^4^Complejo Hospitalario Burgos, Gynecology, Burgos-Spain

####### **Correspondence:** Maria Talavera (rmlvalentin@hotmail.com)

The screening of aneuploidies through the study of circulating fetal DNA is being implanted worldwide due to its high sensitivity and specificity, together with the high positive and negative predictive values. The aim of implantation is to reduce invasive techniques and the possibility of fetal losses as a result of false positives from classical screening and, as far as possible, to increase detection rates by setting the cutoff value above the classic 1/270.

MATERIAL AND METHODS

In the Autonomous Community of Castilla y León, this advanced test is performed sequentially to classic screening (maternal age + MoM free fraction β-hCG and PAPP-A + NT) that classifies pregnant women into three risk groups: high risk ( <1 / 100, direct invasive test), low risk (≥1000) and intermediate risk (1 / 100-1 / 1000, study in circulating fetal DNA).

RESULTS

1406 tests have been requested.

In 4% of the samples it was not possible to obtain results for reasons such as haemolysis, low fetal fraction, failure in the internal quality control of the sample or low number of readings.

The total number of samples with results has been 1349 (96%). 1327 have provided low risk results (98.4%) and of them 265 amniocentesis (up to 1/270) have been avoided.

Of the 22 reported high-risk samples, this has been confirmed in 16 pregnant women. A chromosomally normal karyotype (false positives, all with a combined screening value of 558, 214, 566, 928, 248, 239) has been reported in 6 samples.

CONCLUSIONS

The results obtained in this first year of implementation are in the upper range of non-informative samples and in terms of false positive results, it is necessary to take into account that the program covers 14 different hospitals that perform the classic screening with greater or less success.

The decrease of invasive tests on average by 65% is expected in the first year, but it is expected to increase in the following years.

Finally, it is important to highlight the increase of detection rates of T21 since, thanks to the program, 3 cases have been identified that would have been overlooked with traditional screening.

#### 3.P12 Pre and postnatal findings in a patient with a rec(8)(qter >q21.11 p23.3 >qter) due to a paternal inv(8)(p23.3q21.11)

##### Wisam Habhab^1^, Sylke Singer^1^, Angelika Rieß^1^, Karl Oliver Kagan^2^, Thomas Liehr^3^, Karin Schäferhoff^1^, Andreas Dufke^1^, Ulrike Mau-Holzmann^1^, Martin Kehrer^1^

###### ^1^University of Tuebingen, Institute of Medical Genetics and Applied Genomics, Tuebingen-Germany; ^2^University of Tuebingen, Department of Obstetrics and Gynecology, Tuebingen-Germany; ^3^Jena University Hospital, Friedrich Schiller University, Institute of Human Genetics, Jena-Germany

####### **Correspondence:** Wisam Habhab (Wisam.Habhab@med.uni-tuebingen.de)

BACKGROUND: Recombinant chromosome 8 (Rec8) syndrome (OMIM #179613), also known as San Luis Valley syndrome, is a rare chromosome disorder, that is associated with intellectual disability, congenital heart defects, variable skeletal and urogenital anomalies, and dysmorphic features. It is characterized by a partial terminal deletion of 8p and a partial terminal duplication of 8q, what is usually due to recombination of a pericentric inversion of chromosome 8 of a healthy parent. There are only very few reports of cases with breakpoints defined at the molecular level resp. by molecular karyotyping.

CASE PRESENTATION: We report a case of a partial monosomy 8p/trisomy 8q (recombinant chromosome 8) with previously unreported breakpoints in a fetus of non-consanguineous healthy parents with intrauterine growth retardation, hypogenesis of corpus callosum, bilateral cleft lip/palate and congenital heart defect.

METHODS: Chromosome G-banding analysis, FISH-analysis with subtelomeric und centromeric probe of chromosome 8 as well as SNP array (Affymetrix CytoScan Optima Array) were performed.

RESULTS: Cytogenetic analysis and molecular karyotyping revealed an abnormal chromosome 8 (karyotype: 46,XY,rec(8)(qter->q21.11::p23.3->qter) due to a paternal pericentric inversion of chromosome 8 (karyotype: 46,XY,inv(8)(p23.3q21.11).

CONCLUSION: To our knowledge, this is the third reported Rec8 case diagnosed prenatally. We describe the pre- and postnatal clinical findings and compare the phenotypic features with those of similar patients with Rec8 and mosaic trisomy 8 karyotype in the literature.

#### 3.P13 Trisomy 8 mosaicism in prenatal samples Management and Counseling

##### Cornelia Daumer-Haas, Daniela Köhler, Andrea Brans, Eva Kahles, Thomas Schramm, Sabine Minderer, Karl-Philipp Gloning

###### Praenatal-Medizin Muenchen, Mvz, Munich-Germany

####### **Correspondence:** Cornelia Daumer-Haas (daumer-haas@praenatal-medizin.de)

We present 25 cases of trisomy 8 mosaicism from a single center which were detected between 1997 and 2019, the largest series reported so far. All patients had chorionic villi biopsy with short and long term culture and a consecutive amniocentesis.

In 12 cases the mosaicism was limited to the cytotrophoblast (short term culture). Subsequent amniocentesis revealed only normal cells using FISH analysis with the centromeric probe in 50-200 analysed nuclei. Detailed sonografic investigations especially at 20/21 weeks of pregnancy were normal and no anomalies were reported in the born children.

In 13 cases mosaicism was present in the long term culture of the chorionic villi and in 3 of these FISH analysis of the uncultured amniotic fluid cells was abnormal. Only in one of these cases the pathological cell line was also detected in cultured cells. Two pregnancies with fetal abnormalities were terminated (14% abnormal nuclei and 4,3% abnormal nuclei in the FISH analysis) and one is still ongoing (2,9% nuclei abnormal) with reamniocentesis and umbilical blood sampling planned. In 10 pregnancies FISH results and results of the cultured amniocytes were normal with a favourable outcome.

Conclusion: In all patients with trisomy 8 mosaicism restricted to cytotrophoblast we found confined placental mosaicism. If the abnormal cell line was present in the long term culture the risk of having an abnormal FISH result in amniocytes was 23%. In these cases counseling is difficult especially if there is a low level mosaicism without significant sonografic fetal abnormalities. FISH analysis in repeated amniocentesis and umbilical cord blood sampling as well as detailed ultrasound is necessary for these cases.

#### 3.P14 Clinical use of chromosomal microarray analysis in detection of fetal chromosomal abnormalities

##### Sevilhan Artan^1^, Hasan Bas^1^, Mete Tanir^2^, Ebru Erzurumluoglu Gokalp^1^, Melih Velipasaoglu^2^, Sinem Kocagil^1^, Beyhan Durak Aras^1^, Mehmet Arda Temena^1^, Gulcin Panal^1^, Sara Khadem Ansari^1^, Oguz Cilingir^1^

###### ^1^Eskisehir Osmangazi University, Faculty of Medicine, Department of Medical Genetics, Eskisehir-Turkey; ^2^Eskisehir Osmangazi University, Faculty of Medicine, Department of Obstetrics And Gynecology, Eskisehir-Turkey

####### **Correspondence:** Sevilhan Artan (drhasanbas@hotmail.com)

Chromosome microarray analysis (CMA) is a genome-wide, high-resolution technique that can identify submicroscopic chromosomal abnormalities down to 20-to 100 kb level. Since the technique has a greater resolution than conventional karyotyping, it has been widely used for routine postnatal genetic diagnosis and there is a growing interest for arrays in the prenatal diagnosis. Here we present the CMA results of the prenatal diagnosis in our center. CGH+SNP Microarray (Agilent, 4x180) analyses were performed on chorionic villus sampling (4,7%), amniocentesis (93,3%) or fetal blood (2%) materials of the pregnancies with normal QF/PCR analyses results due to variety of indications. Of the received 255 samples, 56 likely benign (LB), variant of uncertain significance (VUS), likely pathogenic(LP) or pathogenic(P) copy number variants were found on 51 of the samples (22%). 19 patients (8.2%) had P/LP CNVs and one interesting finding was a paternal UPD of 6th chromosome. 32 (13.8%) had CNVs reported as VUS/LB. 68% patients with P/LP variants were referred with the indication of ultrasound abnormality (UA), 16% had increased risk on prenatal screening tests (PST) and 16% had both of UA and PST. The most common UA on patients with P/LP variants were fetal cardiac anomalies with a rate of 42,1%. Out of the patients with clinically significant CNVs, 16% had cytogenetically visible aberrations.

Therefore, this study highlights the value of CMA with an incremental yield of 6.3% over karyotyping (550 bands), especially on the pregnancies with fetal cardiac abnormalities. Moreover, SNP arrays are able to identify variants with loss of heterozygosity which can not be detected by conventional cytogenetics. In conclusion, CMA testing is efficient to improve diagnostic yield in prenatal diagnosis but the challenges to classify variants are still there and compelling.

#### 3.P15 The first case of prenatally detected NFIB deletion

##### Nusa Trost, Barbara Stojanov, Borut Peterlin, Luca Lovrečić

###### University Medical Center Ljubljana, Clinical Institute of Medical Genetics, Ljubljana-Slovenia

####### **Correspondence:** Nusa Trost (nusa.trost@gmail.com)

The haploinsufficiency of NFIB, located in the chromosomal region 9p23p22.3, has been recently proposed as a cause for macrocephaly-intellectual disability syndrome. Deletions ranging in size from 225 kb to 4.3 Mb have been reported in 10 individuals, with the smallest region of overlap of 107 kb, including only NFIB gene. The neurodevelopmental phenotype of these patients comprise mild intellectual disability or learning disability, motor and speech delay, behavioral anomalies, macrocephaly, and minor unspecific dysmorphic features. No data on prenatal development of these patients were provided. Also, there have been no prenatal cases reported so far.

Here we report on a case of prenatally detected NFIB deletion in a male fetus with increased nuchal translucency. The pregnant woman was referred to our department for genetic counselling and prenatal testing due to increased nuchal translucency. No other ultrasound anomalies were reported. Molecular cytogenetic analysis revealed a de novo interstitial deletion of 1.8±0.1Mb including NFIB gene. Post mortem examination showed no additional anomalies of the fetus. Increased nuchal translucency is a common marker for chromosomal aneuploidies and monogenic diseases, as well as structural heart anomalies. The later was excluded in here reported case. We suggest that the increased nuchal translucency might be related to deletion reported here, but further cases need to be described to determine the prenatal phenotype of NFIB haploinsufficiency.

#### 3.P16 Multiple structural chromosomal aberrations in a fetus with holoprosencephaly median cleft lip palate nasal agenesy and hypotelorism

##### Sylke Singer^1^, Wisam Habhab^1^, Karin Schaeferhoff^1^, Martin Kehrer^1^, Ute Grasshoff^1^, Andreas Dufke^1^, Karl Oliver Kagan^2^, Thomas Liehr^3^, Ulrike A. Mau-Holzmann^1^

###### ^1^University of Tuebingen, Institute of Medical Genetics and Applied Genomics, Tuebingen-Germany; ^2^University of Tuebingen, Department of Obstetrics and Gynecology, Tuebingen-Germany; ^3^University of Jena, Institute of Human Genetics, Jena-Germany

####### **Correspondence:** Sylke Singer (sylke.singer@med.uni-tuebingen.de)

We report a case of a fetus with massive sonographic abnormalities diagnosed at 20+1 weeks of gestation. Chromosome analysis of chorionic villus sampling showed no numerical aberrations in short term culture, but several structural abnormalities like a shortened p-arm of chromosome 3, an interstitial deletion in chromosome 14 (bands q12 to q21.3) and additional material of unknown origin on the long arm of chromosome 16 (not resembling 3p) in long term culture.

SNP array analysis confirmed the interstitial deletion in 14q, of 17.5 Mb in size. Additionally, a 3 Mb sized interstitial deletion in 3q13.11 was found, but no imbalance of 3p or 16q. Using a painting probe for chromosome 3, a reciprocal translocation t(3;16) was confirmed.

Banding cytogenetics of the parents showed normal karyotypes in both, and additional FISH tests did not reveal any cryptic aberrations for chromosomes 3, 14 and 16.

At 23+0 weeks of gestation, parents decided to terminate the pregnancy.

As described in the literature, interstitial deletions 3q are not associated with severe abnormalities, whereas a number of patients carrying interstitial deletions 14q smaller than in our case presented severe developmental disorders. Overall, a combination of different structural aberrations in one patient is very rare in occurrence; potential mechanisms of formation including chromothripsis will be discussed.

#### 3.P17 Chromosome 18p11.31p11.23 Triplication

##### Vera Lima^1^, Joel Pinto^1^, Francisco Valente^2^, Cristina Godinho^2^, Sergio Castedo^1^, Alberto Barros^1^, Sofia Dória^1^

###### ^1^Faculty of Medicine, I3s-Instituto De Investigação E Inovação Em Saúde, University of Porto, Genetics, Porto-Portugal; ^2^Centro Hospitalar Vila Nova De Gaia/espinho, Diagnóstico Prenatal E Ecografia- Serviço De Ginecologia/obstetricia, Porto-Portugal

####### **Correspondence:** Vera Lima (veralima2308@gmail.com)

A 39 years old pregnant woman was referred for array Comparative Genomic Hybridization (aCGH) study due to poor obstetrical history. This was the 3rd pregnancy of a non-consanguineous couple, who has a healthy 11 years old girl and had a previous neonatal death at 29 weeks with increased Nuchal Translucency and fetal hydrops. There was no familial history of genetic diseases, malformations or psychomotor delay. The aCGH (8x60K from Agilent) showed the presence of a CNV with a log ratio compatible with the presence of 4 copies on chromosome 18p. The result was described as: arr 18p11.31p11.23(6,942,021-8,055,875)x4. aCGH performed in the parents revealed that the father is carrier of the same CNV, suggesting an inherited triplication in the fetus. As far as we know, this is the first case of a 18p11.31p11.23 triplication. In this segment, LAMA 1 is the most relevant gene but is associated with an autosomal recessive disease (Poretti-Boltshauser Syndrome).

In this case, even if we admit that the triplication may compromise the structure of the gene and hence the integrity of the gene on the paternal chromosome 18, the allele maternal should remain normal, making the possibility of Poretti-Boltshauser Syndrome unlikely. Additionally, admitting that the gain of the involved gene(s) could cause malformations or psychomotor delay, it is logical to presume that the effects of a triplication could be clearly higher comparing to a duplication, and therefore it was more likely to find clinical features in a carrier of a triplication.

In conclusion, the existence of a healthy father with the same alteration detected in the fetus constitutes a favorable prognostic sign, although incomplete penetrance of genomic imbalances cannot be excluded. Thus, a slightly increased risk of malformations/mental retardation comparison with general population should be considered.

“Written, informed consent for publication was obtained from the patient [or parent/guardian for patients under 16]”

#### 3.P18 Prenatal diagnosis of monozygotic twins discordant for non mosaic tetrasomy 9p

##### Ana Vicic^1^, Ivanka Bekavac Vlatkovic^1^, Kristina Crkvenac Gornik^2^, Feodora Stipoljev^1^

###### ^1^Clinical Hospital Sveti Duh, Department of Obstetrics and Gynecology, Zagreb-Croatia; ^2^University Hospital Centre Zagreb, Department of Laboratory Diagnostics, Division of Cytogenetics, Zagreb-Croatia

####### **Correspondence:** Ana Vicic (vicic.ana@gmail.com)

It has been established that monozygosity does not necessarily denotes the same genetic or chromosomal constitution, since discrepancies were recorded for several genetic, epigenetic and chromosomal disorders. However, discrepancies for structural chromosomal abnormalities are extremely rare. We present prenatally diagnosed case of dichorionic-diamniotic (DC/DA) monozygotic twins discordant for non-mosaic tetrasomy 9p.

A 42-year-old woman, G2P1, with spontaneous twin pregnancy was referred for ultrasound examination, which revealed DC/DA twins with bilateral hydronephrosis present in one fetus. Amniocentesis from both gestational sacs was performed at 18 weeks gestation. Cytogenetic analysis showed non-mosaic male karyotype with a supernumerary marker chromosome in affected twin. FISH analysis on cultured amniotic fluid cells revealed an existence of extra isochromosome 9p, resulting in tetrasomy 9p. A 46,XY karyotype was found in other twin. Parental karyotypes were normal. Microsatellite analysis of DNA extracted from both amniotic fluid samples was consistent with monozygosity. Fetal echocardiography showed complex heart defect in fetus with tetrasomy 9p, while sonographic examination besides the hydronephrosis revealed ambiguous genitalia, hydrocephalus, facial dysmorphy and anomalies of extremities. Serial ultrasound examinations were unremarkable for normal twin. After genetic counseling parents opted for selective feticide. Chromosomally normal co-twin was delivered at 36 weeks gestation, weighting 2,240 g, with no observed malformations, except for hypospadias. Placental examination confirmed dichorionic-diamniotic placentation, and microsatellite analysis of chorionic villi and skin sample of affected fetus confirmed monozygosity. Array CGH analysis of DNA isolated from skin sample confirmed non-mosaic tetrasomy 9p in abnormal fetus.

Our case emphasizes the significance of microsatellite analysis for the determination of zygosity in dichorionic like-sex twins, in order of planning obstetric management of twin pregnancies, as well as counseling parents regarding possible outcome of pregnancy. Furthermore, cytogenetic analysis is mandatory in both monozygotic diamniotic twins discordant for structural anomalies.

“Written, informed consent for publication was obtained from the patient [or parent/guardian for patients under 16]”

#### 3.P19 Recombinant chromosome derived from two independent translocations of the same maternal homologues Incidental finding in a fetus

##### Claudia Alves^1^, Mafalda Lopes^1^, Isabel Cerveira^2^, Carolina Ferreira^2^, Fernanda Baltar^1^, Rita Monteiro^1^, Cecília Correia^1^, Margarida Reis Lima^3^

###### ^1^Synlabhealth, Cytogenetics, Porto-Portugal; ^2^CHTV-Hospital S. Teotónio, Obstetrics and Gynaecology Dept, Fetal Medicine Unit, Viseu-Portugal; ^3^Synlabhealth, Medical Genetics, Porto-Portugal

####### **Correspondence:** Claudia Alves (claudia.alves@synlab.pt)

Context:

Complex chromosome rearrangements (CCR) account for a very small number of cases described in the literature. It is very rare that both homologues of the same chromosome pair are involved each in a different rearrangement. We report a case where a reconstructed derivative chromosome was observed in a fetus of a female carrier of two different translocations involving both chromosomes 4.

Methods and Results:

A 22 years-old primigesta was referred for an invasive procedure due to cystic hygroma, fetal hydrops, omphalocele, malformation of the left forearm/hand and of the right foot. CVS was performed on the 13th week of gestation.

QF-PCR revealed trisomy 18 in a male fetus. Karyotype analysis showed trisomy 18 resulting from a CCR involving chromosomes 4, 10 and 18.

Parental cytogenetic studies were carried out. Two reciprocal translocations involving both homologues of chromosome 4 were observed in the mother: one between one chromosome 4 and a 10; and the other between the chromosome 4 homologue and an 18. Her karyotype was described as 46,XX,t(4;10)(q21.1;p13),t(4;18)(q33;q21.1).

This unveiled the presence of a recombinant chromosome 10 in the fetus, resulting from (at least one) recombination event between the derivative 10 of t(4;10) and the derivative 4 of t(4;18).

Fetal autopsy confirmed the ultrasound findings and revealed also microcephaly, alterations of the cerebral cortication and cardiopathy.

Discussion:

Depending on the type and size of the chromosomal segments involved in a CCR, it is acknowledged that the chance of viable conceptuses is low when such events are observed. Trisomy 18 was the net imbalance of this particular case. If only QF-PCR was requested, the CCR could have been missed before a new pregnancy, and genetic counselling not offered. Such a set of translocations involving both homologues of a particular chromosome pair and the observation of a newly formed chromosome as a result of a recombination event between two derivatives of different translocations, makes this a unique case.

#### 3.P20 Importance of systematic FISH to complete the genetic counseling in de novo microdeletionnel duplicationnel recurrent syndrome. A case of 17q12 prenatal diagnosis

##### Dorothée Reboul^1^, Cécile Arnoult^1^, Frédéric Grosjean^2^, Yuliya Pétrov^1^, Thiérry Lavabre-Bertrand^1^, Jean Chiesa^1^

###### ^1^Chu, Cytogénétique, Nîmes-France, Metropolitan; ^2^Chu, Gynécologie, Nîmes-France, Metropolitan

####### **Correspondence:** Dorothée Reboul (dorothee.reboul@chu-nimes.fr)

In ACLF guidelines, when a pathogenic rearrangement is identified by ACPA, a parental origin must be excluded. FISH method when possible is strongly adapted for genetic investigations: FISH can detect balanced chromosomal abnormalities.

We describe a case of a prenatal 17q12 micro-deletion diagnosed by ACPA in a fetus with a small weight at 24 gestational weeks .

The deletions/duplications of 17q12 region are linked with 2 recurrent chromosomic abnormalities mirrored. The 17q12 deletion is widely described in the literature and registered in the frame of a micro-deletion syndrome characterized by a late-onset diabete, nephropathy, neurodevelopmental abnormalities and rarely disturbances of autistic spectrum. The duplication of the 17q12 region has a weaker penetrance and a great intrafamilial phenotypic variability.

In our case, the microdeletion is considered de novo with normal parental results in ACPA and quantitative PCR. So the couple decided a TOP.

The subsequent analysis of in situ hybridization from locus 17q12 confirm the fetal deletion but also show in the mother a single copy on a single 17 metaphase chromosome and 3 copies on interphase nucleus, so revealing a 17q12 deletion on a 17 chromosome with duplication on the homologous 17 chromosome.

The extended family study at the parents of the mother shows that her father is carrier of a 17q12 microduplication and her mother a 17q12 microdeletion. The haplotypes analysis in 17q12 confirm the transmission of theses 2 rearrangements to their daughter.

They are few cases of balanced microdeletionnel / duplicationnel recurrent syndrome (1) This dual transmission of the 2 imbalanced alleles is the first to our knowledge.

This first case described recalls the ACPA limits and the interest of FISH in the balanced microdeletionnel / duplicationnel recurrent syndrome to exclude a equilibrated parental rearrangement.

Effectively, the genetic counseling is totally different with a recurring risk of 100 % for the next pregnancies in the patient carrier of the dual mirror abnormality.

1-Delicado and all BMC Medical Genetics 2014; 15:116

### 4. Animal and Plant Cytogenomics

#### 4.P1 Cytogenomics of Deschampsia species and some grasses from related genera (Aveneae Poeae Poaceae) growing under environmental stress conditions

##### Olga Muravenko^1^, Svyatoslav Zoshchuk^1^, Olga Yurkevich^1^, Tatiana Samatadze^1^, Igor Loskutov^2^, Elizaveta Punina^3^, Alexander Rodionov^3^, Alexandra Amosova^1^

###### ^1^Engelhardt Institute of Molecular Biology, Russian Academy of Sciences, Moscow-Russia; ^2^Frc N.i. Vavilov All-Russian Institute of Plant Genetic Resources (vir), Ministry of Science And Higher Education, St. Petersburg-Russia; ^3^V.l. Komarov Botanical Institute, Russian Academy of Sciences, St. Petersburg-Russia

####### **Correspondence:** Olga Muravenko (olgmur1@yandex.ru)

Molecular cytogenetic analysis of eight pasture species grown under environmental stress conditions and represented related genera Deschampsia, Alopecurus, Avena, Beckmannia and Holcus from the Aveneae/Poeae tribe complex was performed for the first time by DAPI-banding, FISH with 45S rDNA, 5S rDNA and other repeated DNAs and also sequential rapid GISH. Based on distribution patterns of the examined chromosomal markers, the ploidy status and karyotypic structure of these species were specified; peculiarities and common features of their genomes were described. Different chromosomal rearrangements were detected in Beckmannia syzigachne (Steud.) Fernald, Deschampsia cespitosa (L) P. Beauv., Deschampsia sukatschewii (Popl.) Roshev. and Deschampsia flexuosa (L.) Trin. (= Avenella flexuosa (L.) Drejer). B chromosomes with distinct DAPI-bands were observed in karyotypes of D. cespitosa and Holcus lanatus L. Some karyotypic similarities between diploid Alopecurus aequalis Sobol. and allotetraploid Alopecurus arundinaceus Poir. revealed here could be related to the common progenitor for these species. In karyotype of allotetraploid Alopecurus arundinaceus, one chromosome pair had a pattern of molecular markers similar to chromosome 1 of Avena longiglumis Dur. (AlAl), and this specific chromosome pair was also described earlier in diploid and polyploid Avena species with different types of the A genome. The chromosomes of diploid B. syzigachne differed greatly from the studied Alopecurus accessions. The performed comparative cytogenomic analysis confirms the recent molecular phylogenetic data considered that the genera Alopecurus, Beckmannia, Deschampsia and Holcus should be placed into different subtribes within the Poeae tribe. Our findings are important for further taxonomic, phylogenetic, genetic and biotechnological studies and also selection of new productive varieties of cool-season forage grasses. This work was supported by the Russian Foundation of Basic Research, projects no. 17-00-00340 KOMFI (17-00-00336, 17-00-00337, 17-00-00338).

#### 4.P2 Sex chromatin identifies atypical W chromosomes in loopers (Geometridae)

##### Martina Flegrová^1^, Magda Zrzavá^2^, František Marec^1^

###### ^1^Institute of Entomology, Biology Centre, Cas, Molecular Biology and Genetics, České Budějovice-Czech Republic; ^2^Faculty of Science, University of South Bohemia, Molecular Biology and Genetics, České Budějovice-Czech Republic

####### **Correspondence:** Martina Flegrová (martina.flegrova@entu.cas.cz)

Moths and butterflies (Lepidoptera) are the biggest group with female heterogamety (i.e. WZ/ZZ sex chromosome system). In the advanced group Ditrysia, the presence of a female-specific W chromosome appears to be a typical feature, although several exceptions occur. The presence or absence of the W chromosome in Lepidoptera is typically investigated via the presence/absence of the sex-chromatin body, a dark round object formed by the W chromosome in polyploid somatic nuclei due to its partial or complete heterochromatinization. However, low level of W chromosome degeneration and/or its high transcriptional activity (e.g. caused by fusion with an autosome) might disrupt the sex-chromatin formation. Such sex-chromatin malformation or absence is noticeably frequent in the group Geometridae (Ditrysia). This might indicate that W-autosome fusions or other chromosomal rearrangements could be common in Geometridae. To uncover possible link between sex chromatin appearance and the W chromosome composition, we have screened multiple species for the sex chromatin presence. In selected species, we have investigated their sex chromosomes via comparative genomic hybridization (CGH). The results have confirmed our premise since the sex chromatin occurrence and shape corresponds with the W chromosome appearance. To conclude, this study revealed that sex chromatin is not a reliable marker of W chromosome presence, but can be used as an indicator of chromosome rearrangements or low level of WZ differentiation.

#### 4.P3 Independent evolution in one homolog of the 20 21 syntenic association in Cercopithecini monkeys (possibly) involving evolutionary neocentromere seeding

##### Doron Tolomeo^1^, Giorgia Chiatante^2^, Roscoe R Stanyon^1^, Mariano Rocchi^2^, Nicoletta Archidiacono^2^, Luca Sineo^3^, Oronzo Capozzi^2^

###### ^1^University of Florence, Department of Biology, Florence-Italy; ^2^University of Bari, Department of Biology, Bari-Italy; ^3^University of Palermo, Department of Biological, Chemical, and Pharmaceutical Sciences and Technologies, Palermo-Italy

####### **Correspondence:** Doron Tolomeo (doron.tolomeo@uniba.it)

Although the Cercopithecini is the most karyotypically diverse tribe of Old World monkeys (2n= 48 to 72), all species share a syntenic association of chromosomes homologous to human 20 and 21. Various forms, particularly in centromere position, are known in both between and within species. We used molecular cytogenetic methods, including detailed BAC-FISH assays, to analyze this chromosome in four species: Chlorocebus aethiops (CAE), Erythrocebus patas (EPA), Cercopithecus mitis albogularis (CAL) and Cercopithecus petaurista (CPE). We defined the ancestral form (form A) and traced evolutionary pathways to four variant forms. CAE was homozygous for form B, EPA was homozygous for form C and, notably, both Cercopithecus species were heterozygous for the ancestral form and highly derived forms: CAL (A, D) and CPE (A, E). A series of common inversions show that forms D and E emerged before CAL/CPE divergence (five million years ago). Because all four derivative forms share an initial inversion, ancestral and derived forms of CAL/CPE may have coexisted for 8 million years (time of CPE-CAL/CAE-EPA divergence). The heterozygosity of ancestral and derived forms for such a long evolutionary time could be explained by a selective advantage and, even if theoretically heterozygous inversions produce unbalanced gametes, crossing over may be suppressed. Finally, FISH shows that derivative forms D and E harbor much smaller amount of centromeric alpha satellite DNA than either the ancestral form A or other chromosomes. This may be due to multiple rearrangements that occurred in the centromeric regions of these chromosomes or the presence of an evolutionary new centromere in the last common ancestor of CAL and CPE. Future research, including sequencing, may help determine between these hypotheses.

#### 4.P4 New insights into chromomere organization provided by lampbrush chromosome microdissection and high throughput sequencing

##### Anna Zlotina^1^, Antonina Maslova^1^, Olga Pavlova^2^, Nadezda Kosyakova^3^, Thomas Liehr^3^, Alla Krasikova^1^

###### ^1^Saint Petersburg State University, Cytology and Histology Department, Saint-Petersburg-Russia; ^2^Saint Petersburg State University, Research Resource Center, Saint-Petersburg-Russia; ^3^Friedrich Schiller University, Jena University Hospital, Institute of Human Genetics, Jena-Germany

####### **Correspondence:** Anna Zlotina (anna-zlotina@yandex.ru)

Giant lampbrush chromosomes (LBCs) typical for growing oocytes of various animal species are characterized by specific chromomere-loop appearance and massive transcription. Chromomere-loop complexes represent universal units of chromatin packaging at LBC stage. While quite a good progress has been done in investigation of LBCs structure and function, chromomere organization still remains poorly understood.

Our previous studies showed that lampbrush chromosome microdissection is a powerful tool for investigation of tiny chromosomal regions with high precision. To extend our knowledge on chromomere organization and genomic context, we applied microdissection on chicken LBCs. In particular, 30 individual chromomeres were dissected one after another along the macrochromosome 4. FISH on LBCs allowed to map the microdissected regions precisely and to evaluate their transcriptional activity in growing oocytes. The data on genomic context of individual chromomeres were obtained by high-throughput sequencing. Alignment of adjacent chromomeres to chicken genome assembly (build Galgal5) provided information on chromomeresʼ size and genomic boarders indicating that prominent marker chromomeres are about 4-5 Mb in size, while common chromomeres - 1.5-3.5 Mb. Analysis of genomic features showed that the majority of chromomeres combine gene-dense and gene-poor regions, while massive loopless DAPI-positive chromomeres lack genes and are remarkably enriched with different repetitive elements. Finally, LBC chromomeres were compared with chromatin domains (topologically associated domains (TADs) and A/B-compartments) earlier identified by Hi-C technology in interphase nucleus of chicken embryonic fibroblasts. Generally, the results obtained suggest that chromomeres of lampbrush chromosomes do not correspond unambiguously to any type of well-established spatial domains of interphase nucleus of somatic cells.

The research was supported by Interuniversity Partnership Programme, grant of the President of the Russia (МК-1630.2017.4) and RSF (19-74-20075) and performed using experimental equipment of the Research Resource Center MCT of SPbU.

#### 4.P5 Cytogenomics of a sex specific marker on the W chromosome of the invasive mosquitofish Gambusia affinis

##### Stefan Müller^1^, Sofia Adolfsson^2^, Alistair M. Senior^3^, Verena Kottler^4^, Maria Pichler^5^, Manfred Schartl^4^, Shinichi Nakagawa^6^, Dunja K. Lamatsch^5^

###### ^1^Ludwig-Maximilians University, Institute of Human Genetics, Munich University Hospital, Munich-Germany; ^2^Lund University, Division of Molecular Hematology, Lund-Sweden; ^3^University of Sydney, Charles Perkins Centre, Sydney-Australia; ^4^University of Würzburg, Physiological Chemistry, Biocenter, Würzburg-Germany; ^5^University of Innsbruck, Research Department for Limnology, Innsbruck-Austria; ^6^University of New South Wales, School of Biological, Earth and Environmental Sciences, Sydney-Australia

####### **Correspondence:** Stefan Müller (stefan.mueller@med.uni-muenchen.de)

The Western mosquitofish, Gambusia affinis is an interesting model for sex chromosome organization and evolution, because it shows female sex chromosome heterogamety and a ZW/ZZ sex chromosome system. Using genomic and transcriptomic data, we previously identified a G. affinis female sex specific marker highly homologous to the aminomethyl transferase (amt) gene of the closely related platyfish (Xiphophorus maculatus).

In the study presented here, we dissected the genomic region using exonic PCR probes from the G. affinis amt gene, which we localized on the long arm of the W chromosome (Wq) by fluorescent in-situ hybridization. We then obtained a deeper insight into the large-scale genomic structure of the G. affinis W chromosome in comparison to its sister species G. holbrooki with male heterogamety and an XY sex chromosome system. To this end, we applied intra- and interspecific comparative genomic hybridization (CGH) and comparative expressed sequence hybridization (CESH), as well as FISH with rDNA and oligonucleotide repeat probes, and immuno-fluorescence.

Our CGH analyses showed that the long arm of the G. affinis W chromosome is enriched for repetitive sequences. Despite this, by conventional C-banding and by anti-5-methylcytosine immuno-fluorescence staining we could demonstrate that the G. affinis Wq is neither heterochromatic nor hypermethylated and therefore not epigenetically silenced. In contrast, according to our CESH data the entire Wq appears to be highly transcribed. In addition, the terminal region of Wq comprises one of the three major active NORs, which we detected by silver staining and rDNA FISH.

Taken together, these findings led us to speculate that certain expressed noncoding elements from the amt genomic region with architectural localization along Wq may play a role in sex specific gene dosis compensation in this ZZ/ZW system.

#### 4.P6 An overview of the most frequent chromosomal anomalies in domestic animals and their consequences

##### Dana Liana Pusta^1^, Anamaria Ioana Paştiu^1^, Alexandra Tăbăran^1^, Rodica Sobolu^2^, Oana Reget^3^, Camelia Răducu^4^

###### ^1^USAMV Cluj-Napoca, Veterinary Medicine, Cluj-Napoca-Romania; ^2^USAMV Cluj-Napoca, Horticulture- Statistics and Mathematics, Cluj-Napoca-Romania; ^3^USAMV Cluj-Napoc, Veterinary Medicine, Cluj-Napoca-Romania; ^4^USAMV Cluj-Napoc, Animal Breading, Cluj-Napoca-Romania

####### **Correspondence:** Dana Liana Pusta (dana.pusta@usamvcluj.ro)

The aim of this paper is to highlight the most frequent chromosomal anomalies identified in animals and their consequences.

Chromosomal abnormalities can occur as a result of alterations in number and structure of chromosomes. The most frequent cytogenetic anomalies cited in animals are the sexual chromosomes abnormalities, autosomal translocations, trisomy and mutations.

Disorders of sex development (DSD) were reported in cats, dogs, cattle and small ruminants. In cats XXY trisomy, XX/XY chimerism (Szczerbal et al., 2015a), nonmosaic X monosomy (Szczerbal et al., 2015b), mosaicism 37,X/38,XY (Balogh et al., 2015), translocation between X and Y chromosomes (Szczerbal et al., 2015a) mosaicism with a cell line carrying a ring Y chromosome - 37,X/38,X,r (Y) were found (Szczerbal et al., 2017).

Trisomy-X was reported in dogs with abnormal estrous cycles and infertility (O'Connor et al., 2011).

Robertsonian translocations (chromosome 1 and 29), chimerism (XX/XY) and sex chromosome aneuploidy, XXX, XXY, were reported in cattle (Slota et al., 2003; Citek et al., 2009). In small ruminates, beside that mentioned in big ruminants, centric fusion of different chromosomes and rarely deletions and inversions were diagnosed, while in swine reciprocal translocations represent the most common disorders with significant economic loss due to reduction in litter size by up to 50% (Yimer et al., 2014).

Autosomal trisomy was identified in a Standardbred Colt (65, XY + 27) (Brito et al., 2008) but also in a captive chimpanzee (49, XX + 22) (Hirata et al., 2017). In both cases the animals presented a variety of symptoms common to that of humans with trisomy 21.

In conclusion, in animals the most frequent chromosomal abnormalities are the disorder of sex development presenting the same symptoms like in humans and they are not so studied, being presented only as case studies in veterinary medicine.

#### 4.P7 Robertsonian translocations are not a single event of centric fusion mechanistic insights driven by the Bovinae rob(1;29) model

##### Ana Escudeiro^1^, Filomena Adega^1^, T.j Robinson^2^, Margarida Gama-Carvalho^3^, Pat Heslop-Harrison^4^, Raquel Chaves^1^

###### ^1^Bioisi-Utad, Cag- Laboratory of Cytogenomics and Animal Genomics, Vila Real-Portugal; ^2^Stellenbosch University, Department of Botany and Zoology, Stellenbosch-South Africa; ^3^Bioisi-Fcul, Gere Rna Systems Biology, Lisboa-Portugal; ^4^University of Leicester, Department of Genetics and Genome Biology, Leicester-United Kingdom

####### **Correspondence:** Raquel Chaves (rchaves@utad.pt)

Considering the richness of Bovinae genomes in different (peri)centromeric satDNAs, we refined the chromosomal organization of these sequences, thus developing a high resolution centromeric map as a tool to study chromosomal rearrangements involving the centromeric region, specifically Robertsonian Translocations (robs). The interest in the study of robs derives not only from their high frequency in mammalian karyotype evolution, but also from their influence in fertility and disease. The formation of a rob chromosome implies the occurrence of double strand breaks at the peri(centromeric) constitutive heterochromatin of two acrocentric chromosomes, being satDNA sequences the core hotspot for these breaks. In this work, using (peri)centromeric satDNA families, the robertsonian translocation rob(1;29) was analysed in the cattle where it presents as a chromosomal abnormality and in wild Bovinae subspecies, where it is an evolutionary fixed rearrangement. Six species from the Tragelaphini tribe present the homologous rob(1;29) as an established autosome. The dual context of this robertsonian translocation represents a unique opportunity to study presently a translocation event that occurred million years ago in the evolution of Bovinae.

The main aim of our study was determining the molecular mechanism(s) underling these frequent translocations. Analysing how satDNAs reorganized in the centromere of the translocated chromosomes, we were able to show that the analysed robs are multistep complex rearrangements involving a precise elimination and reorganization of specific peri(centromeric) satDNA sequences. Our data highlight the active role of satDNA sequences in the translocation mechanism and reinforces the functional meaning of these repetitive sequences in the chromosomes’ stability, suggesting further the existence of a common mechanism for the other robs in mammals, namely the human rob(14;21).

Acknowledgments

This work was supported by Ph.D. grant SFRH/BD/98122/2013 from the Science and Technology Foundation (FCT) from Portugal. This work was funded by the BioISI project UID/MULTI/04046/2019 Research Unit grant from FCT, Portugal.

#### 4.P8 Cytogenetic investigations in Romanian cattle and buffalo females with reproductive disturbances

##### Ioana Nicolae^1^, Adrian Bota^2^, Daniela Vidmichi^1^

###### ^1^Research&development Institute for Bovine, Cytogenetics, Balotesti-Romania; ^2^Research&development Station for Buffalo, Reproduction, Sercaia-Romania

####### **Correspondence:** Ioana Nicolae (ioana_nicolae2002@yahoo.com)

The main objective of the special project developed in the last 5 years was to identify the carriers of chromosomal abnormalities which particularly influence their reproductive performances. The cytogenetic investigation was carried out for 209 females (144 cattle and 65 buffaloes) reared in different farms from all over the country. Our study revealed chromosomal instability for 31 females (22 cattle and 9 buffaloes). One of these females had a calf with posterior limb malformation, characterized by the lack of the left posterior leg. The chromosomal complement of the malformed calf and its mother was severely affected, the number of mono-and bi-chromatid breakages on autosomes and heterosomes, loss of chromosome fragments and gaps being much higher than in all the other described cases. Our investigation revealed also the Turner’s syndrome (2n = 49,X0 ) in the case of a buffalo females with prominent withers and tight pelvis. According to these results, the role of chromosomal abnormalities as causes of reproductive failure is very important and involve both the chromosome number and the chromosome structure which are very often associated with developmental abnormalities and various levels of infertility (repeated inseminations, lack of estrus and loss of pregnancy).

Acknowledgements

This study was supported by the ASAS Foundation Project 167/2014-2018.

#### 4.P9 Gradual chromosome elimination during gametogenesis of interspecies hybrids from Pelophylax esculentus complex

##### Dmitrij Dedukh^1^, Sergey Rymin^1^, Magdalena Chmielewska^2^, Beata Rozenblut-Kościsty^2^, Krzysztof Kolenda^2^, Mikołaj Kazmierczak^2^, Maria Ogielska^2^, Alla Krasikova^1^

###### ^1^Saint-Petersburg State University, Cytology and Histology, Saint-Petersburg-Russia; ^2^University of Wroclaw, Evolutionary Biology and Conservation of Vertebrates, Wroclaw-Poland

####### **Correspondence:** Dmitrij Dedukh (dmitrijdedukh@gmail.com)

Genome integrity is a crucial feature of eukaryotic organisms. However, some organisms are able to selectively eliminate a certain part of the genome. Moreover, the selective elimination of genetic material was also found in some interspecies hybrids harbouring asexual reproductive modes. During such reproductive mode, named hybridogenesis, one parental genome is selectively eliminated from germ cells while the other one doubled to overcome normal meiosis. To uncover how the genome is eliminated during hybrid gametogenesis we selected European water frogs complex (Pelophylax esculentus complex) as a model. Two parental species, P. lessonae (LL), P. ridibundus (RR), hybridize to produce interspecies hybrids, P. esculentus (RL). Not only diploid but also triploid (RRL, LLR) hybrids exist in nature and exploit genome elimination for their reproduction. To detect elimination which is known to occur premeiotically, we analyzed gonads of hybrid tadpoles obtained after backcrosses and crosses of parental species from separate localities. In gonads of all hybrid tadpoles, we found micronuclei in the cytoplasm of germ cells. Immunostaining with anti-centromere CREST serum revealed one signal per each micronucleus indicating that each micronucleus comprises one centromere. FISH with the probe specific to P. ridibundus centromeric sequences revealed that triploid LLR hybrids preferentially eliminate R chromosomes in their micronuclei while diploid hybrid frogs preferentially eliminate L chromosomes. Rarely found misaligned P. lessonae chromosomes in diploid hybrids may indicate the appearance of some of the micronuclei of chromosomal lagging. Immunostaining against heterochromatin marks showed their enrichment in the micronuclei. Thus in gonads of hybrid tadpoles chromosomes subsequently eliminate and enclosed into micronuclei.

The work was funded by RSF No 18-74-00115 and partially performed using facilities of the Research Resource Centers “Environmental Safety Observatory” and “Molecular and Cell Technologies” of St. Petersburg State University.

#### 4.P10 Nucleolus organizers transposition in the genome of Japanese quail

##### Alsu Saifitdinova^1^, Svetlana Galkina^2^, Maria Kulak^2^, Valérie Fillon^3^, Valeria Volodkina^2^, Olga Pavlova^4^, Elena Gaginskaya^2^

###### ^1^Herzen State Pedagogical University of Russia, International Centre of Reproductive Medicine, Department of Human and Animal Anatomy and Physiology, Saint Petersburg-Russia; ^2^Saint Petersburg State University, Biological Faculty, Saint Petersburg-Russia; ^3^National Institute of Agricultural Research, Genphyse Laboratory, Toulouse-France; ^4^International Centre O of f Reproductive Medicine, Beagle Ltd., Laboratory of Assisted Reproductive Technologies, Saint Petersburg-Russia

####### **Correspondence:** Alsu Saifitdinova (saifitdinova@mail.ru)

The vast majority of bird species have a single pair of the nucleolus organizer (NOR) chromosomes in the karyotype. Japanese quail is an exception and has three pairs of active NOR chromosomes. Repetitive nature of the NOR do not allow to analyze the particular organization of each NOR by high throughput sequencing. Our study was aimed at the deciphering of the individual sequences that make up the NORs in Japanese quail genome in order to complete data about their organization. Using primers to the conserved region of the 18S ribosomal RNA gene, we amplified rDNA the range of fragments from the quail genome karyotype. The rDNA fragments were found to localize by FISH on short heterochromatic arms of all acrocentric chromosomes in the complement. A set of the fragments was cloned, sequenced and analyzed bioinformatically. In addition to 18S rDNA, we have found chimeric sequences containing fragments of transposable elements, fragments of MHC genes and some others. Аs it was shown earlier, active transcription makes NOR a target for transposons and causes mutual amplification of the chimeric sequences of transposons and ribosomal genes. In mammals, this may cause cell transformation and malignancy. In the genome of Japanese quail, a similar event apparently led to the dispersal and amplification of NORs, three of which retained their functionality.

Acknowledgements

Financial and technical support: RFBR #18-04-01276-а, Research Resource Centre Chromas and Research Resource Centre Molecular and Cell Technologies of Saint Petersburg State University.

#### 4.P11 Description of new tandem repeats in the genome of Japanese quail

##### Maria Kulak^1^, Aleksei Komissarov^1^, Alexander Dyomin^2^, Valerie Fillon^3^, Elena Gaginskaya^4^, Alsu Saifitdinova^5^, Svetlana Galkina^1^

###### ^1^Saint Petersburg State University, Biological Faculty, Saint Petersburg-Russia; ^2^Saratov State Medical University, Research Institute of Fundamental And Clinical Uronephrology, Saratov-Russia; ^3^Inra Toulouse-Occitanie, Genphyse, Castanet Tolosan-France; ^4^Research Institute of Fundamental and Clinical Uronephrology, Biological Faculty, Saint Petersburg-Russia; ^5^Herzen State Pedagogical University of Russia, Department of Human and Animal Anatomy and Physiology, Saint Petersburg-Russia

####### **Correspondence:** Maria Kulak (ontica@mail.ru)

Japanese quail (Coturnix japonica) is the highest-producing poultry species and a model species in developmental, behavioral, vertebrate physiology and disease studies. The size of its genome is ~1.41 Gb being 1/7 time bigger than the chicken’s one. According to the International Quail Genome Consortium estimation, the amount of repeat sequences in C. japonica genome is about 25% that is much higher than in other avian genomes. Repetitive elements compose an essential part of constitutive heterochromatin of the centromere regions as well as the p-arms of acrocentric macrochromosomes CJA3-5 and most of the microchromosomes. Therefore, accumulation of repetitive sequences and transposable elements probably led to quail genome increased size. Besides telomeric (TTAGGG)n repeat, only PO41 and BglII 41 bp tandem repeats were described in the Japanese quail genome to date.

In this work, we performed a search of tandem repeats in unassembled short raw read datasets of the Japanese quail genome deep sequencing. We found 23 tandem repeats representing at least 4.8% of the Japanese quail genome. FISH with specific oligonucleotide probes revealed some of them contribute to heterochromatin of short arms of CJA3, CJA4 and microchromosomes. Repeat CjapSAT was found in pericentromeric heterochromatin on CJA 1-6 and three pairs of microchromosomes, as well as in p- and q-arms of CJAW. The description of satellite repeats is a necessary step to understand the significance of satellite DNA for the genome functioning and genome changes during the evolutionary process.

Acknowledgements

SPbSU grant #1.40.1625.2017, Research Resource Centres “Chromas”, “Molecular and Cell Technologies” and Theodosius Dobzhansky Center for Genome

Bioinformatics of Saint Petersburg State University.

#### 4.P12 Co localization of highly amplified ribosomal and telomeric sequences in Bacillus (Insecta Phasmatodea)

##### Susanna Salvadori^1^, Elisabetta Coluccia^1^, Federica Deidda^1^, Valerio Scali^2^

###### ^1^Università Di Cagliari, Dipartimento Di Scienze Della Vita E Dell'ambiente, Cagliari-Italy; ^2^Università Di Bologna, Dipartimento Di Bigea, Bologna-Italy

####### **Correspondence:** Susanna Salvadori (salvador@unica.it)

Stick insects are well known for their reproductive plasticity: in fact beside bisexual reproduction, thelytokous parthenogenesis, hybridogenesis and androgenesis are at work among them. Stick insects karyotypes very often show a variable number and size of cytological satellites, which are the site of highly-amplified, interspersed NOR and telomeric sequences (Scali et al, 2016). Here we investigate by dual FISH of 28S ribosomal and (TTAGG)n pentameric telomeric probes the NOR and telomeric features of Bacillus, which encompasses three parental (B. grandii, B. atticus, B. rossius) and two derived hybrid parthenogens (B. whitei, 2n=rossius/grandii; B. lynceorum, 3n=rossius/grandii/atticus). Bacillus specimens were field collected on Italian mainland, Sardinia and Sicily. In parthenogens a few fissions and translocations causing both numerical increases and re-patterned chromosomes for deletions and translocations were observed. After dual FISH, cytological satellites were always marked by both probes, suggesting that the amplification/interspersion of NOR and telomeric sequences, recently demonstrated in other unrelated species (Scali et al, 2016; Liehr et al, 2017) could be a shared and widespread trait in phasmids. Chromosome locations and number of NORs were found to differ among species: in B. grandii and B. atticus only a maximum of two locations were noticed, while in B. rossius and the two hybrids up to 10 different locations were encountered with striking differences even within the same species. The high number of re-patterned chromosomes and the satellite variability appear to be linked to the B. rossius genome, which has been demonstrated to embody a high copy-number of R2 retrotransposons presenting an insertion target-site in the 28S rRNA gene (Martoni et al, 2015).

### 5. Accreditation, Quality Control, Education

#### 5.P1 External Quality Assessment (EQA) for Genomics and Clinical Genetics ensuring the quality of the entire genetics service

##### Rosalind Hastings^1^, Katrina Rack^1^, Jenni Fairley^1^, Fiona Morgan^2^, Mark Sales^1^, Sandi Deans^1^

###### ^1^GENQA, Oxford-United Kingdom; ^2^GENQA, Edinburgh-United Kingdom

####### **Correspondence:** Rosalind Hastings (ros.hastings@ouh.nhs.uk)

External Quality Assessment (EQA) is educational and aims to improve and validate the overall quality of the entire genomic and genetic service to the user, from sample receipt to genetic counselling. EQA involves the analysis and interpretation of the same clinical scenario, providing an ideal means to study reporting practices. A key component of a genomic laboratory report is ensuring that the referring clinician is given a clear interpretation of the results, including potential implications for the patient and the family. EQA has highlighted the variable approaches to reporting the genetic results emphasising the need for (a) close interaction between the laboratory and clinician; (b) continued EQA participation and (c) guidelines to promote consistency and ensure high quality testing for the benefit of patients.

Genomics Quality Assessment (GenQA) is a member of the UK NEQAS Consortium and operates from two sites, Oxford and Edinburgh. GenQA offers over 80 EQAs covering Somatic and Acquired genetics within the areas of Cytogenomics, Haematology, Newborn Screening, Non-invasive prenatal testing (NIPT), Molecular Genetics, Molecular Pathology, Prenatal Diagnosis and Preimplantation Genetics. In addition to the above EQAs, GenQA also offers educational EQAs, a competency and training tool (G-TACT) and a tissue assessment EQA (Tissue-i). Specific EQAs are also provided to cover the technical aspects of next generation sequencing and DNA extraction.

Quality assessment has long been associated with successful and efficient laboratory testing, but not clinical services. To address this obvious gap, EQAs for clinical genetics and genetic counselling services are now also provided through GenQA.

EQAs have highlighted the need to implement methods that independently assess laboratory and clinical accuracy of genetic services. This talk will give an overview of GenQA’s EQA repertoire with a focus on the EQAs that involve the classification of the pathogenicity of variants, NIPT as well as the competency EQAs such as the implementation of G-TACT for laboratories and Clinical Genetics EQAs for Clinical Geneticists. This talk will also show how EQA can help improve the diagnostic service offered by genetic services to patients.

#### 5.P2 Quality control CHECK YOUR CULTURES! Karyotyping identifies genetic instability in iPSC

##### Ulrike A. Mau-Holzmann^1^, Maria Joana Osorio^2^, Niklas Schwarz^3^, Benjamin Schmid^4^

###### ^1^University of Tuebingen, Institute of Medical Genetics And Applied Genomics, Tuebingen-Germany; ^2^University of Copenhagen, Center For Basic And Translational Neuroscience, Copenhagen-Denmark; ^3^University of Tuebingen, Hertie Institute, Tuebingen-Germany; ^4^Technological University of Denmark, Bioneer A/s, Hoersholm-Denmark

####### **Correspondence:** Ulrike A. Mau-Holzmann (ulrike.mau@med.uni-tuebingen.de)

Many useful guides and guidelines exist for those working with cell cultures to be aware of contamination with microorganisms, phenotypic and genetic instability or even cell-line misidentification. A lot of these problems are avoidable with the nescessary foresight. Though, simple rules are frequently ignored. Consequently, with depressing regularity retraction or modification of these data is seen.

Among the problems that continue to affect cell culture, genetic instability is an important mosaic stone. Depending on the cell type (senescent or immortal cell lines, human or not human, iPSC, cancer cell line) the chromosomal content can be euploid, aneuploid (abnormal chromosome content) and heteroploid (variable chromosome content within the population). Aneuploidy, heteroploidy or even special structural aberrations can be typical for specific cell lines. The latter can be very useful for authentication.

For reprogramming (iPSC), transdifferentiation or the more and more popular technology of gene-editing, cells normally have to undergo single cell subcloning steps, where they are often treated with inhibitors that prevent the cells from undergoing apoptosis. In fact, around 10-20% of cells show chromosomal aberrations after reprogramming, transdifferentiation or gene-editing technologies. Therefore, they need to be periodically karyotyped.

The primary purpose of this poster is to increase awareness for genetic instability to those new in the field and those engaged in teaching and instruction. Results of conventional karyotyping (which has the advantage to detect balanced structural aberrations compared to array technologies) of different cell types and cell lines (mainly iPSC) will be presented.

#### 5.P3 Interactive online karyotyping to teach cytogenetics in undergraduate medical education

##### Emran Mahmoud Tawalbeh, Harald Rieder

###### University Clinics Duesseldorf, Institute of Human Genetics, Duesseldorf-Germany

####### **Correspondence:** Harald Rieder (harald.rieder@uni-duesseldorf.de)

Teaching cytogenetics in undergraduate medical education is a demanding task. In most medical faculties cytogenetics is being taught by paper-based karyotyping using chromosomes which have to be cut from printed metaphases. Students consider this approach old-fashioned and outdated and the handling of the chromosomes is cumbersome and error-prone. Therefore, we created an internet browser based online karyotyping module using an e-learning course development software (articulate storyline®)). The chromosomes of a karyotype are presented as draggable objects and can be moved by mouse or by gestures when using a touch screen. To complete a karyotype the chromosomes have to be moved to the correct position of a karyogram form. The students submit the completed karyogram to the system. The karyogram is rejected if any chromosome has not been placed at its correct position, and the students have to correct the mistake before they resubmit the karyogram. If the karyogram has been accepted, the students have to write the correct karyotype. The karyotype is rejected if it is not written according to ISCN. A course containing seven different karyotypes including numerical as well as structural chromosomal changes was developed and was integrated in the learning management system ILIAS as a sharable content object reference (SCORM) module. In a practical lesson, which was attended by 150 students, each student accessed the course using his own mobile electronic device via WIFI. The students were assisted by cytogeneticists so that each student was able to complete at least one online karyotype. Thus, the online karyotyping course proved to be a robust and platform independent tool for teaching cytogenetics. The system may also be used to address different levels of cytogenetic skills and/or specific learning objectives such as complex karyotypes in acute leukemias, for instance, in graduate medical education and cytogenetic specialization. Supported by the eLearning Foederfonds of the Heinrich-Heine-University, Duesseldorf

### 6. Genomics

#### 6.P1 Inversion variants in human and primate genomes

##### Claudia R Catacchio^1^, Flavia AM Maggiolini^1^, Pietro D'addabbo^1^, Miriana Bitonto^1^, Oronzo Capozzi^1^, Martina Lepore Signorile^1^, Mattia Miroballo^1^, Nicoletta Archidiacono^1^, Evan E. Eichler^2^, Mario Ventura^1^, Francesca Antonacci^1^

###### ^1^University of Bari, Dept. of Biology, Bari-Italy; ^2^University of Washington, Dept. of Genome Sciences, Seattle-United States

####### **Correspondence:** Claudia R Catacchio (claudiarita.catacchio@uniba.it)

For many years, inversions have been proposed to be a direct driving force in speciation since they suppress recombination when heterozygous. Inversions are the most common large-scale differences among humans and great apes. Nevertheless, they represent large events easily distinguishable by classical cytogenetics, whose resolution, however, is limited. Here, we performed a genome-wide comparison between human, great ape, and macaque genomes using the net alignments for the most recent releases of genome assemblies. We identified a total of 156 putative inversions, between 103 kb and 91 Mb, cor- responding to 136 human loci. Combining literature, sequence, and experimental analyses, we analyzed 109 of these loci and found 67 regions inverted in one or multiple primates, including 28 newly identified inversions. These events overlap with 81 human genes at their breakpoints, and seven correspond to sites of recurrent rearrangements associated with human dis- ease. This work doubles the number of validated primate inversions larger than 100 kb, beyond what was previously doc- umented. We identified 74 sites of errors, where the sequence has been assembled in the wrong orientation, in the reference genomes analyzed. Our data serve two purposes: First, we generated a map of evolutionary inversions in these genomes representing a resource for interrogating differences among these species at a functional level; second, we provide a list of misassembled regions in these primate genomes, involving over 300 Mb of DNA and 1978 human genes. Accurately annotating these regions in the genome references has immediate applications for evolutionary and biomedical studies on primates.

#### 6.P2 Genomic inversions and GOLGA core duplicons underlie disease instability at the 15q25 locus

##### Flavia A.M. Maggiolini^1^, Stuart Cantsilieris^2^, Pietro D’addabbo^1^, Michele Manganelli^1^, Bradley P. Coe^2^, Beth L. Dumont^3^, Ashley D. Sanders^4^, Andy Wing Chun Pang^5^, Mitchell R. Vollger^2^, Orazio Palumbo^6^, Pietro Palumbo^6^, Maria Accadia^7^, Massimo Carella^6^, Evan E. Eichler^2^, Francesca Antonacci^1^

###### ^1^University of Bari, Dipartimento Di Biologia, Bari-Italy; ^2^University of Washington School of Medicine, Department of Genome Sciences, Seattle-United States; ^3^The Jackson Laboratory, The Jackson Laboratory, Bar Harbor-United States; ^4^European Molecular Biology Laboratory (embl), Genome Biology Unit, Heidelberg-Germany; ^5^Bionano Genomics, Bionano Genomics, San Diego-United States; ^6^Irccs Casa Sollievo Della Sofferenza, Medical Genetics Unit, San Giovanni Rotondo-Italy; ^7^Hospital cardinale G. Panico”, Medical Genetics Service, Tricase-Italy

####### **Correspondence:** Flavia A.M. Maggiolini (flavia.maggiolini@uniba.it)

Human chromosome 15q25 is involved in several disease-associated structural rearrangements, including microdeletions and chromosomal markers with inverted duplications. Using comparative fluorescence in situ hybridization, strand-sequencing, single-molecule, real-time sequencing and Bionano optical mapping analyses, we investigated the organization of the 15q25 region in human and nonhuman primates. We found that two independent inversions occurred in this region after the fission event that gave rise to phylogenetic chromosomes XIV and XV in humans and great apes. One of these inversions is still polymorphic in the human population today and may confer differential susceptibility to 15q25 microdeletions and inverted duplications. The inversion breakpoints map within segmental duplications containing core duplicons of the GOLGA gene family and correspond to the site of an ancestral centromere, which became inactivated about 25 million years ago. The inactivation of this centromere likely released segmental duplications from recombination repression typical of centromeric regions. This increased the frequency of ectopic recombination creating a hotspot of hominid inversions where dispersed GOLGA core elements now predispose this region to recurrent genomic rearrangements associated with disease.

#### 6.P3 Characterization of structural variation of the human glycophorin locus using multicolour fibre FISH

##### Sandra Louzada^1^, Walid Algady^2^, Paulina Brajer^2^, Fengtang Yang^1^, Edward J. Hollox^2^

###### ^1^Wellcome Sanger Institute, Molecular Cytogenetics Core Facility, Cambridge-United Kingdom; ^2^University of Leicester, Department of Genetics and Genome Biology, Cambridge-United Kingdom

####### **Correspondence:** Sandra Louzada (sg14@sanger.ac.uk)

Structural variants (SVs) represent an important source of variation among individual human genomes. These variants are prone to arise in repetitive regions and may involve multiallelic gene families with internal complex structures, which make its characterization a challenging task. Multicolor fibre-FISH on combed DNA-fibres has been successfully used for the visualization and characterization of structural variants, leading to a better interpretation and understanding of genomic structural variation identified by different genomic technologies. The human glycophorin gene cluster is known to undergo extensive structural variation and gene conversion. This region harbours the tandemly arranged glycophorin genes GYPE, GYPB and GYPA, sharing ∼97% identity. Glycophorin A and glycophorin B are red blood cell surface proteins and act as receptors for the parasite Plasmodium falciparum which is the principal cause of malaria in sub-Saharan Africa. An interesting structural variant within this locus is the DUP4 that carries extra copies of a glycophorin A-glycophorin B fusion gene and has shown to reduce the risk of severe malaria by up to 40%. Besides contributing for the validation of this structural rearrangement, multicolour fibre-FISH also revealed somatic variation in copy number of the glycophorin B-glycophorin A fusion gene. Moreover, after analysing high-throughput sequencing from individuals across the world, 11 new variants were identified and a selection of them was further characterized using fibre-FISH revealing some interesting structures. Our results show the structural diversity within the glycophorin locus vindicating the use of fibre-FISH to resolve and characterize multi-allelic SVs.

#### 6.P4 How to detect mobile retrocopies during routine genetic testing and manage pitfalls

##### Nicolas Chatron^1^, Kevin Cassinari^2^, Olivier Quenez^2^, Stéphanie Baert-Desurmont^3^, Claire Bardel^4^, Marie-Pierre Buisine^5^, Eduardo Calpena^6^, Yline Capri^7^, Jordi Corominas Galbany^8^, Flavie Diguet^9^, Patrick Edery^1^, Bertrand Isidor^10^, Audrey Labalme^9^, Cédric Le Caignec^11^, Jonathan Levy^12^, François Lecoquierre^3^, Pierre Lindenbaum^13^, Olivier Pichon^10^, Pierre-Antoine Rollat-Farnier^14^, Thomas Simonet^15^, Pascale Saugier-Veber^3^, Anne-Claude Tabet^16^, Annick Toutain^17^, Andrew O.m. Wilkie^6^, Gaétan Lesca^1^, Damien Sanlaville^1^, Gaël Nicolas^2^, Caroline Schluth-Bolard^1^

###### ^1^Equipe Gendev, Crnl, Inserm U1028, Cnrs Umr5292, Ucbl1, Service De Génétique, Hospices Civils De Lyon, Bron-France; ^2^Normandy Center for Genomic And Personalized Medicine, Normandie Univ, Unirouen, Inserm U1245 And Rouen University Hospital, Department of Genetics and Cnr-Maj, Rouen-France; ^3^Normandy Center for Genomic And Personalized Medicine, Normandie Univ, Unirouen, Inserm U1245 And Rouen University Hospital, Department Of Genetics, Rouen-France; ^4^Service De Biostatistique Bioinformatique, Hcl, Cellule Bioinformatique De La Plateforme De Séquençage Ngs Du Chu De Lyon, Groupement Hospitalier Est, Bron-France; ^5^Lille University Hospital, Inserm Umr-s 1172, Jpa Research Center, Lille University, And Department Of Biochemistry And Molecular Biology, Lille-France; ^6^University Of Oxford, Clinical Genetics Group, Mrc Weatherall Institute Of Molecular Medicine, Oxford-United Kingdom; ^7^Hôpital Universitaire Robert Debré, Ap-hp, Uf De Génétique Clinique, Département De Génétique, Paris-France; ^8^Radboud University Medical Center, Department Of Human Genetics, Nijmegen-The Netherlands; ^9^Hospices Civils De Lyon, Service De Génétique, Bron-France; ^10^Chu Nantes, Service De Génétique Médicale, Nantes-France; ^11^Université De Nantes, Service De Génétique Médicale, Chu Nantes, Nantes-France; ^12^Hôpital Universitaire Robert Debré, Ap-hp, Uf De Cytogénétique, Département De Génétique, Paris-France; ^13^Cnrs, Umr 6291, Inserm, Umr_s1087, L’institut Du Thorax, Nantes-France; ^14^Cellule Bioinformatique De La Plateforme De Séquençage Ngs Du Chu De Lyon, Groupement Hospitalier Est, Service De Génétique, Hospices Civils De Lyon, Bron-France; ^15^Nerve-muscle Interactions Team, Institut Neuromyogène Cnrs Umr 5310 - Inserm U1217 - Université Claude Bernard Lyon 1, Centre De Biotechnologie Cellulaire, Hospices Civils De Lyon, Bron-France; ^16^Unité Génétique Humaine Et Fonction Cognitive, Département De Neurosciences, Institut Pasteur, Uf De Cytogénétique, Département De Génétique, Hôpital Universitaire Robert Debré, Ap-hp, Paris-France; ^17^Umr 1253, Ibrain, Université De Tours, Inserm, Service De Génétique, Hôpital Bretonneau, Chu, Tours, Tours-France

####### **Correspondence:** Caroline Schluth-Bolard (caroline.schluth-bolard@chu-lyon.fr)

Human retrocopies, i.e. mRNA transcripts benefitting from the LINE-1 machinery for retrotransposition, may have specific consequences for genomic testing. NGS techniques allow the detection of such mobile elements but they may be misinterpreted as genomic duplications or be totally overlooked. Here, we present eight observations of retrocopies detected during diagnostic NGS analyses of targeted gene panels, exome, or genome sequencing. For seven cases, while an exon-only copy number gain was called, read alignment inspection revealed a depth of coverage shift at every exon-intron junction where indels were also systematically called. Moreover, aberrant chimeric read pairs spanned entire introns or were paired with another locus for terminal exons. The 8th retrocopy was present in the reference genome and thus showed a normal NGS profile. It was identified during the cDNA study performed to validate an intronic SNV by the preferential amplification of the retrocopy. We emphasize the existence of retrocopies and strategies to accurately detect them at a glance during genetic testing. We discuss pitfalls for genetic testing and their potential clinical consequences.

#### 6.P5 CNTN6 expression in human IPSC derived neurons from patient with neurodevelopmental disorders and 3p26.3 microduplication and the same microduplication healthy carrier

##### Maria Gridina^1^, Natalia Matveeva^2^, Aleksey Mensorov^2^, Tatiana Nikitina^3^, Anna Kashevarova^3^, Nikolai Skryabin^4^, Liudmila Nazarenko^3^, Maria Lopatkina^3^, Igor Lebedev^3^, Oleg Serov^2^

###### ^1^Institute of Cytology And Genetics Sb Ras, Genomic Mechanisms of Ontogenesis, Novosibirsk-Russia; ^2^Institute of Cytology And Genetics Sb Ras, Laboratory Developmental Genetic, Novosibirsk-Russia; ^3^Research Institute of Medical Genetics,, Laboratory of Cytogenetics., Tomsk-Russia; ^4^Research Institute of Medical Genetics,, Laboratory Developmental Genetic, Tomsk-Russia

####### **Correspondence:** Maria Gridina (gridinam@gmail.com)

Copy number variations (CNVs) of the 3p26.3 region are often the cause of neurodevelopmental disorders, including intellectual disability and developmental delay. Recently, we described a patient with neurodevelopmental and neuropsychiatric disorder; who had a microduplication involving a single CNTN6 gene (Kashevarova et al., 2014). The duplication was inherited from the healthy father. Whole-genome sequencing of the patients DNA allowed to determine the exact boundaries of the chromosomal rearrangements, and we did not reveal any structural variations either within the CNTN6 gene or the surrounding region.

We obtained the lines of induced pluripotent stem cells (iPSC) from the patient cells and the healthy carrier the same duplication. The iPSCs were differentiated in vitro into cortical neurons. The level of CNTN6 gene expression was significantly lower than in the neurons of two healthy donors (without the duplication). Allele-specific analysis of its expression revealed that expression of the duplicated allele of the partenal origin was significantly weaker than a normal one.

On the contrary, the level of CNTN6 expression was comparable in the neurons of the healthy carrier of the same duplication inhereted from his healthy mother and in the neurons of the healthy donors. Moreover, allele-specific analysis showed that expression of the duplicated allele, in this case of the maternal origin, was almost in two times higher than a normal allele in the neurons of duplication healthy carrier.

The significant reduction of the CNTN6 expression in neurons obtained from patient iPSCs can explain the similarity of the symptoms observed for the patients with deletions and duplications of the CNTN6 gene.

This study was supported by Russian Science Foundation, grant 14-15-00772-P and by budget project # 0324-2018-0041.

#### 6.P6 STAG1 haploinsufficiency an emerging phenotype

##### Ana Sousa, Sónia Custódio, André Travessa, Ana Berta Sousa

###### CHULN-HSM, Epe, Medical Genetics Department, Lisbon-Portugal

####### **Correspondence:** Ana Sousa (anaduarte.cbhs@gmail.com)

Introduction: Cohesinophaties are rare neurodevelopmental disorders characterized by distinctive facial dysmorphism, growth retardation, developmental delay/intellectual disability (DD/ID), and limb abnormalities. They originate from a dysfunction in the cohesin pathway, which enables chromosome segregation and regulates transcription. So far eight genes have been identified belonging to this pathway, including STAG1. We report a patient with a STAG1 intragenic deletion, thus contributing to reinforce the association between STAG1 haploinsufficiency and the cohesinopathy syndromic ID spectrum.

Case report: A 4-year-old boy was referred to our medical genetics department due to mild DD. Physical examination showed minor dysmorphism, namely arched and sparse eyebrows, downslanting palpebral fissures, wide nose with short columella, and thick lips. Family history was unremarkable. Array-CGH analysis detected a 206.24 Kb intragenic deletion, involving exons 2 to 12 of STAG1 – arr[GRCh37]3q22.3(136184662_136390897)x1. Parental studies are ongoing.

Discussion and Conclusion: Two different cohorts including a total of twenty individuals with syndromic ID and putative STAG1 haploinsufficiency were recently reported. Nine patients presented CNVs involving STAG1, including small intragenic deletions. All patients had mild to moderate DD/ID and common facial features. There was no clear phenotypic difference between patients with gene deletions and those with single nucleotide variants. Transmission of STAG1 variants was reported. Our patient shares common characteristics with these patients, reinforcing STAG1 as a new cohesinopathy gene that acts via a loss-of-function mechanism.

“Written, informed consent for publication was obtained from the patient [or parent/guardian for patients under 16]”

#### 6.P7 Upregulation of mir 155 in a 22q11.2 deletion syndrome cohort

##### Anelisa Gollo Dantas^1^, Marcos Leite Santoro^2^, Natália Nunes^1^, Malú Zamariolli^1^, Diogo Cordeiro Queiroz Soares^3^, Sintia Belangero^4^, Chong Ae Kim^3^, Maria Isabel Melaragno^1^

###### ^1^Universidade Federal De São Paulo, Morphology And Genetics, São Paulo-Brazil; ^2^Universidade Federal De São Paulo, Interdisciplinary Laboratory of Clinical Neurosciences (linc), São Paulo-Brazil; ^3^Instituto Da Criança, Universidade De São Paulo, Genetics Unit, São Paulo-Brazil; ^4^Universidade Federal De São Paulo, Morphology And Genetics, Interdisciplinary Laboratory of Clinical Neurosciences (linc), São Paulo-Brazil

####### **Correspondence:** Anelisa Gollo Dantas (anegdantas@gmail.com)

The 22q11.2 deletion syndrome (22q11.2DS) results from heterozygous deletions of chromosome 22. Even though most 22q11.2DS patients have similar size deletions, the phenotype is highly variable among individuals. A genetic mechanism that may play a role in the clinical heterogeneity is related to microRNAs (miRs) that can act as post-translational regulatory elements. A key component of miR biogenesis is the DGCR8, a gene disrupted by typical 22q11.2 deletions. Presuming that the phenotype observed is not only caused by the hemizygosity of the genes, we investigated the whole-genome gene expression, by microarray, in peripheral blood of 11 patients with 3 Mb deletion and controls. We observed that, among other genes differentially expressed, the mir-155 was upregulated in 22q11.2DS patients compared with controls (fold change 1.49; adjusted p-value 0.01). The mir-155 is mapped on 21q21.2 region and regulates more than 200 target genes (score >80, miRDB), that enrich most pathways related to T cell receptor and TNF signaling (adjusted p-value 0.002, Enrichr). Several studies showed that the mir-155 has a role in the differentiation of CD4+ T into T helper cells and it is essential for normal B cell differentiation. Increased levels of the mir-155 have also been observed in patients with trisomy 21. These patients also showed decreased levels of memory B cells as observed in adults 22q11.2DS patients, even though the mechanism involved in mir-155 expression may differ in both cases. Up to 75% of 22q11.2DS pediatric patients have immunodeficiency as a result of thymic hypoplasia, resulting in a deficiency in the production of T cells. We suggest that miRs should be considered as possible contributors for the immune phenotype, building a model of genetic causation that extends beyond protein-coding genes. Financial support: FAPESP, Brazil.

#### 6.P8 Cytogenomics NGS SNP array” for detection of genomic rearrangements on cell free DNA

##### Aldesia Provenzano^1^, Paolo Reho^1^, Debora Vergani^1^, Andrea La Barbera^1^, Lucia Tiberi^1^, Marilena Pantaleo^2^, Silvia Guarducci^2^, Orsetta Zuffardi^3^, Sabrina Giglio^1^

###### ^1^University of Florence, Medical Genetics Unit, Department of Experimental and Clinical Biomedical Sciences, Firenze-Italy; ^2^Meyer Children Hospital, Medical Genetics Unit, Firenze-Italy; ^3^University of Pavia, Medical Genetics Unit, Dpt. Molecular Medicine, Firenze-Italy

####### **Correspondence:** Aldesia Provenzano (aldesia.provenzano@unifi.it)

Array-based technology is used as first-tier test in pediatric settings however, do to the presence of low mosaicism level of genomic rearrangements, it is not possible to reach a specific diagnosis.

There are several diseases where mosaicism is known to occur, but the currently observed frequency is presumably an underestimation due to the difficulty of detecting changes in only a percentage of cells.

To date all SNP/CGH array applications are focused on tumor cell free DNA analysis to identify somatic CNVs for individualized cancer treatment, prognosis monitoring, although detecting early-stage cancers.

As cell free DNA (cfDNA) is present in healthy people giving us a complete picture of genomic assessment of all tissues, plasmatic cfDNA could be used to detect mosaicism also in "healthy people" without tumors, although normally cfDNA is present at low levels in the blood.

We applied cytogenomics NGS-SNP array analysis in cfDNA of some cancer free pediatric patients previously tested for a strong clinical suspicion of chromosomal disorders at mosaic and resulted negative at high resolution CGH-array on genomic DNA.

The analysis of cfDNA in different pathological constitutional cases and it might be a good candidate to study conditions characterized by the presence of mosaicism. Plasma DNA contains a treasure trove of genomic information and enhances its potential in clinical applications.

#### 6.P9 Detection of intron 22 inversion of the factor VIII gene in patients with severe hemophilia A using long distance PCR

##### Anita Pokupec Bilic, Kristina Crkvenac Gornik, Ivana Tonkovic Djurisevic, Morana Miklos

###### University Hospital Centre Zagreb, Division of Cytogenetics, Department of Laboratory Diagnostics, Zagreb-Croatia

####### **Correspondence:** Anita Pokupec Bilic (anita.pokupec.bilic@kbc-zagreb.hr)

Hemophilia A (HA) is the most severe inherited bleeding disorder that affects humans. A deficiency in FVIII clotting activity leads to this coagulopathy, which affects 1 in 5,000 males worldwide. This makes HA one of the most common X-linked inherited diseases. HA is expressed in a wide range of clinical severities and these differences associate with the type and location of the causative gene defect. Intron 22 inversion mutation of the F8 gene accounts for 50% of severe HA. Intron 22 inversions are the result of a homologous recombination between the int22h-1 region within the F8 locus and either int22h-2 (Inv22 type 2) or int22h-3 (Inv22 type 1), which lie approximately 400 kb distal to F8.

Genomic DNA samples of 80 severe HA patients were analysed in the course of molecular F8 gene diagnostics by long–distance PCR (LD-PCR) for the detection of intron 22 inversion. A combination of four primers (P, Q, A, B) was used in LD-PCR in order to distinguish between wildtype and hemi- or heterozygous intron 22 inversions. Two primers, P and Q, located within the F8 at positions −1,212 bp and +1,334 bp flanking int22h-1, when combined with two different primers, A and B, flanking the two extragenic repeats int22h-2 and int22h-3 each at −167 bp and +118 bp, yield segments PQ (12 kb) and AB (10 kb) in a hemizygous individual without Inv22 and segments PB (11 kb) and AQ (11 kb) along with the 10 kb AB segment from the intact extragenic homolog in a patient with the Inv22.

A screen for the presence of intron 22 and intron 1 inversions at the F8 gene in 101 severe HA patients revealed that 33 of patients showed intron 22 inversion. All patients were negative for intron 1 inversion. Analysis of int22 inversions should be the first-line test in DNA diagnostics, carrier detection and genetic counseling for severe hemophilia A, and could be particularly useful when there is no affected male family member, or when intervening family members are unavailable for testing. This method can also be used in prenatal diagnosis of the disease.

### 7. Other Cytogenomics topics

#### 7.P1 iPSC model of ring chromosome 13 syndrome

##### Anna Kashevarova^1^, Tatiana Nikitina^1^, Aleksei Menzorov^2^, Maria Gridina^3^, Anna Khabarova^3^, Yuliya Yakovleva^4^, Stanislav Vasilyev^1^, Marina Raspopova^5^, Maria Lopatkina^1^, Oleg Serov^6^, Igor Lebedev^1^

###### ^1^Research Institute of Medical Genetics, Tomsk Nrmc, Laboratory of Cytogenetics, Tomsk-Russia; ^2^Institute of Cytology and Genetics, Sb Ras, Section of The Cell Technology, Novosibirsk-Russia; ^3^Institute of Cytology and Genetics, Sb Ras, Section of The Genomic Mechanisms of Ontogenesis, Novosibirsk-Russia; ^4^Research Institute of Medical Genetics, Tomsk Nrmc, Clinical-Diagnostic Laboratory, Tomsk-Russia; ^5^Siberian State Medical University, Medical-Biological Faculty, Tomsk-Russia; ^6^Institute of Cytology and Genetics, Sb Ras, Department of Molecular Mechanisms of Ontogenesis, Novosibirsk-Russia

####### **Correspondence:** Anna Kashevarova (kashevarova.anna@gmail.com)

Generation of iPSCs with ring chromosome opens new possibilities for studying such aberration in vitro. Until recently it was believed that generating a ring chromosome cell model is impossible due to its adherent instability and loss during cultivation (Plona et al., 2016). We aimed to generate iPSC model for r(13) syndrome and characterize it using both cytogenetic and cytogenomic techniques. Four iPSC lines were established from skin fibroblasts of a patient with r(13). All lines showed a wide variety of karyotypes investigated by conventional cytogenetics and FISH. Two of them (iTAF6-6 and iTAF6-23) consisted mainly of cells with 46,XY,r(13) karyotype (65-98%). In the other two lines (iTAF6-25 and iTAF6-36) a low frequency of cells with r(13) and cells with 46,XY,-13,+mar and 45,XY,-13 karyotypes were observed at the early passages. However, cells with r(13) were prevalent in all lines by the later passages. Further all cell lines were analyzed using Agilent 180K+SNP arrays. As a result, 26 kb 13q31.3 duplication, 2.53 Mb 13q34 deletion and trisomy 17 were observed in iTAF6-6. Trisomy 17 often occurs during long-term iPSC culturing. In iTAF6-23 a single 2 Mb 13q34 deletion was identified. Multiple deletions were detected on chromosome 13 in iTAF6-25. A normal copy number region at 13q14.11-q14.13 5.3 Mb in size was found on chromosome 13, surrounded by deletions. In iTAF6-36 multiple deletions on chromosome 13 were detected. Overall iTAF6-25 and iTAF6-36 demonstrated cellular heterogeneity with pronounced fragmentation of r(13). The presence of the normal copy number region in iTAF6-25 suggests chromoanagenesis events, resembling chromoanasynthesis. In conclusion, three of four iPSC lines with r(13) were characterized by structural instability of chromosome 13. However, one cell line can be a good model for ring chromosome 13 investigation.

This study was supported by Russian Science Foundation, grant 16-15-10231.

#### 7.P2 Proteomic analysis of human isogenic cell lines with normal and ring chromosome 18

##### Anna Gabashvili^1^, Egor Vitushev^1^, Natalia Petushkova^2^, Viktor Zgoda^2^, Sergey Kiselev^1^, Andrey Lisitsa^2^

###### ^1^Vavilov Institute of General Genetics, Epigenetics, Moscow-Russia; ^2^Institute of Biomedical Chemistry, Proteomics and Mass-Spectrometry, Moscow-Russia

####### **Correspondence:** Anna Gabashvili (gabashvili.anna@gmail.com)

The ring chromosome 18 is a rare cytogenetic aberration causing the r(18) syndrome. In most individuals, r(18) changes the way the brain develops and works. Chromosome 18 ring is usually caused by spontaneous errors very early in the development of the embryo that appear to occur sporadically. While most people with r(18) have the ring chromosome in all of their body cells, some people are mosaic. People with r(18) mosaicism may have milder symptoms. It indicates that ring chromosome 18 usually occurs during zygote formation or shortly after blastomeres division. r(18) occurs when the tips of the chromosome join together to form a ring-shaped chromosome, the tips of both the q and the p arms are stochastically deleted. During embryonic development, pluripotent stem cells are present only transiently and quickly differentiate into various somatic cells through developmental process. However, it is possible to isolate ex vivo pluripotent human embryonic stem cells (hESC) from the inner cell mass of blastocyst and maintain them in laboratory. hESCs are capable of expanding indefinitely and differentiating into all human germ layers both in vitro and in vivo. Previously we established hESC (HESM01) line that after three dozens of passages acquired a mosaic karyotype possessing ring chromosome 18. Using cloning technique we isolated and characterized r(18)HESM01 cell line. Thus two isogenic hESC cell lines with the only differences in ring chromosome 18 were obtained. These cell line and their differentiated derivatives in various lineages were used for proteomic analysis. The proteomes of these cell lines and their differentiated derivatives in various lineages were analyzed using shotgun proteomic – high performance liquid chromatography connected with tandem mass spectrometry (LC-MS/MS). Mass spectra in raw format were processed using Progenesis LC-MS software (“Nonlinear Dynamics Ltd.”). Mascot engine (www.matrixscience.com) was used to perform a database search using a target decoy approach with FDR ≤ 1%. The Mascot data in xml format was re-imported into Progenesis LC-MS software for protein/peptide quantification. Few hundred proteins were revealed in our analysis some of them presumably reflected differences in genome structure. These pilot results demonstrate the efficiency of the LC-MS/MS approach for detecting the differences in the protein profiles of the isogenic HESM01 and r(18)HESM01 cell lines.

#### 7.P3 Somatic chromosomal mosaicism and instability in neurodevelopmental diseases from brain to blood and back again

##### Ivan Iourov^1^, Svetlana Vorsanova^2^, Maria Zelenova^1^, Oxana Kurinnaia^2^, Victor Kravets^2^, Alexei Kolotii^2^, Irina Demidova^2^, Kirill Vasin^1^, Vasili Sharonin^1^, Yuri Yurov^1^

###### ^1^Mental Health Research Center, Moscow, Russia, Yurov’s Laboratory of Molecular Genetics and Cytogenomics of The Brain, Moscow-Russia; ^2^Veltischev Research and Clinical Institute for Pediatrics of The Pirogov Russian National Research Medical University, Laboratory of Molecular Cytogenetics of Neuropsychiatric Diseases, Moscow-Russia

####### **Correspondence:** Ivan Iourov (ivan.iourov@gmail.com)

Somatic chromosomal mosaicism and instability confined to the brain have been repeatedly shown to be involved in the pathogenesis of neurodevelopmental diseases. Furthermore, somatic mosaicism is consistently identified in children with autism, intellectual disability and related disorders. Here, we have comparatively analyzed chromosome instability (CIN) and mosaicism in post-mortem brain samples of individuals with neurodevelopmental diseases (autism, intellectual disability and related disorders; n=37) and children with intellectual disability, autism, epilepsy and/or congenital malformations (n=10,000). Cytogenetic analysis, several FISH-based approaches (metaphase/interphase) and molecular karyotyping (SNP array) have been applied. We have found CIN in 5 brain samples (aneuploidy, interphase chromosome breaks and chromothripsis). Similar patterns of CIN have been identified in 435 children from the aforementioned cohort. Chromosomal mosaicism (aneuploidy of chromosomes 1, 15, 18, and X; additional rearranged chromosomes 14, 15 and X) has been found in 8 brain samples. Somatic chromosomal mosaicism (including those types of mosaicism detected in the diseased brain) has been uncovered in 399 children from the aforementioned cohort. Accordingly, it seems that neurodevelopmental diseases may be mediated by CIN and somatic mosaicism in 8.34% of cases. Additionally, all the results of molecular karyotyping have been processed using an original algorithm for pathway-based (network-based) classification of imbalanced genomic variations. Importantly, we have found that related molecular and cellular pathways are affected by imbalanced genomic variations in mitotic (blood) and post-mitotic (brain) tissues of individuals with neurodevelopmental diseases. In summary, this study seems to provide an empirical basis for targeting brain-specific causative cytogenomic variations by genomic and molecular cytogenetic analyses of biopsies. Supported by RFBR and CITMA according to the research project №18 515-34005.

#### 7.P4 Chitosan whey protein nanocarrier system to improve the intracellular oxidative stress of RAW264.7 cell by enhance cell proliferation and suppressed inflammatory response gene expression

##### Yi-Cheng Ho^1^, Chia-Hua Lin^1^, Ch Lin^1^, Jing-Chi Lin^2^, Fwu-Long Mi^3^

###### ^1^Chiayi University, Department of Bioagricultural Sciences, Chiayi-Taiwan; ^2^Chang Gung Memorial Hospital, Division of Allergy and Immunology and Rheumatology, Chiayi-Taiwan; ^3^Taipei Medical University, Department of Biochemistry and Molecular Cell Biology, School of Medicine, College of Medicine, Taipei-Taiwan

####### **Correspondence:** Yi-Cheng Ho (ichengho@mail.ncyu.edu.tw)

Chitosan is a positive charge natural polymer, its non-toxic to cells. In this work we develop a nanocarries system for the natural product delivery system with whey protein. The natural chemical compound of Ophiopogon Japonicus (OJP) aqueous extract after this nanocarrier system encapsulated, that could be enhance the cell proliferation and change the morphology of RAW264.7 cell. Our group using a oxidative response tracing dye DCFDA to detect the cell oxidative state. This nanocarries system can reduce the intracellular oxidative stress of the LPS-induced oxidative stress and the nano carrier system also to enhance the RAW264.7 cell proliferation. To understand this physiological phenomenon, the methods we use include flow cytometry, measuring cellular NO, measuring intracellular ROS, measuring cellular protein performance, and measuring the immunse response gene expression. We want to know more about the interaction between cells and nanoparticles, and use the FITC-labeling probe to locate the nanocarriers. The efficient nanoparticles can be efficiently combined with cells and have higher adhesion to cells. This cell screening platform can serve as a platform for efficient nanocarrier development.

#### 7.P5 Emergence of clonal chromosomal aberrations after reprogramming of Induced pluripotent stem cells

##### Isadora May Vaz^1^, Tamara Borgonovo^1^, Carolina Thome Barreto^1^, Juliana Ferreira Vasques^2^, Fernanda Gubert^2^, Maria Fernanda Toledo^2^, Rosalia Mendez-Otero^2^, Alexandra Cristina Senegaglia^1^, Carmen Lúcia Kuniyoshi Rebelatto^1^, Paulo Roberto Slud Brofman^1^

###### ^1^Pontifícia Universidade Católica Do Paraná, Núcleo De Tecnologia Celular, Curitiba-Brazil; ^2^Universidade Federal Do Rio De Janeiro, Biofísica, Rio De Janeiro-Brazil

####### **Correspondence:** Isadora May Vaz (isavaz92@yahoo.com.br)

Pluripotent stem cells can differentiate into cells of any of the three germ layers. Stem cell research is, therefore, interest in the cell therapy field and experimental models. Induced pluripotent stem cells (iPSCs) are generated from adult cells that have been reprogrammed to a condition of pluripotency. These can be used therapeutically to replace different types of tissues, without presenting ethical problems that are normally associated with the use of embryonic tissue. For clinical applications and studies in experimental models, it is important that the genetic stability is maintained after reprogramming. Chromosomal abnormalities may be an indication of genetic instability. Objective: To verify the frequency of chromosomal alterations before and after the reprogramming. Materials and methods: CytoTune™-iPS 2.0 Sendai Reprogramming Kit (reprogramming factors OCT3/4, KLF4, SOX2 and cMYC) was used to generate the iPSC cell lines. Cytogenetic analyses were performed using G-banded karyotyping. The metaphases were analyzed using the LUCIA program. Results: Two samples of mesenchymal stem cells from wharton's jelly were evaluated first and then generated two iPSCs lines each one. The mesenchymal sample ME310 analysis showed the karyotype 92,XXXX[4]/46,XX[29] and the iPSC cells analysis showed karyotypes 92,XXXX[20]. The mesenchymal sample ME311 showed a normal karyotype and, after reprogramming, trisomy of chromosome 12 in both of the iPSC cells lines. Conclusion: These results show how in the reprogramming and cultivation some clones can be selected and generate a completely altered lineage, as it happened in the ME310 case. There is also evidence that reprogramming may lead to the clonal cytogenetic changes emergence, even using cells that initially had normal karyotype, as in ME311.

#### 7.P6 Telomere length in mitotic spermatogonia and meiotic spermatocytes of patients with azoospermia

##### Anna Pendina^1^, Olga Efimova^1^, Anastasiia Petrovskaia-Kaminskaia^2^, Mikhail Krapivin^2^, Andrei Tikhonov^1^, Irina Mekina^3^, Evgeniia Komarova^3^, Vladislav Baranov^1^

###### ^1^D.o.ott Research Institute of Obstetrics, Gynecology and Reproductology, Department Of Genomic Medicine, St. Petersburg-Russia; ^2^St. Petersburg State University, Department of Genetics And Biotechnology, St. Petersburg-Russia; ^3^D.o.ott Research Institute Of Obstetrics, Gynecology And Reproductology, Department of Assisted Reproductive Technologies, St. Petersburg-Russia

####### **Correspondence:** Anna Pendina (pendina@mail.ru)

Telomeres are specialized terminal elements of linear chromosomes and are necessary to protect them from fusion and degradation. Telomeres are progressively shortened with each cell division and by environmental factors. To avoid inheritance of short telomeres by offspring, in spermatogenesis telomeres lengthen. We studied alterations of telomere length in spermatogenic cells of karyotypically normal patients aged 24-60 years with obstructive (n=9) and non-obstructive (n=5) azoospermia. Telomere length was measured by quantitative fluorescence in situ hybridization on cytogenetic preparations of mitotic spermatogonia (n=210) and meiotic spermatocytes I (n=157). Telomere length did not differ significantly between the patients with obstructive and non-obstructive azoospermia either in spermatogonia or spermatocytes I (P=0.7242 and P=0.9760, respectively). Telomere length in spermatogonia was significantly shorter compared to spermatocytes I (P*=0.0134). Telomere length in spermatogonia did not depend on the patient's age (r=-0.2228, P=0.4614) in contrast to that one in spermatocytes I where telomeres shortened with the increase of patient's age (r=-0.5396, P*=0.0489). Telomere length in spermatogonia correlated strongly positively with the ability of preimplantation embryos to develop up to the 5th day (r=0.7687, P*=0.0094). For telomere length in spermatocytes I a similar tendency was shown, however, it did not reach statistical significance (0.4644, P=0.1736). Our results suggest that in human spermatogenesis, telomeres lengthen from spermatogonia to spermatocytes I and that the longer are telomeres in spermatogonia, the more capable are preimplantation embryos to develop up to the 5th day.

Supported by RSF (grant №18-75-10046).

#### 7.P7 Genome wide 5 hydroxymethylcytosine and 5 methylcytosine patterns in human spermatogenic cells

##### Olga Efimova^1^, Anna Pendina^1^, Andrei Tikhonov^1^, Irina Mekina^2^, Evgeniia Komarova^2^, Mariia Mazilina^2^, Alexander Gzgzyan^2^, Igor Kogan^2^, Vladislav Baranov^1^

###### ^1^D.o. Ott Research Institute of Obstetrics, Gynecology and Reproductology, Department of Genomic Medicine, St. Petersburg-Russia; ^2^D.o. Ott Research Institute of Obstetrics, Gynecology and Reproductology, Department of Assisted Reproductive Technologies, St. Petersburg-Russia

####### **Correspondence:** Olga Efimova (efimova_o82@mail.ru)

DNA methylation and hydroxymethylation are major mechanisms of human genome reprogramming. We performed immunofluorescent analysis of 5-hydroxymethylcytosine and 5-methylcytosine patterns in human testicular spermatogenic cells from 15 azoospermic patients and ejaculated spermatozoa from 13 sperm donors and 37 patients from infertile couples. In contrast to DNA methylation, which was uniformly present throughout spermatogenesis, hydroxymethylation was either high or almost undetectable in both spermatogenic cells and ejaculated spermatozoa. On direct testicular cytogenetic preparations, 5-hydroxymethylcytosine was undetectable in mitotic and meiotic chromosomes, and was present exclusively in interphase spermatogonia Ad and in a minor spermatid population. The proportions of hydroxymethylated and non-hydroxymethylated diploid and haploid nuclei were established among 5000 nuclei from each sample. They were similar among samples, suggesting that the observed alterations of 5-hydroxymethylcytosine patterns in differentiating spermatogenic cells are programmed. In ejaculates, a few spermatozoa had high 5-hydroxymethylcytosine level, while in the other ones hydroxymethylation was almost undetectable. The percentage of highly hydroxymethylated spermatozoa varied strongly among individuals. In patients from infertile couples, it was higher than in sperm donors (P<0.0001) and varied in a wider range: 0.12-21.24% versus 0.02-0.46%. The percentage of highly hydroxymethylated (5-hydroxymethylcytosine-positive) spermatozoa correlated strongly negatively with the indicators of good semen quality – normal morphology (r=-0.567, P<0.0001) and normal head morphology (r=-0.609, P<0.0001) – and strongly positively with the indicator of poor semen quality: sperm DNA fragmentation (r=0.46, P=0.001). Thus, the immunocytochemically detected increase in hydroxymethylation in individual spermatozoa is associated with infertility in a couple and with deterioration of sperm parameters. We hypothesize that this increase is not programmed, but represent an induced abnormality and, therefore, it can be potentially used as a novel indicator of semen quality.

Supported by RSF (grant №18-75-10046).

#### 7.P8 Polystyrene nanoparticles can affect cell mitosis and compromise early mammalian embryo development

##### Vincenza Barbato, Riccardo Talevi, Roberto Gualtieri, Maria M Pallotta, Maddalena Di Nardo, Valentina Costanzo, Teresa Capriglione

###### University of Naples Federico Ii, Biology, Napoli-Italy

####### **Correspondence:** Teresa Capriglione (teresa.capriglione@unina.it)

A great interest surrounds the development of nanoparticles (NPs) for potential biomedical applications such as drug delivery and cancer therapy but the interplay and hazards between nanoscale materials and biological systems have not yet been completely clarified. In this study, Oviductal Epithelial Cells (BOEC) and bovine embryos were used as in vitro models to investigated whether cell mitosis and thus early mammalian embryo development could be affected by exposure to polystyrene nanoparticles. Analysis of the karyotype performed on PS-NP-BOEC did not show statistically significant chromosomal anomalies compared to the control, although it was observed a statistically significant higher presence of tetraploid metaphase plates in the BOEC cells treated with PS-NPs compared to the CTRL. In vitro fertilization experiments designed to understand whether PS-NPs treatment could affect bovine pre-implantation development showed that 8-cell embryo and blastocyst rates were significantly compromised in a dose-dependent manner after incubation with PS-NPs, however the quality of blastocysts in terms of mean cell number percentages of blastomeres with fragmented DNA were not significantly different in treated blastocysts compared to the corresponding control. The results of our study suggest PS-NP exposure can induce a cleavage delay which may affect the rate of mitosis in embryos with a lower developmental competence to reach blastocyst stage. Therefore, environmental release and subsequent accumulation of PS-NPs into organisms should be carefully monitored to prevent the occurrence of cytotoxic effects, which may compromise reproduction rates.

#### 7.P9 Pathologies of helicases and premature ageing study by derivation of induced pluripotent stem cells

##### Vincent Gatinois^1^, Romain Desprat^2^, Carole Corsini^3^, Jacques Puechberty^4^, Jean-Baptiste Gaillard^1^, Anouck Schneider^1^, Mylène Tharreau^1^, Thomas Guignard^1^, Lydiane Pichard^2^, Fabienne Becker^2^, Jean-Marc Lemaitre^2^, Franck Pellestor^1^

###### ^1^Hôpital A. De Villeneuve, Génétique Chromosomique, Montpellier-France; ^2^Hôpital St Eloi, Plateforme Safe-Ips, Montpellier-France; ^3^Hôpital A. De Villeneuve, Oncogénétique, Montpellier-France; ^4^Hôpital A. De Villeneuve, Génétique Médicale, Montpellier-France

####### **Correspondence:** Vincent Gatinois (v-gatinois@chu-montpellier.fr)

Helicases process the double-stranded DNA dissociation. They are involved in replication, DNA repair and maintenance of telomeres. In human, 3 helicases display mutations responsible for clinical syndromes: WRN for the Werner syndrome, BLM for the Bloom syndrome and RECQL4 for the Rothmund-Thomson syndrome. All these diseases cause premature ageing and high risk of cancer. Molecular and cellular mechanisms involved in these diseases are not well defined. Particularly, little is known concerning the link between genomic instability and ageing. During this project, we used blood samples and skin biopsies of affected patients to generate models by reprogramming cells to induced pluripotent stem cells (iPSCs). These cells have the advantage of self-renewing and could theoretically be differentiated into all cell types. At the same time, an iPSC senescence control was performed from cells of a Hutchinson-Gilford Progeria syndrome patient. iPSCs were characterized for pluripotency. With the aim to recapitulate these pathologies in vitro, we identified sets of cellular and molecular phenotypes. We also engaged differentiation of iPSCs in cell pathways close to the affected tissues in vivo. Finally, we studied the genomic stability of iPSCs and derived cells. We observed that Bloom cells are susceptible to frequent recombinations and are characterized by genome instability through all studied cell types. Werner cells showed an instability of telomere length. Finally, all premature ageing diseases displayed mitochondrial defects.

This work was supported by an internal public hospital fellowship “AOI Jeunes chercheurs, CHU de Montpellier.

#### 7.P10 Integrative assessment of genomic instability and anthropometric inflammatory and micro nutritional biomarkers in childhood obesity

##### Moonisah Usman^1^, Jessica Britto^1^, Ivona Bilkevic^1^, Martha Ford-Adams^2^, Simon Chapman^2^, Bethany Glassar^2^, Ashish Desai^3^, Murray Bain^4^, Ihab Tewfik^1^, Emanuela Volpi^1^

###### ^1^University of Westminster, Life Sciences, London-United Kingdom; ^2^Kings College Hospital NHS Foundation Trust, Pediatrics, London-United Kingdom; ^3^Kings College Hospital NHS Foundation Trust, Bariatric Surgery, London-United Kingdom; ^4^St. George's Hospital, Pediatrics, London-United Kingdom

####### **Correspondence:** Emanuela Volpi (e.volpi@westminster.ac.uk)

Obesity in children is one of the most serious public health challenges of the 21st century. The accumulation of adipose tissue is associated with a range of metabolic complications including diabetes, cardiovascular disease and dyslipidaemia. Epidemiological evidence links obesity in childhood with developing certain types of cancer later in life, but the mechanisms are only partially understood. It is postulated that chronic inflammation in obesity may promote genomic instability and drive carcinogenesis. Furthermore, micronutrient deficiencies may compromise DNA repair and further promote a pathological state.

The aim of this research has been to evaluate genomic instability and accelerated genomic ageing in childhood obesity through the deployment of minimally invasive testing approaches. Body composition, inflammation and micronutrient status were assessed in 132 children recruited from obesity clinics and local schools in London, and compared to biomarkers of genomic integrity and stability in urine, saliva and buccal cell samples.

The results demonstrate vitamin D deficiency and excessive C- reactive protein in children with obesity when compared to healthy weight controls. Furthermore, obesity in children corresponds with increased morphological nuclear abnormalities in buccal epithelial cells. Statistical analysis suggests that the percentage of body fat and level of vitamin D deficiency can predict the extent of early, pre-cancerous DNA damage assessed via the buccal cytome assay.

The findings of this research suggest that biomonitoring of ‘genome health’ in obese patients may contribute to inform prioritization and the severity of clinical intervention measures.

